# Time‐Restricted Feeding Alters Behavior in a Sex‐Specific Manner in Mice With Neuropathic Pain

**DOI:** 10.1002/mnfr.70479

**Published:** 2026-04-24

**Authors:** Manqing Wen, Wenjing Dai, Eija Kalso, Vinko Palada

**Affiliations:** ^1^ Department of Physiology, Faculty of Medicine University of Helsinki Helsinki Finland; ^2^ SleepWell Research Programme, Faculty of Medicine University of Helsinki Helsinki Finland; ^3^ Department of Pharmacology, Faculty of Medicine University of Helsinki Helsinki Finland; ^4^ Department of Anaesthesiology, Intensive Care and Pain Medicine Helsinki University Hospital Helsinki Finland

**Keywords:** anxiety, neuropathic pain, time‐restricted feeding, transcriptome

## Abstract

Evidence suggests that neuropathic pain (NP) and comorbid mood disorders are associated with circadian abnormalities. This suggests a role for chronotherapies, such as time‐restricted feeding (TRF), to alleviate pain and comorbid anxiety. We investigated the effects of TRF on pain and anxiety‐related behaviours in spared nerve injury (SNI) mice with NP and diurnal changes in the hypothalamus transcriptome, an important hub for modulating fear and anxiety. SNI male and female C57BL/6JRj mice received TRF during the dark active phase or ad libitum feeding (ALF) for three weeks post‐surgery. Behavioral tests, von Frey, hot plate, light‐dark box (LDT), and open field (OFT), were performed at baseline and post‐surgery; hypothalamus tissues were dissected in the morning (zeitgeber time (ZT) 2–6) and afternoon (ZT 8–12). Male, but not female, SNI mice under TRF showed significantly reduced anxiety‐like behaviors (LDT: *p *= 0.0259, Cohen's d = 1.245; OFT: *p *= 0.0054, Cohen's d = 1.643). Differentially expressed gene (DEG) analysis identified 33 DEGs in the hypothalamus in male SNI mice between TRF and ALF in the afternoon, with enriched anxiety‐related, mitochondrial, and circadian genes. These findings support TRF as a potential therapeutic approach to alleviate comorbid anxiety in NP.

## Graphical abstract

Time‐restricted feeding (TRF) alleviates anxiety‐like behavior in male mice with neuropathic pain (NP), but not in females. Upon nerve injury, TRF altered the diurnal expression of transcripts in the hypothalamus. These findings suggest that TRF should be explored as a new therapeutic approach to alleviate the comorbid anxiety in NP.

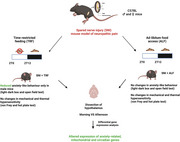

**Abbreviations**
ALF: Ad libitum food access
DEG: differentially expressed gene
DVG: differentially variable gene
LDT: light‐dark box test
NP: neuropathic pain
OFT: open field test
SNI: spared nerve injury
TRF: time‐restricted feeding
ZT: zeitgeber time

## 1 Introduction

Neuropathic pain (NP), pain caused by nerve injury, is characterized by hypersensitivity symptoms such as allodynia and hyperalgesia that negatively affect quality of life [1]. Management of NP is often complicated by the presence of comorbid mood disorders. It has been estimated that 39% of NP patients suffer from anxiety disorders during their lifetime [2]. Therefore, understanding the mechanisms underlying this comorbidity is crucial for finding more effective treatments.

The circadian system synchronizes biological clocks with the environment [3], and disruptions in the alignment of rhythms to external cues are associated with several clinical conditions [4], including the regulation of pain and anxiety [5, 6, 7]. Circadian disruption upon night shift work is associated with increased pain responses in nurses and elevated cold pain sensitivity in night shift workers [8, 9]. Similarly, nurses with shift work disorder showed increased anxiety scores [10]. We have recently shown that painful peripheral nerve injury in mice disrupts the rhythmic expression of hundreds of transcripts in pain‐regulating sensory tissues, indicating circadian disruption [11]. In addition, rhythmic expression of melatonin receptors in the hypothalamus is altered in sciatic nerve‐ligated male mice with NP [12]. Similar changes following circadian disruption are also found as increased anxiety‐like behaviors in young mice subjected to dim light [13]. These studies emphasize the relationship between circadian disruption and comorbid pain and anxiety.

Some of the best characterized physiological processes under circadian control are the feeding and fasting cycles [14], and these rhythms are absent in clock‐deficient mice [15]. Time‐restricted feeding (TRF), restricting access to food to defined times of the day, is one of the strategies to investigate the effects of chrononutrition as a treatment for various diseases [14], including neurological disorders. A previous study showed that intermittent fasting promotes nerve regeneration in mice after nerve injury [16]. However, how TRF affects pain intensity in NP is currently completely unexplored. Since TRF was recently shown to modulate anxiety in rats [17], we postulated that pain intensity and anxiety levels would also be reduced upon TRF in the spared nerve injury (SNI) mouse model of NP. Since it is well established that the prevalence and underlying mechanisms of anxiety and pain are quite different between the sexes [18, 19], we aimed to assess the effects of TRF on both sexes upon SNI.

The hypothalamus is an important hub in the modulation of fear and anxiety [20, 21, 22, 23], and its suprachiasmatic nucleus contains the light‐responsive neurons that are master regulators of mammalian circadian clocks [22]. To further investigate the molecular mechanisms that are modulated by TRF after nerve injury, we investigated the impact of TRF on diurnal changes in the expression of hypothalamic transcripts in TRF‐treated mice versus controls with ad libitum food (ALF) access.

## 2 Experimental Section

### 2.1 Animals

**FIGURE 1:**
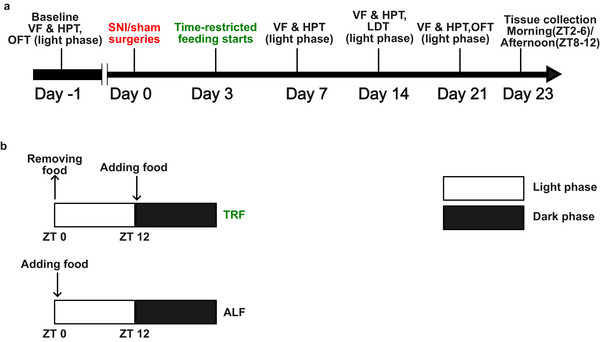
Experimental protocol. (a) Overview of behavioral tests. Pain behavioral testing (VF and HPT) was performed at baseline (day ‐1) before surgeries (day 0) and on post‐surgical days 3, 7, 14, and 21. Anxiety‐like behavior was accessed by OFT at baseline (day 1) before the surgeries (day 0) and on post‐surgical day 21, and by LDT on post‐surgical day 14. Abbreviations: VF, von Frey test; HPT, hot plate test; OFT, open field test; LDT, light‐dark box test; SNI, spared nerve injury. (b) Overview of feeding protocols. During TRF, mice only had food access during the dark phase. During ALF, mice received ad libitum food access. Abbreviations: TRF, time‐restricted feeding; ALF, ad libitum feeding.

Experiments were performed with C57BL/6JRj mice (11 weeks old, Janvier Labs, France), housed in a pathogen‐free facility at the University of Helsinki. The mice were kept in a 12 h:12 h light/dark cycle (lights on: zeitgeber time (ZT) 0; off: ZT 12). A total of 64 mice (8/group/sex) were randomly assigned into four experimental groups: (1) SNI TRF, (2) SNI ALF, (3) sham TRF, and (4) sham ALF. SNI and sham‐operated TRF mice were placed under TRF protocol on post‐surgical day 4, while the ALF groups had ad libitum access to standard food during the whole protocol (Envigo, Huntingdon, United Kingdom) (Figure 1a,b). Mice under TRF had access to food only during the dark active phase, as previously described [24]. Behavioral testing was performed at the same time of the day to minimize the effects of circadian variation on the results. Von Frey testing was performed during ZT2‐ZT4, the hot plate test during ZT7‐ZT9, and the light‐dark box test (LDT) and open field test (OFT) during ZT2‐ZT5. All animal procedures were approved by the Regional State Administrative Agency for Southern Finland (ethical approval number ESAVI/4935/2024). The weight of mice was monitored post‐surgery with a 15% weight drop as criterion for euthanasia (Figure S1) (not met for any mouse).

### 2.2 Spared Nerve Injury

NP was induced in the left hind paw by SNI surgeries [25]. First, 5% isoflurane was used to deeply anaesthetize mice in a chamber (Attane Vet, Piramal Critical Care B.V, The Netherlands), while anesthesia was maintained during surgery by 2% isoflurane through a mask. The muscle layer was separated to reveal the sciatic nerve, and the common peroneal and tibial branches of the sciatic nerve were ligated by surgical knots using 6.0 suture (B. Braun, Melsungen, Germany). Next, 2 mm of the nerves were cut and removed, while the sural nerve remained intact. Sham surgeries were performed using the same procedure but without nerve ligation.

### 2.3 Pain Behavioral Testing

Behavioral tests were performed at baseline before the surgeries and on post‐surgical days 3, 7, 14, and 21 (Figure 1a). Mice were habituated in the behavioral room in a red transparent cylinder for 30–60 min for two consecutive days before testing. Mechanical thresholds were measured by von Frey tests [25]: a monofilament (North Coast Medical, Inc., Morgan Hill, California, USA) was applied to the lateral aspect of the plantar surface five times at intervals of 10 s for 2 min. Sudden paw withdrawal, flinching, lifting, or shaking was regarded as a positive response. The mechanical withdrawal threshold was defined as the lowest force which elicited positive responses to three out of five stimuli [25]. Thermal allodynia was tested by the hot plate test (Ugo Basile, Gemonio, Varese, Italy), placing the mice individually on a 52°C heated metal plate surrounded by a transparent cylinder [26]. The latency time of the first typical nociceptive reactions, jumping, flinching, and licking of the hind paw, was recorded. The cutoff time of the hot plate was 40 s to prevent tissue damage.

### 2.4 Light‐Dark Box and Open Field Tests

The animals were transferred to the behavioral room for habituation 1 h before the tests. Light‐dark box (LDT) and open field (OFT) tests were performed in 42 cm × 42 cm behavioral chambers (Med Associates, USA) to measure anxiety‐related behaviors, as previously described [27]. The LDT was performed on day 14 post‐SNI/sham surgeries, and the OFT was performed at baseline before the surgeries and on post‐surgical day 21. In the LDT, the mice were placed in the dark compartment and allowed to move freely for 10 min. In the OFT, mice were placed in the center of arena and allowed to move freely for 15 min. The center area refers to the central part of the arena with a length and width of 21 cm. The times spent in the dark and light chambers in the LDT, and in the central and peripheral parts of the arena in the OFT were recorded, and the data were analysed by Activity Monitor software (Med Associates, USA) to measure changes in anxiety levels. Between tests, the chamber was cleaned to minimize the effect of odor from previous animals.

### 2.5 Tissue Collection and RNA Sequencing

At the end of the TRF protocol and based on observed sex‐specific effects of TRF on anxiety‐like behavior, hypothalamus tissues were dissected from male SNI and sham mice in the morning (ZT 2‐6) and afternoon (ZT 8‐12). Total RNA was extracted by TRIzol reagent (Thermo Fisher Scientific, Waltham, Massachusetts, USA) following the manufacturer's instructions. The purity and quantity of RNA and quality ratios 260/280 (mean 1.88) and 260/230 (mean 1.72) were measured by NanoDrop 2000/2000c (Thermo Fisher Scientific, Waltham, Massachusetts, USA).

A total of 1000 ng of messenger RNA from each sample was purified by poly‐T oligo‐attached magnetic beads and fragmented. The random hexamer primers were used to synthesize complementary DNA, which was end‐repaired and adapter‐ligated. The library was constructed and checked with Qubit, quantified by real‐time PCR, and analyzed for size distribution by Bioanalyser. Quantified libraries were pooled and sequenced on Illumina platform (Novogene, Cambridge, England). Low‐quality reads and reads with adapters were removed. HISAT2 (v2.2.1) [28] was used to align filtered reads to the reference genome mm39, and the number of Fragments Per Kilobase of transcript sequence per Millions base pairs sequenced (FPKM) was calculated by FeatureCounts (v2.0.6) [29].

### 2.6 Bioinformatic Analysis

DESeq2 (v1.42.0) was used to identify differentially expressed genes (DEG) between TRF and ALF groups [30]. Transcripts with |foldchange| > 1.1 and adjusted *p*‐values < 0.05 according to the Benjamini–Hochberg procedure were considered as differentially expressed and were visualized by volcano plots.

The function varFit from R package missMethyl (v1.38.0) was applied to identify nonrhythmic transcripts with significant change in variability in gene expression [31]. The variability of gene expression was by the absolute deviation from the mean expression of each gene within the group. A linear model with empirical Bayes shrinkage was then applied. The *p* < 0.01 was used as standard to identify differentially variable genes (DVG) with significant group difference in variability, and the ratios of the log change in group variance (LogVarRatios) were calculated, as previously described [32].

### 2.7 Statistics

The statistical tests were performed in Prism (v9.5.1, GraphPad Software Inc, San Diego, USA). Results of von Frey and hot plate tests are presented as means ± SD, and LDT and OFT results as means ± SEM. Two‐way ANOVA followed by multiple comparisons using Tukey's multiple comparisons was used to analyze the data from von Frey and hot plate tests. Unpaired *t*‐tests were used to analyze the data from LDT and OFT. The sequencing data were analyzed and plotted in R as described in Section 2.6. The sample size was chosen to reliably investigate the effects of TRF on behavior and diurnal transcriptomes, while minimizing the use of animals according to the 3R principles. For the behavioral testing, sample size was determined based on a previous study with similar experimental setup [33]. For behavioral tests, sample size (*n* = 8 per group) exceeds the minimum required for large effects (Cohen's d = 0.8, α = 0.05, power = 0.8), as calculated with G*Power (v3.1). For RNA‐sequencing, n = 4 per group was chosen based on previous similar studies where the selected sample size was sufficient to reliably detect the transcriptomic changes in mice upon SNI or intermittent fasting [11, 34]. The effect sizes for behavioral tests were calculated following Cohen´s d formula, where *d* = 0.2 represents small, *d* = 0.5 medium, and *d* = 0.8 large effect sizes [33].

## 3 Results

### 3.1 Time‐Restricted Feeding (TRF) Does Not Alter the Mechanical and Thermal Sensory Thresholds Upon Spared Nerve Injury (SNI)

**FIGURE 2:**
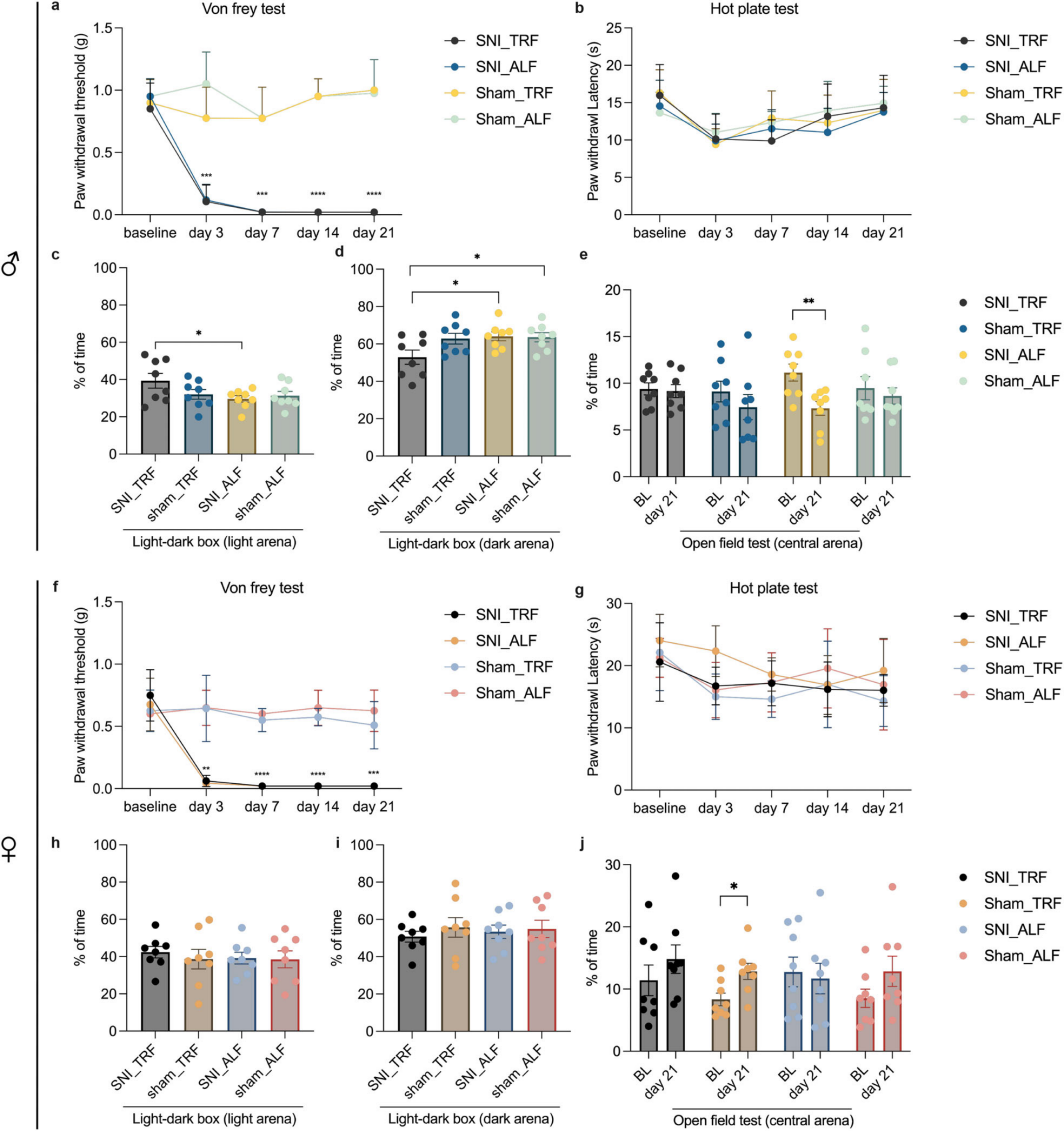
TRF alters behavior in male mice with NP. (a, f) Changes in paw withdrawal thresholds during Von Frey test for male (a) or female mice (f). (Two‐way ANOVA) (b, g) Changes in thermal thresholds during hot plate test in male (b) or female mice (g). (Two‐way ANOVA). Data are presented as mean ± SD, and error bars represent SD. (c‐d, h‐i) Percentage of time spent in the light arena (c/h) and dark arena (d/j) during the light‐dark box test for male/female mice, respectively. (Unpaired *t*‐test) (e, j) Percentage of time spent in central arena during open field test for male (e) or female mice (j). (Unpaired *t*‐test). Data are presented as mean ± SEM, error bars represent SEM. For both behavioral tests, *n* = 8 per group (biological replicates). *p < 0.05, **p < 0.01, ***p < 0.001, ****p < 0.0001. Abbreviations: TRF, time‐restricted feeding; ALF, ad libitum feeding; SNI, spared nerve injury.

Significant reduction in mechanical sensory thresholds was observed in the von Frey test in male and female SNI mice from post‐surgical day 3 compared to sham‐operated controls. No significant differences in mechanical thresholds were observed between TRF and ALF from day 7 onwards (*p* = 0.7545, CI: −0.01078 to 0.005775) in SNI mice (Figure 2a,f). Additionally, paw withdrawal latencies were not altered in SNI mice in the hot plate test compared with the controls in both sexes (males: *p* = 0.6375, CI: −5.526 to 2.301; females: *p* = 0.8028, CI: −6.107 to 3.232) (Figure 2b,g). These results indicate that TRF does not alter mechanical and thermal sensory thresholds following SNI.

### 3.2 TRF Alters Behavior in Male, but Not in Female SNI Mice

Male SNI mice under TRF spent more time in the light arena (*p *= 0.0408, Cohen's d = 1.127) and less time in the dark arena (*p *= 0.0259, Cohen's d = 1.245) during LDT compared with SNI mice in the ALF group (Figure 2c,d), while no significant differences were observed in female mice (Figure 2h,i). In addition, the time spent in the center of the arena during OFT was significantly reduced in SNI ALF male mice (*p *= 0.0054, Cohen's d = 1.643) on day 21 post‐surgery compared with baseline, which was not observed in SNI mice under TRF, indicating altered behavior in SNI TRF mice compared to ALF controls (Figure 2e). In female mice, there was a significant increase in the duration of time in the centre of the arena in control sham mice under TRF (*p *= 0.0158, Cohen's d = 1.372) on day 21 post‐surgery compared with baseline (Figure 2j), but no changes were observed in the SNI groups. These results suggest that TRF alters behaviour in the LDT and OFT under NP conditions exclusively in male mice.

In the LDT, there were no significant differences in the number of entries between compartments among the four groups for either sex (Figure S2a,c). Similarly, in the OFT, no significant differences in total distance were found between the TRF and ALF groups on Day 21 (Figure S2b,d). These results indicate that the TRF schedule did not negatively impact the behavioral readouts through alterations in locomotion or exploratory drive.

### 3.3 Neuropathic Pain Significantly Alters the Hypothalamic Transcriptome

**FIGURE 3:**
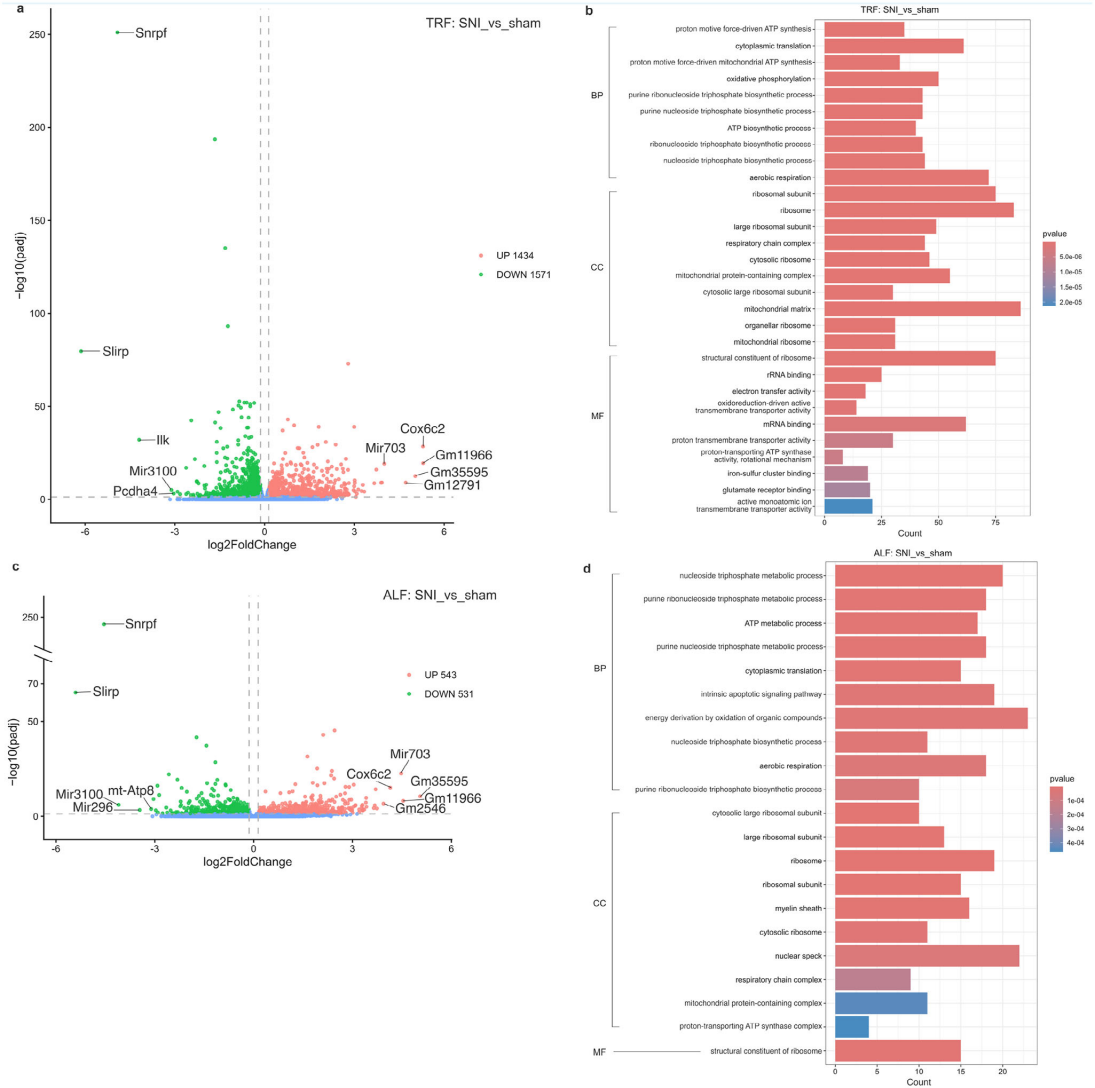
Differentially expressed genes (DEGs) in the hypothalamus between SNI and sham mice. (a, c) Volcano plots of DEGs in the comparison of SNI versus sham mice under the TRF (a) and the ALF schedule (c). (b, d) Gene ontology (GO) annotations of DEGs from the comparison of SNI_TRF versus sham_TRF (b) and SNI_ALF versus sham_ALF (d). The horizontal dashed line represents y  = −log_10_(0.05). Two vertical dashed lines represent x = log_2_(10/11) and x = log_2_(1.1), respectively. For RNA‐seq, *n* = 4 per group (biological replicates). Abbreviations: TRF, time‐restricted feeding; ALF, ad libitum feeding; BP, biological process; CC, cellular component; MF, molecular function; SNI, spared nerve injury.

Since the behavioral results showed that TRF alters behavior in the LDT and OFT under NP conditions only in males, hypothalamus tissues were collected from male SNI and sham mice under TRF or ALF protocols during the morning (ZT 2‐6) and afternoon (ZT 8‐12) for RNA‐sequencing. Differential expression analysis identified 3005 DEGs between SNI and sham mice under the TRF, with 1434 upregulated and 1571 downregulated transcripts (Figure 3a, Tables S1 and S2). Among these, 11 transcripts were reported to be associated with pain regulation, including nuclear receptor subfamily two group F member 6 (*Nr2f6*) [35], alpha tubulin acetyltransferase 1 (*Atat1*) [36], glutamate receptor ionotropic NMDA1 (*Grin1*) [37], cyclin dependent kinase 5 (*Cdk5*) [38], Eph receptor B1 (*Ephb1*) [39], neuroligin 2 (*Nlgn2*) [40], sodium channel voltage‐gated type III alpha (*Scn3a*) [41], glutamate receptor ionotropic NMDA2A (*Grin2a*) [42], C‐X‐C motif chemokine ligand 12 (*Cxcl12*) [43], mitogen‐activated protein kinase 3 (*Mapk3*) [44], and Kv channel interacting protein 3, calsenilin (*Kcnip3*) [45]. Five transcripts were related to regulation of feeding; eukaryotic translation initiation factor two alpha kinase 4 (*Eif2ak4*) [46], ankyrin repeat domain 26 (*Ankrd26*) [47], apelin (*Apln*) [48], melanocortin 2 receptor accessory protein 2 (*Mrap2*) [49], and 5‐hydroxytryptamine (serotonin) receptor 1B (*Htr1b*) [50]. Additionally, 24 transcripts were associated with circadian regulation, and 228 transcripts were involved in mitochondria‐related processes (Table S3).

For the comparison between SNI and sham mice under the ALF, there were 1074 DEGs in total, with 543 upregulated and 531 downregulated transcripts (Figure 3c). Among these, *Nr2f6* [35] was associated with pain regulation, and pro‐melanin‐concentrating hormone (*Pmch*) [51] was related to feeding behavior. In addition, there were eight DEGs associated with circadian regulation, and 55 DEGs associated with mitochondrial function (Table S3). Gene ontology analysis reveals that the DEGs from both comparisons are enriched in terms associated with ribosome and mitochondrial‐related processes (Figure 3b,d). Collectively, the high number of DEGs identified across both feeding schedules suggests that the SNI procedure significantly impacts the hypothalamic transcriptome and exerts a strong influence on hypothalamic gene expression.

### 3.4 TRF Alters the Diurnal Expression of Transcripts in Hypothalamus of Male Mice Upon Nerve Injury

**FIGURE 4:**
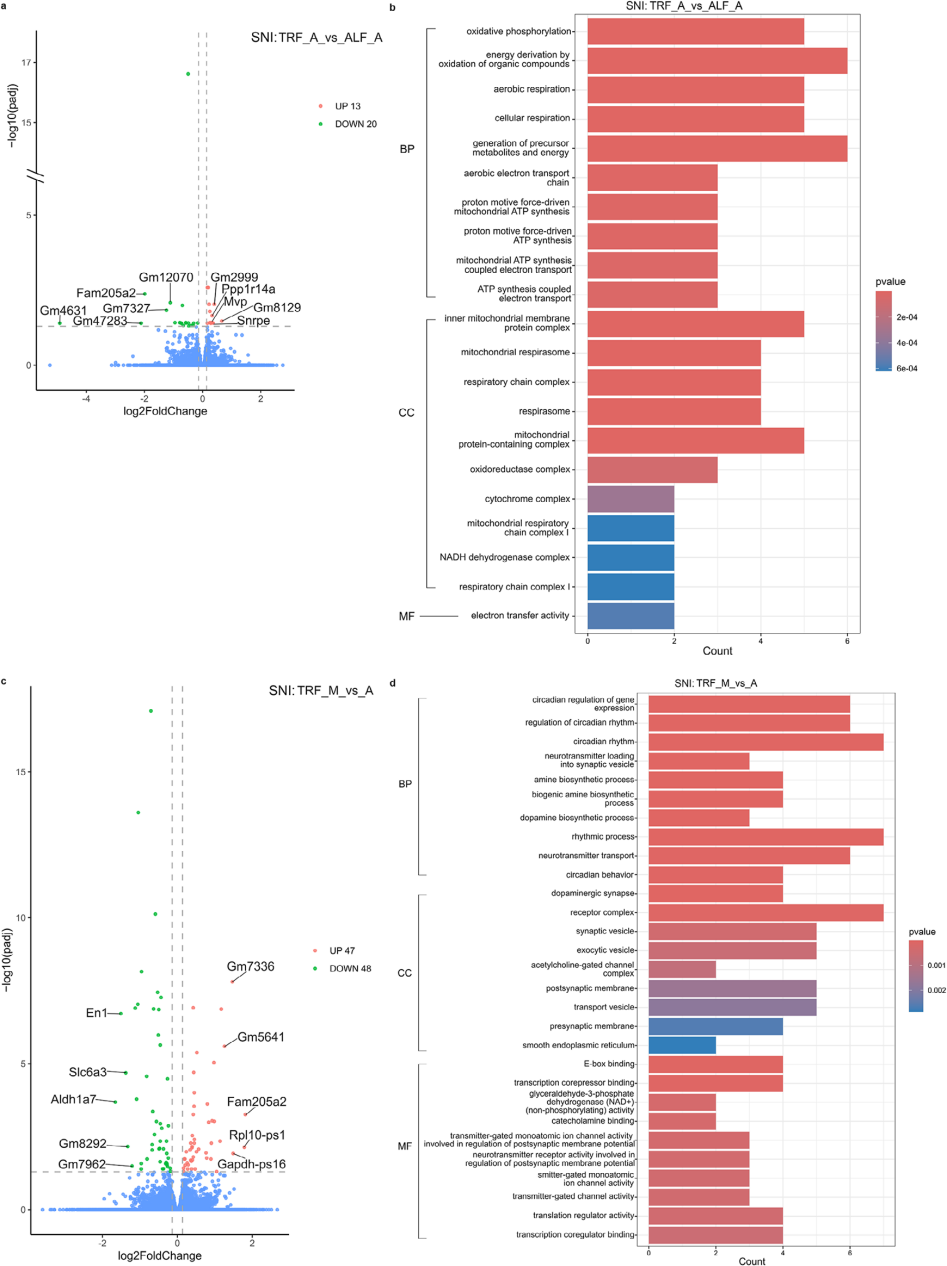
Differential expression analysis in SNI mice. (a, c) Volcano plot of DEGs in SNI mice for the comparison of TRF_A versus ALF_A (a) and TRF_M versus TRF_A (c). (b, d) Gene ontology annotations of DEGs from the comparison of TRF_A versus ALF_A (b) and TRF_M versus TRF_A (d). For RNA‐seq, *n* = 4 per group (biological replicates). Abbreviations: M = morning, A = afternoon.

To characterize the effects of TRF on diurnal changes in the hypothalamic transcriptome, we first examined how the feeding schedules modulate gene expression in the afternoon (ZT 8‐12). In SNI mice, comparing the TRF group to ALF controls revealed 33 DEGs in HT, 13 upregulated and 20 downregulated (Figure 4a). Five of these DEGs are linked to mitochondrial pathways, including ubiquinol‐cytochrome c reductase complex III subunit XI (*Uqcr11*) [52], ATP synthase H+ transporting mitochondrial F0 complex subunit F (*Atp5j*) [53], NADH:ubiquinone oxidoreductase subunit A3 (*Ndufa3*) [54], mitochondrially encoded cytochrome c oxidase III (*mt‐Co3*) [55], and NADH:ubiquinone oxidoreductase subunit S6B (*Ndufs6*) [56]. Furthermore, Uqcr11 and nuclear receptor subfamily four group A member 1 (*Nr4a1*) are associated with the regulation of sleep deprivation, with *Uqcr11* also regulating fasting in mice [57, 58, 59]. Additionally, five DEGs were previously linked to the regulation of mood disorders, including *Nr4a1* [60, 61], major vault protein (*Mvp*) [62], cell adhesion molecule 4 (*Cadm4*) [63], glycogen synthase kinase 3 alpha (*Gsk3a*) [64], and *mt‐Co3* [55]. Enrichment analysis indicates that these DEGs are associated with ATP synthesis, cellular respiration, and mitochondrial functions (Figures 4b and S3a,b).

**FIGURE 5:**
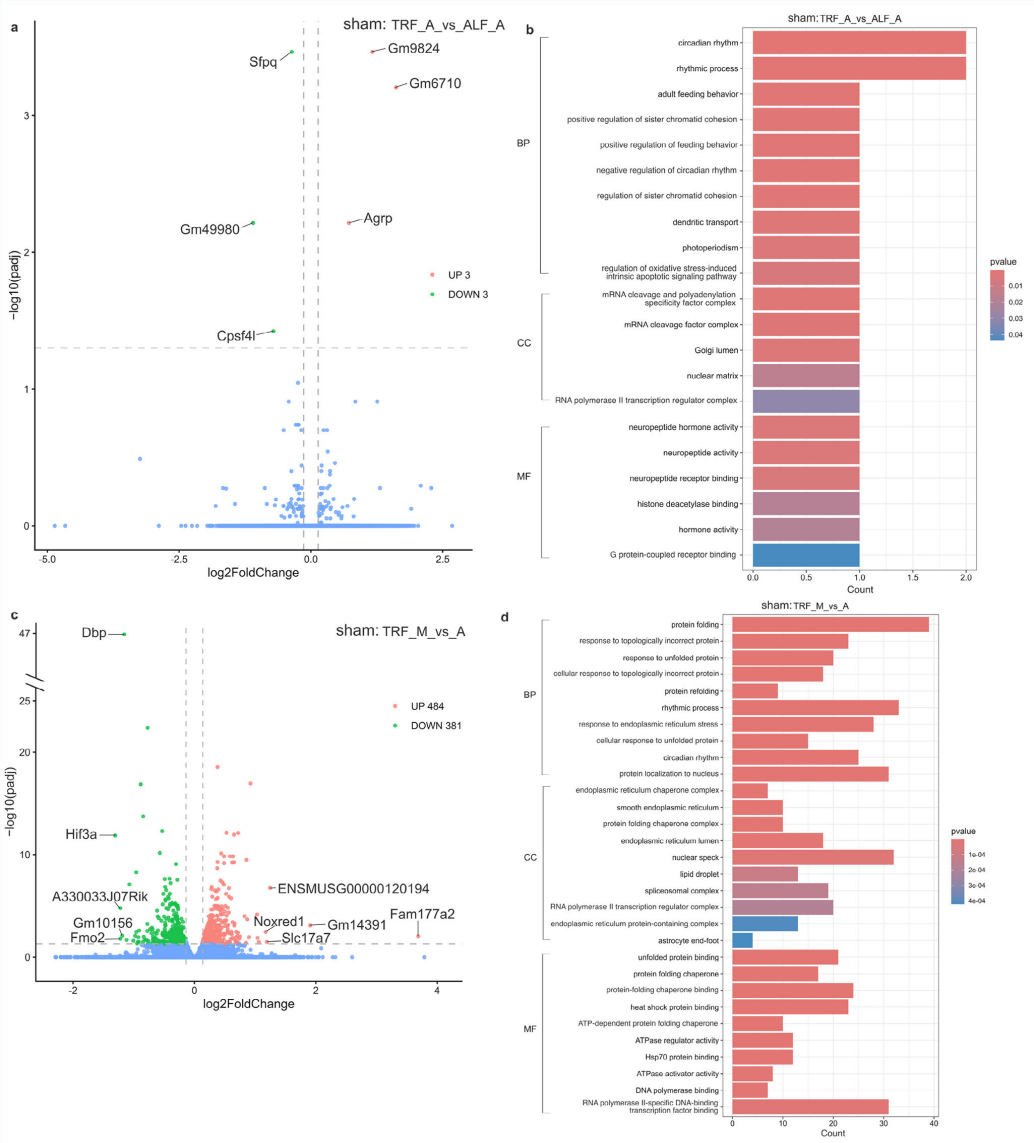
Differential expression analysis in sham mice. (a, c) Volcano plots of DEGs in sham mice for the comparison of TRF_A versus ALF_A (a) and TRF_M versus TRF_A (c). (b, d) Gene ontology (GO) annotations of DEGs from the comparison of TRF_A versus ALF_A (b) and TRF_M versus TRF_A (d). For RNA‐seq, *n* = 4 per group (biological replicates). Abbreviations: ALF, ad libitum feeding; M, morning; A, afternoon; TRF, time‐restricted feeding.

The impact of the feeding schedule was less pronounced in sham‐operated mice, where only six DEGs were identified between the TRF and ALF groups in the afternoon (Figure 5a). Among these, splicing factor proline/glutamine rich (*Sfpq*) [65] has been linked to circadian rhythms, while agouti‐related protein (*AgRP*) [66] regulates feeding (Figure 5b). Importantly, the effects of TRF appear to be highly time‐dependent. No hypothalamic DEGs were observed between the SNI TRF and ALF groups in the morning (ZT 2‐6), and only one transcript, H1.2 linker histone cluster member (*H1f2*), was downregulated in sham mice during the same period (Table S1).

We then shifted our analysis to diurnal variation, comparing morning (ZT 2‐6) and afternoon (ZT 8‐12) expression within each group. In SNI mice under the TRF protocol, we identified 95 diurnal DEGs in HT, 47 upregulated and 48 downregulated (Figure 4c). Seven of these genes were previously reported to be associated with circadian regulation, including nuclear receptor subfamily 1 group D member 1 (*Nr1d1*), nuclear receptor subfamily 1 group D member 2 (*Nr1d2*), period 1 (*Per1*), period 2 (*Per2*), period 3 (*Per3*), basic helix‐loop‐helix ARNT like 1 (*Bmal1*), and cold‐inducible RNA binding protein (*Cirbp*) [67, 68, 69]. Three DEGs, *Nr1d1* [70], cytoplasmic polyadenylation element binding protein 1 (*Cpeb1*) [71], and LIM homeobox transcription factor 1 alpha (*Lmx1a*) [72], were previously linked to mitochondrial regulation. Furthermore, seven DEGs, including gamma‐aminobutyric acid (GABA) A receptor subunit alpha 2 (*Gabra2*) [73], *Nr1d1* [74], *Nr1d2* [75], *Per1* [76], solute carrier family 6 member 3 (*Slc6a3*) [77, 78], *Cpeb1* [69], and *Bmal1* [79], were reported to be involved in anxiety. The gene ontology analysis showed that these diurnal DEGs were enriched in regulation of circadian rhythms (Figures 4d and S3c).

A significantly more robust diurnal shift was observed in sham mice under the TRF protocol, with 865 diurnal DEGs, 484 upregulated, and 381 downregulated (Figure 5c). Among these, six DEGs, *Atat1*, ATP synthase C subunit lysine N‐methyltransferase (*Atpsckmt*) [80], potassium voltage‐gated channel shaker‐related subfamily member 2 (*Kcna2*) [81], arrestin beta 2 (*Arrb2*) [82], *Cxcl12*, and transmembrane protein 100 (*Tmem100*), were previously linked to pain regulation. Three DEGs, including corticotropin‐releasing hormone receptor 1 (*Crhr1*) [83], *Apln*, and *Htr1b*, are associated with the regulation of feeding. Additionally, 24 DEGs were related to circadian regulation, and 68 were related to mitochondrial processes (Table S3). Enrichment analysis showed that these transcripts were associated with protein folding and circadian rhythms (Figures 5d and S4a,b).

In contrast, diurnal variation was markedly reduced in mice under the ALF protocol. Only a single DEG, stromal cell‐derived factor 2‐like 1 (*Sdf2l1*), showed altered diurnal expression in SNI ALF mice, and only 29 DEGs were identified in sham ALF mice (Table S1). These results indicate that the TRF protocol significantly shifts the diurnal expression patterns within the hypothalamus.

### 3.5 TRF Alters the Variability of Transcripts in Hypothalamus of Male Mice

**FIGURE 6:**
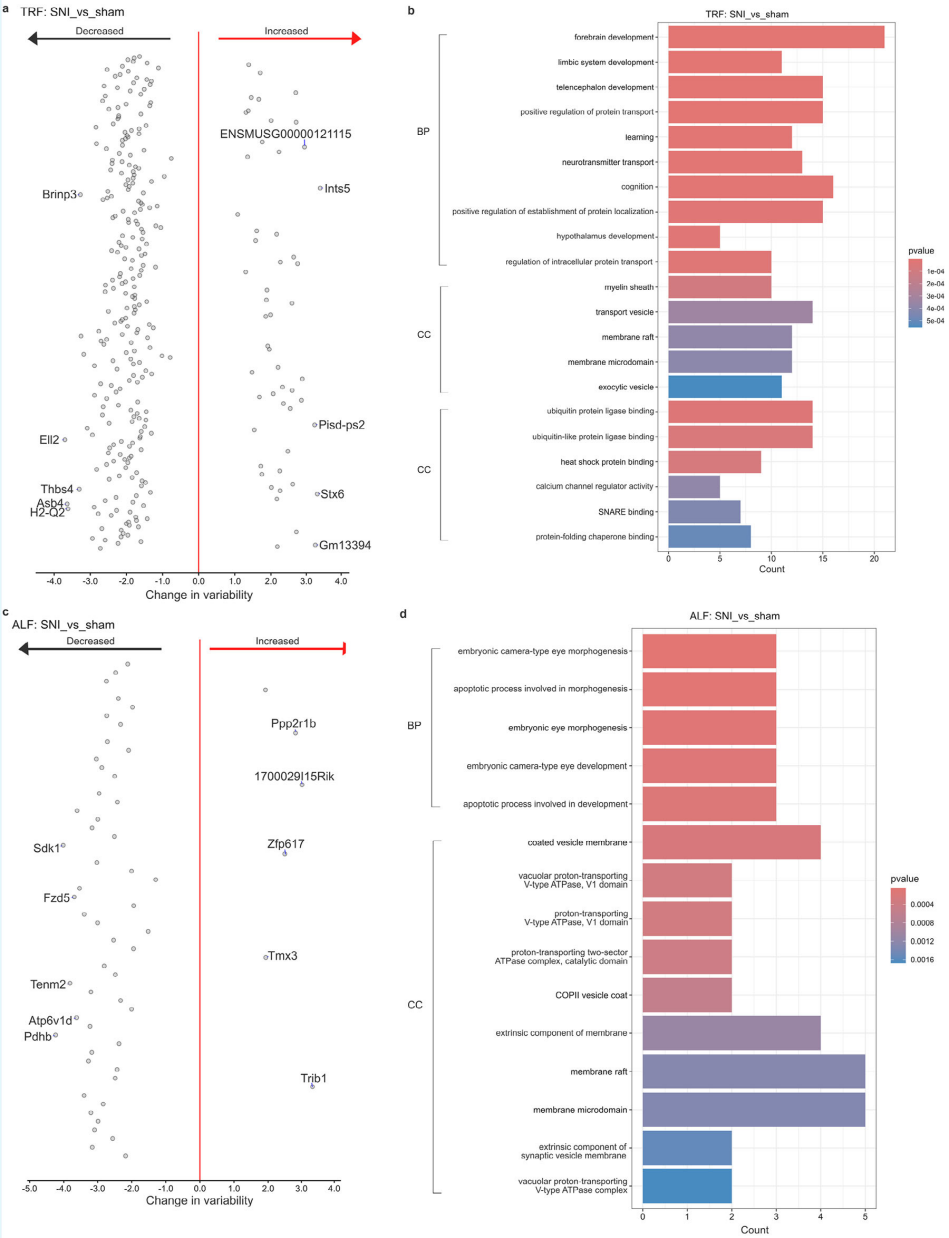
Differentially variable genes (DVGs) in the comparison between SNI and sham mice. (a, c) DVGs in the comparison between SNI versus sham mice under TRF (a) and ALF schedules (c) visualized by dispersion plot. (b, d) GO annotations of DVGs from the comparison between SNI versus sham mice under TRF (b) and ALF schedule (d). Sample size: *n* = 4/group (biological replicates). Abbreviations: TRF, time‐restricted feeding; ALF, ad libitum feeding; BP, biological process; CC, cellular component; MF, molecular function; SNI, spared nerve injury.

Since the variability analysis provides additional insight into potential gene dysregulation as part of disease progression [84], we next analyzed changes in the variance of gene expression. As a result, there were 300 DVGs between SNI and sham mice under TRF schedule, 247 with decreased and 53 with increased variability, and 58 DVGs between SNI and sham under ALF schedule, 52 with decreased and six with increased variability (Figure 6a,c and Table S4). The gene ontology analysis shows that DVGs in TRF between SNI and sham were enriched in brain development, while the DVGs in ALF are related to eye development (Figure 6b,d).

**FIGURE 7:**
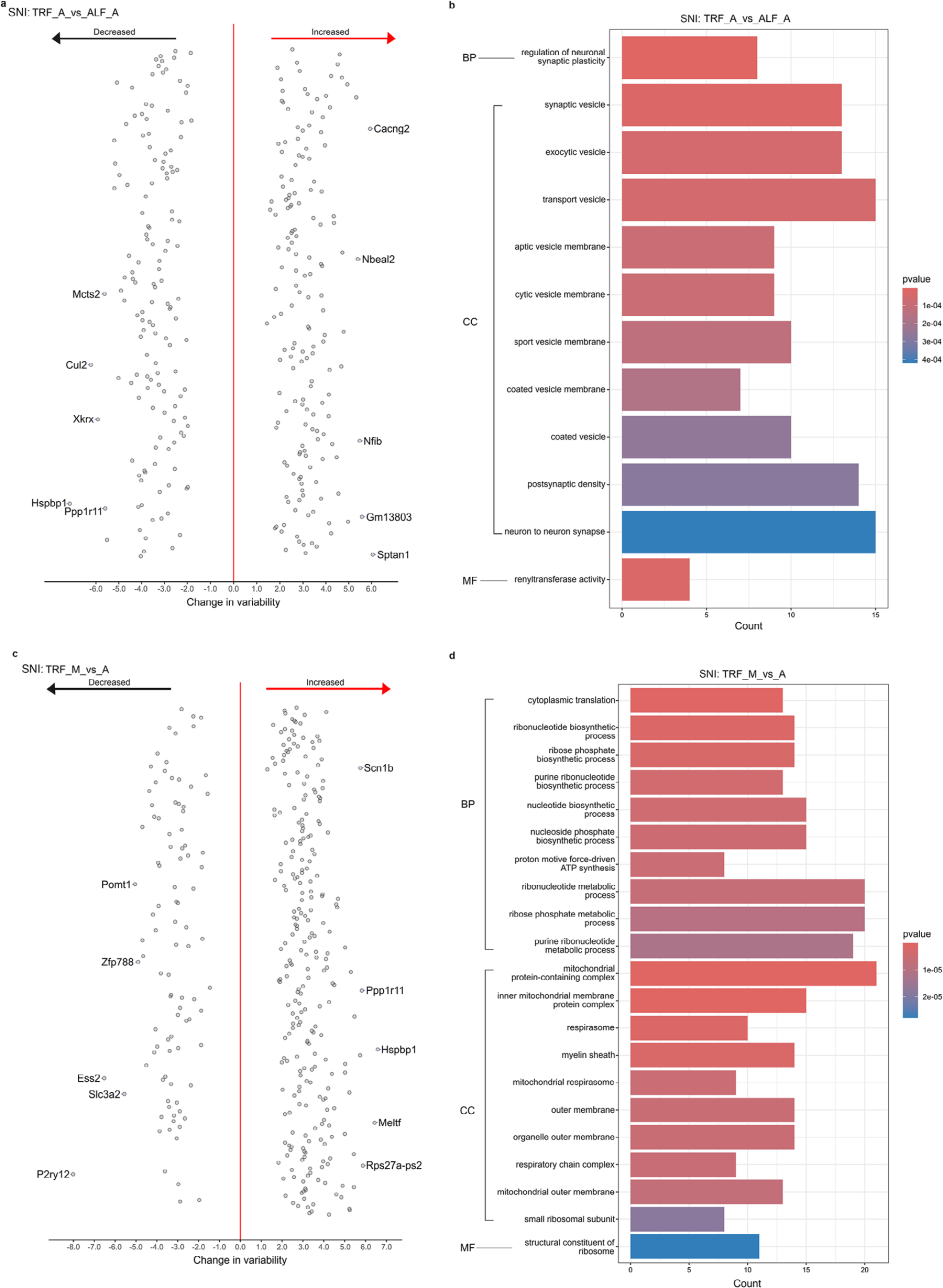
Differential variability analysis in SNI mice. (a, c) DVGs in the comparison of SNI mice from TRF_A versus ALF_A (a) and TRF_M versus TRF_A (c) visualized by dispersion plot. (b, d) GO annotations of DVGs from the comparison of TRF_A versus ALF_A (b) and TRF_M versus TRF_A (d). Sample size: *n* = 4 per group (biological replicates). Abbreviations: M, morning; A, afternoon; TRF, time‐restricted feeding; ALF, ad libitum feeding; BP, biological process; CC, cellular component; MF, molecular function; SNI, spared nerve injury.

**FIGURE 8:**
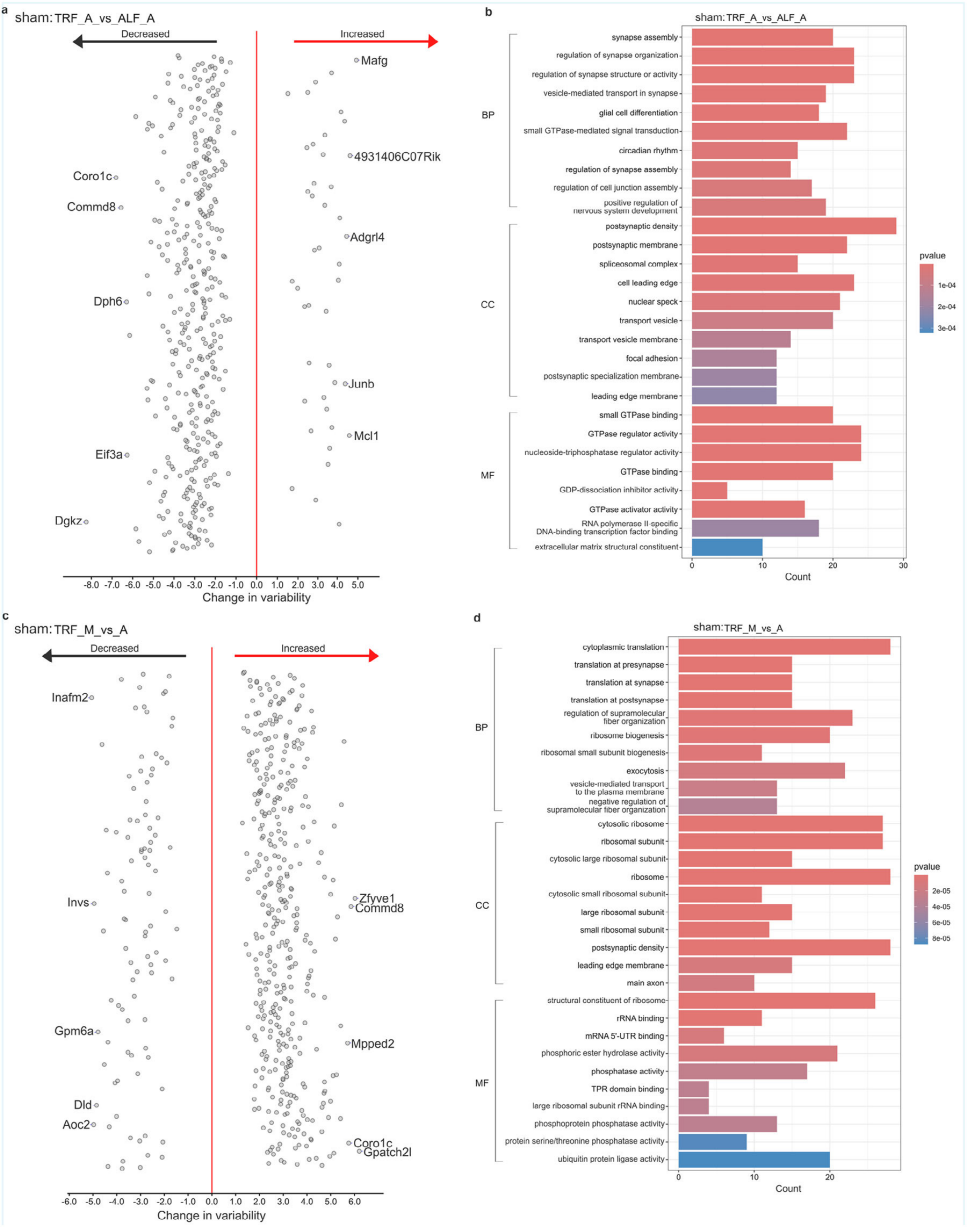
Differential variability analysis in sham mice. (a, c) DVGs in the comparison of sham mice from TRF_A versus ALF_A (a) and TRF_M versus TRF_A (c) visualized by dispersion plot. (b, d) GO annotations of DVGs from the comparison of TRF_A versus ALF_A (b) and TRF_M versus TRF_A (d). Sample size: *n* = 4 per group (biological replicates). Abbreviations: TRF, time‐restricted feeding; ALF, ad libitum feeding; BP, biological process; CC, cellular component; MF, molecular function; SNI, spared nerve injury.

A total of 308 DVGs were detected between TRF‐ and ALF‐treated SNI mice in the afternoon, out of which 142 transcripts had reduced and 166 increased variability. Among these DVGs, tachykinin 1 (*Tac1*) [85] is a known pain regulator, while nuclear factor interleukin 3 regulated (*Nfil3*) is involved in regulation of circadian rhythms [86]. Gene ontology analysis shows that these DVGs between TRF and ALF SNI mice are enriched in vesicle‐related cellular components (Figure 7a,b). In sham mice, 432 DVGs were identified between TRF and ALF schedules in the afternoon, with 389 DVGs with decreased and 43 with increased variability. The enrichment analysis shows that these DVGs are related to synapse activity and circadian rhythms (Figure 8a,b).

The comparison between morning and afternoon in SNI TRF mice revealed 354 hypothalamic transcripts with significant variability change, with 96 transcripts with decreased and 258 transcripts with increased variability. Among the DVGs with diurnal change in SNI, two DVGs, protein kinase cGMP‐dependent type I (*Prkg1*) and period 3 (*Per3*), are related to circadian regulation [87, 88]. Enrichment analysis showed that the DVGs with diurnal change in SNI were enriched for metabolic processes and mitochondrial pathways (Figure 7c,d). For the comparison between morning and afternoon in sham mice under TRF schedule, there were 488 DVGs, 97 with reduced and 391 with increased variability. Enrichment analysis reveals that these DVGs are related to translation and ribosomes (Figure 8c,d).

## 4 Discussion

Growing evidence suggests that chronic pain and associated comorbid conditions are characterized by circadian abnormalities, which might open the possibility of using chronotherapies, such as TRF, to alleviate pain and comorbid conditions such as anxiety. In the current study, we investigated the effects of TRF on NP in mice with spared nerve injury, potential changes in anxiety‐like behavior, and transcriptomic alterations in the hypothalamus, an important region for regulating anxiety and fear [89]. As our main finding, we observed that TRF alters behavior in male but not in female mice under NP conditions in the LDT and OFT, while not affecting mechanical hypersensitivity upon nerve injury.

TRF, an eating pattern that allows food access during a specific time window without calorie reduction, was previously shown to alleviate anxiety and depression scores in humans [90, 91, 92]. Similar beneficial effects of TRF on mood regulation have been reported in pre‐clinical studies. A rat model of shift work shows increased anxiety‐like behavior, which was prevented by TRF with food access only during the active phase [17]. Similarly, a 4‐week‐long protocol of intermittent fasting was able to reduce depression levels in male mice upon chronic unpredictable mild stress [93]. In our study, we observed that TRF was associated with altered behavior only in male SNI mice, which might be due to well‐known differences in anxiety regulation between the sexes [18, 94]. For example, it was previously shown that the lateral septum administration of arginine vasopressin, a known modulator of social behavior, was effective in alleviating anxiety only in male rats [95]. In line with this, male mice with a loss of arginine vasopressin‐expressing cells showed increased anxiety in the elevated plus maze (EPM) test, which was absent in female mice [96]. These studies indicate the presence of sex differences in the modulation of anxiety‐related neuronal networks. Observed behavioral differences in the LDT and OFT between male and female mice upon SNI might be also due to differences in the hormones and estrous cycle. It has been shown that female rodents experience lower anxiety levels compared to males during the pro‐estrus and estrous+pro‐estrous stages [97, 98]. In addition, changes in ovarian hormones during the estrous cycle, such as estradiol and progesterone, affect the brain regions involved in regulation of anxiety and fear, including the amygdala and the hypothalamic‐pituitary‐adrenal axis [99, 100].

While we expected that TRF might be also effective in reversing the pain hypersensitivity, the lack of the effects on mechanical and thermal thresholds might be due to the mild severity of our TRF protocol, with fasting occurring only during the light ‘resting’ phase. A TRF protocol of longer duration might have been more effective in restoring the sensory thresholds, as in a recently used 18‐week TRF protocol in a mouse model of Alzheimer's disease [101]. The daily duration of TRF might also affect behavior since the timing of feeding is a strong zeitgeber. In rodents, the feeding is adjusted according to the daily duration of fasting, which was associated with realignment of clock genes to the new schedule [102]. In humans, 8 h time‐restricted eating was more efficient for weight loss than a 12 h protocol in obese adults [103]. Therefore, longer fasting periods might have stronger effects on restoring circadian rhythms and potentially improving pain‐related behaviors.

Differential gene expression analysis of hypothalamic tissues collected from male SNI and sham mice showed that the painful nerve injury alters the transcriptomic landscape in HT under both feeding schedules, with 3005 DEGs identified in the TRF and 1074 DEGs in the ALF schedule. These DEGs were predominantly associated with regulation of circadian rhythms and ribosomal and mitochondrial processes. Among these, *Nr2f6* was downregulated in both TRF and ALF schedules between SNI and sham mice. It has been reported that *Nr2f6* mutants showed increased sensitivity to noxious heat in the hot plate test, and their adaptability was impaired under shifting LD schedule [35]. Additionally, the time‐dependent effects of TRF on the hypothalamic transcriptome are primarily evident in the afternoon, since we did not observe any DEGs between the fasting and nonfasting mice for tissues collected during the morning upon SNI, while there was a single DEG, *H1f2*, in sham controls. Out of 33 DEGs between SNI under TRF or ALF treatment in the afternoon, five DEGs, *mt‐Co3*, *Nr4a1*, *Mvp*, *Cadm4*, *Gsk3a*, have previously been linked to regulation of mood disorders. Transcription factor *Nr4a1* has increased activity under chronic stress [60], and the expression levels of *Nr4a1* in hippocampus from lupus‐prone mice impacted the anxiety scores in OFT [61]. Similarly, mutant male mice heterozygous for *Mvp* reduce anxiety‐like behaviors [62]. Mice with inhibited palmitoylation of *Cadm4*, a regulator of myelin growth and axonal organization, had increased anxiety scores in OFT compared to wild‐type mice [63]. Knockout of *Gsk3a*, encoding for serine/threonine protein kinase, increases anxiety levels specifically in female mice. Previous reports that some of these DEGs, *Nr4a1*, *Cadm4*, and *Gsk3a*, regulate anxiety in gender‐specific manner, might further explain why the TRF treatment was more efficient in reducing anxiety in male mice upon SNI in our study. Among six DEGs between the TRF and ALF in sham mice, *Sfpq* is a known suppressor of core circadian gene *Per1* [65], whereas *Agrp* plays a crucial role in the regulation of food intake as part of the NPY/AGRP pathway in the arcuate nucleus of HT [66].

Regarding the DEGs with diurnal changes in expression levels between morning and afternoon, there were 865 DEGs in sham and 95 DEGs in SNI mice under TRF, and 29 DEGs in sham and only a single DEG, *Sdf2l*, in SNI under ALF schedule. These results suggest that the SNI procedure may suppress or ‘dampen’ diurnal gene expression in the HT, while TRF appears to enhance it. Among the diurnal DEGs in SNI under TRF, seven, *Nr1d1*, *Nr1d2*, *Per1*, *Per2*, *Per3*, *Bmal1*, and *Cirbp*, are known to be core circadian genes [67, 68]. In addition to circadian regulation, some of these genes, *Nr1d1* [74], *Nr1d2* [75], *Per1*, *Per2* [76], and *Bmal1* [79], also regulate anxiety. Among the diurnal DEGs in sham mice under the TRF, three DEGs, including *Crhr1*, *Apln*, and *Htr1b*, are related to regulation of feeding. *Crhr1*‐knockout mice show increased food intake during the light phase [83]. Mice with high‐fat diet show higher *Apln* expression in HT [48], and the hypothalamic expression of *Htr1b* is altered in mice with hyperphagia [50].

In addition to its important roles in regulating the circadian system and anxiety‐like behavior, the hypothalamus is a key hub for controlling feeding and metabolism. Food intake is regulated by neurons in the arcuate nucleus of the hypothalamus (ARC), which detect hunger and satiety signals. Agouti‐related protein (AgRP) neurons in the ARC decrease energy expenditure, whereas pro‐opiomelanocortin (POMC) neurons increase it [104]. Compared to 95 DEGs with diurnal change in SNI mice undergoing TRF, there was only a single DEG, *Sdf2l1*, with altered diurnal expression in SNI mice under ALF protocol, recently linked to feeding responses in the liver [105]. Time‐restricted feeding (TRF) can mitigate alterations in the rhythmicity of plasma ghrelin levels caused by circadian disruption, a key signal for AgRP neurons [106]. Changes in the eating‐fasting pattern induced by TRF in our study may influence neuronal activity in the ARC, as well as the central clock in the hypothalamus, resulting in downstream metabolic, transcriptomic, and behavioral effects. Furthermore, TRF has been reported to elevate ketone body levels during fasting periods [107, 108], and the daily switch from glucose to ketones helps to improve metabolic circadian rhythms [109, 110]. Therefore, reinforcement of peripheral circadian rhythms via TRF may also enhance the rhythmicity of the central clock in the suprachiasmatic nucleus of the hypothalamus.

Variability of gene expression is considered an important indicator of abnormalities in gene regulation [111]. As a result of the variability analysis, we identified 308 DVGs in the hypothalamus from SNI mice between TRF and ALF during afternoon, which were largely enriched in terms related to vesicle membrane. Gene ontology (GO) analysis of DVGs showed that the majority are associated with mitochondrial‐related processes and the mitochondrial respiratory chain. This important link between changes in neuronal mitochondria and anxiety is further supported by the reports of altered mitochondrial function in anxious individuals [112].

In addition to TRF, chronobiotics, drugs which can modulate circadian rhythms, might also be considered for alleviating pain and anxiety upon painful nerve injury. The antidepressant agomelatine, a known phase‐shifting agent, can reduce anxiety and depression levels in rats subjected to chronic constant light [113]. Other nonpharmacological approaches were also recently reported to improve anxiety‐related symptoms, such as bright light therapy [114] and sleep interventions [115].

Our study has some limitations. The TRF protocol used in our study does not change total calorie intake, and future studies should also investigate the effects of restricted food consumption on these behaviors. The choice of mouse strain might affect the anxiety readouts due to known large differences in the innate anxiety levels between the strains [94, 116]. Additionally, differences in estrous cycle might have an effect on behavioral testing in female mice, and this should be closely monitored in future studies [97]. Given the effect of TRF on changes in the LDT and OFT tests in our study, which are commonly used to assess anxiety‐related behavior, it is also possible that TRF might modulate the affective component of pain. Brain regions such as the nucleus accumbens, the medial prefrontal cortex, and amygdala, which regulate the affective component of pain, are also involved in anxiety regulation [89, 117, 118]. Therefore, behavioral tests which assess emotional aspects of pain, such as the place‐conditioning paradigm and the burrowing test, should be included in future studies [119, 120].

In summary, our results show that TRF alters behavior under NP conditions in a sex‐specific manner, followed by changes in diurnal expression of hypothalamic transcripts related to circadian rhythms, anxiety regulation, and mitochondrial functions. These findings suggest that time‐restricted food access should be explored as a new therapeutic approach to alleviate comorbid anxiety in NP and emphasize the importance of considering neurobiological sex differences as part of treatment strategies for pain and comorbid conditions.

## Conflicts of Interest

The authors declare no conflicts of interest.

## Supporting information



Supplementary figures and tables are available online in the Supporting Information section.**Supporting File**: mnfr70479‐sup‐0001‐SupMat.pdf.

## Data Availability

The data that support the findings of this study are available from the corresponding author upon reasonable request.
