# Supplementary material for: Time‐Restricted Feeding Alters Behavior in a Sex‐Specific Manner in Mice With Neuropathic Pain
Source: Mol Nutr Food Res. 2026 Apr 24;70:e70479. doi: 10.1002/mnfr.70479 (PMC13109693; doi:10.1002/mnfr.70479)
Supplement: Supplementary file 1 — Supplementary figures and tables are available online in the Supporting Information section.Supporting File: mnfr70479‐sup‐0001‐SupMat.pdf. [file MNFR-70-e70479-s001.pdf]

Supplementary Fig 1.: Changes in the body weight monitored from the baseline till post-surgical day 21 in male (a) or female mice (b). Time-restricted feeding (TRF) was initiated from post-surgical day 4. ALF = ad libitum feeding, SNI = spared-nerve injury

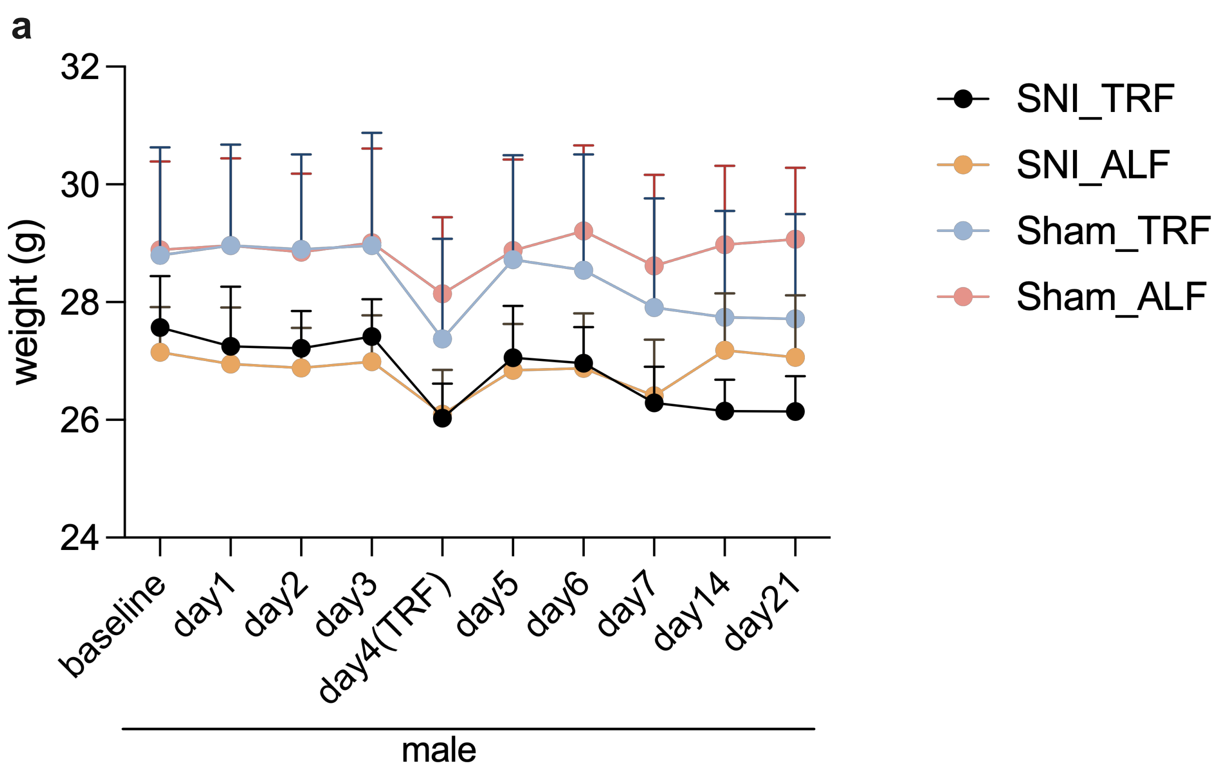

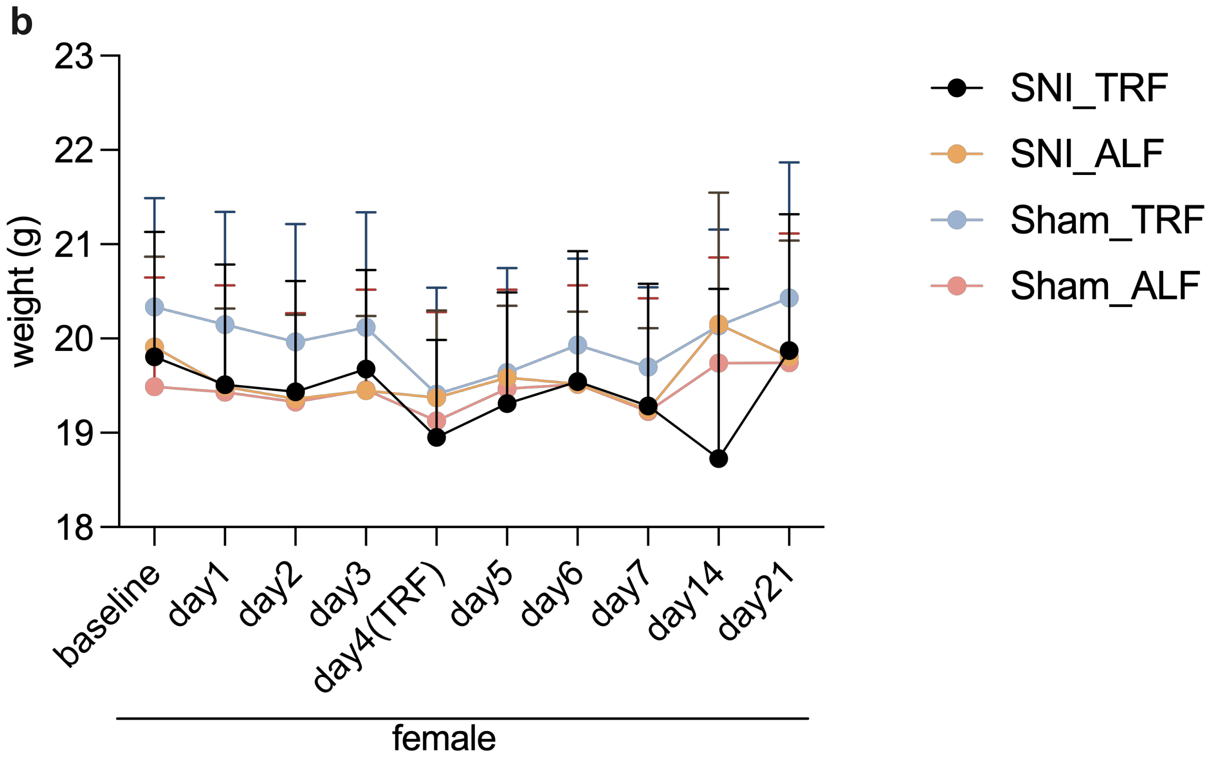

Supplementary Fig 2.: The numbers of entry in light/dark arena in light-dark box test in male (a) or female mice (c) (Two-way ANOVA). The total distance traveled in open field test in male (b) or female (d) mice (Two-way ANOVA). For behavioural tests, n = 8 per group (biological replicates). Data are presented as mean ± SD and error bars represent SD. *p < 0.05, **p < 0.01, ***p < 0.001, ****p < 0.0001.

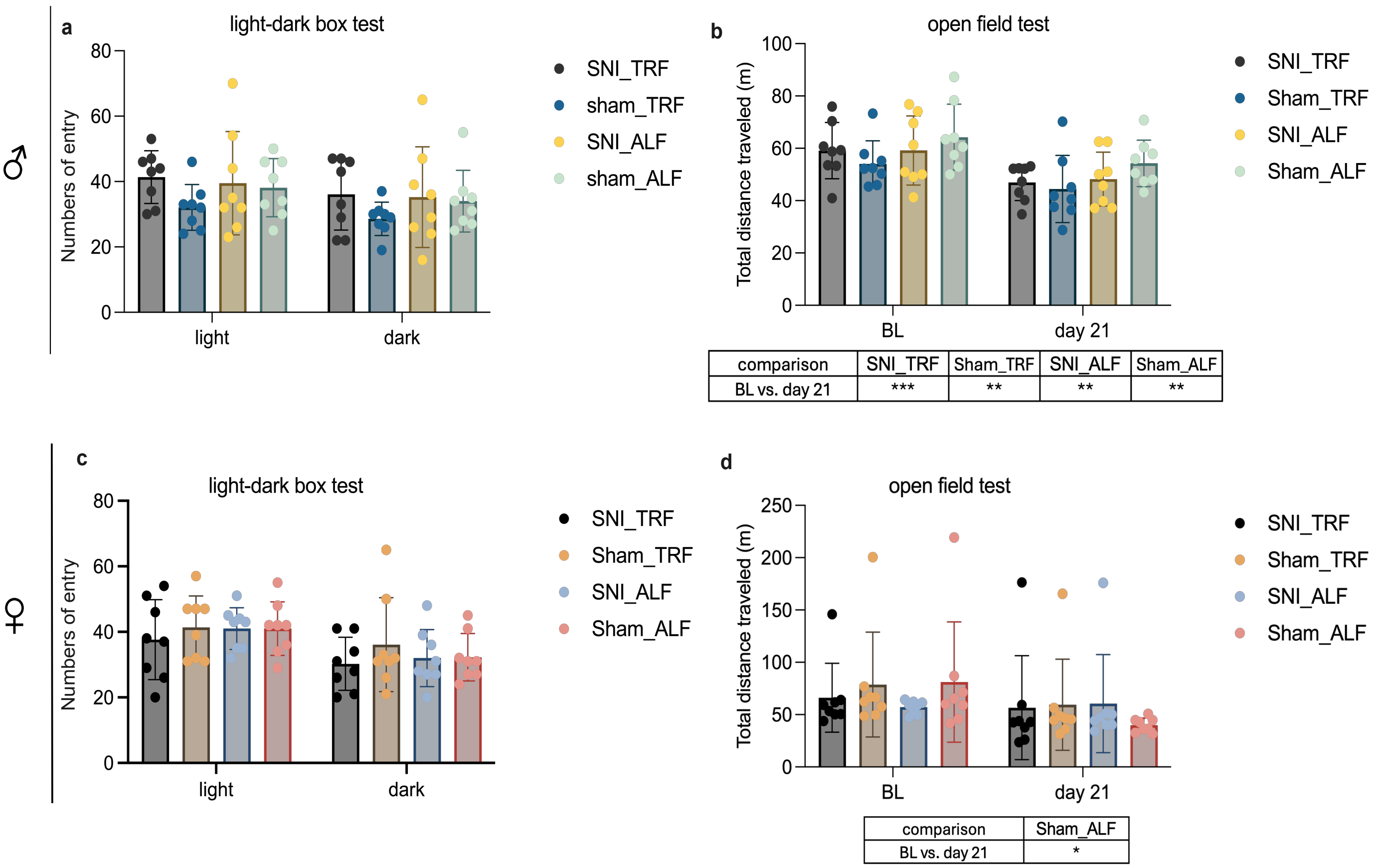

Supplementary Fig 3.: Gene ontology annotations of upregulated and downregulated differentially expressed genes (DEGs) within SNI mice. A comparison of TRF_A versus ALF_A upregulated (a) and downregulated DEGs (b), and TRF_M versus TRF_A downregulated transcripts (c). For RNA-seq, n = 4 per group (biological replicates). TRF = time-restricted feeding, ALF = ad libitum feeding, M = morning, A = afternoon. BP = biological process, CC = cellular component, MF = molecular function.

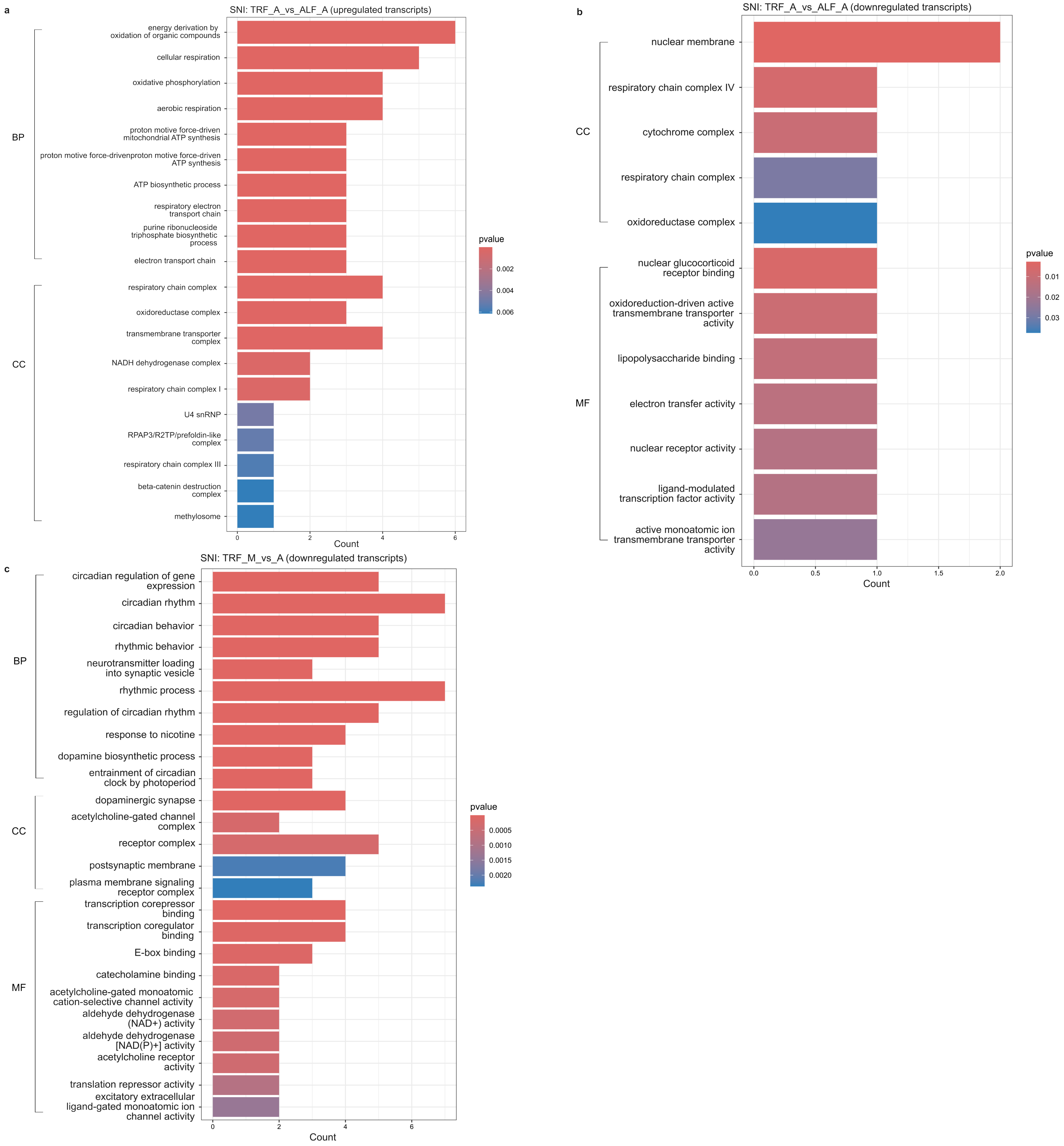

Supplementary Fig 4.: Gene ontology annotations of upregulated and downregulated differentially expressed genes (DEGs) within sham mice. A comparison of TRF_M versus TRF_A upregulated (a) and downregulated DEGs (b). For RNA-seq, n = 4 per group (biological replicates). TRF = time-restricted feeding, M = morning, A = afternoon. BP = biological process, CC = cellular component, MF = molecular function.

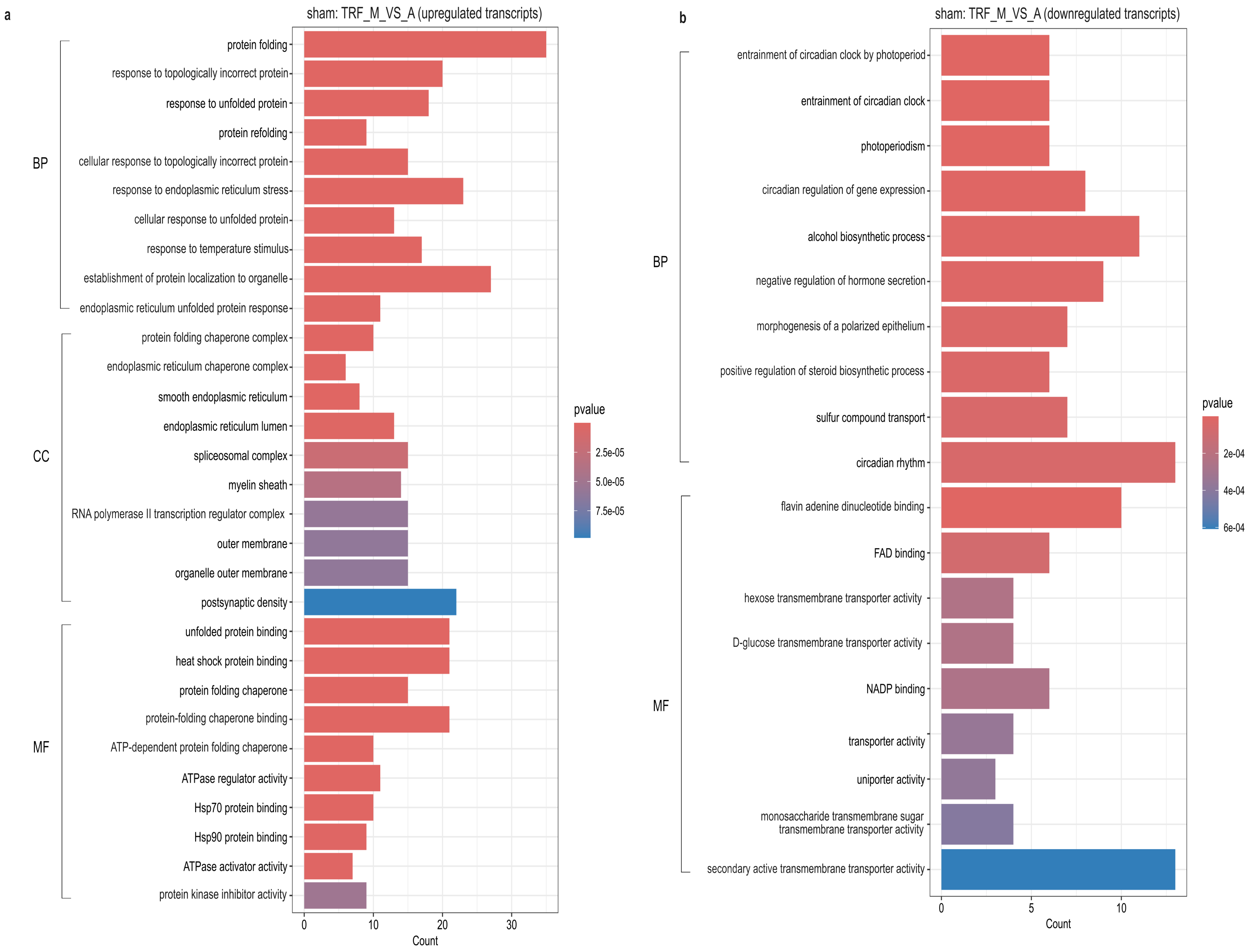

Supplementary Table 1: Total number of differentially expressed genes (DEGs) in different comparsions			
Comparison	Number of DEGs	Upregulated	Downregulated
TRF: SNI_VS_sham	3005	1434	1571
ALF: SNI_VS_sham	1074	543	531
SNI: TRF_VS_ALF	0	0	0
SNI: TRF_M_VS_A	95	47	48
SNI: ALF_M_VS_A	1	1	0
SNI: TRF_M_VS_ALF_M	0	0	0
SNI: TRF_A_VS_ALF_A	33	13	20
sham: TRF_VS_ALF	0	0	0
sham: TRF_M_VS_A	865	484	381
sham: ALF_M_VS_A	29	12	17
sham: TRF_M_VS_ALF_M	1	0	1
sham: TRF_A_VS_ALF_A	6	3	3

Supplementary Table 2: A list of DEGs in different comparisons				
Gene ID	-Log10(padj)	log2(FoldChange)	Expression	Gene
TRF: SNI_VS_sham				
ENSMUSG00000000078	2.3108	-0.185	DOWN	Klf6
ENSMUSG00000000088	18.8858	-0.5131	DOWN	Cox5a
ENSMUSG00000000149	3.5124	-0.1651	DOWN	Gna12
ENSMUSG00000000184	3.2764	-0.2541	DOWN	Ccnd2
ENSMUSG00000000399	10.379	-0.3163	DOWN	Ndufa9
ENSMUSG00000000552	3.6079	-0.1965	DOWN	Zfp385a
ENSMUSG00000000869	11.2377	-0.4828	DOWN	Il4
ENSMUSG00000000934	2.9916	-0.1845	DOWN	Top1mt
ENSMUSG00000001098	3.1632	-0.2415	DOWN	Kctd10
ENSMUSG00000001247	2.094	-0.4144	DOWN	Lsr
ENSMUSG00000001323	3.3831	-0.1729	DOWN	Srr
ENSMUSG00000001383	2.4119	-0.1705	DOWN	Zmat2
ENSMUSG00000001666	31.7878	-0.8804	DOWN	Ddt
ENSMUSG00000001741	1.3911	-0.3648	DOWN	Il16
ENSMUSG00000001768	2.3508	-0.1804	DOWN	Rin2
ENSMUSG00000001783	2.4346	-0.1388	DOWN	Rtcb
ENSMUSG00000001911	16.9398	-0.4902	DOWN	Nfix
ENSMUSG00000002006	8.8766	-0.2524	DOWN	Pdzd4
ENSMUSG00000002064	3.3579	-0.232	DOWN	Sdf2
ENSMUSG00000002274	10.0745	-0.3515	DOWN	Metrn
ENSMUSG00000002307	2.6069	-0.1892	DOWN	Daxx
ENSMUSG00000002326	3.9957	-0.3616	DOWN	Gmpr2
ENSMUSG00000002329	4.9821	-0.2723	DOWN	Mdp1
ENSMUSG00000002341	3.5374	-0.233	DOWN	Ncan
ENSMUSG00000002345	4.2811	-0.2126	DOWN	Borcs8
ENSMUSG00000002379	2.732	-0.2487	DOWN	Ndufa11
ENSMUSG00000002393	4.4746	-0.364	DOWN	Nr2f6

ENSMUSG00000002459	4.0887	-0.2452	DOWN	Rgs20
ENSMUSG00000002603	2.7345	-0.4088	DOWN	Tgfb1
ENSMUSG00000002608	2.1432	-0.1475	DOWN	Ccdc97
ENSMUSG00000002633	3.3963	-0.3093	DOWN	Shh
ENSMUSG00000002658	5.0489	-0.2418	DOWN	Gtf2f1
ENSMUSG00000002778	4.3334	-0.1727	DOWN	Kdelr1
ENSMUSG00000002820	1.7878	-0.1634	DOWN	Atg4d
ENSMUSG00000002825	2.9142	-0.2181	DOWN	Qtrt1
ENSMUSG00000002948	13.6559	-0.2489	DOWN	Map2k7
ENSMUSG00000002957	11.2976	-0.1968	DOWN	Ap2a2
ENSMUSG00000002996	2.1432	-0.2019	DOWN	Hbp1
ENSMUSG00000003031	3.3579	-0.1653	DOWN	Cdkn1b
ENSMUSG00000003038	1.5216	-0.3064	DOWN	Hmgn2
ENSMUSG00000003123	2.8429	-0.2647	DOWN	Lipe
ENSMUSG00000003131	3.443	-0.1397	DOWN	Pafah1b2
ENSMUSG00000003429	10.3682	-0.4547	DOWN	Rps11
ENSMUSG00000003444	1.3413	-0.2203	DOWN	Med29
ENSMUSG00000003518	7.4069	-0.2263	DOWN	Dusp3
ENSMUSG00000003762	2.3792	-0.1891	DOWN	Coq8b
ENSMUSG00000003948	3.0841	-0.1869	DOWN	Mmd
ENSMUSG00000004070	8.7458	-0.2761	DOWN	Hmox2
ENSMUSG00000004366	1.8857	-0.165	DOWN	Sst
ENSMUSG00000004610	4.2325	-0.5758	DOWN	Etfb
ENSMUSG00000004842	1.7275	-1.7645	DOWN	Pou1f1
ENSMUSG00000004891	3.1669	-0.3549	DOWN	Nes
ENSMUSG00000005125	6.3824	-0.2775	DOWN	Ndrg1
ENSMUSG00000005338	11.1239	-0.2121	DOWN	Cadm3
ENSMUSG00000005469	12.6276	-0.2308	DOWN	Prkaca
ENSMUSG00000005505	2.3145	-0.262	DOWN	Kbtbd4
ENSMUSG00000005506	2.4602	-0.1509	DOWN	Celf1
ENSMUSG00000005674	5.2391	-0.2461	DOWN	Tomm40l
ENSMUSG00000005677	1.4428	-1.6853	DOWN	Nr1i3
ENSMUSG00000005732	1.5797	-0.1529	DOWN	Ranbp1
ENSMUSG00000005907	1.3683	-0.1627	DOWN	Pex1
ENSMUSG00000006005	3.5905	-0.1739	DOWN	Tpr
ENSMUSG00000006299	9.4563	-0.225	DOWN	Aamp
ENSMUSG00000006418	2.144	-0.1399	DOWN	Rnf114
ENSMUSG00000006435	5.8524	-0.1711	DOWN	Neurl1a
ENSMUSG00000006586	1.3308	-0.173	DOWN	Runx1t1
ENSMUSG00000006638	3.4096	-1.135	DOWN	Abhd1
ENSMUSG00000007033	2.5815	-0.5715	DOWN	Hspa1l
ENSMUSG00000007476	21.0836	-0.5757	DOWN	Lrrc8a
ENSMUSG00000007659	6.4953	-0.1513	DOWN	Bcl2l1
ENSMUSG00000007670	5.9848	-0.2329	DOWN	Khsrp
ENSMUSG00000007682	2.8677	-0.3257	DOWN	Dio2

ENSMUSG00000007783	8.3662	-0.2202	DOWN	Cpt1c
ENSMUSG00000007815	3.7741	-0.1874	DOWN	Rhoa
ENSMUSG00000007891	8.613	-0.2065	DOWN	Ctsd
ENSMUSG00000008035	10.7821	-0.2599	DOWN	Mid1ip1
ENSMUSG00000008153	6.1369	-0.1976	DOWN	Clstn3
ENSMUSG00000008373	3.8263	-0.1587	DOWN	Prpf31
ENSMUSG00000008450	3.5496	-0.2093	DOWN	Nutf2
ENSMUSG00000008730	4.1767	-0.1482	DOWN	Hipk1
ENSMUSG00000008855	3.6933	-0.1493	DOWN	Hdac5
ENSMUSG00000009030	1.5305	-0.1437	DOWN	Pdcl
ENSMUSG00000009075	1.3802	-0.2037	DOWN	Cabp7
ENSMUSG00000009076	5.5625	-0.4239	DOWN	Zmat5
ENSMUSG00000009246	1.3474	-0.8804	DOWN	Trpm5
ENSMUSG00000009281	10.1125	-0.5708	DOWN	Rarres2
ENSMUSG00000009291	4.1226	-0.1387	DOWN	Pttg1ip
ENSMUSG00000009292	1.8194	-0.1431	DOWN	Trpm2
ENSMUSG00000009575	8.7913	-0.2007	DOWN	Cbx5
ENSMUSG00000009647	3.6079	-0.1883	DOWN	Mcu
ENSMUSG00000010067	1.919	-0.2783	DOWN	Rassf1
ENSMUSG00000010476	1.3614	-0.2141	DOWN	Ebf3
ENSMUSG00000011382	1.7372	-0.2105	DOWN	Dhdh
ENSMUSG00000011877	7.1128	-0.2059	DOWN	Git1
ENSMUSG00000013033	13.0632	-0.327	DOWN	Adgrl1
ENSMUSG00000013663	6.361	-0.215	DOWN	Pten
ENSMUSG00000013973	13.4412	-0.6317	DOWN	Dedd
ENSMUSG00000014245	3.2112	-0.2924	DOWN	Pigl
ENSMUSG00000014503	16.9398	-0.476	DOWN	Pkd2l2
ENSMUSG00000014747	2.2835	-0.5312	DOWN	Ankrd53
ENSMUSG00000014748	2.0464	-0.1382	DOWN	Tex261
ENSMUSG00000014837	2.396	-0.1731	DOWN	4931428F04Rik
ENSMUSG00000015085	3.9879	-0.2993	DOWN	Entpd2
ENSMUSG00000015126	2.414	-0.2429	DOWN	Tsr3
ENSMUSG00000015461	12.6801	-0.3284	DOWN	Atf6b
ENSMUSG00000015474	8.4501	-0.4227	DOWN	Ppt2
ENSMUSG00000015478	3.7018	-0.3141	DOWN	Rnf5
ENSMUSG00000015488	3.4874	-0.1728	DOWN	Cacfd1
ENSMUSG00000015659	1.3613	-0.1952	DOWN	Serac1
ENSMUSG00000015668	1.7437	-0.1704	DOWN	Pdzd11
ENSMUSG00000015745	2.5352	-0.227	DOWN	Plekho1
ENSMUSG00000015749	11.2867	-0.27	DOWN	Anp32e
ENSMUSG00000015750	3.0273	-0.1569	DOWN	Aph1a
ENSMUSG00000015806	4.1192	-0.306	DOWN	Qdpr
ENSMUSG00000015869	1.4482	-0.1584	DOWN	Prpsap1
ENSMUSG00000016409	1.9126	-0.171	DOWN	Nkap
ENSMUSG00000016503	9.0391	-0.4695	DOWN	Gtf3a

ENSMUSG00000016510	3.6901	-0.2483	DOWN	Mtif3
ENSMUSG00000016554	8.265	-0.2907	DOWN	Eif3d
ENSMUSG00000016831	3.5013	-0.1384	DOWN	Tox4
ENSMUSG00000016833	1.7402	-0.265	DOWN	Mrps18c
ENSMUSG00000017002	1.4568	-0.7532	DOWN	Slpi
ENSMUSG00000017009	9.7593	-0.4115	DOWN	Sdc4
ENSMUSG00000017286	2.2859	-0.1427	DOWN	Glod4
ENSMUSG00000017299	2.9343	-0.2001	DOWN	Dnttip1
ENSMUSG00000017307	3.4812	-0.2112	DOWN	Acot8
ENSMUSG00000017400	1.8782	-0.1751	DOWN	Stac2
ENSMUSG00000017404	26.3494	-0.585	DOWN	Rpl19
ENSMUSG00000017428	4.1146	-0.1675	DOWN	Psmd11
ENSMUSG00000017639	3.2724	-0.2101	DOWN	Rab11fip4
ENSMUSG00000017754	4.5871	-0.2408	DOWN	Pltp
ENSMUSG00000017764	1.6927	-0.1485	DOWN	Zswim1
ENSMUSG00000017929	7.836	-0.2465	DOWN	B4galt5
ENSMUSG00000018042	3.487	-0.1721	DOWN	Cyb5r3
ENSMUSG00000018160	2.8433	-0.1802	DOWN	Med1
ENSMUSG00000018427	1.6921	-0.152	DOWN	Ypel2
ENSMUSG00000018451	4.5544	-0.2342	DOWN	6330403K07Rik
ENSMUSG00000018547	3.1428	-0.1696	DOWN	Pip4k2b
ENSMUSG00000018559	7.0496	-0.2787	DOWN	Ctdnep1
ENSMUSG00000018666	1.775	-0.1439	DOWN	Cbx1
ENSMUSG00000018752	1.5263	-0.918	DOWN	Tnfsfm13
ENSMUSG00000018761	2.3824	-0.1647	DOWN	Mpdu1
ENSMUSG00000018921	5.4507	-0.2009	DOWN	Pelp1
ENSMUSG00000018983	1.6086	-0.4	DOWN	E2f2
ENSMUSG00000019370	4.2555	-0.2072	DOWN	Calm3
ENSMUSG00000019373	6.926	-0.3548	DOWN	Cops3
ENSMUSG00000019464	5.3641	-0.7131	DOWN	Ptger1
ENSMUSG00000019518	4.7628	-0.3169	DOWN	Ap4m1
ENSMUSG00000019578	13.3278	-0.444	DOWN	Ubxn6
ENSMUSG00000019710	2.8492	-0.2064	DOWN	Mrpl24
ENSMUSG00000019734	1.4396	-0.2088	DOWN	Tmc4
ENSMUSG00000019768	1.579	-0.2132	DOWN	Esr1
ENSMUSG00000019831	8.2698	-0.2068	DOWN	Wasf1
ENSMUSG00000019836	8.5954	-2.2086	DOWN	Amd-ps4
ENSMUSG00000019842	1.4949	-0.2233	DOWN	Traf3ip2
ENSMUSG00000019853	1.3738	-0.1985	DOWN	Hebp2
ENSMUSG00000019868	3.1249	-0.2022	DOWN	Vta1
ENSMUSG00000019883	14.2337	-0.635	DOWN	Echdc1
ENSMUSG00000020018	251.0715	-4.9199	DOWN	Snrpf
ENSMUSG00000020048	8.4927	-0.3618	DOWN	Hsp90b1
ENSMUSG00000020069	10.0183	-0.3543	DOWN	Hnrnph3
ENSMUSG00000020083	1.944	-0.15	DOWN	Fam241b

ENSMUSG00000020086	10.2186	-0.3283	DOWN	Macroh2a2
ENSMUSG00000020101	1.8353	-0.145	DOWN	Vsir
ENSMUSG00000020108	2.2588	-0.3603	DOWN	Ddit4
ENSMUSG00000020152	4.4215	-0.1481	DOWN	Actr2
ENSMUSG00000020160	2.6728	-0.3714	DOWN	Meis1
ENSMUSG00000020178	1.4142	-0.3824	DOWN	Adora2a
ENSMUSG00000020205	3.8691	-0.4299	DOWN	Phlda1
ENSMUSG00000020224	6.6315	-0.4139	DOWN	Llph
ENSMUSG00000020257	3.1603	-0.1695	DOWN	Wdr82
ENSMUSG00000020287	1.5455	-0.2557	DOWN	Mpg
ENSMUSG00000020331	1.7926	-0.1747	DOWN	Hcn2
ENSMUSG00000020333	7.1132	-0.1491	DOWN	Acsl6
ENSMUSG00000020346	4.022	-0.1546	DOWN	Mgat1
ENSMUSG00000020358	7.3154	-0.2415	DOWN	Hnrnpab
ENSMUSG00000020368	16.7128	-0.2301	DOWN	Canx
ENSMUSG00000020377	3.6128	-0.8731	DOWN	Ltc4s
ENSMUSG00000020381	1.5216	-0.3	DOWN	Mrnip
ENSMUSG00000020396	6.8089	-0.2849	DOWN	Nefh
ENSMUSG00000020422	3.6489	-0.1914	DOWN	Tns3
ENSMUSG00000020430	9.6126	-0.2663	DOWN	Pes1
ENSMUSG00000020483	33.6248	-0.4466	DOWN	Dynll2
ENSMUSG00000020492	6.3613	-0.3168	DOWN	Ska2
ENSMUSG00000020544	4.7783	-0.3725	DOWN	Cox11
ENSMUSG00000020607	2.5491	-0.1763	DOWN	Lratd1
ENSMUSG00000020612	7.9675	-0.1414	DOWN	Prkar1a
ENSMUSG00000020646	5.1206	-0.2113	DOWN	Mboat2
ENSMUSG00000020654	1.6408	-0.1491	DOWN	Adcy3
ENSMUSG00000020680	20.8597	-0.4414	DOWN	Taf15
ENSMUSG00000020684	5.2926	-0.1918	DOWN	Rasl10b
ENSMUSG00000020705	4.0971	-0.1573	DOWN	Ddx42
ENSMUSG00000020709	3.6209	-0.5056	DOWN	Adap2
ENSMUSG00000020755	5.6835	-0.1742	DOWN	Sap30bp
ENSMUSG00000020821	3.7447	-0.1792	DOWN	Kif1c
ENSMUSG00000020848	2.0167	-0.146	DOWN	Doc2b
ENSMUSG00000020849	3.6224	-0.1743	DOWN	Ywhae
ENSMUSG00000020857	5.3257	-0.8229	DOWN	Nme2
ENSMUSG00000020894	6.9304	-0.1888	DOWN	Vamp2
ENSMUSG00000020902	1.9575	-0.2049	DOWN	Ntn1
ENSMUSG00000020921	1.3342	-0.1417	DOWN	Tmem101
ENSMUSG00000020922	1.364	-0.1377	DOWN	Lsm12
ENSMUSG00000020923	2.0194	-0.1499	DOWN	Ubtf
ENSMUSG00000020940	2.1688	-0.2962	DOWN	Efcab15
ENSMUSG00000020949	6.3861	-0.2631	DOWN	Fkbp3
ENSMUSG00000020962	5.2104	-0.234	DOWN	Gtf2a1
ENSMUSG00000020992	1.6513	-0.5584	DOWN	4930512B01Rik

ENSMUSG00000021037	1.5109	-0.1889	DOWN	Ahsa1
ENSMUSG00000021039	5.0008	-0.2017	DOWN	Snw1
ENSMUSG00000021040	79.6424	-6.1349	DOWN	Slirp
ENSMUSG00000021109	4.8019	-0.1863	DOWN	Hif1a
ENSMUSG00000021113	4.6849	-0.1737	DOWN	Snapc1
ENSMUSG00000021120	2.3976	-0.2522	DOWN	Pigh
ENSMUSG00000021125	2.4252	-0.4041	DOWN	Arg2
ENSMUSG00000021139	4.0285	-1.1385	DOWN	Gm20498
ENSMUSG00000021253	2.6035	-0.2958	DOWN	Tgfb3
ENSMUSG00000021260	1.8792	-0.1593	DOWN	Hhipl1
ENSMUSG00000021262	4.5055	-0.1497	DOWN	Evl
ENSMUSG00000021268	2.838	-0.1729	DOWN	Meg3
ENSMUSG00000021377	5.3536	-0.2012	DOWN	Dek
ENSMUSG00000021379	4.2702	-0.1894	DOWN	Id4
ENSMUSG00000021395	3.0325	-0.1385	DOWN	Spin1
ENSMUSG00000021427	7.3094	-0.178	DOWN	Ssr1
ENSMUSG00000021482	1.419	-0.1713	DOWN	Prxl2c
ENSMUSG00000021514	3.8207	-0.2608	DOWN	Zfp369
ENSMUSG00000021552	2.9948	-0.2679	DOWN	Gkap1
ENSMUSG00000021578	3.514	-0.1419	DOWN	Ccdc127
ENSMUSG00000021665	4.7974	-0.1841	DOWN	Hexb
ENSMUSG00000021685	4.4714	-0.2731	DOWN	Otp
ENSMUSG00000021703	2.2088	-0.1556	DOWN	Serinc5
ENSMUSG00000021732	1.4097	-0.2947	DOWN	Fgf10
ENSMUSG00000021770	7.9675	-0.224	DOWN	Samd8
ENSMUSG00000021774	2.8429	-0.2111	DOWN	Ube2e1
ENSMUSG00000021838	2.9059	-0.1773	DOWN	Samd4
ENSMUSG00000021840	6.6613	-0.2136	DOWN	Mapk1ip1l
ENSMUSG00000021957	7.9363	-0.2019	DOWN	Tkt
ENSMUSG00000021972	4.2377	-0.187	DOWN	Hmbox1
ENSMUSG00000022048	38.5425	-0.5902	DOWN	Dpysl2
ENSMUSG00000022054	5.5085	-0.3637	DOWN	Nefm
ENSMUSG00000022055	1.3161	-0.1386	DOWN	Nefl
ENSMUSG00000022056	1.4525	-0.7898	DOWN	Adam7
ENSMUSG00000022103	2.3475	-0.1863	DOWN	Gfra2
ENSMUSG00000022114	5.1454	-0.3117	DOWN	Spry2
ENSMUSG00000022130	1.5889	-0.1796	DOWN	Tgds
ENSMUSG00000022148	1.5387	-0.3643	DOWN	Fyb
ENSMUSG00000022151	5.1923	-0.2548	DOWN	Ttc33
ENSMUSG00000022159	4.7747	-0.3607	DOWN	Rab2b
ENSMUSG00000022185	3.0986	-0.2077	DOWN	Acin1
ENSMUSG00000022193	10.3434	-0.3912	DOWN	Psmb5
ENSMUSG00000022194	52.012	-0.6776	DOWN	Pabpn1
ENSMUSG00000022219	21.6397	-2.329	DOWN	Cideb
ENSMUSG00000022223	1.7758	-0.1634	DOWN	Sdr39u1

ENSMUSG00000022228	5.2161	-0.1777	DOWN	Zscan26
ENSMUSG00000022285	15.0414	-0.2239	DOWN	Ywhaz
ENSMUSG00000022296	2.8409	-0.1611	DOWN	Baalc
ENSMUSG00000022313	2.7011	-0.3375	DOWN	Utp23
ENSMUSG00000022403	3.2058	-0.2161	DOWN	St13
ENSMUSG00000022412	5.7534	-0.2157	DOWN	Mief1
ENSMUSG00000022415	3.836	-0.1432	DOWN	Syngr1
ENSMUSG00000022416	1.4057	-0.1689	DOWN	Cacna1i
ENSMUSG00000022421	3.2683	-0.1507	DOWN	Nptxr
ENSMUSG00000022451	3.5555	-0.1659	DOWN	Twf1
ENSMUSG00000022472	1.4454	-0.16	DOWN	Desi1
ENSMUSG00000022476	1.5946	-0.1538	DOWN	Polr3h
ENSMUSG00000022500	1.5625	-0.2084	DOWN	Litaf
ENSMUSG00000022503	1.5824	-0.1833	DOWN	Nubp1
ENSMUSG00000022508	1.6263	-0.1916	DOWN	Bcl6
ENSMUSG00000022521	6.8348	-0.2297	DOWN	Crebbp
ENSMUSG00000022623	1.4121	-0.185	DOWN	Shank3
ENSMUSG00000022641	3.1249	-0.1693	DOWN	Bbx
ENSMUSG00000022718	2.9166	-0.1909	DOWN	Dgcr8
ENSMUSG00000022827	1.875	-0.1963	DOWN	Rabl3
ENSMUSG00000022842	20.3608	-0.4794	DOWN	Ece2
ENSMUSG00000022897	11.8864	-0.2586	DOWN	Dyrk1a
ENSMUSG00000022956	193.5329	-1.6631	DOWN	Atp5o
ENSMUSG00000022971	3.1781	-0.2477	DOWN	Ifnar2
ENSMUSG00000022972	7.9374	-0.5436	DOWN	Cfap298
ENSMUSG00000023004	38.5729	-0.5503	DOWN	Tuba1b
ENSMUSG00000023018	2.5567	-0.1529	DOWN	Smarcd1
ENSMUSG00000023019	5.5625	-0.3003	DOWN	Gpd1
ENSMUSG00000023026	2.4279	-0.1609	DOWN	Dip2b
ENSMUSG00000023266	2.8026	-0.1652	DOWN	Frs3
ENSMUSG00000023845	2.0737	-0.146	DOWN	Lnpep
ENSMUSG00000023861	19.3727	-0.5826	DOWN	Mpc1
ENSMUSG00000023883	9.0608	-0.2183	DOWN	Phf10
ENSMUSG00000023951	2.4551	-0.1886	DOWN	Vegfa
ENSMUSG00000023966	1.6266	-0.1423	DOWN	Rsph9
ENSMUSG00000023979	1.626	-0.5656	DOWN	Guca1b
ENSMUSG00000024018	1.8393	-0.2967	DOWN	Ccdc167
ENSMUSG00000024019	7.6538	-0.439	DOWN	Cmtr1
ENSMUSG00000024055	1.6001	-0.2079	DOWN	Cyp4f13
ENSMUSG00000024063	7.3817	-0.2635	DOWN	Lbh
ENSMUSG00000024121	18.7056	-0.3709	DOWN	Atp6v0c
ENSMUSG00000024140	5.3979	-0.2228	DOWN	Epas1
ENSMUSG00000024160	14.6212	-0.6491	DOWN	Spsb3
ENSMUSG00000024209	3.3185	-0.563	DOWN	Acsbg3
ENSMUSG00000024213	4.2563	-0.1852	DOWN	Nudt3

ENSMUSG00000024217	2.3145	-0.2003	DOWN	Snrpc
ENSMUSG00000024218	2.4884	-0.1842	DOWN	Taf11
ENSMUSG00000024260	3.0865	-0.145	DOWN	Sap130
ENSMUSG00000024261	6.2795	-0.2364	DOWN	Syt4
ENSMUSG00000024294	7.1225	-0.1892	DOWN	Mib1
ENSMUSG00000024309	1.7214	-0.1754	DOWN	Pfdn6
ENSMUSG00000024350	5.1402	-0.1908	DOWN	Dnajc18
ENSMUSG00000024423	5.3211	-0.221	DOWN	Impact
ENSMUSG00000024426	1.3716	-0.1491	DOWN	Atat1
ENSMUSG00000024429	3.2383	-0.1573	DOWN	Gnl1
ENSMUSG00000024436	12.3429	-0.5204	DOWN	Mrps18b
ENSMUSG00000024457	3.2724	-0.1872	DOWN	Trim26
ENSMUSG00000024491	3.1001	-0.1643	DOWN	Rbm27
ENSMUSG00000024570	8.3068	-0.3955	DOWN	Rbfa
ENSMUSG00000024590	4.1663	-0.2207	DOWN	Lmnb1
ENSMUSG00000024660	1.471	-0.1792	DOWN	Incenp
ENSMUSG00000024732	1.5079	-0.2106	DOWN	Ccdc86
ENSMUSG00000024736	1.7206	-0.1577	DOWN	Tmem132a
ENSMUSG00000024742	10.9279	-1.0701	DOWN	Fen1
ENSMUSG00000024743	1.7534	-0.1378	DOWN	Syt7
ENSMUSG00000024792	1.9847	-0.1677	DOWN	Zfpl1
ENSMUSG00000024807	3.0915	-0.1463	DOWN	Syvn1
ENSMUSG00000024854	7.7377	-0.5796	DOWN	Pold4
ENSMUSG00000024896	2.074	-0.1446	DOWN	Minpp1
ENSMUSG00000024897	3.3041	-0.2104	DOWN	Apba1
ENSMUSG00000024921	4.724	-0.197	DOWN	Smarca2
ENSMUSG00000024926	2.1233	-0.1406	DOWN	Kat5
ENSMUSG00000024935	4.5314	-0.1986	DOWN	Slc1a1
ENSMUSG00000024955	7.6374	-0.2379	DOWN	Esrra
ENSMUSG00000024958	2.4491	-0.1429	DOWN	Gpr137
ENSMUSG00000024959	22.1278	-0.761	DOWN	Bad
ENSMUSG00000024970	2.7255	-0.1584	DOWN	Spindoc
ENSMUSG00000024985	7.5908	-0.3617	DOWN	Tcf7l2
ENSMUSG00000024991	5.7607	-0.2136	DOWN	Eif3a
ENSMUSG00000025010	2.0509	-0.1932	DOWN	Ccnj
ENSMUSG00000025142	2.6915	-0.2185	DOWN	Aspscr1
ENSMUSG00000025192	1.4835	-0.2106	DOWN	Entpd7
ENSMUSG00000025204	33.9867	-0.6465	DOWN	Ndufb8
ENSMUSG00000025209	2.1571	-0.2093	DOWN	Twnk
ENSMUSG00000025226	1.5944	-0.2415	DOWN	Fbxl15
ENSMUSG00000025245	2.13	-0.141	DOWN	Lztfl1
ENSMUSG00000025265	1.3597	-0.1833	DOWN	Fgd1
ENSMUSG00000025266	4.1877	-0.156	DOWN	Gnl3l
ENSMUSG00000025314	2.6417	-0.1722	DOWN	Ptprj
ENSMUSG00000025318	5.5261	-0.1676	DOWN	Jph3

ENSMUSG00000025352	6.0998	-0.3856	DOWN	Gdf11
ENSMUSG00000025371	2.8603	-0.2805	DOWN	Chmp6
ENSMUSG00000025374	2.6802	-0.1615	DOWN	Nabp2
ENSMUSG00000025408	2.3516	-0.2443	DOWN	Ddit3
ENSMUSG00000025422	2.115	-0.1924	DOWN	Agap2
ENSMUSG00000025427	1.8979	-0.1689	DOWN	Rnf165
ENSMUSG00000025429	1.8004	-0.2126	DOWN	Pstpip2
ENSMUSG00000025432	1.5026	-0.3504	DOWN	Avil
ENSMUSG00000025486	7.4616	-0.2916	DOWN	Sirt3
ENSMUSG00000025505	3.2684	-0.1742	DOWN	Tmem80
ENSMUSG00000025578	2.7982	-0.2549	DOWN	Cbx8
ENSMUSG00000025646	1.8058	-0.1503	DOWN	Atrip
ENSMUSG00000025735	9.3671	-0.7335	DOWN	Rhbdl1
ENSMUSG00000025739	21.9619	-1.2451	DOWN	Gng13
ENSMUSG00000025743	2.462	-0.1424	DOWN	Sdc3
ENSMUSG00000025795	2.1453	-0.2013	DOWN	Rassf3
ENSMUSG00000025813	2.4521	-0.1839	DOWN	Homer2
ENSMUSG00000025880	6.1543	-0.3149	DOWN	Smad7
ENSMUSG00000026078	2.273	-0.1808	DOWN	Pdcl3
ENSMUSG00000026104	1.5349	-0.2153	DOWN	Stat1
ENSMUSG00000026155	2.669	-0.1678	DOWN	Smap1
ENSMUSG00000026156	3.017	-0.2735	DOWN	B3gat2
ENSMUSG00000026204	4.3359	-0.1466	DOWN	Ptprn
ENSMUSG00000026220	1.5603	-0.3364	DOWN	Slc16a14
ENSMUSG00000026234	7.3014	-0.1779	DOWN	Ncl
ENSMUSG00000026238	43.9622	-0.5375	DOWN	Ptma
ENSMUSG00000026276	3.9067	-0.5938	DOWN	Septin2
ENSMUSG00000026344	4.0715	-0.2517	DOWN	Lypd1
ENSMUSG00000026421	2.8683	-0.1445	DOWN	Csrp1
ENSMUSG00000026434	25.1487	-0.3742	DOWN	Nucks1
ENSMUSG00000026466	2.4448	-0.1446	DOWN	Tor1aip1
ENSMUSG00000026470	2.0854	-0.2554	DOWN	Stx6
ENSMUSG00000026473	12.3209	-0.2285	DOWN	Glul
ENSMUSG00000026499	3.7628	-0.1651	DOWN	Acbd3
ENSMUSG00000026500	4.9158	-0.9789	DOWN	Cox20
ENSMUSG00000026565	3.2534	-0.2052	DOWN	Pou2f1
ENSMUSG00000026600	3.3327	-0.1752	DOWN	Soat1
ENSMUSG00000026623	3.9771	-0.2073	DOWN	Lpgat1
ENSMUSG00000026632	1.5216	-0.17	DOWN	Tatdn3
ENSMUSG00000026659	1.6797	-0.1763	DOWN	Dusp12
ENSMUSG00000026672	2.3517	-0.1539	DOWN	Optn
ENSMUSG00000026678	1.3668	-0.1835	DOWN	Rgs5
ENSMUSG00000026715	1.9491	-1.0951	DOWN	Serpinc1
ENSMUSG00000026799	4.8554	-0.2736	DOWN	Med27
ENSMUSG00000026810	4.1814	-0.1695	DOWN	Dpm2

ENSMUSG00000026858	2.8173	-0.1379	DOWN	Miga2
ENSMUSG00000026927	2.5129	-0.1498	DOWN	Entr1
ENSMUSG00000026959	2.7147	-0.1548	DOWN	Grin1
ENSMUSG00000027001	4.6303	-0.3511	DOWN	Dusp19
ENSMUSG00000027080	2.1491	-0.1384	DOWN	Med19
ENSMUSG00000027122	4.8284	-0.1936	DOWN	Arl14ep
ENSMUSG00000027223	17.2098	-0.2466	DOWN	Mapk8ip1
ENSMUSG00000027245	11.7199	-0.9335	DOWN	Hypk
ENSMUSG00000027246	14.5208	-1.4649	DOWN	Ell3
ENSMUSG00000027255	1.9481	-0.1498	DOWN	Arfgap2
ENSMUSG00000027349	3.189	-0.2154	DOWN	Fam98b
ENSMUSG00000027350	7.1611	-0.2325	DOWN	Chgb
ENSMUSG00000027351	1.4121	-0.1515	DOWN	Spred1
ENSMUSG00000027374	2.5824	-0.1421	DOWN	Mrps5
ENSMUSG00000027376	1.6662	-0.7726	DOWN	Prom2
ENSMUSG00000027419	3.6351	-0.1588	DOWN	Pcsk2
ENSMUSG00000027425	1.8222	-0.1455	DOWN	Kat14
ENSMUSG00000027434	1.7591	-0.2178	DOWN	Nkx2-2
ENSMUSG00000027489	1.7429	-0.1788	DOWN	Necab3
ENSMUSG00000027520	2.4462	-0.1404	DOWN	Zdbf2
ENSMUSG00000027523	26.0527	-0.4567	DOWN	Gnas
ENSMUSG00000027570	2.6233	-0.196	DOWN	Col9a3
ENSMUSG00000027610	1.709	-0.1432	DOWN	Gss
ENSMUSG00000027618	3.1781	-0.1604	DOWN	Nfs1
ENSMUSG00000027630	3.1603	-0.1791	DOWN	Tbl1xr1
ENSMUSG00000027637	6.1306	-0.3435	DOWN	Rab5if
ENSMUSG00000027663	3.2926	-0.1942	DOWN	Zmat3
ENSMUSG00000027674	1.6588	-0.1966	DOWN	Pex5l
ENSMUSG00000027680	2.0407	-0.1727	DOWN	Fxr1
ENSMUSG00000027710	6.9954	-0.2282	DOWN	Acad9
ENSMUSG00000027712	1.5882	-0.1543	DOWN	Anxa5
ENSMUSG00000027714	2.9323	-0.1768	DOWN	Exosc9
ENSMUSG00000027765	1.5266	-0.1993	DOWN	P2ry1
ENSMUSG00000027777	17.6093	-1.0151	DOWN	Schip1
ENSMUSG00000027881	2.1707	-0.1551	DOWN	Prpf38b
ENSMUSG00000027942	1.6979	-0.191	DOWN	4933434E20Rik
ENSMUSG00000027957	3.1651	-0.1601	DOWN	Slc35a3
ENSMUSG00000027983	6.7572	-0.3381	DOWN	Cyp2u1
ENSMUSG00000028042	2.7169	-0.2078	DOWN	Zbtb7b
ENSMUSG00000028049	4.4741	-0.1883	DOWN	Scamp3
ENSMUSG00000028069	2.4501	-0.2001	DOWN	Gpatch4
ENSMUSG00000028104	13.1583	-0.4256	DOWN	Polr3gl
ENSMUSG00000028150	2.2236	-0.2824	DOWN	Rorc
ENSMUSG00000028152	3.0065	-0.1742	DOWN	Tspan5
ENSMUSG00000028333	21.2957	-0.4171	DOWN	Anp32b

ENSMUSG00000028337	1.4215	-0.1455	DOWN	Coro2a
ENSMUSG00000028426	20.4765	-0.2176	DOWN	Rad23b
ENSMUSG00000028439	21.7222	-0.3856	DOWN	Fam219a
ENSMUSG00000028458	6.8954	-0.2608	DOWN	Tesk1
ENSMUSG00000028459	2.4497	-0.881	DOWN	Cd72
ENSMUSG00000028466	1.9514	-0.1558	DOWN	Creb3
ENSMUSG00000028538	3.089	-0.1577	DOWN	St3gal3
ENSMUSG00000028565	3.7109	-0.1644	DOWN	Nfia
ENSMUSG00000028618	6.3297	-0.2013	DOWN	Tmem59
ENSMUSG00000028642	3.0328	-0.7055	DOWN	Tmem269
ENSMUSG00000028645	1.5499	-0.1444	DOWN	Slc2a1
ENSMUSG00000028664	1.8506	-0.1837	DOWN	Ephb2
ENSMUSG00000028668	1.6484	-0.1485	DOWN	Eloa
ENSMUSG00000028688	1.4508	-0.2179	DOWN	Toe1
ENSMUSG00000028701	10.9019	-0.3397	DOWN	Lurap1
ENSMUSG00000028703	3.4564	-0.1962	DOWN	Lrrc41
ENSMUSG00000028747	1.3843	-0.2861	DOWN	Htr6
ENSMUSG00000028795	10.3114	-0.3214	DOWN	Ccdc28b
ENSMUSG00000028841	1.3582	-1.0924	DOWN	Cnksr1
ENSMUSG00000028851	4.3048	-0.2686	DOWN	Nudc
ENSMUSG00000028882	18.3356	-0.3867	DOWN	Ppp1r8
ENSMUSG00000028931	5.4614	-0.2067	DOWN	Kcnab2
ENSMUSG00000028943	2.9301	-0.3012	DOWN	Espn
ENSMUSG00000028975	17.1436	-0.4535	DOWN	Pex14
ENSMUSG00000028978	2.0638	-0.2137	DOWN	Nos3
ENSMUSG00000028982	3.6236	-0.2416	DOWN	Slc25a33
ENSMUSG00000028999	1.4407	-0.1695	DOWN	Rint1
ENSMUSG00000029028	5.0279	-0.3048	DOWN	Lrrc47
ENSMUSG00000029131	2.6529	-0.1537	DOWN	Dnajb6
ENSMUSG00000029141	1.6662	-0.1732	DOWN	Slc4a1ap
ENSMUSG00000029227	4.2549	-0.157	DOWN	Fip1l1
ENSMUSG00000029276	6.2086	-0.4178	DOWN	Glmn
ENSMUSG00000029279	1.5216	-0.1696	DOWN	Brdt
ENSMUSG00000029394	8.7537	-0.2952	DOWN	Cdk2ap1
ENSMUSG00000029402	4.2005	-0.312	DOWN	Snrnp35
ENSMUSG00000029408	2.3947	-0.1386	DOWN	Abcb9
ENSMUSG00000029426	15.4736	-0.2641	DOWN	Scarb2
ENSMUSG00000029463	4.3505	-0.2167	DOWN	Fam216a
ENSMUSG00000029475	2.2648	-0.1745	DOWN	Kdm2b
ENSMUSG00000029545	2.7804	-0.2523	DOWN	Acads
ENSMUSG00000029550	3.1651	-0.1417	DOWN	Sppl3
ENSMUSG00000029570	2.0474	-0.2441	DOWN	Lfng
ENSMUSG00000029610	2.5056	-0.2178	DOWN	Aimp2
ENSMUSG00000029627	1.3715	-0.1837	DOWN	Zkscan14
ENSMUSG00000029636	2.1935	-0.163	DOWN	Wasf3

ENSMUSG00000029672	4.3953	-0.2573	DOWN	Fam3c
ENSMUSG00000029708	2.0952	-0.1875	DOWN	Gcc1
ENSMUSG00000029713	4.4227	-0.1893	DOWN	Gnb2
ENSMUSG00000029720	10.947	-1.5109	DOWN	Gm20605
ENSMUSG00000029726	5.0564	-0.1604	DOWN	Mepce
ENSMUSG00000029729	2.908	-0.159	DOWN	Zkscan1
ENSMUSG00000029823	2.9731	-0.1492	DOWN	Luc7l2
ENSMUSG00000029836	51.3145	-0.4792	DOWN	Cbx3
ENSMUSG00000029868	2.2289	-0.4468	DOWN	Trpv6
ENSMUSG00000029878	1.6101	-0.1655	DOWN	Dbpht2
ENSMUSG00000030032	1.354	-0.1505	DOWN	Wdr54
ENSMUSG00000030056	1.9817	-0.1789	DOWN	Isy1
ENSMUSG00000030134	8.9359	-0.179	DOWN	Rasgef1a
ENSMUSG00000030172	1.8758	-0.2223	DOWN	Erc1
ENSMUSG00000030189	1.4228	-0.1586	DOWN	Ybx3
ENSMUSG00000030220	3.4337	-0.4763	DOWN	Arhgdib
ENSMUSG00000030264	2.0792	-0.2012	DOWN	Thumpd3
ENSMUSG00000030283	1.4749	-0.1701	DOWN	St8sia1
ENSMUSG00000030329	9.9985	-0.2279	DOWN	Pianp
ENSMUSG00000030342	1.6662	-0.1587	DOWN	Cd9
ENSMUSG00000030400	1.59	-0.1822	DOWN	Ercc2
ENSMUSG00000030411	8.6846	-0.3486	DOWN	Nova2
ENSMUSG00000030428	7.2041	-0.1659	DOWN	Ttyh1
ENSMUSG00000030551	3.7924	-0.2286	DOWN	Nr2f2
ENSMUSG00000030584	1.3316	-0.1738	DOWN	Dpf1
ENSMUSG00000030604	1.6424	-0.2108	DOWN	Zfp626
ENSMUSG00000030611	1.3623	-0.1697	DOWN	Mrps11
ENSMUSG00000030613	1.3407	-0.184	DOWN	Ccdc90b
ENSMUSG00000030649	4.0566	-0.3147	DOWN	Anapc15
ENSMUSG00000030703	1.5771	-0.3634	DOWN	Gdpd3
ENSMUSG00000030737	1.7217	-0.2032	DOWN	Slco2b1
ENSMUSG00000030739	1.9897	-0.172	DOWN	Myh14
ENSMUSG00000030780	4.6834	-0.2419	DOWN	Rusf1
ENSMUSG00000030786	3.7018	-0.3942	DOWN	Itgam
ENSMUSG00000030869	10.2107	-0.3441	DOWN	Ndufab1
ENSMUSG00000030870	5.2796	-0.2446	DOWN	Ubfd1
ENSMUSG00000030890	32.0523	-4.1937	DOWN	Ilk
ENSMUSG00000030917	1.4407	-0.1786	DOWN	Tmem159
ENSMUSG00000030960	1.3225	-0.1666	DOWN	Eef1akmt2
ENSMUSG00000030983	6.7582	-0.2502	DOWN	Bccip
ENSMUSG00000031012	4.4301	-0.1629	DOWN	Cask
ENSMUSG00000031029	1.85	-0.1473	DOWN	Eif3f
ENSMUSG00000031077	2.3792	-0.3476	DOWN	Fadd
ENSMUSG00000031143	1.6532	-2.373	DOWN	Ccdc22
ENSMUSG00000031144	7.8202	-0.2179	DOWN	Syp

ENSMUSG00000031149	6.4187	-0.3438	DOWN	Praf2
ENSMUSG00000031153	6.927	-0.1631	DOWN	Gripap1
ENSMUSG00000031202	3.2534	-0.1391	DOWN	Rab39b
ENSMUSG00000031245	2.8048	-0.3287	DOWN	Hmgn5
ENSMUSG00000031299	5.1259	-0.1809	DOWN	Pdha1
ENSMUSG00000031302	16.8928	-0.3275	DOWN	Nlgn3
ENSMUSG00000031327	5.7712	-0.1584	DOWN	Chic1
ENSMUSG00000031340	2.3527	-0.234	DOWN	Gabre
ENSMUSG00000031431	2.8411	-0.2979	DOWN	Tsc22d3
ENSMUSG00000031445	3.2655	-0.2222	DOWN	Proz
ENSMUSG00000031447	9.856	-0.2115	DOWN	Lamp1
ENSMUSG00000031568	3.9269	-0.1757	DOWN	Rwdd4a
ENSMUSG00000031577	2.8739	-0.1937	DOWN	Tti2
ENSMUSG00000031672	3.7483	-0.1629	DOWN	Got2
ENSMUSG00000031691	5.0977	-0.1996	DOWN	Tnpo2
ENSMUSG00000031714	2.3539	-0.1879	DOWN	Gab1
ENSMUSG00000031732	3.3204	-0.1773	DOWN	Phlpp2
ENSMUSG00000031734	2.6746	-0.3385	DOWN	Irx3
ENSMUSG00000031775	9.0378	-0.6745	DOWN	Pllp
ENSMUSG00000031990	3.5857	-0.2825	DOWN	Jam3
ENSMUSG00000032012	11.4092	-0.3872	DOWN	Nectin1
ENSMUSG00000032024	2.7218	-0.1859	DOWN	Clmp
ENSMUSG00000032034	1.6085	-0.1849	DOWN	Kcnj5
ENSMUSG00000032040	1.7247	-0.1959	DOWN	Dcps
ENSMUSG00000032050	3.4252	-0.1577	DOWN	Rdx
ENSMUSG00000032058	1.4947	-0.1473	DOWN	Ppp2r1b
ENSMUSG00000032085	1.4032	-1.1174	DOWN	Tagln
ENSMUSG00000032097	16.5985	-0.3328	DOWN	Ddx6
ENSMUSG00000032182	1.3284	-0.1721	DOWN	Yipf2
ENSMUSG00000032215	9.8509	-0.3369	DOWN	Rsl24d1
ENSMUSG00000032245	3.574	-0.2724	DOWN	Cln6
ENSMUSG00000032285	1.5127	-0.2385	DOWN	Dnaja4
ENSMUSG00000032305	2.4673	-0.1807	DOWN	Fam219b
ENSMUSG00000032307	2.4679	-0.1383	DOWN	Ube2q2
ENSMUSG00000032309	4.914	-0.1901	DOWN	Fbxo22
ENSMUSG00000032312	3.0028	-0.168	DOWN	Csk
ENSMUSG00000032327	1.892	-0.7534	DOWN	Stra6
ENSMUSG00000032383	6.9887	-0.1904	DOWN	Ppib
ENSMUSG00000032478	2.3599	-0.2443	DOWN	Nme6
ENSMUSG00000032537	3.5271	-0.1583	DOWN	Ephb1
ENSMUSG00000032578	1.9638	-0.3203	DOWN	Cish
ENSMUSG00000032640	1.3661	-0.1894	DOWN	Chsy1
ENSMUSG00000032666	3.0572	-0.1465	DOWN	1700025G04Rik
ENSMUSG00000032803	8.326	-0.1975	DOWN	Cdv3
ENSMUSG00000032867	17.2602	-0.3788	DOWN	Fbxw8

ENSMUSG00000032952	3.0724	-0.7359	DOWN	Ap4b1
ENSMUSG00000033029	1.3935	-0.3003	DOWN	1700088E04Rik
ENSMUSG00000033039	2.2024	-0.2337	DOWN	Micall1
ENSMUSG00000033061	13.7037	-0.3194	DOWN	Resp18
ENSMUSG00000033088	1.3635	-0.2067	DOWN	Triobp
ENSMUSG00000033126	2.5563	-0.3221	DOWN	Ybey
ENSMUSG00000033152	50.0266	-0.4785	DOWN	Podxl2
ENSMUSG00000033530	3.5124	-0.1432	DOWN	Ttc7b
ENSMUSG00000033565	4.5107	-0.2045	DOWN	Rbfox2
ENSMUSG00000033707	1.3319	-0.178	DOWN	Lrrc24
ENSMUSG00000033720	8.7915	-0.2234	DOWN	Sfxn5
ENSMUSG00000033751	93.1039	-1.2265	DOWN	Gadd45gip1
ENSMUSG00000033763	6.1357	-0.203	DOWN	Mtss2
ENSMUSG00000033768	2.1168	-0.1749	DOWN	Nrxn2
ENSMUSG00000033813	2.84	-0.1424	DOWN	Tcea1
ENSMUSG00000033862	5.4281	-0.2023	DOWN	Cdk10
ENSMUSG00000033931	2.2604	-0.16	DOWN	Rbm34
ENSMUSG00000033948	1.8601	-0.1686	DOWN	Zswim5
ENSMUSG00000033955	1.5461	-0.1586	DOWN	Tnks1bp1
ENSMUSG00000034063	1.6424	-0.3514	DOWN	4930590J08Rik
ENSMUSG00000034101	4.0012	-0.3351	DOWN	Ctnnd1
ENSMUSG00000034120	8.1669	-0.2789	DOWN	Srsf2
ENSMUSG00000034158	12.8617	-0.245	DOWN	Lrrc58
ENSMUSG00000034210	2.2987	-0.1669	DOWN	Efcab14
ENSMUSG00000034245	10.0257	-0.216	DOWN	Hdac11
ENSMUSG00000034254	2.0371	-0.1439	DOWN	Agpat1
ENSMUSG00000034271	2.786	-0.2089	DOWN	Jdp2
ENSMUSG00000034292	3.1654	-0.1911	DOWN	Traf3ip1
ENSMUSG00000034300	3.0957	-0.1699	DOWN	Fam53c
ENSMUSG00000034312	1.9317	-0.1455	DOWN	Iqsec1
ENSMUSG00000034377	6.485	-0.2075	DOWN	Tulp4
ENSMUSG00000034595	1.5998	-0.2099	DOWN	Ppp1r18
ENSMUSG00000034613	3.8733	-0.2165	DOWN	Ppm1h
ENSMUSG00000034681	7.1958	-0.1762	DOWN	Rnps1
ENSMUSG00000034723	3.2218	-0.1411	DOWN	Tmx4
ENSMUSG00000034731	1.8758	-0.1431	DOWN	Dgkh
ENSMUSG00000034768	1.5932	-0.2189	DOWN	Asb16
ENSMUSG00000034786	3.0801	-0.5111	DOWN	Gpsm3
ENSMUSG00000034818	2.4092	-0.1739	DOWN	Celf5
ENSMUSG00000034853	3.3408	-0.2044	DOWN	Acot11
ENSMUSG00000034854	9.7406	-0.3932	DOWN	Mfsd12
ENSMUSG00000034868	1.7499	-0.3094	DOWN	Myl12b
ENSMUSG00000034930	3.7086	-0.2798	DOWN	Rtkn
ENSMUSG00000034993	2.503	-0.1475	DOWN	Vat1
ENSMUSG00000035047	5.267	-0.3431	DOWN	Kri1

ENSMUSG00000035067	1.6617	-0.272	DOWN	Xkr6
ENSMUSG00000035086	17.5179	-0.2838	DOWN	Becn1
ENSMUSG00000035109	1.4228	-0.202	DOWN	Shc4
ENSMUSG00000035150	3.487	-0.1852	DOWN	Eif2s3x
ENSMUSG00000035226	2.3792	-0.2038	DOWN	Rims4
ENSMUSG00000035227	1.75	-0.1539	DOWN	Spcs2
ENSMUSG00000035228	3.5745	-0.2728	DOWN	Ccdc106
ENSMUSG00000035248	2.5662	-0.1626	DOWN	Tut7
ENSMUSG00000035274	1.7978	-0.1976	DOWN	Tpbg
ENSMUSG00000035390	12.1928	-0.2623	DOWN	Brsk1
ENSMUSG00000035513	1.4328	-0.1751	DOWN	Ntng2
ENSMUSG00000035530	3.8452	-0.1945	DOWN	Eif1
ENSMUSG00000035632	1.7756	-0.1668	DOWN	Cnot3
ENSMUSG00000035697	1.3092	-0.2118	DOWN	Arhgap45
ENSMUSG00000035726	4.2786	-0.2095	DOWN	Supt16
ENSMUSG00000035770	4.2555	-0.1607	DOWN	Dync1li2
ENSMUSG00000035773	1.5235	-0.1894	DOWN	Kiss1r
ENSMUSG00000035781	5.1145	-0.1949	DOWN	R3hdm4
ENSMUSG00000035863	2.8722	-0.1569	DOWN	Palm
ENSMUSG00000035960	16.4999	-0.6957	DOWN	Apex1
ENSMUSG00000036120	3.8818	-0.3018	DOWN	Rfxank
ENSMUSG00000036155	7.7702	-0.2358	DOWN	Mgat5
ENSMUSG00000036167	3.1081	-0.1702	DOWN	Pphln1
ENSMUSG00000036180	5.9673	-0.2095	DOWN	Gatad2a
ENSMUSG00000036186	14.4504	-0.3392	DOWN	Dipk1b
ENSMUSG00000036306	5.4281	-0.3296	DOWN	Lzts1
ENSMUSG00000036390	1.3999	-0.218	DOWN	Gadd45a
ENSMUSG00000036430	3.8104	-0.2719	DOWN	Tbcc
ENSMUSG00000036452	2.8618	-0.1695	DOWN	Arhgap26
ENSMUSG00000036504	7.2041	-0.6724	DOWN	Phpt1
ENSMUSG00000036560	1.3293	-0.2538	DOWN	Lgi4
ENSMUSG00000036613	4.4224	-0.2121	DOWN	Eipr1
ENSMUSG00000036672	2.7101	-0.1823	DOWN	Cenpt
ENSMUSG00000036693	2.353	-0.1698	DOWN	Nop14
ENSMUSG00000036731	8.3381	-0.5611	DOWN	Cysrt1
ENSMUSG00000036775	2.4285	-0.1488	DOWN	Decr2
ENSMUSG00000036790	2.5137	-0.152	DOWN	Slitrk2
ENSMUSG00000036894	6.1155	-0.2892	DOWN	Rap2b
ENSMUSG00000036918	2.3893	-0.2546	DOWN	Ttc7
ENSMUSG00000036957	2.3517	-0.1614	DOWN	Lrfn3
ENSMUSG00000036966	7.3493	-0.1866	DOWN	Spryd3
ENSMUSG00000037010	1.3787	-0.3141	DOWN	Apln
ENSMUSG00000037071	2.8569	-0.1606	DOWN	Scd1
ENSMUSG00000037104	5.2654	-0.2148	DOWN	Socs5
ENSMUSG00000037217	2.7011	-0.1406	DOWN	Syn1

ENSMUSG00000037428	5.0966	-0.3456	DOWN	Vgf
ENSMUSG00000037432	1.5279	-0.2312	DOWN	Fer1l5
ENSMUSG00000037578	1.3031	-0.2911	DOWN	Pkd2l1
ENSMUSG00000037706	11.7155	-0.2415	DOWN	Cd81
ENSMUSG00000037826	7.2424	-0.2056	DOWN	Ppm1k
ENSMUSG00000037845	2.3635	-0.3289	DOWN	Fdxacb1
ENSMUSG00000037857	1.343	-0.1727	DOWN	Nufip2
ENSMUSG00000037905	2.178	-0.182	DOWN	Bri3bp
ENSMUSG00000038007	1.315	-0.2437	DOWN	Acer2
ENSMUSG00000038013	3.1169	-0.1964	DOWN	Wipf2
ENSMUSG00000038121	2.449	-0.1635	DOWN	Fam210a
ENSMUSG00000038146	1.5148	-0.1937	DOWN	Notch3
ENSMUSG00000038150	8.0773	-0.2338	DOWN	Ormdl3
ENSMUSG00000038152	3.7915	-1.2453	DOWN	5033430I15Rik
ENSMUSG00000038349	2.6607	-0.1944	DOWN	Plcl1
ENSMUSG00000038374	2.1846	-0.1577	DOWN	Rbm8a
ENSMUSG00000038400	4.6401	-0.2315	DOWN	Pmepa1
ENSMUSG00000038406	2.9742	-0.1553	DOWN	Scaf1
ENSMUSG00000038437	2.5666	-0.1862	DOWN	Mllt6
ENSMUSG00000038453	1.5275	-0.1592	DOWN	Srcin1
ENSMUSG00000038473	2.3949	-0.2373	DOWN	Nos1ap
ENSMUSG00000038539	2.2806	-0.2141	DOWN	Atf5
ENSMUSG00000038545	1.3982	-0.1385	DOWN	Cul7
ENSMUSG00000038593	5.1315	-0.31	DOWN	Tctn1
ENSMUSG00000038607	5.1998	-1.1015	DOWN	Gng10
ENSMUSG00000038717	52.6957	-0.8451	DOWN	Atp5l
ENSMUSG00000038738	3.564	-0.2843	DOWN	Shank1
ENSMUSG00000038742	2.2882	-0.3756	DOWN	Angptl6
ENSMUSG00000038803	9.1116	-0.469	DOWN	Ost4
ENSMUSG00000038855	2.0393	-0.1547	DOWN	Itpkb
ENSMUSG00000038872	5.0794	-0.2588	DOWN	Zfhx3
ENSMUSG00000038876	2.4865	-0.2223	DOWN	Rnf146
ENSMUSG00000038880	1.3034	-0.1599	DOWN	Mrps34
ENSMUSG00000038976	5.5785	-0.2065	DOWN	Ppp1r9b
ENSMUSG00000039037	2.6626	-0.1702	DOWN	St6galnac5
ENSMUSG00000039059	1.3545	-0.1522	DOWN	Hrh3
ENSMUSG00000039108	15.6017	-0.3354	DOWN	Lsm14b
ENSMUSG00000039114	1.4468	-0.1598	DOWN	Nrn1
ENSMUSG00000039148	3.8351	-0.1616	DOWN	Sart1
ENSMUSG00000039159	6.1921	-0.1604	DOWN	Ube2h
ENSMUSG00000039202	1.5349	-0.1561	DOWN	Abhd2
ENSMUSG00000039206	2.1765	-0.1512	DOWN	Daglb
ENSMUSG00000039221	48.312	-1.0605	DOWN	Rpl22l1
ENSMUSG00000039253	2.2648	-0.1741	DOWN	Fn3krp
ENSMUSG00000039349	2.1686	-0.1785	DOWN	C130074G19Rik

ENSMUSG00000039356	2.9716	-0.1924	DOWN	Exosc2
ENSMUSG00000039382	7.0751	-0.3125	DOWN	Wdr45
ENSMUSG00000039395	1.3425	-0.2506	DOWN	Mreg
ENSMUSG00000039452	3.2157	-0.2232	DOWN	Snx22
ENSMUSG00000039485	3.2069	-0.1588	DOWN	Tspyl4
ENSMUSG00000039492	4.7034	-0.6803	DOWN	Ccdc27
ENSMUSG00000039515	8.0314	-0.1553	DOWN	Ptpa
ENSMUSG00000039542	11.9215	-0.2184	DOWN	Ncam1
ENSMUSG00000039546	3.7257	-0.178	DOWN	Ajap1
ENSMUSG00000039556	12.2215	-0.7502	DOWN	Ppp1r3f
ENSMUSG00000039633	1.7024	-0.1476	DOWN	Lonrf1
ENSMUSG00000039648	2.0645	-0.1501	DOWN	Kyat1
ENSMUSG00000039716	1.9599	-0.1465	DOWN	Dock3
ENSMUSG00000039771	5.9907	-0.3752	DOWN	Polr2j
ENSMUSG00000039798	1.684	-0.3931	DOWN	2600006K01Rik
ENSMUSG00000039810	4.4224	-0.2667	DOWN	Zc3h10
ENSMUSG00000039835	1.9907	-0.2029	DOWN	Nhsl1
ENSMUSG00000040007	2.405	-0.1801	DOWN	Bahd1
ENSMUSG00000040028	7.2875	-0.2487	DOWN	Elavl1
ENSMUSG00000040043	2.0324	-0.2329	DOWN	Rbms2
ENSMUSG00000040093	1.4461	-0.338	DOWN	Bmf
ENSMUSG00000040148	1.3969	-0.249	DOWN	Hmx3
ENSMUSG00000040263	2.6055	-0.185	DOWN	Klhdc4
ENSMUSG00000040289	3.0935	-0.1657	DOWN	Hey1
ENSMUSG00000040302	1.3366	-0.1712	DOWN	Rbm48
ENSMUSG00000040423	5.5087	-0.2221	DOWN	Rc3h1
ENSMUSG00000040424	6.29	-0.261	DOWN	Hipk4
ENSMUSG00000040490	1.8886	-0.1902	DOWN	Lrfn2
ENSMUSG00000040606	3.3695	-0.15	DOWN	Kazn
ENSMUSG00000040649	2.6162	-0.1602	DOWN	Rimklb
ENSMUSG00000040731	11.5996	-0.1889	DOWN	Eif4h
ENSMUSG00000040842	27.6679	-0.5385	DOWN	Szrd1
ENSMUSG00000040904	2.6129	-1.5813	DOWN	Gm21988
ENSMUSG00000040929	1.5543	-0.1452	DOWN	Rfx3
ENSMUSG00000040945	4.0265	-0.1535	DOWN	Rcc2
ENSMUSG00000040990	2.5563	-0.1481	DOWN	Sh3kbp1
ENSMUSG00000041025	3.3655	-0.205	DOWN	Iffo2
ENSMUSG00000041035	1.5634	-0.1879	DOWN	Dpcd
ENSMUSG00000041040	3.2738	-0.1501	DOWN	Fam117b
ENSMUSG00000041133	3.2259	-0.1683	DOWN	Smc1a
ENSMUSG00000041263	19.015	-0.2728	DOWN	Rusc1
ENSMUSG00000041375	3.1175	-0.1997	DOWN	Ccdc9
ENSMUSG00000041417	3.0986	-0.1566	DOWN	Pik3r1
ENSMUSG00000041625	3.6956	-0.2106	DOWN	Ggact
ENSMUSG00000041671	2.7945	-0.2883	DOWN	Pyroxd1

ENSMUSG00000041837	3.7475	-0.2221	DOWN	Pdcd7
ENSMUSG00000042066	2.7979	-0.1889	DOWN	Tmcc2
ENSMUSG00000042073	2.4738	-0.2188	DOWN	Abhd14b
ENSMUSG00000042190	1.3819	-0.3235	DOWN	Cmklr1
ENSMUSG00000042202	1.4164	-0.1513	DOWN	Slc35e2
ENSMUSG00000042203	3.8298	-0.1756	DOWN	Tbc1d22b
ENSMUSG00000042275	5.1883	-0.6102	DOWN	Pelo
ENSMUSG00000042320	1.3984	-0.2472	DOWN	Prox2
ENSMUSG00000042348	1.5604	-0.17	DOWN	Arl15
ENSMUSG00000042390	6.7561	-0.2257	DOWN	Gatad2b
ENSMUSG00000042419	1.7283	-0.2847	DOWN	Nfkbil1
ENSMUSG00000042492	5.0545	-0.1766	DOWN	Tbc1d10b
ENSMUSG00000042524	3.7058	-0.2034	DOWN	Sun2
ENSMUSG00000042532	9.2915	-0.2613	DOWN	Golga7b
ENSMUSG00000042589	2.4211	-0.191	DOWN	Cux2
ENSMUSG00000042595	3.367	-0.2479	DOWN	Fam199x
ENSMUSG00000042604	2.0788	-0.1427	DOWN	Kcna4
ENSMUSG00000042606	8.3691	-0.3048	DOWN	Hirip3
ENSMUSG00000042650	2.4391	-0.1911	DOWN	Alkbh5
ENSMUSG00000042751	11.918	-0.2037	DOWN	Nmnat2
ENSMUSG00000042757	3.5719	-0.206	DOWN	Tmem108
ENSMUSG00000042761	2.6537	-0.2345	DOWN	Mrap2
ENSMUSG00000042831	49.3279	-0.7925	DOWN	Alkbh6
ENSMUSG00000042873	15.2259	-0.3348	DOWN	Lhfpl4
ENSMUSG00000042903	2.9712	-0.2291	DOWN	Foxo4
ENSMUSG00000043004	3.2582	-0.1584	DOWN	Gng2
ENSMUSG00000043311	2.3721	-0.2332	DOWN	D17H6S53E
ENSMUSG00000043460	1.4823	-0.1765	DOWN	Elfn2
ENSMUSG00000043670	4.5055	-0.1949	DOWN	Diras1
ENSMUSG00000043687	1.6215	-0.6242	DOWN	1190005I06Rik
ENSMUSG00000043962	6.9218	-0.2386	DOWN	Thrap3
ENSMUSG00000044024	12.732	-0.3889	DOWN	Rell2
ENSMUSG00000044068	11.8673	-0.3392	DOWN	Zrsr1
ENSMUSG00000044147	2.0202	-0.1532	DOWN	Arf6
ENSMUSG00000044164	1.5109	-0.1632	DOWN	Rnf182
ENSMUSG00000044165	2.4316	-0.651	DOWN	Bcl2l15
ENSMUSG00000044211	1.4587	-0.2284	DOWN	Gm7887
ENSMUSG00000044477	6.0973	-0.1788	DOWN	Zfand3
ENSMUSG00000044708	1.8638	-0.1378	DOWN	Kcnj10
ENSMUSG00000044788	1.6457	-0.1458	DOWN	Fads6
ENSMUSG00000044795	10.4784	-0.3824	DOWN	Cyb5d1
ENSMUSG00000044912	3.8748	-0.1703	DOWN	Syt16
ENSMUSG00000044927	8.0476	-0.4191	DOWN	H1f10
ENSMUSG00000045005	1.733	-0.3515	DOWN	Fzd5
ENSMUSG00000045106	1.9105	-0.1858	DOWN	Ccdc73

ENSMUSG00000045392	1.4072	-0.6937	DOWN	Olfr1033
ENSMUSG00000045435	4.9636	-0.3848	DOWN	Tmem60
ENSMUSG00000045438	1.9863	-0.1573	DOWN	Cox19
ENSMUSG00000045515	3.481	-0.2508	DOWN	Pou3f3
ENSMUSG00000045763	1.6344	-0.1575	DOWN	Basp1
ENSMUSG00000045790	4.0038	-0.1601	DOWN	Ccdc149
ENSMUSG00000046056	1.4949	-0.2231	DOWN	Sbsn
ENSMUSG00000046062	8.0763	-0.2185	DOWN	Ppp1r15b
ENSMUSG00000046269	13.9722	-0.3523	DOWN	Usp27x
ENSMUSG00000046434	19.8001	-0.3067	DOWN	Hnrnpa1
ENSMUSG00000046447	8.5481	-0.25	DOWN	Camk2n1
ENSMUSG00000046574	1.7758	-0.1914	DOWN	Prr12
ENSMUSG00000046711	2.6939	-0.195	DOWN	Hmga1
ENSMUSG00000046805	2.5801	-0.2553	DOWN	Mpeg1
ENSMUSG00000046876	8.2414	-0.2033	DOWN	Atxn1
ENSMUSG00000046958	1.3599	-0.6623	DOWN	4930432E11Rik
ENSMUSG00000047045	1.9374	-0.1727	DOWN	Tmem164
ENSMUSG00000047085	16.841	-0.2899	DOWN	Lrrc4b
ENSMUSG00000047261	2.2357	-0.1641	DOWN	Gap43
ENSMUSG00000047379	7.5432	-1.3322	DOWN	B4gat1
ENSMUSG00000047514	6.3139	-0.2199	DOWN	Tspyl1
ENSMUSG00000047606	2.0513	-0.2047	DOWN	Ankrd34c
ENSMUSG00000047654	2.8328	-0.639	DOWN	Tssk6
ENSMUSG00000047658	15.6227	-0.4704	DOWN	Gal3st3
ENSMUSG00000047731	2.3382	-0.1417	DOWN	Wbp1l
ENSMUSG00000047747	2.6534	-0.1726	DOWN	Rnf150
ENSMUSG00000047786	1.5347	-0.2121	DOWN	Lix1
ENSMUSG00000047888	3.4704	-0.1764	DOWN	Tnrc6b
ENSMUSG00000047986	1.3054	-0.2945	DOWN	Palm3
ENSMUSG00000047988	6.4092	-0.5285	DOWN	4933428G20Rik
ENSMUSG00000048022	10.4894	-0.284	DOWN	Tmem229a
ENSMUSG00000048039	1.6519	-0.1798	DOWN	Isg20l2
ENSMUSG00000048047	3.1982	-0.1647	DOWN	Zbtb33
ENSMUSG00000048100	4.3251	-0.303	DOWN	Taf13
ENSMUSG00000048142	3.7877	-0.2083	DOWN	Nat8l
ENSMUSG00000048251	2.1902	-0.2546	DOWN	Bcl11b
ENSMUSG00000048332	3.8449	-0.243	DOWN	Lhfp
ENSMUSG00000048429	8.3737	-0.1917	DOWN	Timm29
ENSMUSG00000048481	3.6118	-0.2726	DOWN	Mypop
ENSMUSG00000048483	4.4708	-0.205	DOWN	Zdhhc22
ENSMUSG00000048731	5.0511	-0.5394	DOWN	Ggnbp1
ENSMUSG00000048787	2.5315	-0.2092	DOWN	Dcun1d3
ENSMUSG00000048827	2.1802	-0.5019	DOWN	Pkd1l3
ENSMUSG00000048897	1.6361	-0.1765	DOWN	Zfp710
ENSMUSG00000048915	3.3829	-0.2389	DOWN	Efna5

ENSMUSG00000049086	1.6368	-0.263	DOWN	Bmyc
ENSMUSG00000049265	9.2225	-0.3007	DOWN	Kcnk3
ENSMUSG00000049295	4.18	-0.2391	DOWN	Zfp219
ENSMUSG00000049303	1.9891	-0.1621	DOWN	Syt12
ENSMUSG00000049422	50.4238	-0.8706	DOWN	Chchd10
ENSMUSG00000049470	3.7789	-0.1403	DOWN	Aff4
ENSMUSG00000049511	1.5275	-0.2111	DOWN	Htr1b
ENSMUSG00000049672	2.9697	-0.1784	DOWN	Zbtb14
ENSMUSG00000049751	1.395	-0.463	DOWN	Rpl36al
ENSMUSG00000049823	14.5843	-0.5932	DOWN	Zbtb12
ENSMUSG00000049932	4.9212	-0.2784	DOWN	H2ax
ENSMUSG00000050017	7.1128	-0.2576	DOWN	Pitpnb
ENSMUSG00000050064	1.5677	-0.1787	DOWN	Zfp697
ENSMUSG00000050100	1.4413	-0.297	DOWN	Hmx2
ENSMUSG00000050288	1.6483	-0.23	DOWN	Fzd2
ENSMUSG00000050357	5.3401	-0.2426	DOWN	Carmil2
ENSMUSG00000050708	25.8931	-0.5518	DOWN	Ftl1
ENSMUSG00000050751	4.2148	-0.161	DOWN	Pgbd5
ENSMUSG00000050860	6.758	-1.4609	DOWN	Phospho1
ENSMUSG00000050891	2.0915	-0.2096	DOWN	Tatdn1
ENSMUSG00000050908	3.2598	-0.2253	DOWN	Tvp23a
ENSMUSG00000050910	1.3399	-0.1616	DOWN	Cdr2l
ENSMUSG00000050989	3.5272	-0.1899	DOWN	Selenon
ENSMUSG00000051149	2.5315	-0.7302	DOWN	Adnp
ENSMUSG00000051184	4.1871	-0.7703	DOWN	Zfp524
ENSMUSG00000051323	9.1498	-0.1947	DOWN	Pcdh19
ENSMUSG00000051355	9.8804	-0.4932	DOWN	Commd1
ENSMUSG00000051391	7.9004	-0.1825	DOWN	Ywhag
ENSMUSG00000051401	2.6526	-0.3606	DOWN	Kctd16
ENSMUSG00000051435	2.6941	-0.2066	DOWN	Fhad1
ENSMUSG00000051495	2.6556	-0.2665	DOWN	Irf2bp2
ENSMUSG00000051527	9.6205	-0.2316	DOWN	Usp29
ENSMUSG00000051537	3.1445	-0.2059	DOWN	Gm5124
ENSMUSG00000051550	1.6035	-0.1698	DOWN	Zfp579
ENSMUSG00000051627	2.1727	-0.6159	DOWN	H1f4
ENSMUSG00000051650	27.5003	-1.3813	DOWN	B3gnt2
ENSMUSG00000051790	3.565	-0.171	DOWN	Nlgn2
ENSMUSG00000051853	6.8484	-0.1956	DOWN	Arf3
ENSMUSG00000051855	3.509	-0.2764	DOWN	Mest
ENSMUSG00000051965	2.6782	-0.8329	DOWN	Nanos2
ENSMUSG00000052031	1.8307	-0.1545	DOWN	Tagap1
ENSMUSG00000052040	6.9133	-0.3417	DOWN	Klf13
ENSMUSG00000052056	1.3831	-0.2165	DOWN	Zfp217
ENSMUSG00000052105	2.3307	-0.2047	DOWN	Mtcl1
ENSMUSG00000052144	2.6371	-0.1688	DOWN	Ppp4r2

ENSMUSG00000052188	2.5883	-0.3661	DOWN	Gm14964
ENSMUSG00000052241	3.5022	-1.0298	DOWN	A930035D04Rik
ENSMUSG00000052293	2.0704	-1.2953	DOWN	Taf9
ENSMUSG00000052310	2.747	-0.2246	DOWN	Slc39a1
ENSMUSG00000052525	1.9561	-0.4609	DOWN	Spdya
ENSMUSG00000052581	3.0179	-0.2571	DOWN	Lrrtm4
ENSMUSG00000052629	2.0788	-0.3087	DOWN	Gm9885
ENSMUSG00000052632	1.6867	-0.1452	DOWN	Asap2
ENSMUSG00000052759	1.4423	-0.7559	DOWN	Gpr25
ENSMUSG00000052794	2.6536	-0.1889	DOWN	1700030K09Rik
ENSMUSG00000053093	21.3791	-0.8116	DOWN	Myh7
ENSMUSG00000053128	10.0597	-0.8478	DOWN	Rnf26
ENSMUSG00000053166	2.2616	-0.2057	DOWN	Cdh22
ENSMUSG00000053192	6.5088	-0.2299	DOWN	Mllt11
ENSMUSG00000053291	18.3578	-1.2869	DOWN	Rab4b
ENSMUSG00000053293	1.4413	-0.1411	DOWN	Pom121
ENSMUSG00000053310	2.1549	-0.2269	DOWN	Nrgn
ENSMUSG00000053353	1.5694	-1.4586	DOWN	2310001K24Rik
ENSMUSG00000053395	4.4228	-0.3026	DOWN	Cacng8
ENSMUSG00000053453	1.9642	-0.1852	DOWN	Thoc7
ENSMUSG00000053460	1.8806	-0.1624	DOWN	Ggcx
ENSMUSG00000053536	9.8509	-0.2244	DOWN	Cstf2t
ENSMUSG00000053604	2.732	-0.2112	DOWN	Rpia
ENSMUSG00000053626	2.356	-0.4499	DOWN	Tll1
ENSMUSG00000053769	4.2343	-0.3144	DOWN	Lysmd1
ENSMUSG00000053877	2.3551	-0.4795	DOWN	Srcap
ENSMUSG00000054034	22.3848	-0.419	DOWN	Tceal5
ENSMUSG00000054256	6.813	-0.2791	DOWN	Msi1
ENSMUSG00000054280	2.0313	-0.1781	DOWN	Prr14l
ENSMUSG00000054408	2.3519	-0.1379	DOWN	Spcs3
ENSMUSG00000054517	2.2038	-0.2803	DOWN	Trim65
ENSMUSG00000054619	2.874	-0.2314	DOWN	Mettl7a1
ENSMUSG00000054716	2.7092	-0.2233	DOWN	Zfp771
ENSMUSG00000054717	1.418	-0.3392	DOWN	Hmgb2
ENSMUSG00000054766	24.2052	-0.312	DOWN	Set
ENSMUSG00000054931	1.3705	-0.2592	DOWN	Zkscan4
ENSMUSG00000054934	24.9517	-1.6015	DOWN	Kcnmb4
ENSMUSG00000055148	1.3229	-0.4589	DOWN	Klf2
ENSMUSG00000055302	9.5373	-0.307	DOWN	Mrfap1
ENSMUSG00000055447	7.7942	-0.211	DOWN	Cd47
ENSMUSG00000055633	1.983	-0.2004	DOWN	Zfp580
ENSMUSG00000055675	2.6814	-0.157	DOWN	Kbtbd11
ENSMUSG00000055725	1.5037	-0.2332	DOWN	Paqr3
ENSMUSG00000055799	1.4353	-0.2345	DOWN	Tcf7l1
ENSMUSG00000055805	3.1001	-0.1948	DOWN	Fmnl1

ENSMUSG00000056076	2.3675	-0.143	DOWN	Eif3b
ENSMUSG00000056486	14.1498	-0.2387	DOWN	Chn1
ENSMUSG00000056501	3.6489	-0.6426	DOWN	Cebpb
ENSMUSG00000056508	13.6092	-0.5939	DOWN	1700001K19Rik
ENSMUSG00000056537	4.1896	-0.1841	DOWN	Rlim
ENSMUSG00000056666	1.7658	-0.1511	DOWN	Retsat
ENSMUSG00000056708	1.6175	-0.2058	DOWN	Ier5
ENSMUSG00000056851	13.9934	-0.2826	DOWN	Pcbp2
ENSMUSG00000056888	1.6411	-0.4537	DOWN	Glipr1
ENSMUSG00000057060	1.9256	-0.1587	DOWN	Slc35f3
ENSMUSG00000057103	2.2076	-0.2594	DOWN	Nat8f1
ENSMUSG00000057137	2.1635	-0.6603	DOWN	Tmem140
ENSMUSG00000057411	5.7425	-0.2637	DOWN	Antkmt
ENSMUSG00000057522	16.1742	-0.2642	DOWN	Spop
ENSMUSG00000057561	4.5773	-0.1652	DOWN	Eif1a
ENSMUSG00000057605	2.3423	-0.7006	DOWN	Gm6807
ENSMUSG00000057789	2.3528	-0.2477	DOWN	Bak1
ENSMUSG00000058239	1.6519	-0.1612	DOWN	Usf2
ENSMUSG00000058297	6.9632	-0.2243	DOWN	Spock2
ENSMUSG00000058446	1.7756	-0.1435	DOWN	Znrf2
ENSMUSG00000058546	135.1198	-1.3165	DOWN	Rpl23a
ENSMUSG00000058586	1.8917	-0.2921	DOWN	Serhl
ENSMUSG00000058600	2.9388	-0.4093	DOWN	Rpl30
ENSMUSG00000058799	5.1818	-0.1785	DOWN	Nap1l1
ENSMUSG00000058922	24.1035	-0.5708	DOWN	Gm10052
ENSMUSG00000059003	3.0987	-0.1981	DOWN	Grin2a
ENSMUSG00000059040	24.7057	-0.6931	DOWN	Eno1b
ENSMUSG00000059278	9.8474	-0.4737	DOWN	Naa38
ENSMUSG00000059436	3.2828	-0.1853	DOWN	Max
ENSMUSG00000059810	4.7725	-0.2164	DOWN	Rgs3
ENSMUSG00000059981	1.6424	-0.1504	DOWN	Taok2
ENSMUSG00000060029	1.5726	-0.4013	DOWN	4930473A02Rik
ENSMUSG00000060180	1.3721	-0.5928	DOWN	Myh13
ENSMUSG00000060181	4.8513	-0.2097	DOWN	Slc35e3
ENSMUSG00000060206	2.0851	-0.1569	DOWN	Zfp462
ENSMUSG00000060257	1.433	-0.256	DOWN	Scrt2
ENSMUSG00000060376	2.4193	-0.2814	DOWN	Bckdha
ENSMUSG00000060380	1.5969	-1.3682	DOWN	C030014I23Rik
ENSMUSG00000060477	2.6768	-0.1907	DOWN	Irak2
ENSMUSG00000060510	4.7411	-0.1774	DOWN	Zfp266
ENSMUSG00000060538	7.0294	-0.4583	DOWN	Tmem219
ENSMUSG00000060860	2.2636	-0.1495	DOWN	Ube2s
ENSMUSG00000060935	15.4922	-0.3532	DOWN	Tmem263
ENSMUSG00000060989	11.3062	-0.7612	DOWN	Gm11847
ENSMUSG00000061046	2.6676	-0.1554	DOWN	Haghl

ENSMUSG00000061099	2.2662	-0.5108	DOWN	Gapdhs
ENSMUSG00000061118	11.7611	-0.4402	DOWN	Dnajc30
ENSMUSG00000061353	2.9689	-0.2923	DOWN	Cxcl12
ENSMUSG00000061718	4.9586	-0.3306	DOWN	Ppp1r1b
ENSMUSG00000062078	3.5745	-0.2095	DOWN	Qk
ENSMUSG00000062081	21.9489	-0.9505	DOWN	Gm6055
ENSMUSG00000062270	5.5877	-0.1887	DOWN	Morf4l1
ENSMUSG00000062353	3.6959	-0.4583	DOWN	Gm15772
ENSMUSG00000062526	2.8343	-0.2296	DOWN	Mppe1
ENSMUSG00000062661	7	-0.1692	DOWN	Ncs1
ENSMUSG00000062691	1.5014	-0.297	DOWN	Cebpzos
ENSMUSG00000062825	9.7161	-0.3422	DOWN	Actg1
ENSMUSG00000062944	2.3077	-0.3523	DOWN	9130023H24Rik
ENSMUSG00000062997	3.2385	-0.277	DOWN	Rpl35
ENSMUSG00000063015	16.3316	-0.3251	DOWN	Ccni
ENSMUSG00000063146	3.935	-0.1639	DOWN	Clip2
ENSMUSG00000063235	9.2959	-0.399	DOWN	Ptpmt1
ENSMUSG00000063506	3.3431	-0.4089	DOWN	Arhgap22
ENSMUSG00000063535	1.7176	-0.4135	DOWN	Zfp773
ENSMUSG00000063556	9.8561	-1.072	DOWN	Gm10132
ENSMUSG00000063646	14.0921	-0.4594	DOWN	Jakmip1
ENSMUSG00000063785	1.3145	-0.161	DOWN	Utp14a
ENSMUSG00000063856	2.669	-0.456	DOWN	Gpx1
ENSMUSG00000064125	3.2236	-0.2168	DOWN	Prr36
ENSMUSG00000064247	5.0377	-0.2597	DOWN	Plcxd1
ENSMUSG00000064307	2.5075	-0.4541	DOWN	Lrrc51
ENSMUSG00000064356	16.9398	-2.6237	DOWN	mt-Atp8
ENSMUSG00000064368	10.7533	-0.3913	DOWN	mt-Nd6
ENSMUSG00000064372	13.6546	-0.9886	DOWN	mt-Tp
ENSMUSG00000064377	3.9583	-0.5561	DOWN	Gm24966
ENSMUSG00000064380	1.7474	-1.5423	DOWN	Gm26448
ENSMUSG00000064658	2.4343	-1.2096	DOWN	Gm24166
ENSMUSG00000064871	3.2819	-1.0286	DOWN	Snord58b
ENSMUSG00000064916	2.0795	-0.8738	DOWN	Gm22573
ENSMUSG00000065431	1.7268	-0.5449	DOWN	Mir186
ENSMUSG00000065470	4.3417	-1.1706	DOWN	Mir149
ENSMUSG00000065485	9.5022	-2.261	DOWN	Mir219a-2
ENSMUSG00000065521	2.8052	-2.6216	DOWN	Mir296
ENSMUSG00000065524	9.2032	-1.1366	DOWN	Mir135a-2
ENSMUSG00000065530	2.8066	-1.2689	DOWN	Mir99a
ENSMUSG00000065557	1.9056	-1.8003	DOWN	Mirlet7c-1
ENSMUSG00000065637	1.3294	-0.7559	DOWN	Gm26397
ENSMUSG00000065676	1.5753	-0.6575	DOWN	Snord42b
ENSMUSG00000065947	4.3189	-1.7043	DOWN	mt-Nd4l
ENSMUSG00000066058	1.5007	-0.6435	DOWN	Cldn19

ENSMUSG00000066149	13.6137	-0.3715	DOWN	Cdc26
ENSMUSG00000066456	1.5688	-0.1512	DOWN	Hmgn3
ENSMUSG00000066538	2.6254	-0.9134	DOWN	Gm6254
ENSMUSG00000066551	14.5208	-0.3663	DOWN	Hmgb1
ENSMUSG00000066640	3.8558	-0.2566	DOWN	Fbxl18
ENSMUSG00000066798	2.3786	-0.2288	DOWN	Zbtb6
ENSMUSG00000066877	1.5982	-0.1559	DOWN	Nck2
ENSMUSG00000066892	3.0384	-0.2247	DOWN	Fbxl12
ENSMUSG00000066900	2.3517	-0.1438	DOWN	Suds3
ENSMUSG00000067038	3.4143	-0.3318	DOWN	Rps12-ps3
ENSMUSG00000067547	28.3214	-1.2485	DOWN	Gm7666
ENSMUSG00000067629	3.1717	-0.2747	DOWN	Syngap1
ENSMUSG00000067653	3.2196	-0.7662	DOWN	Ankrd23
ENSMUSG00000067713	8.051	-0.3939	DOWN	Prkag1
ENSMUSG00000067929	1.3265	-0.4123	DOWN	Gm10226
ENSMUSG00000068039	6.0241	-0.1966	DOWN	Tcp1
ENSMUSG00000068099	11.4958	-0.2897	DOWN	Smim45
ENSMUSG00000068134	1.6253	-0.1721	DOWN	Zfp120
ENSMUSG00000068141	2.1065	-0.342	DOWN	Gm10232
ENSMUSG00000068206	6.2336	-0.2878	DOWN	Pick1
ENSMUSG00000068240	17.9858	-0.5445	DOWN	Gm11808
ENSMUSG00000068267	2.7386	-0.2301	DOWN	Cenpb
ENSMUSG00000068396	16.3653	-0.7394	DOWN	Rpl34-ps1
ENSMUSG00000068579	1.9246	-0.955	DOWN	Rpl7a-ps3
ENSMUSG00000068732	4.9319	-0.1545	DOWN	Tmem167b
ENSMUSG00000068823	12.2938	-0.2205	DOWN	Csde1
ENSMUSG00000068966	4.9521	-0.3018	DOWN	Zbtb34
ENSMUSG00000069014	5.9104	-1.4642	DOWN	Gm5641
ENSMUSG00000069272	3.1299	-1.755	DOWN	H2ac8
ENSMUSG00000069662	5.9907	-0.226	DOWN	Marcks
ENSMUSG00000069769	9.8384	-0.3203	DOWN	Msi2
ENSMUSG00000069806	2.7583	-0.1905	DOWN	Cacng7
ENSMUSG00000070167	2.7462	-0.8946	DOWN	Snora57
ENSMUSG00000070284	2.33	-0.313	DOWN	Gmppb
ENSMUSG00000070304	4.3865	-0.1964	DOWN	Scn2b
ENSMUSG00000070348	4.7617	-0.2235	DOWN	Ccnd1
ENSMUSG00000070498	2.5816	-0.1647	DOWN	Tmem132b
ENSMUSG00000070509	1.9452	-0.15	DOWN	Rgma
ENSMUSG00000070532	4.7933	-0.4075	DOWN	Ccdc190
ENSMUSG00000070697	3.9717	-0.2271	DOWN	Utp3
ENSMUSG00000071014	4.5559	-0.3472	DOWN	Ndufb6
ENSMUSG00000071064	2.2218	-0.1529	DOWN	Zfp827
ENSMUSG00000071072	2.5052	-0.2519	DOWN	Ptges3
ENSMUSG00000071076	44.1553	-0.77	DOWN	Jund
ENSMUSG00000071265	6.1966	-0.4297	DOWN	1700086L19Rik

ENSMUSG00000071341	1.7758	-0.5736	DOWN	Egr4
ENSMUSG00000071533	12.8128	-0.291	DOWN	Pcnp
ENSMUSG00000071647	5.0939	-0.2953	DOWN	Eml3
ENSMUSG00000071648	2.0907	-0.2303	DOWN	Rom1
ENSMUSG00000071659	7.2157	-0.2303	DOWN	Hnrnpul2
ENSMUSG00000071757	1.7248	-0.157	DOWN	Zhx2
ENSMUSG00000071796	2.2815	-0.1783	DOWN	6820431F20Rik
ENSMUSG00000071856	7.2694	-0.2839	DOWN	Mcc
ENSMUSG00000072214	52.012	-0.5989	DOWN	Septin5
ENSMUSG00000072494	6.9409	-0.3239	DOWN	Ppp1r3e
ENSMUSG00000072591	1.3433	-0.3218	DOWN	Fzd10os
ENSMUSG00000072772	4.5748	-0.4501	DOWN	Grcc10
ENSMUSG00000072847	2.5251	-0.2508	DOWN	A530017D24Rik
ENSMUSG00000073096	1.9906	-0.2129	DOWN	Lrrc61
ENSMUSG00000073405	1.9677	-0.3705	DOWN	H2-T-ps
ENSMUSG00000073411	1.8061	-0.168	DOWN	H2-D1
ENSMUSG00000073639	10.6358	-0.2149	DOWN	Rab18
ENSMUSG00000073680	2.255	-0.2683	DOWN	Tmem88b
ENSMUSG00000073755	12.3828	-0.2693	DOWN	5730409E04Rik
ENSMUSG00000073771	2.7433	-0.3422	DOWN	Btbd19
ENSMUSG00000073787	2.3992	-0.7001	DOWN	Gm10575
ENSMUSG00000073910	3.1277	-0.3885	DOWN	Mob3b
ENSMUSG00000074030	2.139	-0.1635	DOWN	Exoc8
ENSMUSG00000074102	2.786	-0.1394	DOWN	Rbm15b
ENSMUSG00000074129	2.1688	-0.2204	DOWN	Rpl13a
ENSMUSG00000074182	2.8683	-0.2028	DOWN	Znhit6
ENSMUSG00000074211	1.9178	-0.3832	DOWN	Sdhaf1
ENSMUSG00000074238	4.5612	-0.1674	DOWN	Ap1ar
ENSMUSG00000074269	1.3089	-0.4309	DOWN	Rec114
ENSMUSG00000074513	8.8519	-0.614	DOWN	Arfip1
ENSMUSG00000074607	6.2344	-0.1951	DOWN	Tox2
ENSMUSG00000074643	2.8442	-1.1769	DOWN	Cpne1
ENSMUSG00000074649	7.6609	-0.3088	DOWN	BC029722
ENSMUSG00000074782	1.9185	-0.2424	DOWN	4833422C13Rik
ENSMUSG00000074800	30.9459	-1.5971	DOWN	Gm4149
ENSMUSG00000074884	29.4914	-0.8812	DOWN	Serf2
ENSMUSG00000074918	15.7518	-0.3856	DOWN	Inafm2
ENSMUSG00000075254	1.6746	-0.2589	DOWN	Heg1
ENSMUSG00000075268	1.4659	-0.5293	DOWN	Gm10819
ENSMUSG00000075576	2.1257	-0.5177	DOWN	Gm12359
ENSMUSG00000075585	4.6967	-0.2547	DOWN	6330403L08Rik
ENSMUSG00000075595	10.2908	-0.2538	DOWN	Zfp652
ENSMUSG00000076052	3.111	-0.917	DOWN	Mir541
ENSMUSG00000076269	4.1853	-1.5712	DOWN	Mir374b
ENSMUSG00000076315	9.7194	-1.336	DOWN	Mir343

ENSMUSG00000076435	2.2971	-0.1639	DOWN	Acsf2
ENSMUSG00000077450	4.2648	-0.1851	DOWN	Rab11b
ENSMUSG00000077493	1.5107	-0.3524	DOWN	Snord91a
ENSMUSG00000077704	1.9054	-0.842	DOWN	Snord89
ENSMUSG00000078201	5.2387	-0.4419	DOWN	Tmem203
ENSMUSG00000078202	1.6645	-0.352	DOWN	Nrarp
ENSMUSG00000078235	2.1251	-0.1882	DOWN	Fam43b
ENSMUSG00000078453	1.3281	-0.3993	DOWN	Abracl
ENSMUSG00000078532	7.3188	-0.3126	DOWN	Nkain1
ENSMUSG00000078566	15.3179	-0.4083	DOWN	Bnip3
ENSMUSG00000078622	11.9044	-0.2051	DOWN	Ccdc47
ENSMUSG00000078656	2.2614	-0.813	DOWN	Vps25
ENSMUSG00000078684	1.9375	-0.3303	DOWN	5830417I10Rik
ENSMUSG00000078813	1.8244	-0.2156	DOWN	Leng1
ENSMUSG00000078887	3.6118	-1.659	DOWN	Gm6710
ENSMUSG00000079003	4.3953	-0.3573	DOWN	Samd1
ENSMUSG00000079019	1.3522	-0.9834	DOWN	Insl3
ENSMUSG00000079056	4.134	-0.1829	DOWN	Kcnip3
ENSMUSG00000079061	1.8321	-1.2409	DOWN	Gm11042
ENSMUSG00000079111	2.4825	-0.161	DOWN	Kdelr2
ENSMUSG00000079224	1.5608	-0.4417	DOWN	Gm6565
ENSMUSG00000079297	1.3438	-0.3133	DOWN	Gm2223
ENSMUSG00000079415	1.9116	-1.348	DOWN	Cntf
ENSMUSG00000079426	8.7336	-0.2878	DOWN	Arpc4
ENSMUSG00000079470	1.5891	-0.2689	DOWN	Utp14b
ENSMUSG00000079484	4.1001	-0.5135	DOWN	Phyhd1
ENSMUSG00000079499	2.4368	-0.3134	DOWN	6530402F18Rik
ENSMUSG00000079511	2.2105	-0.4776	DOWN	Gm42688
ENSMUSG00000079550	2.5183	-0.4827	DOWN	Mpp4
ENSMUSG00000080364	2.9173	-0.8511	DOWN	Gm25777
ENSMUSG00000080645	2.4545	-1.5532	DOWN	Mir1198
ENSMUSG00000080797	1.337	-0.375	DOWN	Gm15760
ENSMUSG00000081152	3.0376	-0.853	DOWN	Gm12430
ENSMUSG00000081382	3.0532	-1.1827	DOWN	Rpl18-ps1
ENSMUSG00000081485	2.4807	-0.3083	DOWN	Gm12338
ENSMUSG00000081603	2.4884	-0.4375	DOWN	Gm14681
ENSMUSG00000081738	1.8109	-0.6504	DOWN	Hmgb1-ps2
ENSMUSG00000081752	15.374	-0.4785	DOWN	Sms-ps
ENSMUSG00000081809	3.039	-1.4056	DOWN	Gm15539
ENSMUSG00000082063	1.6344	-0.417	DOWN	Gm12993
ENSMUSG00000082144	3.671	-0.6743	DOWN	Gm12788
ENSMUSG00000082329	7.541	-0.7368	DOWN	Gm14287
ENSMUSG00000082383	1.3275	-1.0201	DOWN	Gm9670
ENSMUSG00000082585	5.7389	-1.2171	DOWN	Gm15387
ENSMUSG00000082724	1.7199	-1.2088	DOWN	Gm14416

ENSMUSG00000083307	11.3759	-0.4979	DOWN	AA414768
ENSMUSG00000083353	1.6948	-0.696	DOWN	Gm12540
ENSMUSG00000083596	7.9123	-0.5043	DOWN	Rpl21-ps15
ENSMUSG00000083658	1.7591	-2.1754	DOWN	Gm15798
ENSMUSG00000083811	2.1068	-0.709	DOWN	Gm13071
ENSMUSG00000084106	6.4409	-0.341	DOWN	Gm6136
ENSMUSG00000084407	4.1193	-0.8311	DOWN	Gm14018
ENSMUSG00000084758	1.8415	-2.1884	DOWN	Gm12798
ENSMUSG00000084797	1.6253	-0.5686	DOWN	Gm14321
ENSMUSG00000084799	7.1972	-0.5185	DOWN	Ino80dos
ENSMUSG00000084803	1.5347	-0.7059	DOWN	5830444B04Rik
ENSMUSG00000084808	1.3467	-0.3102	DOWN	9430091E24Rik
ENSMUSG00000084880	9.6024	-0.8173	DOWN	Tomm6os
ENSMUSG00000084899	2.1515	-0.6063	DOWN	Gm15344
ENSMUSG00000084910	1.3042	-0.238	DOWN	C630043F03Rik
ENSMUSG00000084925	1.3362	-0.6176	DOWN	1810062O18Rik
ENSMUSG00000084941	1.3848	-0.4818	DOWN	Gm11944
ENSMUSG00000084946	1.7423	-0.2098	DOWN	Dlx1as
ENSMUSG00000084959	2.0438	-1.9787	DOWN	4933407I08Rik
ENSMUSG00000085001	1.4398	-0.7233	DOWN	Rapgef4os2
ENSMUSG00000085010	1.3845	-1.8937	DOWN	Gssos1
ENSMUSG00000085151	1.3559	-0.2948	DOWN	1110018N20Rik
ENSMUSG00000085178	1.3786	-0.6608	DOWN	Kdm6bos
ENSMUSG00000085181	3.7924	-0.509	DOWN	Gm12709
ENSMUSG00000085213	2.4316	-0.5304	DOWN	Gm13091
ENSMUSG00000085227	2.7351	-0.6625	DOWN	6330418K02Rik
ENSMUSG00000085317	1.5723	-1.207	DOWN	Gssos2
ENSMUSG00000085334	1.5318	-0.1721	DOWN	Gm12940
ENSMUSG00000085403	1.4272	-0.778	DOWN	Gm13068
ENSMUSG00000085442	17.9217	-2.0164	DOWN	Gm3362
ENSMUSG00000085705	1.6281	-0.5945	DOWN	Gm16046
ENSMUSG00000085767	2.0864	-0.3394	DOWN	Gm13563
ENSMUSG00000085795	3.381	-0.2784	DOWN	Zfp703
ENSMUSG00000085798	1.6251	-0.8454	DOWN	Gm2018
ENSMUSG00000085828	3.4003	-0.3722	DOWN	Gm15612
ENSMUSG00000085830	6.9235	-0.4795	DOWN	Grin1os
ENSMUSG00000085925	8.5408	-0.2916	DOWN	Rtl1
ENSMUSG00000085957	1.337	-0.2707	DOWN	Syna
ENSMUSG00000085962	2.2092	-0.8331	DOWN	Gm16984
ENSMUSG00000086040	2.1806	-0.1727	DOWN	Wipf3
ENSMUSG00000086067	6.8135	-0.8846	DOWN	Gm16183
ENSMUSG00000086119	21.1489	-0.5777	DOWN	Gm2415
ENSMUSG00000086193	15.3383	-0.7091	DOWN	Gm11508
ENSMUSG00000086288	5.5283	-0.7134	DOWN	Gm15265
ENSMUSG00000086446	3.7667	-1.0527	DOWN	Prkag2os1

ENSMUSG00000086507	2.624	-1.1884	DOWN	Adap2os
ENSMUSG00000086536	6.2058	-0.7196	DOWN	Gm12264
ENSMUSG00000086587	1.7028	-0.4593	DOWN	Gm11837
ENSMUSG00000086600	1.8151	-0.3596	DOWN	C030005K06Rik
ENSMUSG00000086614	2.3401	-1.4614	DOWN	Gm14330
ENSMUSG00000086683	1.4954	-1.0508	DOWN	Gm12867
ENSMUSG00000086716	7.5042	-2.0114	DOWN	Gm11629
ENSMUSG00000086774	1.5007	-0.9684	DOWN	Gm11915
ENSMUSG00000086782	2.1238	-0.2737	DOWN	E130102H24Rik
ENSMUSG00000086794	1.4628	-0.3499	DOWN	Gm11642
ENSMUSG00000086826	4.8886	-0.4992	DOWN	Gm11739
ENSMUSG00000086905	15.738	-1.2366	DOWN	Gm13716
ENSMUSG00000086968	2.9827	-0.1469	DOWN	4933431E20Rik
ENSMUSG00000087018	1.401	-0.5383	DOWN	2900072N19Rik
ENSMUSG00000087026	2.6277	-0.3203	DOWN	A230103J11Rik
ENSMUSG00000087038	1.7878	-0.5186	DOWN	2900079G21Rik
ENSMUSG00000087159	2.7047	-0.4163	DOWN	Gm15246
ENSMUSG00000087178	17.0836	-0.5319	DOWN	A230056P14Rik
ENSMUSG00000087268	1.6689	-1.3559	DOWN	Gm14486
ENSMUSG00000087307	2.6186	-0.5065	DOWN	Gm12925
ENSMUSG00000087381	5.298	-0.4447	DOWN	Gm16008
ENSMUSG00000087408	2.7488	-0.1389	DOWN	Cers1
ENSMUSG00000087416	2.3081	-1.2168	DOWN	Gm15906
ENSMUSG00000087535	2.3145	-0.4597	DOWN	Zmiz1os1
ENSMUSG00000087595	1.6049	-0.4163	DOWN	1810012K08Rik
ENSMUSG00000087610	1.3984	-0.416	DOWN	Gm16253
ENSMUSG00000087639	1.5792	-1.6307	DOWN	Gm15512
ENSMUSG00000087644	5.5655	-0.6909	DOWN	Gm14703
ENSMUSG00000087658	7.9023	-1.9538	DOWN	Hotairm1
ENSMUSG00000087672	17.2116	-0.9343	DOWN	Gm15122
ENSMUSG00000087679	6.8328	-0.2911	DOWN	Tmem250-ps
ENSMUSG00000087963	2.669	-1.6196	DOWN	Gm25394
ENSMUSG00000088008	1.75	-0.5998	DOWN	Gm25492
ENSMUSG00000088054	1.4994	-0.7954	DOWN	Mir1968
ENSMUSG00000088139	2.7775	-0.9623	DOWN	Gm27343
ENSMUSG00000088901	1.5948	-1.3316	DOWN	Mir1943
ENSMUSG00000088982	2.9487	-2.2478	DOWN	Gm25600
ENSMUSG00000088984	2.6646	-0.8493	DOWN	Gm25604
ENSMUSG00000089417	6.2555	-1.6118	DOWN	Gm22009
ENSMUSG00000089636	5.4765	-0.9978	DOWN	1700058P15Rik
ENSMUSG00000089682	4.8679	-0.1811	DOWN	Bcl2l2
ENSMUSG00000089715	10.4001	-0.2252	DOWN	Cbx6
ENSMUSG00000089736	3.4328	-0.4109	DOWN	Tgfbr3l
ENSMUSG00000089824	3.1968	-0.222	DOWN	Rbm12
ENSMUSG00000089911	2.3639	-0.1419	DOWN	Mfsd14a

ENSMUSG00000089929	1.8986	-1.4324	DOWN	Bcl2a1b
ENSMUSG00000089968	2.6318	-0.6877	DOWN	Nckap5los
ENSMUSG00000090000	1.3523	-0.1585	DOWN	Ier3ip1
ENSMUSG00000090005	2.6371	-1.7288	DOWN	Gm16540
ENSMUSG00000090115	1.4143	-0.1509	DOWN	Usp49
ENSMUSG00000090200	1.9245	-1.0801	DOWN	1700025N21Rik
ENSMUSG00000090223	11.9743	-0.5319	DOWN	Pcp4
ENSMUSG00000090236	2.3954	-0.5995	DOWN	Car15
ENSMUSG00000090247	2.4011	-0.6837	DOWN	Bloc1s1
ENSMUSG00000090327	2.7117	-0.7788	DOWN	Gm17111
ENSMUSG00000090330	3.2084	-0.444	DOWN	9130221H12Rik
ENSMUSG00000090589	4.2092	-0.9963	DOWN	Gm17180
ENSMUSG00000090625	3.8707	-1.9458	DOWN	Gm20721
ENSMUSG00000090761	4.4809	-0.4086	DOWN	Gm17201
ENSMUSG00000090778	4.7411	-0.6322	DOWN	Gm3235
ENSMUSG00000090824	4.1435	-2.9124	DOWN	Olfr1344
ENSMUSG00000091021	4.3265	-1.3445	DOWN	Gm17300
ENSMUSG00000091185	1.383	-1.6848	DOWN	Gm3278
ENSMUSG00000091223	5.5565	-1.0868	DOWN	Gm8775
ENSMUSG00000091264	3.4963	-0.1976	DOWN	Smim13
ENSMUSG00000091272	3.8607	-1.1709	DOWN	Gm17641
ENSMUSG00000091390	3.6352	-0.8157	DOWN	Gm17168
ENSMUSG00000091443	2.8964	-0.7655	DOWN	Gm17023
ENSMUSG00000091514	2.1982	-0.4676	DOWN	Gm17484
ENSMUSG00000091803	46.9284	-1.5393	DOWN	Cox16
ENSMUSG00000091811	3.9503	-0.1995	DOWN	Inafm1
ENSMUSG00000091864	2.303	-1.3181	DOWN	Gm17102
ENSMUSG00000091896	4.5736	-0.1793	DOWN	Ube2d2a
ENSMUSG00000092086	4.7073	-1.2802	DOWN	Gm6793
ENSMUSG00000092187	4.3627	-0.545	DOWN	Gm20457
ENSMUSG00000092210	1.5387	-0.4828	DOWN	A930009A15Rik
ENSMUSG00000092216	1.501	-0.4909	DOWN	Gm19345
ENSMUSG00000092229	3.1879	-0.5376	DOWN	Gm5977
ENSMUSG00000092274	2.8683	-0.2523	DOWN	Neat1
ENSMUSG00000092368	5.0279	-0.6148	DOWN	A930015D03Rik
ENSMUSG00000092375	6.6725	-0.743	DOWN	A730060N03Rik
ENSMUSG00000092509	2.198	-0.3578	DOWN	Gm20394
ENSMUSG00000092593	2.0426	-0.5905	DOWN	Gm20492
ENSMUSG00000092595	2.1946	-1.6608	DOWN	Gm20427
ENSMUSG00000092622	1.486	-0.97	DOWN	Khdc3
ENSMUSG00000092626	3.0488	-0.9096	DOWN	9130230N09Rik
ENSMUSG00000092659	5.1051	-3.1151	DOWN	Mir3100
ENSMUSG00000092827	2.9375	-0.9661	DOWN	Mir3091
ENSMUSG00000093147	1.3705	-1.889	DOWN	Gm23369
ENSMUSG00000093351	3.7336	-0.8583	DOWN	Mir3072

ENSMUSG00000093424	2.5749	-0.8009	DOWN	6330562C20Rik
ENSMUSG00000093445	1.406	-0.8689	DOWN	Lrch4
ENSMUSG00000093452	3.8263	-0.8363	DOWN	Zfhx2os
ENSMUSG00000093502	1.9296	-0.6506	DOWN	Gm20700
ENSMUSG00000093629	6.3833	-0.6359	DOWN	Prox2os
ENSMUSG00000093637	2.6456	-0.5541	DOWN	Gm20636
ENSMUSG00000093686	2.6162	-1.2934	DOWN	Gm4705
ENSMUSG00000093930	7.1319	-0.1899	DOWN	Hmgcs1
ENSMUSG00000093954	3.387	-1.8674	DOWN	Gm16867
ENSMUSG00000093989	3.9024	-0.8448	DOWN	Rnasek
ENSMUSG00000094076	1.4961	-2.0289	DOWN	Gm4767
ENSMUSG00000094083	1.4703	-1.7103	DOWN	Gm1604b
ENSMUSG00000094910	2.2821	-0.1921	DOWN	D430019H16Rik
ENSMUSG00000094958	1.3562	-0.5843	DOWN	3110021N24Rik
ENSMUSG00000094989	1.432	-0.2911	DOWN	Rpl9-ps4
ENSMUSG00000095115	1.6835	-0.2872	DOWN	Itpripl2
ENSMUSG00000095139	1.5216	-0.179	DOWN	Pou3f2
ENSMUSG00000095334	38.3374	-1.4701	DOWN	Gm21984
ENSMUSG00000095440	1.9346	-0.2743	DOWN	Fignl2
ENSMUSG00000095478	3.9281	-0.9982	DOWN	Gm9824
ENSMUSG00000095597	1.7932	-0.2203	DOWN	Rps7-ps3
ENSMUSG00000096145	5.006	-0.6992	DOWN	Vkorc1
ENSMUSG00000096257	1.9495	-0.5046	DOWN	Ccer2
ENSMUSG00000096351	1.6131	-0.2215	DOWN	Samd11
ENSMUSG00000096847	1.7187	-0.1896	DOWN	Tmem151b
ENSMUSG00000096966	1.4823	-0.4919	DOWN	Gm18336
ENSMUSG00000097049	1.464	-0.5974	DOWN	6530411M01Rik
ENSMUSG00000097077	1.7065	-1.5563	DOWN	Gm16712
ENSMUSG00000097101	2.1119	-0.451	DOWN	1810034E14Rik
ENSMUSG00000097167	1.5925	-0.4398	DOWN	Gm16740
ENSMUSG00000097204	4.2024	-0.608	DOWN	Gm17690
ENSMUSG00000097280	7.4804	-0.6215	DOWN	AI849053
ENSMUSG00000097320	10.7862	-0.6954	DOWN	Tmem147os
ENSMUSG00000097325	2.2105	-0.646	DOWN	Gm16897
ENSMUSG00000097343	1.9561	-0.2569	DOWN	9030407P20Rik
ENSMUSG00000097404	1.5292	-0.5576	DOWN	Gm10814
ENSMUSG00000097405	2.6529	-0.6031	DOWN	D630044L22Rik
ENSMUSG00000097414	6.2038	-0.8525	DOWN	B130046B21Rik
ENSMUSG00000097431	2.8094	-0.2387	DOWN	Gm26782
ENSMUSG00000097433	2.236	-0.4688	DOWN	Gm26781
ENSMUSG00000097504	2.7589	-0.2093	DOWN	4930516B21Rik
ENSMUSG00000097535	1.7234	-0.2222	DOWN	Gm26592
ENSMUSG00000097637	1.726	-0.7009	DOWN	4933417D19Rik
ENSMUSG00000097692	1.3522	-0.6958	DOWN	A230060F14Rik
ENSMUSG00000097767	6.4828	-0.251	DOWN	Miat

ENSMUSG00000097785	5.3761	-0.3906	DOWN	B230217O12Rik
ENSMUSG00000097788	5.4574	-1.3184	DOWN	Gm16596
ENSMUSG00000097885	1.4333	-0.1655	DOWN	5031434O11Rik
ENSMUSG00000097915	2.4081	-0.5985	DOWN	A330009N23Rik
ENSMUSG00000097929	8.1551	-0.2491	DOWN	Tunar
ENSMUSG00000098004	1.7058	-1.2289	DOWN	Gm27027
ENSMUSG00000098143	1.6039	-0.7857	DOWN	Gm26937
ENSMUSG00000098172	2.3804	-0.4479	DOWN	Gm26973
ENSMUSG00000098196	7.19	-0.8564	DOWN	Gm26964
ENSMUSG00000098259	8.6502	-1.2034	DOWN	Gm27616
ENSMUSG00000098269	2.6361	-1.2439	DOWN	Mir8094
ENSMUSG00000098274	1.4143	-0.5678	DOWN	Rpl24
ENSMUSG00000098332	2.7503	-0.3881	DOWN	Pigbos1
ENSMUSG00000098338	3.086	-1.2272	DOWN	Mir6941
ENSMUSG00000098376	1.9477	-1.9856	DOWN	Mir6907
ENSMUSG00000098388	1.7667	-2.3811	DOWN	Mir8109
ENSMUSG00000098620	3.9663	-0.5596	DOWN	Gm27209
ENSMUSG00000098661	12.0066	-1.0791	DOWN	Mir7052
ENSMUSG00000098893	3.8682	-1.7214	DOWN	Gm17828
ENSMUSG00000098912	1.8307	-0.1526	DOWN	1500004A13Rik
ENSMUSG00000099033	1.9046	-1.2198	DOWN	Mir7013
ENSMUSG00000099196	1.8937	-1.2201	DOWN	Mir1258
ENSMUSG00000099284	4.0407	-1.0958	DOWN	Mir7026
ENSMUSG00000099343	1.347	-1.6556	DOWN	Gm28836
ENSMUSG00000099384	3.7823	-0.6406	DOWN	1700110C19Rik
ENSMUSG00000099478	6.4569	-0.3046	DOWN	Gm28370
ENSMUSG00000099681	2.8929	-0.3558	DOWN	1700052K11Rik
ENSMUSG00000099696	2.4551	-0.3064	DOWN	2900052N01Rik
ENSMUSG00000099764	9.8798	-0.3678	DOWN	Rps10-ps2
ENSMUSG00000099874	1.6085	-1.4726	DOWN	Gm29629
ENSMUSG00000100514	1.4835	-0.6286	DOWN	Gm12960
ENSMUSG00000100552	4.3542	-1.1453	DOWN	Gm29019
ENSMUSG00000100622	1.7229	-1.1152	DOWN	Gm20379
ENSMUSG00000100954	2.6057	-0.5122	DOWN	Gm10138
ENSMUSG00000101320	1.9155	-0.9171	DOWN	Gm28529
ENSMUSG00000101609	9.1596	-0.3569	DOWN	Kcnq1ot1
ENSMUSG00000102234	3.9553	-1.3882	DOWN	Gm37885
ENSMUSG00000102252	33.093	-0.525	DOWN	Snrpn
ENSMUSG00000102416	3.8996	-0.4939	DOWN	4933424G06Rik
ENSMUSG00000102428	5.9929	-0.3569	DOWN	Pcdhga12
ENSMUSG00000102573	1.7758	-1.8437	DOWN	Gm7265
ENSMUSG00000102869	51.8487	-0.3491	DOWN	Norad
ENSMUSG00000102918	4.2004	-0.2975	DOWN	Pcdhgc3
ENSMUSG00000103436	5.2926	-1.0444	DOWN	Gm36995
ENSMUSG00000103649	1.4002	-0.2855	DOWN	Gm37768

ENSMUSG00000103713	1.9317	-0.2788	DOWN	Gm2136
ENSMUSG00000103761	5.9191	-0.5545	DOWN	Gm37859
ENSMUSG00000104002	1.3687	-0.3659	DOWN	Gm38336
ENSMUSG00000104063	1.8173	-0.2541	DOWN	Pcdhgb7
ENSMUSG00000104156	6.0258	-0.3487	DOWN	Gm38102
ENSMUSG00000104252	3.0922	-3.0392	DOWN	Pcdha4
ENSMUSG00000104283	4.8016	-0.4818	DOWN	Gm37459
ENSMUSG00000104293	3.1121	-1.1432	DOWN	Gm38043
ENSMUSG00000104361	2.5539	-0.7095	DOWN	Gm38080
ENSMUSG00000104369	2.6626	-1.4806	DOWN	Gm38082
ENSMUSG00000104399	2.2617	-0.2921	DOWN	Gm37963
ENSMUSG00000104548	1.5173	-0.3413	DOWN	Gm43857
ENSMUSG00000104885	2.103	-0.9197	DOWN	Gm43758
ENSMUSG00000105161	1.4549	-0.3	DOWN	Gm42595
ENSMUSG00000105224	1.9776	-1.0406	DOWN	Gm3364
ENSMUSG00000105245	2.0942	-0.3379	DOWN	Gm31305
ENSMUSG00000105691	1.4694	-0.8733	DOWN	Gm42876
ENSMUSG00000105843	2.6574	-0.4087	DOWN	Gm19439
ENSMUSG00000105852	2.3528	-0.5175	DOWN	Gm42890
ENSMUSG00000105867	1.3191	-0.1379	DOWN	Gm42517
ENSMUSG00000105881	2.4867	-0.4501	DOWN	4932422M17Rik
ENSMUSG00000105948	2.8275	-1.1053	DOWN	Gm43089
ENSMUSG00000105969	6.2284	-1.2471	DOWN	Gm42525
ENSMUSG00000106028	2.9368	-0.537	DOWN	Gm8493
ENSMUSG00000106275	1.6378	-0.5476	DOWN	Gm42495
ENSMUSG00000106396	1.4296	-0.4926	DOWN	Gm42902
ENSMUSG00000106463	2.0382	-1.0125	DOWN	Mir1291
ENSMUSG00000106464	2.1645	-0.4629	DOWN	C130083M11Rik
ENSMUSG00000106688	1.6039	-0.5378	DOWN	Gm42851
ENSMUSG00000106967	3.0883	-1.0097	DOWN	Gm42477
ENSMUSG00000107068	42.4325	-2.4513	DOWN	Gm42742
ENSMUSG00000107086	4.1861	-0.4877	DOWN	Gm43808
ENSMUSG00000107295	2.8781	-1.4765	DOWN	Gm42896
ENSMUSG00000107331	9.7106	-0.3917	DOWN	Gm42732
ENSMUSG00000107340	1.3802	-1.1403	DOWN	Gm42789
ENSMUSG00000107585	3.3204	-0.2826	DOWN	3300002P13Rik
ENSMUSG00000107859	2.0812	-0.4304	DOWN	Gm30731
ENSMUSG00000107927	6.7158	-1.2316	DOWN	Gm44090
ENSMUSG00000108057	1.3089	-0.4848	DOWN	Gm44234
ENSMUSG00000108070	3.5798	-1.1577	DOWN	Gm43872
ENSMUSG00000108126	3.7356	-0.7738	DOWN	Gm43909
ENSMUSG00000108148	3.2541	-1.3413	DOWN	Gm44020
ENSMUSG00000108320	6.7395	-1.4706	DOWN	Gm44877
ENSMUSG00000108322	1.6698	-0.5914	DOWN	5430431A17Rik
ENSMUSG00000108350	2.582	-0.2295	DOWN	Gm44950

ENSMUSG00000108358	1.4736	-0.1427	DOWN	Gm44509
ENSMUSG00000108402	1.719	-0.5506	DOWN	9430064I24Rik
ENSMUSG00000108452	41.3312	-1.6555	DOWN	4930413G21Rik
ENSMUSG00000108456	1.4374	-0.2438	DOWN	4732496C06Rik
ENSMUSG00000108486	1.5702	-0.7255	DOWN	Gm44836
ENSMUSG00000108543	1.8119	-0.8416	DOWN	Gm44735
ENSMUSG00000108584	1.6651	-0.9366	DOWN	Gm45216
ENSMUSG00000108678	2.9924	-0.6901	DOWN	Gm44758
ENSMUSG00000108736	2.2711	-0.4223	DOWN	Gm45151
ENSMUSG00000108920	2.6106	-0.599	DOWN	Gm44678
ENSMUSG00000109051	1.5216	-0.7485	DOWN	Gm44913
ENSMUSG00000109147	3.564	-0.4869	DOWN	4930431P19Rik
ENSMUSG00000109165	3.2581	-0.8766	DOWN	Gm45148
ENSMUSG00000109198	1.3343	-0.6739	DOWN	D7Bwg0826e
ENSMUSG00000109274	15.3359	-0.6902	DOWN	Gm45133
ENSMUSG00000109324	3.6131	-0.1758	DOWN	Prmt1
ENSMUSG00000109362	16.3316	-0.5726	DOWN	Gm44562
ENSMUSG00000109505	1.4118	-0.3155	DOWN	Gm44677
ENSMUSG00000109695	1.5886	-0.3076	DOWN	Gm31166
ENSMUSG00000109715	1.5342	-0.3121	DOWN	Gm45606
ENSMUSG00000109753	1.4947	-1.7987	DOWN	Gm45633
ENSMUSG00000109783	1.3982	-0.7778	DOWN	Gm45338
ENSMUSG00000109998	1.8704	-0.8569	DOWN	Gm45437
ENSMUSG00000110070	6.7572	-0.8578	DOWN	Gm45619
ENSMUSG00000110230	2.2747	-2.4618	DOWN	Gm45846
ENSMUSG00000110357	2.5189	-0.2762	DOWN	A030001D20Rik
ENSMUSG00000110580	4.6893	-0.7481	DOWN	D830024N08Rik
ENSMUSG00000110698	1.3646	-1.0455	DOWN	Gm45875
ENSMUSG00000110824	1.7672	-0.5638	DOWN	Gm48677
ENSMUSG00000110827	1.5473	-0.6901	DOWN	Gm32281
ENSMUSG00000111063	1.983	-0.2517	DOWN	Zfp660
ENSMUSG00000111068	1.7645	-1.0644	DOWN	Gm35501
ENSMUSG00000111128	7.6885	-0.3815	DOWN	Gm49338
ENSMUSG00000111143	2.1952	-1.3013	DOWN	Gm47134
ENSMUSG00000111291	2.825	-0.2529	DOWN	Gm48604
ENSMUSG00000111497	1.9561	-1.0078	DOWN	Gm38431
ENSMUSG00000111629	2.7253	-1.5558	DOWN	4930442G15Rik
ENSMUSG00000112064	1.8028	-0.7735	DOWN	Gm47399
ENSMUSG00000112117	7.5007	-0.3655	DOWN	Rmst
ENSMUSG00000112201	4.2535	-0.9313	DOWN	4930532I03Rik
ENSMUSG00000112288	1.8862	-1.0042	DOWN	Gm47879
ENSMUSG00000112941	22.442	-1.3224	DOWN	Gm48623
ENSMUSG00000113028	1.5499	-0.7798	DOWN	Gm18065
ENSMUSG00000113029	2.1824	-0.3435	DOWN	Gm40578
ENSMUSG00000113052	1.6797	-0.5066	DOWN	Gm31513

ENSMUSG00000113070	1.3455	-0.6693	DOWN	Gm48420
ENSMUSG00000113137	3.7108	-1.6548	DOWN	Gm7511
ENSMUSG00000113450	1.4142	-0.3817	DOWN	Zfp935
ENSMUSG00000113591	1.3967	-1.2114	DOWN	4930447K03Rik
ENSMUSG00000114014	3.8903	-0.7864	DOWN	Gm48350
ENSMUSG00000114019	4.0566	-0.2971	DOWN	Gm47155
ENSMUSG00000114244	1.4215	-0.7333	DOWN	Gm47119
ENSMUSG00000114267	3.3125	-0.9476	DOWN	Gm48600
ENSMUSG00000114277	2.1807	-0.5664	DOWN	Gm48583
ENSMUSG00000114362	1.7798	-0.5148	DOWN	4933433G19Rik
ENSMUSG00000114493	1.6439	-0.3357	DOWN	Gm47071
ENSMUSG00000114707	2.8575	-0.9026	DOWN	Gm47558
ENSMUSG00000114708	7.3055	-0.8937	DOWN	Gm30177
ENSMUSG00000114796	1.8875	-0.9078	DOWN	A930028N01Rik
ENSMUSG00000114835	2.2085	-0.7349	DOWN	Gm48194
ENSMUSG00000114883	1.4904	-2.0699	DOWN	Gm48860
ENSMUSG00000115005	1.68	-1.2129	DOWN	Gm49195
ENSMUSG00000115138	3.5569	-0.4441	DOWN	Gm36899
ENSMUSG00000115338	1.4605	-0.2157	DOWN	Pnp
ENSMUSG00000115410	1.3344	-1.3636	DOWN	2810457G06Rik
ENSMUSG00000115431	1.4108	-0.6677	DOWN	Gm3219
ENSMUSG00000115518	3.1354	-0.3961	DOWN	Gm10791
ENSMUSG00000115958	2.3551	-2.1496	DOWN	Gm28040
ENSMUSG00000115976	3.0966	-0.8616	DOWN	Gm31462
ENSMUSG00000116038	1.7402	-0.6232	DOWN	Gm46563
ENSMUSG00000116234	1.8102	-0.5812	DOWN	Gm49550
ENSMUSG00000116250	7.5032	-1.1731	DOWN	Gm34939
ENSMUSG00000116564	2.1063	-0.1391	DOWN	Riok2
ENSMUSG00000116594	2.217	-0.28	DOWN	Gm49601
ENSMUSG00000116690	2.4182	-2.3815	DOWN	Gm49736
ENSMUSG00000116927	2.2583	-0.9275	DOWN	Gm30881
ENSMUSG00000116988	2.6529	-1.9634	DOWN	Gm49673
ENSMUSG00000117069	4.4224	-1.179	DOWN	Gm49894
ENSMUSG00000117128	3.1299	-1.813	DOWN	Gm49915
ENSMUSG00000117285	3.7663	-0.4765	DOWN	Gm49863
ENSMUSG00000117289	5.4201	-1.4333	DOWN	1700093J21Rik
ENSMUSG00000117310	15.2271	-1.3104	DOWN	Ptp4a1
ENSMUSG00000117375	3.7966	-0.9981	DOWN	Gm33373
ENSMUSG00000117438	1.5219	-0.2853	DOWN	Rpsa-ps7
ENSMUSG00000117710	3.47	-0.6624	DOWN	Gm10817
ENSMUSG00000117740	1.8821	-1.8212	DOWN	Gm4242
ENSMUSG00000117789	1.3773	-1.7255	DOWN	Gm50388
ENSMUSG00000117814	3.1338	-0.2854	DOWN	Gm50431
ENSMUSG00000117819	8.1219	-1.5136	DOWN	Gm50253
ENSMUSG00000117901	3.9023	-0.3399	DOWN	ENSMUSG00000117901

ENSMUSG00000117942	5.9709	-0.8657	DOWN	Maskbp3
ENSMUSG00000118012	4.8026	-0.2787	DOWN	Gm46620
ENSMUSG00000118084	1.5312	-0.3963	DOWN	AA388235
ENSMUSG00000118125	2.025	-0.3875	DOWN	Gm50387
ENSMUSG00000118202	1.3407	-0.1612	DOWN	C030017B01Rik
ENSMUSG00000118501	3.6746	-0.9256	DOWN	Gm53048
ENSMUSG00000118502	6.2772	-0.7344	DOWN	Gm52964
ENSMUSG00000118504	6.9632	-1.0031	DOWN	Gm53012
ENSMUSG00000118526	1.968	-1.2373	DOWN	Gm52960
ENSMUSG00000118661	2.4051	-1.0719	DOWN	Muc6
ENSMUSG00000118729	4.1571	-1.0327	DOWN	Snord88c
ENSMUSG00000119223	3.4763	-0.8965	DOWN	Mir3070b
ENSMUSG00000119229	3.8978	-1.1009	DOWN	Mir3070a
ENSMUSG00000119442	3.5896	-1.2633	DOWN	Gm23966
ENSMUSG00000119765	1.7822	-0.7365	DOWN	Gm23136
ENSMUSG00000119926	3.8682	-0.8011	DOWN	Gm25047
ENSMUSG00000119980	1.656	-0.6858	DOWN	ENSMUSG00000119980
ENSMUSG00000120116	1.4889	-0.319	DOWN	ENSMUSG00000120116
ENSMUSG00000120144	4.1851	-0.282	DOWN	ENSMUSG00000120144
ENSMUSG00000120198	3.0933	-1.4286	DOWN	ENSMUSG00000120198
ENSMUSG00000120237	2.7735	-0.2402	DOWN	ENSMUSG00000120237
ENSMUSG00000120261	1.8194	-1.3042	DOWN	ENSMUSG00000120261
ENSMUSG00000120483	7.3384	-0.5948	DOWN	ENSMUSG00000120483
ENSMUSG00000120615	3.2885	-1.4257	DOWN	ENSMUSG00000120615
ENSMUSG00000120617	2.7659	-2.8241	DOWN	ENSMUSG00000120617
ENSMUSG00000120681	8.4069	-0.6611	DOWN	ENSMUSG00000120681
ENSMUSG00000120698	3.0759	-1.1147	DOWN	ENSMUSG00000120698
ENSMUSG00000120732	3.0382	-0.2275	DOWN	ENSMUSG00000120732
ENSMUSG00000120742	1.3597	-0.36	DOWN	ENSMUSG00000120742
ENSMUSG00000120806	14.6304	-1.1595	DOWN	ENSMUSG00000120806
ENSMUSG00000120819	3.6054	-1.2679	DOWN	ENSMUSG00000120819
ENSMUSG00000120847	4.3546	-0.7422	DOWN	ENSMUSG00000120847
ENSMUSG00000120857	1.8792	-1.6349	DOWN	ENSMUSG00000120857
ENSMUSG00000120874	1.6344	-1.1725	DOWN	ENSMUSG00000120874
ENSMUSG00000121001	1.7776	-0.3836	DOWN	ENSMUSG00000121001
ENSMUSG00000121052	1.4108	-1.7246	DOWN	ENSMUSG00000121052
ENSMUSG00000121061	5.9166	-1.9931	DOWN	ENSMUSG00000121061
ENSMUSG00000121080	4.4429	-0.5395	DOWN	ENSMUSG00000121080
ENSMUSG00000121097	1.3555	-1.2119	DOWN	ENSMUSG00000121097
ENSMUSG00000121121	1.5216	-0.5873	DOWN	ENSMUSG00000121121
ENSMUSG00000121141	19.3727	-0.4367	DOWN	ENSMUSG00000121141
ENSMUSG00000121178	2.0933	-1.0324	DOWN	ENSMUSG00000121178
ENSMUSG00000121226	2.6786	-0.3629	DOWN	ENSMUSG00000121226
ENSMUSG00000121256	3.8977	-1.1465	DOWN	ENSMUSG00000121256
ENSMUSG00000121274	2.8249	-1.1677	DOWN	ENSMUSG00000121274

ENSMUSG00002074925	2.0737	-0.8438	DOWN	5S_rRNA
ENSMUSG00002074982	2.0325	-1.1029	DOWN	U6
ENSMUSG00002075017	19.9038	-1.0762	DOWN	ENSMUSG00002075017
ENSMUSG00002075075	20.0589	-1.5969	DOWN	ENSMUSG00002075075
ENSMUSG00002075108	1.8514	-1.4228	DOWN	ENSMUSG00002075108
ENSMUSG00002075149	1.9479	-0.7976	DOWN	5S_rRNA
ENSMUSG00002075231	5.2209	-1.2858	DOWN	ENSMUSG00002075231
ENSMUSG00002075232	9.7556	-2.2695	DOWN	ENSMUSG00002075232
ENSMUSG00002075713	1.5668	-1.3535	DOWN	ENSMUSG00002075713
ENSMUSG00002076055	1.6816	-1.8838	DOWN	SNORA63
ENSMUSG00002076103	1.5312	-0.5595	DOWN	ENSMUSG00002076103
ENSMUSG00002076304	4.0021	-1.2206	DOWN	7SK
ENSMUSG00002076443	3.6654	-2.5592	DOWN	ENSMUSG00002076443
ENSMUSG00002076614	5.8245	-1.4257	DOWN	ENSMUSG00002076614
ENSMUSG00000000340	6.4386	0.2537	UP	Dbt
ENSMUSG00000000560	1.4712	0.192	UP	Gabra2
ENSMUSG00000000563	3.1138	0.1801	UP	Atp5pb
ENSMUSG00000000740	2.1481	0.1584	UP	Rpl13
ENSMUSG00000000876	1.3395	0.1805	UP	Pxmp4
ENSMUSG00000000959	4.3985	0.1653	UP	Oxa1l
ENSMUSG00000001018	5.1937	0.2263	UP	Snapin
ENSMUSG00000001039	1.4531	0.1585	UP	B9d1
ENSMUSG00000001082	5.9191	0.248	UP	Mfsd10
ENSMUSG00000001105	7.9681	0.3141	UP	Ift20
ENSMUSG00000001260	3.7105	0.1607	UP	Gabrg1
ENSMUSG00000001435	1.441	0.1506	UP	Col18a1
ENSMUSG00000001507	2.8768	0.1477	UP	Itga3
ENSMUSG00000001663	2.2296	0.1952	UP	Gstt1
ENSMUSG00000001794	9.9157	0.2547	UP	Capns1
ENSMUSG00000001964	2.1404	0.1421	UP	Emd
ENSMUSG00000001999	1.3488	0.1837	UP	Blvra
ENSMUSG00000002010	3.0376	0.2459	UP	Idh3g
ENSMUSG00000002014	10.0616	0.3512	UP	Ssr4
ENSMUSG00000002028	3.4294	0.1673	UP	Kmt2a
ENSMUSG00000002055	2.1872	0.1826	UP	Spag5
ENSMUSG00000002332	2.6154	0.1559	UP	Dhrs1
ENSMUSG00000002475	2.407	0.1389	UP	Abhd3
ENSMUSG00000002617	1.8781	0.1831	UP	Zfp40
ENSMUSG00000002660	1.6948	0.1665	UP	Clpp
ENSMUSG00000002661	2.467	0.2448	UP	Alkbh7
ENSMUSG00000002728	5.5306	0.2487	UP	Naa20
ENSMUSG00000002799	1.762	0.1426	UP	Jag2
ENSMUSG00000002844	3.0207	0.1614	UP	Adprh
ENSMUSG00000003099	8.0439	0.1918	UP	Ppp5c
ENSMUSG00000003161	5.8416	0.2091	UP	Sri

ENSMUSG00000003269	4.6321	0.2911	UP	Cyth2
ENSMUSG00000003299	2.5851	0.1707	UP	Mrpl4
ENSMUSG00000003380	4.4786	0.2266	UP	Rabac1
ENSMUSG00000003528	4.7415	0.195	UP	Slc25a1
ENSMUSG00000003808	2.3448	0.1436	UP	Farsa
ENSMUSG00000003873	3.2451	0.308	UP	Bax
ENSMUSG00000003955	2.3011	0.2093	UP	Fam162a
ENSMUSG00000003970	2.6676	0.1651	UP	Rpl8
ENSMUSG00000004098	2.3147	0.2583	UP	Col5a3
ENSMUSG00000004285	2.6028	0.2112	UP	Atp6v1f
ENSMUSG00000004637	3.3287	0.1948	UP	Wwox
ENSMUSG00000004667	2.013	0.1519	UP	Polr2e
ENSMUSG00000004934	3.2157	0.1817	UP	Pias4
ENSMUSG00000005054	2.4279	0.2337	UP	Cstb
ENSMUSG00000005102	2.7033	0.173	UP	Eif2ak4
ENSMUSG00000005268	1.6503	0.1512	UP	Prlr
ENSMUSG00000005575	2.7648	0.1865	UP	Ube2m
ENSMUSG00000005687	5.3422	0.1974	UP	Bcas2
ENSMUSG00000005881	3.1478	0.17	UP	Ergic3
ENSMUSG00000006095	3.2738	0.3521	UP	Tbcb
ENSMUSG00000006304	4.5748	0.2101	UP	Arpc2
ENSMUSG00000006315	2.8182	0.1936	UP	Tmem147
ENSMUSG00000006333	1.8058	0.1567	UP	Rps9
ENSMUSG00000006342	3.3968	0.1776	UP	Susd2
ENSMUSG00000006360	2.6496	0.4347	UP	Crip1
ENSMUSG00000006386	2.7218	0.2119	UP	Tek
ENSMUSG00000006442	2.3251	0.1625	UP	Srm
ENSMUSG00000006519	2.0289	0.286	UP	Cyba
ENSMUSG00000006732	2.0059	0.1917	UP	Mettl1
ENSMUSG00000006931	3.4958	0.2041	UP	P3h4
ENSMUSG00000006941	10.0336	0.3343	UP	Eif1b
ENSMUSG00000007021	2.063	0.1409	UP	Syngr3
ENSMUSG00000007080	1.9606	0.4486	UP	Pole
ENSMUSG00000007338	5.2619	0.2813	UP	Mrpl49
ENSMUSG00000007827	1.329	0.1876	UP	Ankrd26
ENSMUSG00000007987	5.2663	0.2223	UP	Ift22
ENSMUSG00000008140	3.97	0.1787	UP	Emc10
ENSMUSG00000008200	3.3963	0.2042	UP	Fnbp4
ENSMUSG00000008301	3.3643	0.1678	UP	Phax
ENSMUSG00000008318	4.5397	0.194	UP	Relt
ENSMUSG00000008348	13.5833	0.4833	UP	Ubc
ENSMUSG00000008668	5.5785	0.2755	UP	Rps18
ENSMUSG00000008682	3.2692	0.1776	UP	Rpl10
ENSMUSG00000008892	4.1853	0.2229	UP	Vdac3
ENSMUSG00000009013	2.5246	0.2394	UP	Dynll1

ENSMUSG00000009418	3.2069	0.1631	UP	Nav1
ENSMUSG00000009863	1.6948	0.1502	UP	Sdhb
ENSMUSG00000009927	1.7458	0.1602	UP	Rps25
ENSMUSG00000010045	2.7749	0.1768	UP	Tmem115
ENSMUSG00000010057	2.2632	0.1468	UP	Nprl2
ENSMUSG00000010136	1.3097	0.3889	UP	Pifo
ENSMUSG00000010376	3.1445	0.2209	UP	Nedd8
ENSMUSG00000010406	1.354	0.1864	UP	Mrpl52
ENSMUSG00000011154	1.5387	0.2822	UP	Cfap161
ENSMUSG00000011589	2.049	0.1659	UP	Fsd1
ENSMUSG00000012848	3.564	0.1994	UP	Rps5
ENSMUSG00000013155	1.9281	0.267	UP	Enkd1
ENSMUSG00000013593	3.1445	0.1432	UP	Ndufs2
ENSMUSG00000013997	2.6746	0.1738	UP	Nit1
ENSMUSG00000014402	3.0927	0.1763	UP	Tsg101
ENSMUSG00000014606	3.0212	0.166	UP	Slc25a11
ENSMUSG00000014633	2.3732	0.2387	UP	Cmc2
ENSMUSG00000014769	2.842	0.1633	UP	Psmb1
ENSMUSG00000014786	2.1634	0.1644	UP	Slc9a5
ENSMUSG00000014846	1.5247	0.153	UP	Tppp3
ENSMUSG00000014850	3.0492	0.2103	UP	Msh3
ENSMUSG00000014980	1.6577	0.1571	UP	Tsen15
ENSMUSG00000015013	4.195	0.2952	UP	Trappc2l
ENSMUSG00000015092	2.875	0.1997	UP	Edf1
ENSMUSG00000015094	3.4843	0.1478	UP	Npdc1
ENSMUSG00000015149	4.4429	0.1542	UP	Sirt2
ENSMUSG00000015790	4.3449	0.2455	UP	Surf1
ENSMUSG00000015943	2.9581	0.2655	UP	Bola1
ENSMUSG00000016194	3.7018	0.267	UP	Hsd11b1
ENSMUSG00000016252	2.1024	0.2017	UP	Atp5e
ENSMUSG00000016427	2.6445	0.2857	UP	Ndufa1
ENSMUSG00000016526	2.198	0.2396	UP	Dyrk3
ENSMUSG00000017057	1.9354	0.1905	UP	Il13ra1
ENSMUSG00000017747	2.3145	0.187	UP	Ghdc
ENSMUSG00000017760	6.9919	0.1686	UP	Ctsa
ENSMUSG00000017778	3.1736	0.2411	UP	Cox7c
ENSMUSG00000018286	1.5726	0.146	UP	Psmb6
ENSMUSG00000018581	1.3336	0.2877	UP	Dnah11
ENSMUSG00000018593	8.8549	0.1721	UP	Sparc
ENSMUSG00000018672	1.513	0.3658	UP	Copz2
ENSMUSG00000018809	3.0692	0.2486	UP	Smyd4
ENSMUSG00000018882	1.8781	0.1682	UP	Mrpl45
ENSMUSG00000019139	1.7631	0.1742	UP	Isyna1
ENSMUSG00000019158	14.6283	0.4908	UP	Tmem160
ENSMUSG00000019194	3.955	0.2009	UP	Scn1b

ENSMUSG00000019470	2.9924	0.1522	UP	Xab2
ENSMUSG00000019494	2.4738	0.1729	UP	Cops6
ENSMUSG00000019505	12.0769	0.3413	UP	Ubb
ENSMUSG00000019738	3.1592	0.2655	UP	Polr2i
ENSMUSG00000019795	3.9784	0.1585	UP	Pcmt1
ENSMUSG00000019841	5.1689	0.1821	UP	Rev3l
ENSMUSG00000019876	1.4835	0.1863	UP	Pkib
ENSMUSG00000019951	4.7981	0.1714	UP	Uhrf1bp1l
ENSMUSG00000020014	1.4121	0.1901	UP	Cfap54
ENSMUSG00000020024	1.7402	0.1461	UP	Cep83
ENSMUSG00000020105	2.7625	0.3404	UP	Lrig3
ENSMUSG00000020149	4.7317	0.1687	UP	Rab1a
ENSMUSG00000020150	2.669	0.2657	UP	Gamt
ENSMUSG00000020153	8.2742	0.359	UP	Ndufs7
ENSMUSG00000020163	2.1469	0.2408	UP	Uqcr11
ENSMUSG00000020225	3.8475	0.2113	UP	Tmbim4
ENSMUSG00000020267	6.7119	0.3435	UP	Hint1
ENSMUSG00000020308	1.8321	0.1644	UP	Tpgs1
ENSMUSG00000020309	1.9309	0.2128	UP	Chac2
ENSMUSG00000020321	3.9743	0.2019	UP	Mdh1
ENSMUSG00000020362	2.6391	0.152	UP	Cnot6
ENSMUSG00000020386	3.8903	0.2313	UP	Sar1b
ENSMUSG00000020389	1.4584	0.1537	UP	Cdkl3
ENSMUSG00000020392	1.376	0.1648	UP	Cdkn2aipnl
ENSMUSG00000020415	1.5603	0.1497	UP	Pttg1
ENSMUSG00000020441	3.7134	0.2429	UP	2310033P09Rik
ENSMUSG00000020444	3.609	0.199	UP	Guk1
ENSMUSG00000020464	4.1539	0.1655	UP	Pnpt1
ENSMUSG00000020471	5.919	0.2544	UP	Pold2
ENSMUSG00000020477	1.9345	0.1965	UP	Mrps24
ENSMUSG00000020485	2.1674	0.2103	UP	Supt4a
ENSMUSG00000020530	8.36	0.1984	UP	Ggnbp2
ENSMUSG00000020561	1.7427	0.1895	UP	Polr1f
ENSMUSG00000020648	1.3082	0.2113	UP	Dus4l
ENSMUSG00000020674	1.6852	0.2035	UP	Pxdn
ENSMUSG00000020677	4.9803	0.199	UP	Ddx52
ENSMUSG00000020692	1.5127	0.1591	UP	Nle1
ENSMUSG00000020736	1.3735	0.1511	UP	Nt5c
ENSMUSG00000020794	1.6073	0.1403	UP	Ube2g1
ENSMUSG00000020799	1.3191	0.1894	UP	Tekt1
ENSMUSG00000020801	1.5475	0.1922	UP	Med31
ENSMUSG00000020803	5.2046	0.2789	UP	Txndc17
ENSMUSG00000020844	1.7066	0.1461	UP	Nxn
ENSMUSG00000020888	1.7911	0.1581	UP	Dvl2
ENSMUSG00000020892	1.6087	0.1858	UP	Aloxe3

ENSMUSG00000020948	1.3432	0.1426	UP	Klhl28
ENSMUSG00000020955	3.4941	0.2926	UP	Ap4s1
ENSMUSG00000021013	4.2351	0.1793	UP	Ttc8
ENSMUSG00000021033	4.1082	0.1957	UP	Gstz1
ENSMUSG00000021091	1.603	0.2068	UP	Serpina3n
ENSMUSG00000021102	2.6055	0.1981	UP	Glrx5
ENSMUSG00000021114	1.7756	0.1666	UP	Atp6v1d
ENSMUSG00000021177	2.091	0.2012	UP	Tdp1
ENSMUSG00000021196	1.7781	0.1428	UP	Pfkp
ENSMUSG00000021241	1.7696	0.1653	UP	Isca2
ENSMUSG00000021259	3.8131	0.165	UP	Cyp46a1
ENSMUSG00000021290	11.2396	0.4416	UP	Atp5mpl
ENSMUSG00000021453	3.8263	0.3734	UP	Gadd45g
ENSMUSG00000021460	3.9535	0.2279	UP	Auh
ENSMUSG00000021470	2.9414	0.1787	UP	Ercc6l2
ENSMUSG00000021549	3.9197	0.1866	UP	Rasa1
ENSMUSG00000021645	1.7468	0.159	UP	Smn1
ENSMUSG00000021668	1.5216	0.1607	UP	Polk
ENSMUSG00000021733	3.2199	0.2144	UP	Slc4a7
ENSMUSG00000021748	4.2786	0.1792	UP	Pdhb
ENSMUSG00000021771	2.8655	0.148	UP	Vdac2
ENSMUSG00000021773	1.8102	0.4078	UP	Comtd1
ENSMUSG00000021807	1.534	0.1521	UP	Rtraf
ENSMUSG00000021880	1.8089	0.3184	UP	Rnase6
ENSMUSG00000021908	1.5787	0.2136	UP	Ncoa4-ps
ENSMUSG00000021916	4.3417	0.199	UP	Glt8d1
ENSMUSG00000021928	1.3063	0.1698	UP	Ebpl
ENSMUSG00000021930	2.9768	0.1819	UP	Spryd7
ENSMUSG00000021967	3.7839	0.2305	UP	Mrpl57
ENSMUSG00000021991	8.5242	0.2435	UP	Cacna2d3
ENSMUSG00000021996	2.9842	0.1939	UP	Esd
ENSMUSG00000022108	4.5425	0.1611	UP	Itm2b
ENSMUSG00000022111	1.5216	0.3676	UP	Uchl3
ENSMUSG00000022112	4.2385	0.1804	UP	Gpc5
ENSMUSG00000022132	1.3967	0.197	UP	Cldn10
ENSMUSG00000022139	7.7169	0.1704	UP	Mbnl2
ENSMUSG00000022179	1.4855	0.2704	UP	4931414P19Rik
ENSMUSG00000022262	1.5849	0.2525	UP	Dnah5
ENSMUSG00000022309	2.7628	0.2411	UP	Angpt1
ENSMUSG00000022311	4.6061	0.2935	UP	Csmd3
ENSMUSG00000022337	2.908	0.1938	UP	Emc2
ENSMUSG00000022354	1.5079	0.1983	UP	Ndufb9
ENSMUSG00000022370	5.5695	0.3045	UP	Mrpl13
ENSMUSG00000022420	1.9923	0.1784	UP	Dnal4
ENSMUSG00000022450	2.4619	0.1754	UP	Ndufa6

ENSMUSG00000022452	2.582	0.2137	UP	Smdt1
ENSMUSG00000022550	2.1902	0.1789	UP	Adck5
ENSMUSG00000022551	2.2023	0.1685	UP	Cyc1
ENSMUSG00000022557	3.0572	0.1627	UP	Bop1
ENSMUSG00000022571	1.322	0.1647	UP	Pycrl
ENSMUSG00000022580	2.0942	0.2704	UP	Rhpn1
ENSMUSG00000022587	3.344	0.2048	UP	Ly6e
ENSMUSG00000022658	3.6822	0.2209	UP	Tagln3
ENSMUSG00000022684	9.317	0.2572	UP	Bfar
ENSMUSG00000022696	2.3675	0.1816	UP	Sidt1
ENSMUSG00000022707	2.9047	0.1869	UP	Gbe1
ENSMUSG00000022721	5.0924	0.1637	UP	Trmt2a
ENSMUSG00000022748	2.7283	0.261	UP	Cmss1
ENSMUSG00000022751	1.5785	0.1618	UP	Nit2
ENSMUSG00000022792	1.3716	0.1454	UP	Yars2
ENSMUSG00000022856	1.9958	0.218	UP	Tmem41a
ENSMUSG00000022889	4.2784	0.2327	UP	Mrpl39
ENSMUSG00000022890	2.8725	0.2466	UP	Atp5j
ENSMUSG00000022969	1.5706	0.3264	UP	Il10rb
ENSMUSG00000023089	10.6341	0.476	UP	Ndufa5
ENSMUSG00000023191	4.9175	0.2052	UP	P3h3
ENSMUSG00000023460	2.988	0.1505	UP	Rab12
ENSMUSG00000023707	1.643	0.1426	UP	Ogfod2
ENSMUSG00000023852	1.9573	0.1731	UP	Chd1
ENSMUSG00000023912	3.7831	0.1987	UP	Slc25a27
ENSMUSG00000023932	2.5243	0.2109	UP	Cdc5l
ENSMUSG00000024038	1.6189	0.1399	UP	Ndufv3
ENSMUSG00000024059	6.4601	0.1838	UP	Clip4
ENSMUSG00000024067	2.1167	0.2207	UP	Dpy30
ENSMUSG00000024084	2.7417	0.1552	UP	Qpct
ENSMUSG00000024099	4.4653	0.2653	UP	Ndufv2
ENSMUSG00000024132	3.9663	0.2921	UP	Eci1
ENSMUSG00000024158	4.9118	0.1861	UP	Hagh
ENSMUSG00000024186	2.4999	0.1639	UP	Rgs11
ENSMUSG00000024205	2.2757	1.285	UP	Rpl36-ps2
ENSMUSG00000024211	2.1137	0.16	UP	Grm8
ENSMUSG00000024248	2.1168	0.1485	UP	Cox7a2l
ENSMUSG00000024258	2.4178	0.1969	UP	Polr2d
ENSMUSG00000024271	4.9649	0.1434	UP	Elp2
ENSMUSG00000024293	2.1515	0.1547	UP	Esco1
ENSMUSG00000024299	3.1132	0.1545	UP	Adamts10
ENSMUSG00000024421	1.4163	0.2906	UP	Lama3
ENSMUSG00000024454	6.7038	0.226	UP	Hdac3
ENSMUSG00000024580	1.4788	0.1425	UP	Grpel2
ENSMUSG00000024645	1.8435	0.1604	UP	Timm21

ENSMUSG00000024668	1.8952	0.1453	UP	Sdhaf2
ENSMUSG00000024761	3.1308	0.655	UP	Gm16437
ENSMUSG00000024766	1.3642	0.1782	UP	Lipo3
ENSMUSG00000024790	1.7206	0.2162	UP	Sac3d1
ENSMUSG00000024829	1.9177	0.2091	UP	Mrpl21
ENSMUSG00000024844	5.0564	0.3291	UP	Banf1
ENSMUSG00000024845	7.8037	0.339	UP	Tmem134
ENSMUSG00000024871	3.0687	0.5468	UP	Doc2g
ENSMUSG00000024875	1.469	0.1576	UP	Yif1a
ENSMUSG00000024899	1.7658	0.159	UP	Papss2
ENSMUSG00000024902	2.2561	0.1714	UP	Mrpl11
ENSMUSG00000024911	2.6445	0.1707	UP	Fibp
ENSMUSG00000024925	4.8391	0.3336	UP	Rnaseh2c
ENSMUSG00000024953	6.1511	0.2962	UP	Prdx5
ENSMUSG00000024993	2.5246	0.1637	UP	Dennd10
ENSMUSG00000025135	6.0419	0.3031	UP	Anapc11
ENSMUSG00000025201	5.4318	2.0883	UP	Bloc1s2-ps
ENSMUSG00000025207	3.2435	0.1764	UP	Sema4g
ENSMUSG00000025260	2.654	0.2451	UP	Hsd17b10
ENSMUSG00000025278	3.1022	0.164	UP	Flnb
ENSMUSG00000025289	1.6947	0.1726	UP	Prdx4
ENSMUSG00000025321	2.3063	0.1461	UP	Itgb8
ENSMUSG00000025324	2.0951	0.3262	UP	Atp10a
ENSMUSG00000025381	3.5329	0.2062	UP	Cnpy2
ENSMUSG00000025395	1.9309	0.2351	UP	Prim1
ENSMUSG00000025409	1.8073	0.1534	UP	Mbd6
ENSMUSG00000025436	2.3893	0.2462	UP	Atp23
ENSMUSG00000025468	1.4619	0.1416	UP	Caly
ENSMUSG00000025479	3.0372	1.5867	UP	Cyp2e1
ENSMUSG00000025487	8.7386	0.24	UP	Psmd13
ENSMUSG00000025499	2.7352	0.223	UP	Hras
ENSMUSG00000025512	2.4421	0.1388	UP	Chid1
ENSMUSG00000025529	1.4503	0.151	UP	Zfp711
ENSMUSG00000025531	1.9944	0.1699	UP	Chm
ENSMUSG00000025545	5.4874	0.3257	UP	Clybl
ENSMUSG00000025580	2.7945	0.1659	UP	Eif4a3
ENSMUSG00000025732	1.3586	0.1797	UP	Mcrip2
ENSMUSG00000025789	1.4991	0.1521	UP	St8sia2
ENSMUSG00000025790	2.0407	0.1819	UP	Slco3a1
ENSMUSG00000025825	2.7623	0.2112	UP	Iscu
ENSMUSG00000025856	2.6445	0.2201	UP	Pdgfa
ENSMUSG00000025902	1.6128	0.2381	UP	Sox17
ENSMUSG00000025903	1.7875	0.1588	UP	Lypla1
ENSMUSG00000025909	2.6574	0.2102	UP	Sntg1
ENSMUSG00000026017	2.1232	0.2347	UP	Carf

ENSMUSG00000026087	2.9842	0.1845	UP	Mrpl30
ENSMUSG00000026131	3.0765	0.1558	UP	Dst
ENSMUSG00000026134	1.3738	0.2428	UP	Prim2
ENSMUSG00000026150	2.7628	0.1471	UP	Mff
ENSMUSG00000026270	2.7462	0.1711	UP	Capn10
ENSMUSG00000026281	2.4661	0.1421	UP	Dtymk
ENSMUSG00000026404	2.4793	0.2487	UP	Ddx59
ENSMUSG00000026511	3.2135	0.2102	UP	Srp9
ENSMUSG00000026727	3.4856	0.2159	UP	Rsu1
ENSMUSG00000026839	3.2885	0.1918	UP	Upp2
ENSMUSG00000026842	1.684	0.1437	UP	Abl1
ENSMUSG00000026926	3.8392	0.1649	UP	Pmpca
ENSMUSG00000026972	2.0357	0.1936	UP	Arrdc1
ENSMUSG00000026974	2.6254	0.1721	UP	Zmynd19
ENSMUSG00000026977	2.6397	0.1646	UP	Marchf7
ENSMUSG00000027133	7.9611	0.3638	UP	Nop10
ENSMUSG00000027160	6.29	0.4182	UP	Ccdc34
ENSMUSG00000027167	2.3668	0.2276	UP	Elp4
ENSMUSG00000027188	2.6264	0.2863	UP	Pamr1
ENSMUSG00000027239	2.4533	0.286	UP	Mdk
ENSMUSG00000027384	2.7805	0.2127	UP	Ndufaf5
ENSMUSG00000027404	2.3448	0.1642	UP	Snrpb
ENSMUSG00000027406	2.1686	0.145	UP	Idh3b
ENSMUSG00000027433	5.4493	0.2062	UP	Xrn2
ENSMUSG00000027498	3.8811	0.1965	UP	Cstf1
ENSMUSG00000027569	1.9028	0.1554	UP	Mrgbp
ENSMUSG00000027667	2.165	0.1405	UP	Zfp639
ENSMUSG00000027774	3.1592	0.1456	UP	Gfm1
ENSMUSG00000027800	1.5236	0.2023	UP	Tm4sf1
ENSMUSG00000027809	3.2663	0.1941	UP	Etfdh
ENSMUSG00000027953	2.7676	0.197	UP	Slc50a1
ENSMUSG00000027966	2.8275	0.205	UP	Col11a1
ENSMUSG00000027981	4.869	0.2744	UP	Rnpc3
ENSMUSG00000028013	3.4856	0.2128	UP	Ppa2
ENSMUSG00000028018	2.3782	0.223	UP	Gstcd
ENSMUSG00000028070	4.6698	0.2756	UP	Naxe
ENSMUSG00000028100	2.043	0.2468	UP	Nudt17
ENSMUSG00000028138	3.1121	0.1766	UP	Adh5
ENSMUSG00000028161	14.8568	0.2703	UP	Ppp3ca
ENSMUSG00000028184	5.0134	0.2107	UP	Adgrl2
ENSMUSG00000028218	6.0119	0.2438	UP	Cibar1
ENSMUSG00000028224	1.9315	0.162	UP	Nbn
ENSMUSG00000028271	5.1115	0.2445	UP	Gtf2b
ENSMUSG00000028294	2.0313	0.3652	UP	Cfap206
ENSMUSG00000028322	2.5824	0.253	UP	Exosc3

ENSMUSG00000028367	9.317	0.3855	UP	Txn1
ENSMUSG00000028399	2.3248	0.1798	UP	Ptprd
ENSMUSG00000028441	3.2168	0.4909	UP	1110017D15Rik
ENSMUSG00000028455	2.118	0.1705	UP	Stoml2
ENSMUSG00000028461	1.5708	0.1957	UP	Ccdc107
ENSMUSG00000028470	1.6612	0.2165	UP	Hint2
ENSMUSG00000028478	2.4607	0.1487	UP	Clta
ENSMUSG00000028549	1.378	0.2242	UP	Itgb3bp
ENSMUSG00000028572	5.1329	0.1742	UP	Hook1
ENSMUSG00000028649	4.9065	0.1564	UP	Macf1
ENSMUSG00000028671	6.4788	0.3318	UP	Gale
ENSMUSG00000028729	3.3579	0.1482	UP	Ebna1bp2
ENSMUSG00000028741	2.9643	0.2043	UP	Mrto4
ENSMUSG00000028793	4.0755	0.1899	UP	Rnf19b
ENSMUSG00000028898	2.7706	0.2475	UP	Trnau1ap
ENSMUSG00000028910	1.3819	0.1451	UP	Mecr
ENSMUSG00000028932	6.2856	0.1952	UP	Psmc2
ENSMUSG00000028936	7.6716	0.2224	UP	Rpl22
ENSMUSG00000028964	2.893	0.1866	UP	Park7
ENSMUSG00000028969	3.0987	0.1487	UP	Cdk5
ENSMUSG00000029012	2.1065	0.2028	UP	Orc5
ENSMUSG00000029068	3.417	0.1596	UP	Ccnl2
ENSMUSG00000029152	4.8369	0.1872	UP	Ociad1
ENSMUSG00000029179	1.4519	0.1541	UP	Zcchc4
ENSMUSG00000029198	3.9802	0.2172	UP	Grpel1
ENSMUSG00000029206	1.8108	0.1643	UP	Nsun7
ENSMUSG00000029245	1.4776	0.201	UP	Epha5
ENSMUSG00000029250	3.7915	0.1474	UP	Polr2b
ENSMUSG00000029334	1.3089	0.1841	UP	Prkg2
ENSMUSG00000029432	2.8095	0.1603	UP	Nipsnap2
ENSMUSG00000029535	1.4387	0.1737	UP	Triap1
ENSMUSG00000029559	3.746	0.1728	UP	2210016L21Rik
ENSMUSG00000029575	3.6464	0.1929	UP	Mmab
ENSMUSG00000029614	1.9003	0.1476	UP	Rpl6
ENSMUSG00000029617	1.3738	0.1566	UP	Ccz1
ENSMUSG00000029633	3.2994	1.4857	UP	Gm5578
ENSMUSG00000029681	2.4534	0.1835	UP	Bcl7b
ENSMUSG00000029725	1.618	0.2518	UP	Ppp1r35
ENSMUSG00000029822	2.6371	0.2469	UP	Osbpl3
ENSMUSG00000029831	2.3576	0.3957	UP	Npvf
ENSMUSG00000029864	1.8448	0.2183	UP	Gstk1
ENSMUSG00000029993	9.3087	0.3957	UP	Nfu1
ENSMUSG00000030036	2.886	0.1794	UP	Mogs
ENSMUSG00000030083	4.4215	0.2218	UP	Abtb1
ENSMUSG00000030092	2.0357	0.1532	UP	Cntn6

ENSMUSG00000030127	4.54	0.1575	UP	Cops7a
ENSMUSG00000030204	3.7264	0.179	UP	Ddx47
ENSMUSG00000030235	2.6618	0.1853	UP	Slco1c1
ENSMUSG00000030237	1.9721	0.2461	UP	Slco1a4
ENSMUSG00000030287	3.9328	0.2425	UP	Itpr2
ENSMUSG00000030309	2.8055	0.1731	UP	Caprin2
ENSMUSG00000030314	1.8831	0.1587	UP	Atg7
ENSMUSG00000030335	2.4479	0.2022	UP	Mrpl51
ENSMUSG00000030407	2.5375	0.1685	UP	Qpctl
ENSMUSG00000030431	2.4121	0.4146	UP	Tmem238
ENSMUSG00000030432	2.9923	0.2213	UP	Rpl28
ENSMUSG00000030471	3.7098	0.2055	UP	Zdhhc13
ENSMUSG00000030538	3.1894	0.2858	UP	Cib1
ENSMUSG00000030614	2.0521	0.1527	UP	Tmem126b
ENSMUSG00000030638	1.9614	0.1524	UP	Sh3gl3
ENSMUSG00000030647	4.2173	0.2645	UP	Ndufc2
ENSMUSG00000030652	2.3198	0.1881	UP	Coq7
ENSMUSG00000030671	2.0368	0.199	UP	Pde3b
ENSMUSG00000030682	2.9924	0.1505	UP	Cdipt
ENSMUSG00000030726	1.4482	0.1493	UP	Pold3
ENSMUSG00000030727	2.621	0.2015	UP	Rabep2
ENSMUSG00000030842	1.8104	0.1398	UP	Lamtor1
ENSMUSG00000030946	2.32	0.2406	UP	Lhpp
ENSMUSG00000030986	3.0884	0.1608	UP	Dhx32
ENSMUSG00000031059	2.1883	0.19	UP	Ndufb11
ENSMUSG00000031133	1.643	0.1384	UP	Arhgef6
ENSMUSG00000031169	3.2357	0.2089	UP	Porcn
ENSMUSG00000031170	1.4771	0.3937	UP	Slc38a5
ENSMUSG00000031320	3.847	0.2034	UP	Rps4x
ENSMUSG00000031367	4.472	0.2003	UP	Ap1s2
ENSMUSG00000031391	6.538	0.2321	UP	L1cam
ENSMUSG00000031432	2.3517	0.1451	UP	Prps1
ENSMUSG00000031533	1.4918	0.1649	UP	Mrps31
ENSMUSG00000031551	1.8781	0.8363	UP	Ido1
ENSMUSG00000031556	2.4684	0.1633	UP	Tm2d2
ENSMUSG00000031570	2.1686	0.146	UP	Plpp5
ENSMUSG00000031591	4.4197	0.191	UP	Asah1
ENSMUSG00000031604	1.7245	0.14	UP	Msmo1
ENSMUSG00000031617	4.5649	0.1663	UP	Tmem184c
ENSMUSG00000031633	2.6574	0.1625	UP	Slc25a4
ENSMUSG00000031644	2.2038	0.1399	UP	Nek1
ENSMUSG00000031654	2.4986	0.1783	UP	Cbln1
ENSMUSG00000031760	19.2064	0.8872	UP	Mt3
ENSMUSG00000031774	2.3544	0.1453	UP	Psme3ip1
ENSMUSG00000031776	5.1934	0.1562	UP	Arl2bp

ENSMUSG00000031792	1.6588	0.1852	UP	Usb1
ENSMUSG00000031827	3.604	0.1568	UP	Cotl1
ENSMUSG00000031839	3.3829	0.2161	UP	Hsbp1
ENSMUSG00000031848	3.6805	0.2435	UP	Lsm4
ENSMUSG00000031853	1.4968	0.421	UP	Map3k21
ENSMUSG00000031878	3.6586	0.167	UP	Nae1
ENSMUSG00000031879	9.8309	0.4688	UP	Ciao2b
ENSMUSG00000031907	1.4353	0.1499	UP	Zfp90
ENSMUSG00000032026	2.0509	0.1962	UP	Rexo2
ENSMUSG00000032198	1.7142	0.1746	UP	Dock6
ENSMUSG00000032235	2.2417	0.1672	UP	Ice2
ENSMUSG00000032258	1.6138	0.1977	UP	Lca5
ENSMUSG00000032263	1.9108	0.2197	UP	Bckdhb
ENSMUSG00000032336	7.2451	0.1958	UP	Nptn
ENSMUSG00000032359	2.1597	0.3487	UP	Ctsh
ENSMUSG00000032372	1.517	0.3186	UP	Plscr2
ENSMUSG00000032388	3.9915	0.2033	UP	Spg21
ENSMUSG00000032410	1.4084	0.1615	UP	Xrn1
ENSMUSG00000032489	1.8737	0.212	UP	Kif9
ENSMUSG00000032551	1.4387	0.1842	UP	1110059G10Rik
ENSMUSG00000032553	2.3063	0.1607	UP	Srprb
ENSMUSG00000032563	3.5344	0.1705	UP	Mrpl3
ENSMUSG00000032679	6.42	0.4464	UP	Cd59a
ENSMUSG00000032743	1.5782	0.1631	UP	Katnip
ENSMUSG00000032757	1.3564	0.1548	UP	Bet1
ENSMUSG00000032796	1.5037	0.2468	UP	Lama1
ENSMUSG00000032939	4.8886	0.2438	UP	Nup93
ENSMUSG00000032959	2.2618	0.1933	UP	Pebp1
ENSMUSG00000033004	7.4896	0.2126	UP	Mycbp2
ENSMUSG00000033020	5.73	0.3717	UP	Polr2f
ENSMUSG00000033053	1.5341	0.406	UP	1700028P14Rik
ENSMUSG00000033216	1.7158	0.1624	UP	Eefsec
ENSMUSG00000033257	1.5797	0.1514	UP	Ttll4
ENSMUSG00000033278	2.4876	0.1737	UP	Ptprm
ENSMUSG00000033307	2.3251	0.1651	UP	Mif
ENSMUSG00000033423	4.5716	0.1522	UP	Eri3
ENSMUSG00000033429	4.4419	0.3509	UP	Mcee
ENSMUSG00000033467	4.5662	0.3001	UP	Crlf2
ENSMUSG00000033499	9.1069	0.2577	UP	Larp4b
ENSMUSG00000033569	4.2611	0.1818	UP	Adgrb3
ENSMUSG00000033752	2.3522	1.2899	UP	Mnd1
ENSMUSG00000033916	5.9753	0.2713	UP	Chmp2a
ENSMUSG00000033981	5.9	0.1529	UP	Gria2
ENSMUSG00000034022	3.1081	0.1567	UP	Cpsf1
ENSMUSG00000034118	1.6378	0.1533	UP	Tpst1

ENSMUSG00000034163	2.6188	0.1689	UP	Zfc3h1
ENSMUSG00000034177	1.4094	0.3471	UP	Rnf43
ENSMUSG00000034226	2.2239	0.1823	UP	Rhov
ENSMUSG00000034353	6.3622	0.2342	UP	Ramp1
ENSMUSG00000034361	1.8865	0.1385	UP	Cpne2
ENSMUSG00000034462	1.7402	0.1419	UP	Pkd2
ENSMUSG00000034525	1.6868	0.1386	UP	Ice1
ENSMUSG00000034620	2.1404	0.1837	UP	Rxylt1
ENSMUSG00000034729	1.9424	0.1733	UP	Mrps10
ENSMUSG00000034761	2.7361	0.1489	UP	Map4k5
ENSMUSG00000034774	1.4627	1.4576	UP	Dsg1c
ENSMUSG00000034796	3.669	0.1712	UP	Cpne7
ENSMUSG00000034813	3.0129	0.2006	UP	Grip1
ENSMUSG00000034839	3.1428	0.1484	UP	Larp6
ENSMUSG00000034880	1.8659	0.2421	UP	Mrpl34
ENSMUSG00000034932	1.8088	0.225	UP	Mrpl54
ENSMUSG00000035024	3.5283	0.1836	UP	Ncapd3
ENSMUSG00000035129	1.3723	0.663	UP	Gm6781
ENSMUSG00000035133	3.7655	0.1684	UP	Arhgap5
ENSMUSG00000035198	4.8458	0.2305	UP	Tubg1
ENSMUSG00000035202	2.7277	0.7435	UP	Lars2
ENSMUSG00000035235	2.5447	0.2907	UP	Trim13
ENSMUSG00000035268	3.5463	0.1837	UP	Pkig
ENSMUSG00000035337	3.0214	1.0919	UP	Uchl4
ENSMUSG00000035472	1.5568	0.6779	UP	Slc25a21
ENSMUSG00000035539	1.6797	0.3282	UP	Ccdc180
ENSMUSG00000035674	4.0969	0.3265	UP	Ndufa3
ENSMUSG00000035772	2.3392	0.1722	UP	Mrps2
ENSMUSG00000035919	2.1341	0.1552	UP	Bbs9
ENSMUSG00000035953	6.2284	0.2133	UP	Pip4p1
ENSMUSG00000036036	9.5446	0.3313	UP	Zfp57
ENSMUSG00000036073	2.1173	0.166	UP	Galt
ENSMUSG00000036078	2.0198	0.1669	UP	Sigmar1
ENSMUSG00000036241	2.6365	0.1632	UP	Ube2r2
ENSMUSG00000036292	2.433	0.285	UP	Gramd1c
ENSMUSG00000036305	9.3544	1.4913	UP	Rpl39-ps
ENSMUSG00000036372	1.5206	0.2157	UP	Tmem258
ENSMUSG00000036403	1.7573	0.1675	UP	Cep135
ENSMUSG00000036427	6.6635	0.1616	UP	Gpi1
ENSMUSG00000036552	1.7871	0.2258	UP	Ermard
ENSMUSG00000036568	2.9725	0.1822	UP	Bicral
ENSMUSG00000036572	2.5009	0.1759	UP	Upf3b
ENSMUSG00000036578	9.5008	0.3289	UP	Fxyd7
ENSMUSG00000036748	1.8579	0.1557	UP	Cuedc2
ENSMUSG00000036781	5.1329	0.4045	UP	Rps27l

ENSMUSG00000036850	5.919	0.3453	UP	Mrpl41
ENSMUSG00000036879	1.5773	0.1379	UP	Phkb
ENSMUSG00000036898	3.7916	0.1583	UP	Zfp157
ENSMUSG00000037072	3.6118	0.1835	UP	Selenof
ENSMUSG00000037166	1.7315	0.285	UP	Ppp1r14a
ENSMUSG00000037196	2.4448	0.2254	UP	Pacrg
ENSMUSG00000037254	1.4059	1.2613	UP	Itih2
ENSMUSG00000037270	4.4844	0.1557	UP	4932438A13Rik
ENSMUSG00000037300	2.2636	0.1448	UP	Ttc13
ENSMUSG00000037416	5.13	0.217	UP	Dmxl1
ENSMUSG00000037475	2.9301	0.1595	UP	Thoc2
ENSMUSG00000037499	5.7436	0.3223	UP	Nenf
ENSMUSG00000037563	8.0683	0.3459	UP	Rps16
ENSMUSG00000037716	2.4279	0.6277	UP	Ccdc33
ENSMUSG00000037736	2.6461	0.1444	UP	Limch1
ENSMUSG00000037772	6.9904	0.3504	UP	Mrpl23
ENSMUSG00000037805	2.0915	0.1622	UP	Rpl10a
ENSMUSG00000037815	3.3209	0.1508	UP	Ctnna1
ENSMUSG00000037818	2.3188	0.1801	UP	Abhd18
ENSMUSG00000037843	10.8113	0.3628	UP	Vstm2l
ENSMUSG00000037922	1.5267	0.5693	UP	Bank1
ENSMUSG00000037940	1.7757	0.1799	UP	Inpp4b
ENSMUSG00000037962	1.6578	0.3567	UP	Rflna
ENSMUSG00000037966	6.2582	0.3011	UP	Ninj1
ENSMUSG00000037971	1.4398	0.1654	UP	1110032A03Rik
ENSMUSG00000038005	2.0335	0.159	UP	Hpf1
ENSMUSG00000038046	1.3096	0.1666	UP	Mrm3
ENSMUSG00000038079	2.8083	0.2127	UP	Tmem237
ENSMUSG00000038094	2.165	0.1864	UP	Atp13a4
ENSMUSG00000038102	3.5055	0.1563	UP	Trappc11
ENSMUSG00000038112	5.9371	0.1596	UP	AW551984
ENSMUSG00000038122	1.7402	0.1575	UP	Tbc1d32
ENSMUSG00000038175	3.2112	0.2185	UP	Mylip
ENSMUSG00000038274	6.4319	0.3551	UP	Fau
ENSMUSG00000038387	6.0544	0.4543	UP	Rras
ENSMUSG00000038510	1.9277	0.1725	UP	Rpf2
ENSMUSG00000038570	1.3969	0.2286	UP	Saxo2
ENSMUSG00000038602	1.3038	0.1471	UP	Slc35f1
ENSMUSG00000038605	4.7978	0.2226	UP	Samd10
ENSMUSG00000038690	2.1952	0.1985	UP	Atp5j2
ENSMUSG00000038697	1.4901	0.1511	UP	Taf5l
ENSMUSG00000038736	1.3984	0.1414	UP	Nudcd1
ENSMUSG00000038781	1.7187	0.3707	UP	Stap2
ENSMUSG00000038879	1.8098	0.1818	UP	Nipal2
ENSMUSG00000039001	2.2064	0.2272	UP	Rps21

ENSMUSG00000039048	2.272	0.1427	UP	Foxred1
ENSMUSG00000039183	3.8248	0.2707	UP	Nubp2
ENSMUSG00000039263	2.7181	0.1886	UP	Npepl1
ENSMUSG00000039278	13.4897	0.3758	UP	Pcsk1n
ENSMUSG00000039431	3.8089	0.163	UP	Mtmr7
ENSMUSG00000039568	3.2555	0.1746	UP	Ubald1
ENSMUSG00000039617	1.8026	1.0953	UP	Gm7488
ENSMUSG00000039680	8.3866	0.4719	UP	Mrps6
ENSMUSG00000039765	1.4153	0.1787	UP	Cc2d2a
ENSMUSG00000040003	3.0377	0.1547	UP	Magi2
ENSMUSG00000040139	1.6065	0.4264	UP	9430038I01Rik
ENSMUSG00000040181	1.422	0.1817	UP	Fmo1
ENSMUSG00000040323	4.8536	0.9224	UP	Gm15429
ENSMUSG00000040414	7.8899	0.2665	UP	Slc25a28
ENSMUSG00000040464	1.4706	0.2138	UP	Gtpbp10
ENSMUSG00000040473	3.3109	0.1812	UP	Cfap69
ENSMUSG00000040540	4.2115	0.6703	UP	Ctdsp2-ps
ENSMUSG00000040543	2.5352	0.1566	UP	Pitpnm3
ENSMUSG00000040653	2.9574	0.1823	UP	Ppp1r14c
ENSMUSG00000040658	2.1428	0.3194	UP	Dnph1
ENSMUSG00000040699	2.1972	0.1759	UP	Limd2
ENSMUSG00000040713	2.5816	0.1578	UP	Creg1
ENSMUSG00000040767	3.3862	0.3046	UP	Snrnp25
ENSMUSG00000040824	4.1345	0.3556	UP	Snrpd2
ENSMUSG00000040852	1.5077	0.1814	UP	Plekhh2
ENSMUSG00000040938	4.4715	0.1785	UP	Slc16a11
ENSMUSG00000041084	1.6403	0.1867	UP	Ostc
ENSMUSG00000041144	2.5401	0.2268	UP	Dnah7b
ENSMUSG00000041199	2.8864	0.1832	UP	Rpusd1
ENSMUSG00000041203	3.3715	0.2215	UP	Trir
ENSMUSG00000041215	3.189	0.1728	UP	Yeats2
ENSMUSG00000041235	1.4684	0.161	UP	Chd7
ENSMUSG00000041268	4.1192	0.1541	UP	Dmxl2
ENSMUSG00000041309	1.9473	0.1719	UP	Nkx6-2
ENSMUSG00000041355	1.6216	0.1549	UP	Ssr2
ENSMUSG00000041453	7.9407	1.3861	UP	Rpl21
ENSMUSG00000041483	1.5194	0.1569	UP	Zfp281
ENSMUSG00000041556	1.7406	0.154	UP	Fbxo2
ENSMUSG00000041650	4.2309	0.2119	UP	Pcca
ENSMUSG00000041697	9.1209	0.3949	UP	Cox6a1
ENSMUSG00000041771	3.4444	0.3055	UP	Slc24a4
ENSMUSG00000041841	3.4252	0.2244	UP	Rpl37
ENSMUSG00000041881	3.3043	0.2308	UP	Ndufa7
ENSMUSG00000041921	2.0845	0.1482	UP	Metap1d
ENSMUSG00000041936	2.2856	0.2042	UP	Agrn

ENSMUSG00000041939	2.0769	0.1833	UP	Mvk
ENSMUSG00000041959	2.2437	0.2194	UP	S100a10
ENSMUSG00000042195	2.2283	0.2858	UP	Slc35f2
ENSMUSG00000042293	2.4185	0.3753	UP	Gm5617
ENSMUSG00000042369	1.5804	0.1537	UP	Rbm45
ENSMUSG00000042380	1.7782	0.1871	UP	Smim12
ENSMUSG00000042396	3.7288	0.2186	UP	Rbm7
ENSMUSG00000042406	2.3904	0.1575	UP	Atf4
ENSMUSG00000042462	4.1853	0.4679	UP	Dctpp1
ENSMUSG00000042541	2.206	0.2105	UP	Sem1
ENSMUSG00000042558	3.8207	0.1984	UP	Adprs
ENSMUSG00000042616	4.2498	0.1643	UP	Oscp1
ENSMUSG00000042670	2.6946	0.4245	UP	Immp1l
ENSMUSG00000042705	1.9495	0.1774	UP	Commd10
ENSMUSG00000042737	1.6145	0.2128	UP	Dpm3
ENSMUSG00000042814	1.3936	0.2456	UP	Mcts2
ENSMUSG00000042962	7.0004	0.8581	UP	Gm5436
ENSMUSG00000043165	15.0491	0.5824	UP	Lor
ENSMUSG00000043192	17.5901	2.2455	UP	Gpi-ps
ENSMUSG00000043441	1.5216	0.1658	UP	Gpr149
ENSMUSG00000043445	2.1549	0.1528	UP	Pgp
ENSMUSG00000043483	5.1061	0.2883	UP	Gm6863
ENSMUSG00000043614	1.8798	0.1472	UP	Vps37d
ENSMUSG00000043719	1.6027	0.2665	UP	Col6a6
ENSMUSG00000043866	1.5347	0.1602	UP	Taf10
ENSMUSG00000044033	2.1872	0.1794	UP	Ccdc141
ENSMUSG00000044229	1.3999	0.2132	UP	Nxpe4
ENSMUSG00000044285	2.7443	0.6785	UP	Ubb-ps
ENSMUSG00000044330	2.6951	1.4423	UP	Gm9790
ENSMUSG00000044424	13.0473	0.8096	UP	Gm9493
ENSMUSG00000044442	2.5845	0.1974	UP	N6amt1
ENSMUSG00000044501	2.097	0.1844	UP	Zfp758
ENSMUSG00000044528	2.1713	0.2099	UP	Tram1l1
ENSMUSG00000044709	7.1381	0.3417	UP	Gemin7
ENSMUSG00000044751	2.3737	0.6084	UP	Atp5pb-ps
ENSMUSG00000044757	3.7314	2.411	UP	Gm6430
ENSMUSG00000044783	5.8255	0.2995	UP	Hjurp
ENSMUSG00000044791	3.5793	0.1567	UP	Setd2
ENSMUSG00000044906	3.1001	0.2294	UP	4930503L19Rik
ENSMUSG00000045055	2.9235	0.8158	UP	Rpsa-ps2
ENSMUSG00000045104	5.2005	1.7607	UP	Ldhb-ps
ENSMUSG00000045160	10.3832	0.4235	UP	Bola3
ENSMUSG00000045318	2.3331	0.1961	UP	Adra2c
ENSMUSG00000045665	1.4776	0.1552	UP	Mfsd5
ENSMUSG00000045886	7.0555	1.374	UP	Pam16l

ENSMUSG00000045948	2.5941	0.1921	UP	Mrps12
ENSMUSG00000046138	1.4079	0.143	UP	9930021J03Rik
ENSMUSG00000046169	3.2764	0.2837	UP	Adamts6
ENSMUSG00000046330	6.698	0.3634	UP	Rpl37a
ENSMUSG00000046341	4.7738	0.5902	UP	Gm11223
ENSMUSG00000046432	1.7971	0.1475	UP	Bex3
ENSMUSG00000046573	3.3357	0.2401	UP	Lyrm4
ENSMUSG00000046580	3.7426	1.7395	UP	Gm7862
ENSMUSG00000046834	8.3788	0.3464	UP	Krt1
ENSMUSG00000047067	2.4922	0.1538	UP	Dusp28
ENSMUSG00000047215	1.6816	0.1525	UP	Rpl9
ENSMUSG00000047370	4.434	0.7835	UP	Gm7367
ENSMUSG00000047394	1.579	0.2856	UP	Odf3b
ENSMUSG00000047459	3.2483	0.2684	UP	Dynlrb1
ENSMUSG00000047676	2.4316	0.3389	UP	Rpsa-ps10
ENSMUSG00000047721	14.1873	0.5811	UP	Bola2
ENSMUSG00000047766	3.8667	0.177	UP	Lrrc49
ENSMUSG00000047843	13.452	0.4621	UP	Bri3
ENSMUSG00000047965	6.4191	0.6328	UP	Rpl9-ps7
ENSMUSG00000048087	2.1979	0.2544	UP	Ahcyl
ENSMUSG00000048271	1.7772	0.1401	UP	Rbm33
ENSMUSG00000048334	1.7534	2.1596	UP	Gm8258
ENSMUSG00000048490	3.1113	0.1971	UP	Nrip1
ENSMUSG00000048644	2.4051	0.2068	UP	Ctxn1
ENSMUSG00000048709	2.2589	1.2224	UP	Gm8666
ENSMUSG00000048720	3.0267	0.1852	UP	Tbc1d12
ENSMUSG00000048758	13.2792	0.503	UP	Rpl29
ENSMUSG00000048832	1.9851	0.1768	UP	Vps37c
ENSMUSG00000049164	2.2914	0.2267	UP	Zfp518a
ENSMUSG00000049232	1.6099	0.1739	UP	Tigd2
ENSMUSG00000049233	6.1651	1.3057	UP	Apoo-ps
ENSMUSG00000049235	3.6107	1.2254	UP	Gm7324
ENSMUSG00000049336	5.9152	0.2032	UP	Tenm2
ENSMUSG00000049517	3.0717	0.2568	UP	Rps23
ENSMUSG00000049612	3.878	0.2102	UP	Omg
ENSMUSG00000049760	1.3038	0.17	UP	Micos13
ENSMUSG00000049891	16.511	2.1452	UP	Gm7984
ENSMUSG00000049940	6.5277	0.171	UP	Pgrmc2
ENSMUSG00000050234	1.7384	0.3701	UP	Gja4
ENSMUSG00000050299	25.0215	1.2919	UP	Gm9843
ENSMUSG00000050552	1.429	0.1494	UP	Lamtor4
ENSMUSG00000050822	3.6489	0.1989	UP	Slc29a4
ENSMUSG00000050855	1.5821	0.1699	UP	Zfp940
ENSMUSG00000050912	4.0915	0.2542	UP	Tmem123
ENSMUSG00000050944	1.469	0.3853	UP	Efcab5

ENSMUSG00000051022	2.9959	0.2196	UP	Hs3st1
ENSMUSG00000051062	1.9116	0.1506	UP	Fbll1
ENSMUSG00000051154	3.6236	0.1981	UP	Commd3
ENSMUSG00000051278	2.6075	0.3195	UP	Zgrf1
ENSMUSG00000051373	1.4628	0.1608	UP	Plpp7
ENSMUSG00000051483	4.118	0.2242	UP	Cbr1
ENSMUSG00000051548	2.2648	1.3873	UP	Gm6365
ENSMUSG00000051864	1.4746	0.142	UP	Tbc1d22a
ENSMUSG00000052584	1.7901	0.1857	UP	Serp2
ENSMUSG00000052681	2.7016	0.1498	UP	Rap1b
ENSMUSG00000052707	7.1745	0.1927	UP	Tnrc6a
ENSMUSG00000052726	4.4472	0.3715	UP	Kcnt2
ENSMUSG00000052825	4.4536	0.3846	UP	Gm9892
ENSMUSG00000052906	6.8552	0.3272	UP	Ubxn8
ENSMUSG00000052914	2.4424	0.1584	UP	Cyp2j6
ENSMUSG00000053038	10.0374	0.9803	UP	Gm6180
ENSMUSG00000053111	1.3718	0.2025	UP	Fank1
ENSMUSG00000053173	2.4236	1.7866	UP	Rpl18-ps2
ENSMUSG00000053565	3.0722	0.2018	UP	Eif3k
ENSMUSG00000053574	1.5642	0.2146	UP	4930563E22Rik
ENSMUSG00000053740	4.7429	0.6397	UP	Gm6457
ENSMUSG00000053931	5.4203	0.1994	UP	Cnn3
ENSMUSG00000054091	1.409	0.2007	UP	1810037I17Rik
ENSMUSG00000054099	2.1504	0.1416	UP	Slc25a40
ENSMUSG00000054312	2.0785	0.2195	UP	Mrps21
ENSMUSG00000054426	1.5023	0.29	UP	A930005H10Rik
ENSMUSG00000055078	1.4684	0.1407	UP	Gabra5
ENSMUSG00000055917	2.5823	0.2258	UP	Zfp277
ENSMUSG00000056185	6.0873	0.201	UP	Snx32
ENSMUSG00000056412	2.9777	1.2933	UP	Psenen-ps
ENSMUSG00000056569	1.3205	3.0503	UP	Mpz
ENSMUSG00000056724	1.6066	0.2086	UP	Nbeal2
ENSMUSG00000056904	1.8858	0.7714	UP	Gm5620
ENSMUSG00000057054	1.3362	0.3177	UP	Inca1
ENSMUSG00000057182	3.5553	0.1562	UP	Scn3a
ENSMUSG00000057278	1.6478	0.2518	UP	Snrpg
ENSMUSG00000057322	5.7166	0.3682	UP	Rpl38
ENSMUSG00000057375	2.4842	0.1924	UP	Yipf1
ENSMUSG00000057439	1.5608	0.5526	UP	Kir3dl2
ENSMUSG00000057469	3.5293	0.1938	UP	E2f6
ENSMUSG00000057580	5.3129	3.1291	UP	Cox7c-ps1
ENSMUSG00000057594	3.8532	0.2549	UP	Arl16
ENSMUSG00000057990	2.1902	2.0188	UP	Gm53055
ENSMUSG00000058050	4.4241	0.7559	UP	Gm9234
ENSMUSG00000058064	8.005	1.274	UP	Gm10036

ENSMUSG00000058126	8.4321	0.6847	UP	Tpm3-rs7
ENSMUSG00000058420	1.7696	0.1699	UP	Syt17
ENSMUSG00000058443	15.3588	1.9151	UP	Rpl10-ps3
ENSMUSG00000058558	2.3008	0.1721	UP	Rpl5
ENSMUSG00000058690	2.1837	0.1452	UP	Ccser2
ENSMUSG00000058740	1.6292	0.1584	UP	Kcnt1
ENSMUSG00000058809	2.5766	0.9387	UP	Hspd1-ps3
ENSMUSG00000058833	3.8667	0.2485	UP	Rex1bd
ENSMUSG00000058905	1.5847	1.5971	UP	Gm10051
ENSMUSG00000059013	1.5632	0.1413	UP	Sh2d3c
ENSMUSG00000059031	3.6177	2.3073	UP	Olfr482
ENSMUSG00000059058	2.8294	0.4358	UP	Tma7-ps
ENSMUSG00000059064	1.6263	1.3316	UP	Gm10059
ENSMUSG00000059070	11.6688	0.4142	UP	Rpl18
ENSMUSG00000059159	18.7968	2.5576	UP	Gm8129
ENSMUSG00000059183	1.9408	0.1758	UP	Mtfmt
ENSMUSG00000059195	2.6196	0.4437	UP	Gm12715
ENSMUSG00000059291	6.5771	0.358	UP	Rpl11
ENSMUSG00000059326	2.524	0.1901	UP	Csf2ra
ENSMUSG00000059461	4.528	0.9312	UP	Gm7331
ENSMUSG00000059474	2.414	0.1487	UP	Mbtd1
ENSMUSG00000059554	3.8391	0.2124	UP	Ccdc28a
ENSMUSG00000059645	1.8405	0.3349	UP	Gm7361
ENSMUSG00000059751	8.0763	1.3029	UP	Rps3a3
ENSMUSG00000059775	3.4328	1.4941	UP	Rps26-ps1
ENSMUSG00000059974	19.3246	0.3146	UP	Ntm
ENSMUSG00000060019	30.8137	2.0456	UP	Gm10073
ENSMUSG00000060073	9.9223	0.3005	UP	Psma3
ENSMUSG00000060149	1.6104	0.2065	UP	BC002059
ENSMUSG00000060198	5.2387	1.109	UP	Gm11353
ENSMUSG00000060301	3.3715	0.1861	UP	2610008E11Rik
ENSMUSG00000060377	4.9585	1.4649	UP	Rpl36a-ps1
ENSMUSG00000060419	16.3725	0.586	UP	Rps16-ps2
ENSMUSG00000060438	6.1769	0.9534	UP	Rps10-ps1
ENSMUSG00000060467	2.8489	1.0887	UP	Gm10080
ENSMUSG00000060579	2.0903	0.5193	UP	Fhit
ENSMUSG00000060636	2.6425	0.2231	UP	Rpl35a
ENSMUSG00000060647	7.8524	1.508	UP	Gm7099
ENSMUSG00000060680	2.4457	2.5175	UP	Gm8894
ENSMUSG00000060802	4.0228	0.2365	UP	B2m
ENSMUSG00000060924	2.6483	0.1748	UP	Csmd1
ENSMUSG00000060981	2.503	0.74	UP	H4c8
ENSMUSG00000061062	2.5308	0.4501	UP	Hdac1-ps
ENSMUSG00000061207	1.4684	0.1397	UP	Stk19
ENSMUSG00000061360	2.0471	0.2618	UP	Phf5a

ENSMUSG00000061474	1.3773	0.1977	UP	Mrps36
ENSMUSG00000061518	1.6175	0.1936	UP	Cox5b
ENSMUSG00000061559	1.9643	0.1483	UP	Wdr61
ENSMUSG00000061731	2.9818	0.2103	UP	Ext1
ENSMUSG00000061787	4.3489	0.2256	UP	Rps17
ENSMUSG00000061833	15.8664	2.788	UP	Gm6311
ENSMUSG00000061848	11.0877	1.4511	UP	Gm5805
ENSMUSG00000061950	3.6959	0.1752	UP	Ppp4r1
ENSMUSG00000062040	1.3911	0.1433	UP	Zfp27
ENSMUSG00000062093	1.5603	0.5108	UP	Gm10110
ENSMUSG00000062252	2.6468	0.2043	UP	Lhfpl5
ENSMUSG00000062309	2.255	0.2282	UP	Rpp25
ENSMUSG00000062611	9.7224	1.6713	UP	Rps3a2
ENSMUSG00000062646	1.5026	0.1718	UP	Ganc
ENSMUSG00000062683	14.6823	0.4138	UP	Atp5g2
ENSMUSG00000062713	1.3696	0.5144	UP	Sim2
ENSMUSG00000062753	3.0326	0.2698	UP	AI413582
ENSMUSG00000062846	1.5312	0.6781	UP	Gm14176
ENSMUSG00000062937	1.5852	0.1906	UP	Mtap
ENSMUSG00000062949	1.505	0.1408	UP	Atp11c
ENSMUSG00000062981	2.2856	0.2383	UP	Mrpl42
ENSMUSG00000063065	3.236	0.1398	UP	Mapk3
ENSMUSG00000063275	1.5368	0.3509	UP	Hacd1
ENSMUSG00000063314	6.4953	2.0845	UP	Gm12657
ENSMUSG00000063316	18.3368	0.5664	UP	Rpl27
ENSMUSG00000063406	3.0865	0.1867	UP	Tmed5
ENSMUSG00000063480	2.2617	0.1589	UP	Snu13
ENSMUSG00000063543	2.0167	2.2426	UP	Gm5616
ENSMUSG00000063600	2.0393	0.2169	UP	Egfem1
ENSMUSG00000063652	1.9023	0.2604	UP	Slc22a21
ENSMUSG00000063698	1.3646	0.1391	UP	Sfxn4
ENSMUSG00000063704	2.6361	0.2373	UP	Mapk15
ENSMUSG00000063875	2.6268	1.7207	UP	Rps6-ps1
ENSMUSG00000063882	1.4111	0.1538	UP	Uqcrh
ENSMUSG00000063887	1.7596	0.1486	UP	Nlgn1
ENSMUSG00000064208	1.4413	0.7115	UP	Gm10145
ENSMUSG00000064254	2.1238	0.2146	UP	Ethe1
ENSMUSG00000064317	10.1259	0.7073	UP	Gm10146
ENSMUSG00000064337	2.6915	0.416	UP	mt-Rnr1
ENSMUSG00000064341	3.2239	0.1933	UP	mt-Nd1
ENSMUSG00000064345	2.2648	0.1695	UP	mt-Nd2
ENSMUSG00000064354	6.1553	1.0399	UP	mt-Co2
ENSMUSG00000064357	3.0987	0.8118	UP	mt-Atp6
ENSMUSG00000064358	4.9565	0.9991	UP	mt-Co3
ENSMUSG00000064363	13.9501	0.3655	UP	mt-Nd4

ENSMUSG00000064441	2.5845	1.4078	UP	Snord37
ENSMUSG00000065538	1.3569	1.7805	UP	Mir153
ENSMUSG00000065979	4.2134	0.229	UP	Cpped1
ENSMUSG00000066000	2.4176	0.4924	UP	Zfp979
ENSMUSG00000066068	4.3723	2.2295	UP	Gm13611
ENSMUSG00000066270	7.5758	1.7478	UP	Gm10157
ENSMUSG00000066315	8.5804	0.8652	UP	Gm12918
ENSMUSG00000066362	2.6626	0.8758	UP	Rps13-ps1
ENSMUSG00000066392	1.8022	0.2148	UP	Nrxn3
ENSMUSG00000066491	28.4289	5.2933	UP	Cox6c2
ENSMUSG00000066543	4.7048	1.2572	UP	Rpl17-ps9
ENSMUSG00000066571	2.3664	0.3145	UP	Garre1
ENSMUSG00000066621	3.1656	0.1629	UP	Tecpr1
ENSMUSG00000066629	3.2451	2.3536	UP	Rpl36-ps3
ENSMUSG00000066705	2.996	0.1613	UP	Fxyd6
ENSMUSG00000066724	7.4673	1.5393	UP	Gm10175
ENSMUSG00000066842	5.5877	0.4202	UP	Hmcn1
ENSMUSG00000067028	15.8397	1.0582	UP	Cntnap5b
ENSMUSG00000067058	1.361	2.1987	UP	Rps15a-ps5
ENSMUSG00000067071	1.3545	0.1647	UP	Hes6
ENSMUSG00000067121	4.0875	1.8116	UP	Gm7027
ENSMUSG00000067147	1.8267	0.9201	UP	Rpl7a-ps11
ENSMUSG00000067161	6.1549	2.099	UP	Gm5560
ENSMUSG00000067189	8.153	0.6068	UP	Gm7335
ENSMUSG00000067288	18.6744	0.7484	UP	Rps28
ENSMUSG00000067344	2.9672	1.6691	UP	Rps25-ps1
ENSMUSG00000067377	1.8659	0.1481	UP	Tspan6
ENSMUSG00000067575	5.2403	1.4552	UP	Rpl35a-ps3
ENSMUSG00000067719	9.1987	0.509	UP	Gm10221
ENSMUSG00000067847	4.8784	0.3115	UP	Romo1
ENSMUSG00000067924	3.7823	0.2371	UP	Rtl8b
ENSMUSG00000067931	1.5079	0.1608	UP	Zfp948
ENSMUSG00000068250	1.7871	0.1689	UP	Amn1
ENSMUSG00000068329	3.6224	0.1824	UP	Htra2
ENSMUSG00000068457	2.1404	0.2285	UP	Uty
ENSMUSG00000068466	2.2648	0.2617	UP	Gm5518
ENSMUSG00000068706	6.5807	0.453	UP	Gm10250
ENSMUSG00000069011	1.5349	0.5663	UP	Gm10254
ENSMUSG00000069045	3.3041	0.2274	UP	Ddx3y
ENSMUSG00000069188	1.4307	0.5218	UP	Gm13192
ENSMUSG00000069379	9.4685	1.2853	UP	Gm4950
ENSMUSG00000069804	2.6676	1.759	UP	Gm10277
ENSMUSG00000069917	4.1159	1.3375	UP	Hba-a2
ENSMUSG00000069939	2.057	0.8633	UP	Gm12070
ENSMUSG00000069972	5.8035	1.0414	UP	Rps13-ps2

ENSMUSG00000070003	4.4957	0.1959	UP	Ssbp4
ENSMUSG00000070283	2.3772	0.1876	UP	Ndufaf3
ENSMUSG00000070343	18.7041	1.6493	UP	Gm10288
ENSMUSG00000070443	4.7529	2.0296	UP	Gm10291
ENSMUSG00000070490	3.9067	1.5398	UP	Gm10293
ENSMUSG00000070572	1.3758	0.2314	UP	Trmt112-ps2
ENSMUSG00000070729	4.2385	0.4226	UP	Gm12966
ENSMUSG00000070858	3.157	0.2737	UP	Gm1673
ENSMUSG00000070934	4.7931	0.1733	UP	Rraga
ENSMUSG00000071035	16.5402	2.0787	UP	Gm5499
ENSMUSG00000071052	2.3974	0.9973	UP	Rpl7a-ps5
ENSMUSG00000071078	2.6156	0.6097	UP	Nr2c2ap
ENSMUSG00000071103	2.1261	0.232	UP	1700029J07Rik
ENSMUSG00000071141	6.7345	2.1506	UP	Rpl36a-ps3
ENSMUSG00000071151	2.013	2.5397	UP	Gm4799
ENSMUSG00000071303	2.3383	2.0816	UP	Rps8-ps1
ENSMUSG00000071343	1.5972	1.182	UP	Gm10327
ENSMUSG00000071415	6.5297	0.297	UP	Rpl23
ENSMUSG00000071451	3.3925	0.4137	UP	Psmg4
ENSMUSG00000071528	3.0733	0.2733	UP	Atp5md
ENSMUSG00000071640	5.1288	1.056	UP	Stxbp3-ps
ENSMUSG00000071711	2.0785	0.1845	UP	Mpst
ENSMUSG00000071748	2.1445	0.6432	UP	Styx-ps
ENSMUSG00000071867	2.378	0.7532	UP	Mrpl27-ps
ENSMUSG00000072387	3.2925	1.8484	UP	Gm10356
ENSMUSG00000072407	10.9321	1.5364	UP	Gm6419
ENSMUSG00000072582	1.6569	0.1463	UP	Ptrh2
ENSMUSG00000072663	2.2476	0.3049	UP	Spef2
ENSMUSG00000072694	1.6997	0.1426	UP	1500011B03Rik
ENSMUSG00000072789	1.3553	1.7442	UP	Gm10420
ENSMUSG00000072889	3.443	0.2043	UP	Nfxl1
ENSMUSG00000072915	1.5339	0.1546	UP	Gm12258
ENSMUSG00000072940	12.3687	0.8427	UP	Gm10443
ENSMUSG00000073116	5.324	2.7454	UP	Gm10471
ENSMUSG00000073433	3.1229	0.2325	UP	Arhgdig
ENSMUSG00000073478	1.4809	0.2798	UP	D730003I15Rik
ENSMUSG00000073633	3.5943	0.4716	UP	Fbxo36
ENSMUSG00000073640	1.4398	0.8889	UP	Rpl27-ps3
ENSMUSG00000073647	2.1092	0.2997	UP	Gm10557
ENSMUSG00000073676	2.3772	0.235	UP	Hspe1
ENSMUSG00000073684	2.2126	0.1658	UP	Faap20
ENSMUSG00000073702	9.7492	0.4482	UP	Rpl31
ENSMUSG00000073737	2.4619	1.3695	UP	Gm10566
ENSMUSG00000073775	2.3792	0.2429	UP	Kti12
ENSMUSG00000074166	1.4916	0.1913	UP	AW146154

ENSMUSG00000074247	6.0437	0.2325	UP	Dda1
ENSMUSG00000074264	1.75	0.1559	UP	Amy1
ENSMUSG00000074476	2.4097	0.6548	UP	Spc24
ENSMUSG00000074516	5.5722	0.4416	UP	Gm10709
ENSMUSG00000074733	1.4319	0.1628	UP	Zfp950
ENSMUSG00000074754	7.2258	0.42	UP	Smim26
ENSMUSG00000074797	1.4221	0.1644	UP	Itpa
ENSMUSG00000074868	1.6966	2.6246	UP	Gm6478
ENSMUSG00000074922	1.6415	0.1579	UP	Fam122a
ENSMUSG00000075012	3.4209	0.222	UP	Fjx1
ENSMUSG00000075053	42.9701	0.7761	UP	Vdac3-ps1
ENSMUSG00000075279	3.1168	1.1602	UP	Mrpl23-ps1
ENSMUSG00000075581	2.4532	2.7079	UP	Gm16409
ENSMUSG00000075701	1.5028	0.1722	UP	Selenos
ENSMUSG00000075702	1.4002	0.1411	UP	Selenom
ENSMUSG00000075705	1.3802	0.1988	UP	Msrb1
ENSMUSG00000075706	7.0486	0.2596	UP	Gpx4
ENSMUSG00000077637	3.9198	0.7074	UP	Gm22771
ENSMUSG00000078126	27.528	1.1577	UP	Rpl23a-ps3
ENSMUSG00000078139	4.1678	0.9609	UP	AK157302
ENSMUSG00000078180	2.2589	2.6872	UP	Gm20900
ENSMUSG00000078190	1.6029	1.4031	UP	Dnm3os
ENSMUSG00000078193	27.6328	1.1073	UP	Gm2000
ENSMUSG00000078300	1.89	0.6834	UP	Gm2606
ENSMUSG00000078317	2.521	0.2251	UP	F8a
ENSMUSG00000078377	2.2331	0.9814	UP	Gm4294
ENSMUSG00000078440	2.1544	0.2048	UP	Dohh
ENSMUSG00000078480	11.9044	1.4252	UP	Mrpl48-ps
ENSMUSG00000078572	2.2421	0.2842	UP	Ndufaf8
ENSMUSG00000078592	2.1608	1.0599	UP	Gm4609
ENSMUSG00000078695	4.8295	0.3715	UP	Cisd3
ENSMUSG00000078812	2.4743	0.1473	UP	Eif5a
ENSMUSG00000078875	1.4317	1.9746	UP	Gm14419
ENSMUSG00000078897	2.5749	1.5327	UP	Gm4724
ENSMUSG00000078967	3.8977	1.5986	UP	Gapdh-ps16
ENSMUSG00000079101	1.9239	2.1852	UP	Esd-ps
ENSMUSG00000079104	1.6979	0.2383	UP	Prps1l3
ENSMUSG00000079311	4.0109	1.6771	UP	Gm3222
ENSMUSG00000079316	6.2132	0.2868	UP	Rab9
ENSMUSG00000079339	1.6424	0.2977	UP	Ifit1bl1
ENSMUSG00000079396	2.1323	0.8391	UP	Gm3411
ENSMUSG00000079501	2.4254	1.3038	UP	Gm5138
ENSMUSG00000079508	2.5052	0.239	UP	Apoo
ENSMUSG00000079593	9.319	0.4153	UP	4933416I08Rik
ENSMUSG00000079641	24.8862	0.6888	UP	Rpl39

ENSMUSG00000079941	9.4289	0.5171	UP	Cox5b-ps
ENSMUSG00000079942	5.1267	1.4155	UP	Rpl28-ps3
ENSMUSG00000080006	1.9203	2.0916	UP	Rps19-ps7
ENSMUSG00000080242	3.1382	0.5866	UP	Atp6v0c-ps2
ENSMUSG00000080824	1.7549	0.677	UP	Gm9001
ENSMUSG00000080832	6.3144	2.0461	UP	M6pr-ps
ENSMUSG00000080848	16.2822	1.3194	UP	Gm9385
ENSMUSG00000080870	2.7979	2.7234	UP	Gm14111
ENSMUSG00000080875	1.3828	0.6117	UP	Gm7332
ENSMUSG00000080893	11.1457	1.3665	UP	Ndufa12-ps
ENSMUSG00000080902	8.474	0.5607	UP	Ywhaq-ps3
ENSMUSG00000080904	19.4474	5.2993	UP	Gm11966
ENSMUSG00000080921	4.8901	0.4219	UP	Rpl38-ps2
ENSMUSG00000080994	1.6157	1.3419	UP	Gm13464
ENSMUSG00000081010	1.6737	2.218	UP	Gm13880
ENSMUSG00000081021	3.381	1.5063	UP	Gm11964
ENSMUSG00000081049	2.9135	1.0979	UP	Rps24-ps3
ENSMUSG00000081071	1.6767	1.3654	UP	Gm9836
ENSMUSG00000081087	9.775	1.8419	UP	Rps15a-ps7
ENSMUSG00000081094	3.8496	0.5372	UP	Rpl19-ps11
ENSMUSG00000081111	1.37	0.8592	UP	Gm5913
ENSMUSG00000081113	14.3748	1.2656	UP	Gm7308
ENSMUSG00000081121	9.0121	4.7157	UP	Gm12791
ENSMUSG00000081128	1.6746	1.9199	UP	Gm13328
ENSMUSG00000081214	20.3776	2.1133	UP	Rpl35a-ps2
ENSMUSG00000081239	1.6248	1.3596	UP	Gm11836
ENSMUSG00000081262	6.9251	1.6933	UP	Gm12261
ENSMUSG00000081281	4.3535	1.203	UP	Rpl7-ps9
ENSMUSG00000081344	28.0877	1.5997	UP	Gm14303
ENSMUSG00000081378	2.156	0.8359	UP	Rps13-ps4
ENSMUSG00000081400	1.6967	0.5506	UP	Gm13680
ENSMUSG00000081406	1.8418	0.3141	UP	Rps6-ps4
ENSMUSG00000081476	3.5501	0.6344	UP	Itpa-ps1
ENSMUSG00000081559	2.2432	0.6394	UP	Gm12411
ENSMUSG00000081600	10.26	2.3637	UP	Gm12286
ENSMUSG00000081604	5.6652	1.0052	UP	Gm11518
ENSMUSG00000081673	2.7203	2.4545	UP	Gm14794
ENSMUSG00000081684	5.8809	1.3349	UP	Rps2-ps13
ENSMUSG00000081700	8.9572	3.8912	UP	Atp5k-ps2
ENSMUSG00000081788	6.6887	1.1383	UP	Gm5898
ENSMUSG00000081824	12.929	1.4826	UP	Ndufs5-ps
ENSMUSG00000081855	22.5123	1.3305	UP	Rpl17-ps5
ENSMUSG00000081871	1.8345	1.8525	UP	Gm11488
ENSMUSG00000081929	4.434	1.4489	UP	Rps11-ps2
ENSMUSG00000081992	11.0346	2.0822	UP	Gm13408

ENSMUSG00000081999	5.1436	1.0527	UP	Gm13461
ENSMUSG00000082016	2.2636	0.4397	UP	Pgam1-ps2
ENSMUSG00000082035	4.2239	1.4186	UP	Rpl17-ps8
ENSMUSG00000082044	4.1208	0.9396	UP	Snrpert
ENSMUSG00000082064	2.2816	2.5047	UP	Rpl5-ps2
ENSMUSG00000082072	5.9217	2.6969	UP	Gm15785
ENSMUSG00000082110	1.4162	1.7997	UP	Gm11196
ENSMUSG00000082193	1.5235	1.4934	UP	Rpl5-ps1
ENSMUSG00000082194	2.0634	2.0477	UP	Gm12444
ENSMUSG00000082195	1.7534	0.89	UP	Gm13034
ENSMUSG00000082226	2.3949	0.3789	UP	Gm715
ENSMUSG00000082274	2.6526	0.8858	UP	Gm14026
ENSMUSG00000082284	14.8139	1.6366	UP	H3f3a-ps1
ENSMUSG00000082321	1.6208	0.4292	UP	Gm14253
ENSMUSG00000082394	3.6717	1.6402	UP	Gm4596
ENSMUSG00000082399	5.1626	1.2491	UP	Gm14036
ENSMUSG00000082424	4.4419	1.5938	UP	Gm13292
ENSMUSG00000082431	2.0542	1.7198	UP	Prdx2-ps1
ENSMUSG00000082454	4.2357	0.5104	UP	Gm12183
ENSMUSG00000082475	12.8278	1.9694	UP	Gm7206
ENSMUSG00000082491	2.1093	2.335	UP	Gm5909
ENSMUSG00000082530	10.2202	3.0098	UP	Gm12168
ENSMUSG00000082536	38.9661	1.8097	UP	Gm13456
ENSMUSG00000082588	3.0817	1.8075	UP	Gm15443
ENSMUSG00000082691	1.4413	0.3154	UP	Dynlt1-ps1
ENSMUSG00000082765	4.599	1.2445	UP	Gm14411
ENSMUSG00000082778	3.5087	2.0001	UP	Gm15191
ENSMUSG00000082791	3.6079	1.1861	UP	Gm4875
ENSMUSG00000082809	7.1632	0.9582	UP	Gm14150
ENSMUSG00000082895	3.4081	1.5703	UP	Rpsa-ps9
ENSMUSG00000082896	9.032	1.4944	UP	Gm5844
ENSMUSG00000082953	2.2223	1.0017	UP	Gm13217
ENSMUSG00000083044	2.0542	2.3836	UP	Gm12416
ENSMUSG00000083093	1.3038	0.8478	UP	Gm15385
ENSMUSG00000083139	1.3236	0.6335	UP	Gm12418
ENSMUSG00000083283	1.4482	0.8752	UP	Gm15361
ENSMUSG00000083287	2.3624	1.0104	UP	Idi1-ps1
ENSMUSG00000083325	9.1498	0.7159	UP	Gm14121
ENSMUSG00000083327	2.2274	0.3969	UP	Vcp-rs
ENSMUSG00000083328	2.913	2.3924	UP	Gm11826
ENSMUSG00000083380	1.5603	0.5129	UP	Ndufb4c
ENSMUSG00000083424	7.9797	2.6738	UP	Rpl35a-ps4
ENSMUSG00000083443	2.1149	0.3864	UP	Gm15519
ENSMUSG00000083458	1.4371	1.1841	UP	Gm5510
ENSMUSG00000083477	1.4135	0.4241	UP	Gm5555

ENSMUSG00000083496	15.965	1.455	UP	Gm11263
ENSMUSG00000083563	38.9462	2.9929	UP	Gm13340
ENSMUSG00000083567	3.0987	0.8405	UP	Gm11451
ENSMUSG00000083619	3.1891	2.6693	UP	Gm14414
ENSMUSG00000083621	29.3487	1.7706	UP	Gm14586
ENSMUSG00000083626	7.9931	0.5157	UP	Gm4459
ENSMUSG00000083678	2.9599	0.4537	UP	Gm12989
ENSMUSG00000083679	3.1651	1.1366	UP	Gm12892
ENSMUSG00000083692	9.897	1.2546	UP	Gm9575
ENSMUSG00000083716	8.3982	1.559	UP	Gm13436
ENSMUSG00000083798	8.4651	0.8124	UP	Gm14584
ENSMUSG00000083820	16.4999	2.6135	UP	Ndufs6b
ENSMUSG00000083854	9.7655	1.4578	UP	Dnajc19-ps
ENSMUSG00000083863	5.7262	1.8807	UP	Gm13341
ENSMUSG00000083899	9.7048	0.6445	UP	Gm12346
ENSMUSG00000083902	2.0323	1.6505	UP	Tent2-ps1
ENSMUSG00000083992	12.8588	2.2436	UP	Gm11478
ENSMUSG00000084013	3.059	1.1097	UP	Gm14270
ENSMUSG00000084098	1.3469	1.6122	UP	Gm13422
ENSMUSG00000084111	3.4874	0.5739	UP	Gm15710
ENSMUSG00000084131	2.198	1.1872	UP	Rpl3-ps2
ENSMUSG00000084145	8.7132	1.7887	UP	Gm12263
ENSMUSG00000084159	11.7199	1.23	UP	Gm12696
ENSMUSG00000084166	8.7458	2.6603	UP	Gm6451
ENSMUSG00000084168	3.1022	1.1992	UP	Atp5l2-ps
ENSMUSG00000084235	3.7279	1.1376	UP	Gm15421
ENSMUSG00000084314	2.0268	1.428	UP	Rps15a-ps3
ENSMUSG00000084319	6.3024	0.4794	UP	Tpt1-ps3
ENSMUSG00000084323	2.1533	1.3892	UP	Gm14438
ENSMUSG00000084329	2.9237	1.3272	UP	Gm6733
ENSMUSG00000084349	21.7194	0.6134	UP	Rpl3-ps1
ENSMUSG00000084384	3.7191	0.4853	UP	Gm12251
ENSMUSG00000084786	2.8283	0.2164	UP	Ubl5
ENSMUSG00000084817	11.7809	1.623	UP	Gm5526
ENSMUSG00000084830	18.8489	1.2071	UP	Gm14539
ENSMUSG00000084842	2.0933	0.6008	UP	Pabpc1l2b
ENSMUSG00000084843	1.4842	0.4546	UP	B230312C02Rik
ENSMUSG00000085342	7.935	1.4836	UP	Gm12254
ENSMUSG00000085401	1.4312	1.5746	UP	Slc39a1-ps
ENSMUSG00000085711	6.0081	0.9394	UP	Gm15163
ENSMUSG00000085873	2.2004	0.2575	UP	Ttc39aos1
ENSMUSG00000085917	1.4724	0.8443	UP	Gm15899
ENSMUSG00000085939	9.2333	0.6249	UP	Cd63-ps
ENSMUSG00000086021	1.3822	0.6366	UP	Gm15767
ENSMUSG00000086240	2.7164	0.8607	UP	Gm7846

ENSMUSG00000086324	1.4668	1.0091	UP	Gm15564
ENSMUSG00000086370	6.8831	0.1896	UP	Ftx
ENSMUSG00000086567	15.9937	3.7339	UP	Gm2830
ENSMUSG00000086583	3.5112	0.3437	UP	Gm15500
ENSMUSG00000086691	3.6247	1.5143	UP	Gm15432
ENSMUSG00000086859	6.3662	0.3883	UP	Snhg20
ENSMUSG00000086925	1.542	1.286	UP	Gm6286
ENSMUSG00000087008	1.421	2.3839	UP	Gm5530
ENSMUSG00000087034	2.7345	0.7638	UP	Cbfa2t2-ps1
ENSMUSG00000087075	2.0341	0.2885	UP	Lbhd2
ENSMUSG00000087129	1.4754	1.4852	UP	Gm16316
ENSMUSG00000087153	3.9432	0.7178	UP	Gm6483
ENSMUSG00000087434	1.7534	2.4009	UP	Rab11fip4os1
ENSMUSG00000087534	4.3048	1.2802	UP	Gm11418
ENSMUSG00000087590	2.3836	0.3068	UP	Epb41l4aos
ENSMUSG00000087635	2.6816	0.6453	UP	Gm13414
ENSMUSG00000087701	6.4251	0.9227	UP	Gm13493
ENSMUSG00000089235	1.3637	0.8174	UP	Gm23119
ENSMUSG00000089648	2.2845	1.1252	UP	Gm15790
ENSMUSG00000089670	1.7757	1.4616	UP	Gm16581
ENSMUSG00000089764	2.7741	1.8761	UP	Gm16580
ENSMUSG00000089782	3.0901	0.9094	UP	Btf3-ps1
ENSMUSG00000089838	4.0875	0.9303	UP	Gm2962
ENSMUSG00000089999	4.1198	0.9111	UP	Gm6485
ENSMUSG00000090002	1.9852	0.8043	UP	Gm16006
ENSMUSG00000090021	1.9735	0.8162	UP	Gm6493
ENSMUSG00000090266	1.3293	0.2536	UP	Mettl23
ENSMUSG00000090273	1.3911	0.3835	UP	Prr22
ENSMUSG00000090381	1.5387	1.0876	UP	Gm6158
ENSMUSG00000090516	16.3221	2.4126	UP	Rps11-ps1
ENSMUSG00000090553	2.6116	0.65	UP	Snrpe
ENSMUSG00000090602	5.4051	1.1674	UP	Gm5611
ENSMUSG00000090665	3.485	2.7797	UP	Gad1-ps
ENSMUSG00000090704	8.2632	2.7093	UP	Trp53-ps
ENSMUSG00000091002	2.4051	0.1645	UP	Tcerg1l
ENSMUSG00000091083	1.9543	1.3238	UP	Gm4535
ENSMUSG00000091086	39.8398	0.9861	UP	Rpl6l
ENSMUSG00000091269	7.3557	1.2621	UP	Gm6682
ENSMUSG00000091288	3.017	3.0833	UP	Gm17075
ENSMUSG00000091318	6.524	0.6148	UP	Semp2l1
ENSMUSG00000091475	3.1727	0.217	UP	Cerox1
ENSMUSG00000091509	4.326	0.5106	UP	Gm17066
ENSMUSG00000091561	2.0772	1.5945	UP	Gm6665
ENSMUSG00000091613	2.6482	2.8162	UP	Gm17046
ENSMUSG00000091866	1.4961	1.5185	UP	Gm6257

ENSMUSG00000091900	1.3429	0.8464	UP	Gm4353
ENSMUSG00000091905	3.111	0.4431	UP	Dnajb6-ps
ENSMUSG00000091989	12.9905	0.9768	UP	Ndufab1-ps
ENSMUSG00000092116	1.8102	1.6022	UP	Gm10320
ENSMUSG00000092564	1.5918	0.2843	UP	BC051226
ENSMUSG00000092981	3.9269	0.2223	UP	Mir5125
ENSMUSG00000093384	1.3983	0.8385	UP	Gm20689
ENSMUSG00000093505	1.4598	0.8212	UP	Gm20691
ENSMUSG00000093651	1.5312	0.7734	UP	Gm5873
ENSMUSG00000093674	11.2146	0.4641	UP	Rpl41
ENSMUSG00000093798	3.3394	0.6422	UP	Gm8355
ENSMUSG00000093826	2.604	2.8063	UP	Gm6900
ENSMUSG00000093887	5.3785	1.5131	UP	Gm3033
ENSMUSG00000093898	2.1868	0.91	UP	Gm33933
ENSMUSG00000093909	1.9394	1.5162	UP	Gm3883
ENSMUSG00000094122	1.9506	1.6321	UP	Rpl31-ps9
ENSMUSG00000094320	1.3911	1.16	UP	Chchd2-ps
ENSMUSG00000094344	4.4054	0.9461	UP	Gm11942
ENSMUSG00000094392	2.2803	0.5669	UP	Gm3788
ENSMUSG00000094475	2.3517	1.6033	UP	Gm11007
ENSMUSG00000094497	9.3628	2.141	UP	Gm8210
ENSMUSG00000094530	6.4056	1.1101	UP	Gm21399
ENSMUSG00000094568	2.1547	0.4615	UP	Smarce1-ps1
ENSMUSG00000094974	2.0542	2.1631	UP	Rps19-ps2
ENSMUSG00000095042	1.6507	0.8856	UP	Gm12537
ENSMUSG00000095159	3.5163	0.4923	UP	Tubb4b-ps1
ENSMUSG00000095403	1.4403	0.4112	UP	Gm21092
ENSMUSG00000095588	9.4563	2.2152	UP	Gm12350
ENSMUSG00000095681	2.0409	0.3073	UP	Gm8281
ENSMUSG00000095690	9.4638	0.4445	UP	Rab11b-ps2
ENSMUSG00000095742	18.3356	1.2388	UP	ENSMUSG00000095742
ENSMUSG00000095847	12.0312	1.5	UP	Gm5451
ENSMUSG00000096006	2.6335	1.0315	UP	Gm21596
ENSMUSG00000096140	1.4146	0.4129	UP	Ankrd66
ENSMUSG00000096141	1.626	0.2138	UP	Dnah7a
ENSMUSG00000096361	10.0161	1.8262	UP	Gm5814
ENSMUSG00000096401	2.7209	0.265	UP	Gm21811
ENSMUSG00000096403	1.3413	0.7522	UP	Rnps1-ps
ENSMUSG00000096449	1.97	1.3567	UP	Gm4076
ENSMUSG00000096474	3.0372	2.2382	UP	Gm5561
ENSMUSG00000096544	1.7426	0.6849	UP	Gm4617
ENSMUSG00000096617	7.6529	0.4697	UP	Gm5559
ENSMUSG00000096647	1.5499	1.5601	UP	Tmem41b-ps
ENSMUSG00000096712	3.01	1.7276	UP	Gm15454
ENSMUSG00000096753	2.0846	0.3151	UP	Fam181a

ENSMUSG00000096810	5.2483	2.0256	UP	Gm10481
ENSMUSG00000096842	3.5206	2.0413	UP	Gm10736
ENSMUSG00000096942	4.5429	0.7477	UP	Rps19-ps6
ENSMUSG00000097148	5.172	1.9124	UP	Gm3839
ENSMUSG00000097180	3.7875	0.695	UP	2700038G22Rik
ENSMUSG00000097245	16.9512	1.7407	UP	Gm5421
ENSMUSG00000097388	7.0333	2.9922	UP	Gm3200
ENSMUSG00000097464	1.4113	0.6044	UP	Gm26736
ENSMUSG00000097853	1.3705	1.7975	UP	Gm3532
ENSMUSG00000097877	2.1483	0.3465	UP	Gm26703
ENSMUSG00000097961	1.783	0.528	UP	Gm27000
ENSMUSG00000097974	1.3553	0.2878	UP	Gm10605
ENSMUSG00000097979	9.1621	1.92	UP	Gm4691
ENSMUSG00000097989	1.5428	1.7824	UP	Gm4335
ENSMUSG00000098019	4.4435	3.2428	UP	Gm2546
ENSMUSG00000098104	1.6397	1.3645	UP	Gm6085
ENSMUSG00000098240	1.4931	2.1534	UP	Gm4575
ENSMUSG00000098449	1.4974	0.5472	UP	Gm7467
ENSMUSG00000098915	5.8604	2.683	UP	Rpl15-ps2
ENSMUSG00000098985	1.7136	0.7242	UP	Gm27219
ENSMUSG00000099471	6.6947	1.265	UP	Gm8451
ENSMUSG00000099492	9.0082	3.9221	UP	Gm5525
ENSMUSG00000099779	7.0751	2.5739	UP	Gm8228
ENSMUSG00000099881	1.3528	0.14	UP	2810013P06Rik
ENSMUSG00000099902	5.8106	2.2957	UP	Gm12115
ENSMUSG00000100104	3.4444	2.1255	UP	Gm5644
ENSMUSG00000100131	11.8254	0.9459	UP	Gm28439
ENSMUSG00000100153	2.2761	0.4434	UP	Ppp1ccb
ENSMUSG00000100215	8.6753	2.3329	UP	Gm8292
ENSMUSG00000100555	1.3034	1.8807	UP	Gm8173
ENSMUSG00000100794	3.2961	2.7122	UP	Gm29667
ENSMUSG00000100826	11.9336	0.3014	UP	Snhg14
ENSMUSG00000100862	13.3451	1.0259	UP	Gm10925
ENSMUSG00000100863	3.0924	1.2652	UP	Gm12669
ENSMUSG00000101111	12.9293	1.1607	UP	Gm28437
ENSMUSG00000101249	19.6432	1.4317	UP	Gm29216
ENSMUSG00000101309	3.0382	2.3535	UP	Gm29397
ENSMUSG00000101316	4.0693	0.4877	UP	Gm12663
ENSMUSG00000101389	1.5866	1.9527	UP	Ms4a4a
ENSMUSG00000101431	1.4686	0.6699	UP	Gm7901
ENSMUSG00000101589	10.8406	2.4786	UP	Rbm6-ps1
ENSMUSG00000101795	5.4729	2.2456	UP	Gm5835
ENSMUSG00000101841	2.5904	0.4272	UP	Gm11993
ENSMUSG00000101939	9.3971	1.4889	UP	Gm28438
ENSMUSG00000102038	1.4787	0.9677	UP	Gm12345

ENSMUSG00000102070	5.7546	1.2406	UP	Gm28661
ENSMUSG00000102117	1.8767	2.0883	UP	Rpsa-ps1
ENSMUSG00000102145	2.2359	1.6332	UP	Gm38056
ENSMUSG00000102250	1.7929	2.5459	UP	Gm38260
ENSMUSG00000102275	1.7842	0.7909	UP	Gm37144
ENSMUSG00000102321	1.8156	0.6573	UP	Gm37792
ENSMUSG00000102331	3.7789	0.5475	UP	Gm19938
ENSMUSG00000102386	6.5381	0.819	UP	2900022M07Rik
ENSMUSG00000102478	3.7314	2.0136	UP	BC085271
ENSMUSG00000102494	1.6774	1.305	UP	Gm36988
ENSMUSG00000102657	1.5147	0.9941	UP	Gm37899
ENSMUSG00000102747	3.4963	1.8086	UP	Gm37602
ENSMUSG00000102824	7.3014	0.5163	UP	Pdcd5-ps
ENSMUSG00000102827	2.3017	1.0739	UP	Gm8242
ENSMUSG00000102865	8.273	0.6956	UP	Gm9839
ENSMUSG00000103206	1.578	2.0867	UP	Gm8515
ENSMUSG00000103544	1.5216	2.3301	UP	Gm10048
ENSMUSG00000103546	1.9702	1.6189	UP	Gm37666
ENSMUSG00000103653	19.913	0.8674	UP	Gstp-ps
ENSMUSG00000103922	6.7432	1.0882	UP	Gm6123
ENSMUSG00000104126	2.9731	1.8208	UP	Gm37486
ENSMUSG00000104178	1.5916	0.8826	UP	Gm9916
ENSMUSG00000104222	3.9056	0.656	UP	Gm7292
ENSMUSG00000104257	1.7579	1.5391	UP	Gm20172
ENSMUSG00000104496	4.9763	1.4566	UP	Gm5837
ENSMUSG00000104649	1.5379	1.5701	UP	Gm43712
ENSMUSG00000104699	1.4899	1.3162	UP	Rps4x-ps
ENSMUSG00000104802	8.8427	3.0135	UP	Gm5869
ENSMUSG00000104913	11.4787	0.6022	UP	Gm6560
ENSMUSG00000105233	1.5882	2.6029	UP	Gm20568
ENSMUSG00000105243	2.9746	2.567	UP	Gm43444
ENSMUSG00000105359	5.6133	1.9168	UP	Rpl21-ps10
ENSMUSG00000105558	4.973	2.9759	UP	Gm5855
ENSMUSG00000105687	4.9264	1.3204	UP	Gm6157
ENSMUSG00000105814	19.1269	4.0016	UP	Mir703
ENSMUSG00000105879	3.1464	0.9646	UP	Gm6204
ENSMUSG00000105939	1.3318	1.699	UP	Gm43322
ENSMUSG00000106037	18.4886	1.0366	UP	Gm4332
ENSMUSG00000106379	2.6746	0.1632	UP	Lhfpl3
ENSMUSG00000106408	6.6513	1.0275	UP	Gm43321
ENSMUSG00000106574	5.8809	1.8934	UP	Gm2451
ENSMUSG00000106755	12.7083	0.9075	UP	Tpi-rs11
ENSMUSG00000106826	2.3516	1.3826	UP	Gm42583
ENSMUSG00000106917	2.3681	2.5582	UP	Gm7832
ENSMUSG00000106926	4.5005	2.685	UP	Rpl7-ps7

ENSMUSG00000106988	2.0266	1.1845	UP	Tsg101-ps
ENSMUSG00000107002	3.7314	0.2444	UP	0610012G03Rik
ENSMUSG00000107096	1.4806	0.1515	UP	Gm43597
ENSMUSG00000107145	2.7286	1.3807	UP	Gm43442
ENSMUSG00000107261	2.8411	2.4263	UP	Trmt112-ps1
ENSMUSG00000107280	1.3084	1.1608	UP	Potefam3c
ENSMUSG00000107369	10.3554	1.1682	UP	Gstm2-ps1
ENSMUSG00000107383	9.7593	0.5227	UP	Gm4366
ENSMUSG00000107470	1.4703	0.395	UP	Gm3375
ENSMUSG00000107512	2.8643	0.5478	UP	Gm44433
ENSMUSG00000107707	2.0158	1.7487	UP	Gm44286
ENSMUSG00000107747	11.2396	1.2523	UP	Gm5881
ENSMUSG00000107951	3.0883	1.3918	UP	Gm6210
ENSMUSG00000107996	1.8877	0.606	UP	Gm44228
ENSMUSG00000108231	7.8241	3.2836	UP	Gm4045
ENSMUSG00000108264	7.4054	2.0643	UP	Gm20371
ENSMUSG00000108314	2.6082	0.4185	UP	Prkcz2
ENSMUSG00000108366	2.4296	2.7781	UP	Gm5586
ENSMUSG00000108381	3.003	1.4328	UP	Gm9299
ENSMUSG00000108702	2.3127	0.8126	UP	Gm9333
ENSMUSG00000108772	1.696	1.2836	UP	Gm6063
ENSMUSG00000108799	6.4907	1.1075	UP	Glud-ps
ENSMUSG00000108823	2.6922	2.0157	UP	Gm18959
ENSMUSG00000108852	2.2909	0.3901	UP	Gm44911
ENSMUSG00000108880	1.9477	1.0433	UP	Gm44560
ENSMUSG00000108886	3.8939	1.276	UP	Gm44830
ENSMUSG00000108918	2.7557	2.5351	UP	Gm44802
ENSMUSG00000109082	7.9459	1.3355	UP	Gm44586
ENSMUSG00000109095	3.5048	0.7659	UP	Gm44799
ENSMUSG00000109125	1.6812	1.3161	UP	Gm45159
ENSMUSG00000109222	1.6927	0.2637	UP	Gm10297
ENSMUSG00000109472	2.3448	2.0885	UP	Gm18943
ENSMUSG00000109481	2.5329	1.1406	UP	Gm45130
ENSMUSG00000109509	29.459	2.3757	UP	Rps12-ps4
ENSMUSG00000109536	37.0388	0.6037	UP	9330162G02Rik
ENSMUSG00000109551	3.52	2.1191	UP	4930435N07Rik
ENSMUSG00000109556	1.4143	1.5105	UP	Gm38843
ENSMUSG00000109609	1.8534	0.8	UP	Gm4972
ENSMUSG00000109724	4.3876	1.0983	UP	Gm18194
ENSMUSG00000109894	2.0198	2.1157	UP	Gm5904
ENSMUSG00000109933	3.6209	2.7373	UP	Gm7600
ENSMUSG00000110050	4.1831	3.3293	UP	Gm45259
ENSMUSG00000110057	5.8573	1.7879	UP	Gm2225
ENSMUSG00000110080	2.5036	0.1656	UP	Gm6145
ENSMUSG00000110126	4.9154	1.9318	UP	Gm9347

ENSMUSG00000110275	5.555	1.3869	UP	Gm5905
ENSMUSG00000110331	11.5307	0.5747	UP	Nudc-ps1
ENSMUSG00000110532	2.0235	2.3145	UP	Gm35857
ENSMUSG00000110644	2.5137	0.9124	UP	Gm7390
ENSMUSG00000110679	4.7978	1.4848	UP	Rpl10-ps5
ENSMUSG00000110726	3.5757	1.6117	UP	Gm18101
ENSMUSG00000110744	2.8432	2.4505	UP	Gm5171
ENSMUSG00000110768	1.61	0.4569	UP	Gm18541
ENSMUSG00000110874	1.5997	2.045	UP	D030045P18Rik
ENSMUSG00000110926	3.0087	1.9654	UP	Gm5917
ENSMUSG00000111197	3.181	0.8635	UP	Gm10608
ENSMUSG00000111380	2.9237	1.9794	UP	D9Wsu149
ENSMUSG00000111548	2.6659	2.8093	UP	Gm8162
ENSMUSG00000111594	5.8484	0.609	UP	Gm3365
ENSMUSG00000111877	1.6898	0.5212	UP	Gm6477
ENSMUSG00000111897	72.9492	2.7946	UP	Gm19810
ENSMUSG00000111942	1.4543	0.326	UP	Gm5182
ENSMUSG00000112160	2.4673	0.3938	UP	BC024063
ENSMUSG00000112515	3.2297	2.6673	UP	Gm4928
ENSMUSG00000112550	2.46	1.7356	UP	Gm6627
ENSMUSG00000112614	2.4625	0.7646	UP	Gm9030
ENSMUSG00000112825	2.2027	0.3941	UP	Gm9118
ENSMUSG00000112830	1.6623	0.8606	UP	Gm47765
ENSMUSG00000112908	4.2561	2.3547	UP	Gm7392
ENSMUSG00000112909	2.5811	2.6547	UP	Gm10120
ENSMUSG00000112926	4.2578	3.0172	UP	Gm7172
ENSMUSG00000113061	1.5081	0.8727	UP	Rps18-ps5
ENSMUSG00000113113	2.6371	2.2863	UP	Gm2614
ENSMUSG00000113275	2.8404	1.9085	UP	Tubb2a-ps2
ENSMUSG00000113521	1.6775	1.1813	UP	Gm29787
ENSMUSG00000113606	8.6762	3.6657	UP	Gm47441
ENSMUSG00000113621	1.4066	0.2669	UP	2900060N12Rik
ENSMUSG00000113690	2.4448	1.6276	UP	Gm36501
ENSMUSG00000113743	2.1391	1.1673	UP	Gm8712
ENSMUSG00000114003	5.8728	0.4138	UP	Gm9616
ENSMUSG00000114251	9.3278	0.6323	UP	Tmed10-ps
ENSMUSG00000114282	1.6527	2.324	UP	5330431K02Rik
ENSMUSG00000114321	3.1445	1.0942	UP	Gm8971
ENSMUSG00000114488	14.7167	2.0963	UP	Gm5802
ENSMUSG00000114500	2.7723	1.498	UP	Gm8983
ENSMUSG00000114571	12.5145	5.0369	UP	Gm35595
ENSMUSG00000114579	5.1796	0.7705	UP	Gm4130
ENSMUSG00000114607	1.576	1.5506	UP	Gm8990
ENSMUSG00000114886	2.7398	2.7015	UP	Gm48432
ENSMUSG00000114951	3.7742	1.4168	UP	Gm20784

ENSMUSG00000114970	4.2347	1.0672	UP	Gm49069
ENSMUSG00000114999	1.3735	0.9294	UP	Gm7962
ENSMUSG00000115160	1.6458	1.9532	UP	Gm6532
ENSMUSG00000115194	2.2105	2.6686	UP	Gm48909
ENSMUSG00000115205	4.6522	2.5437	UP	Gm16374
ENSMUSG00000115276	2.9731	0.6519	UP	9930017N22Rik
ENSMUSG00000115280	5.2913	1.318	UP	Gm7107
ENSMUSG00000115448	1.628	1.0514	UP	Gm21178
ENSMUSG00000115457	1.7565	1.731	UP	Gm2387
ENSMUSG00000115497	3.9678	2.2236	UP	Gm49207
ENSMUSG00000115505	1.3569	1.3296	UP	Gm9247
ENSMUSG00000115591	5.4015	0.7126	UP	Gm31282
ENSMUSG00000115637	4.3298	1.3406	UP	Gm30970
ENSMUSG00000116093	1.9642	1.03	UP	Gm3888
ENSMUSG00000116174	4.472	2.3533	UP	Gm10362
ENSMUSG00000116620	2.4619	1.9046	UP	Gm4828
ENSMUSG00000116652	2.0326	0.317	UP	B830017H08Rik
ENSMUSG00000116757	4.5253	2.4623	UP	Gm4786
ENSMUSG00000116875	4.1697	0.2312	UP	Morf4l1-ps1
ENSMUSG00000116958	3.9198	2.4946	UP	Gm6705
ENSMUSG00000117183	1.7534	0.2475	UP	Gm20008
ENSMUSG00000117187	3.5128	1.4475	UP	Gm4708
ENSMUSG00000117202	1.4505	1.87	UP	Gm9349
ENSMUSG00000117241	1.4071	1.229	UP	Gm9214
ENSMUSG00000117278	2.2182	2.4488	UP	Gm36684
ENSMUSG00000117284	3.1996	0.226	UP	Gm7072
ENSMUSG00000117428	6.6417	2.2974	UP	Gm4833
ENSMUSG00000117458	11.2146	1.4479	UP	Gm6552
ENSMUSG00000117730	2.0844	1.2345	UP	Gm5503
ENSMUSG00000117822	21.7695	2.7183	UP	Eef1a1-ps1
ENSMUSG00000117875	14.6444	2.6325	UP	Gm6789
ENSMUSG00000117924	1.4265	0.2096	UP	Tmem223
ENSMUSG00000117962	2.9209	2.6665	UP	Gm6402
ENSMUSG00000118057	1.9229	1.5463	UP	B020010K11Rik
ENSMUSG00000118161	1.9101	1.343	UP	Rps2-ps8
ENSMUSG00000118252	3.5048	1.8268	UP	Gm5521
ENSMUSG00000118264	2.3516	0.8728	UP	Rps15-ps3
ENSMUSG00000118665	2.7918	0.2629	UP	Lin54
ENSMUSG00000120022	1.7271	1.0043	UP	ENSMUSG00000120022
ENSMUSG00000120082	1.4301	1.1163	UP	ENSMUSG00000120082
ENSMUSG00000120113	1.8436	0.2221	UP	ENSMUSG00000120113
ENSMUSG00000120150	1.3802	1.1009	UP	ENSMUSG00000120150
ENSMUSG00000120232	1.5022	0.5946	UP	ENSMUSG00000120232
ENSMUSG00000120425	5.1773	1.0266	UP	ENSMUSG00000120425
ENSMUSG00000120442	1.3034	0.468	UP	ENSMUSG00000120442

ENSMUSG00000120534	1.5884	1.8966	UP	ENSMUSG00000120534
ENSMUSG00000120630	2.3077	0.4097	UP	ENSMUSG00000120630
ENSMUSG00000120849	2.1693	1.4737	UP	ENSMUSG00000120849
ENSMUSG00000120850	2.535	1.8182	UP	ENSMUSG00000120850
ENSMUSG00000120995	1.3099	0.2937	UP	Gm51425
ENSMUSG00000120999	1.8353	0.2701	UP	ENSMUSG00000120999
ENSMUSG00000121005	8.7444	2.8169	UP	ENSMUSG00000121005
ENSMUSG00000121082	1.5632	0.4245	UP	ENSMUSG00000121082
**ALF: SNI_VS_sham**				
ENSMUSG00000000088	1.7168	-0.6593	DOWN	Cox5a
ENSMUSG00000000399	3.3303	-0.4641	DOWN	Ndufa9
ENSMUSG00000001666	17.0372	-1.1616	DOWN	Ddt
ENSMUSG00000001911	3.4012	-0.4124	DOWN	Nfix
ENSMUSG00000002006	3.3228	-0.235	DOWN	Pdzd4
ENSMUSG00000002393	2.3731	-0.4713	DOWN	Nr2f6
ENSMUSG00000003039	2.0593	-0.1752	DOWN	Fam32a
ENSMUSG00000003518	1.6687	-0.1708	DOWN	Dusp3
ENSMUSG00000004070	2.754	-0.2626	DOWN	Hmox2
ENSMUSG00000004609	2.1145	-0.3637	DOWN	Cd33
ENSMUSG00000004610	2.0545	-0.6083	DOWN	Etfb
ENSMUSG00000005125	1.5166	-0.2264	DOWN	Ndrg1
ENSMUSG00000005469	2.0958	-0.2604	DOWN	Prkaca
ENSMUSG00000005674	1.3345	-0.2724	DOWN	Tomm40l
ENSMUSG00000006299	2.9051	-0.2531	DOWN	Aamp
ENSMUSG00000006638	1.611	-1.388	DOWN	Abhd1
ENSMUSG00000006932	3.1066	-0.1579	DOWN	Ctnnb1
ENSMUSG00000007033	2.119	-0.5281	DOWN	Hspa1l
ENSMUSG00000007476	5.3048	-0.7101	DOWN	Lrrc8a
ENSMUSG00000007721	1.8934	-0.3339	DOWN	Ccdc124
ENSMUSG00000008035	1.488	-0.2767	DOWN	Mid1ip1
ENSMUSG00000009076	2.8588	-0.5106	DOWN	Zmat5
ENSMUSG00000009281	8.622	-0.7203	DOWN	Rarres2
ENSMUSG00000011114	1.4386	-0.2706	DOWN	Tbrg1
ENSMUSG00000013973	3.0256	-0.4552	DOWN	Dedd
ENSMUSG00000015085	2.2974	-0.3511	DOWN	Entpd2
ENSMUSG00000015478	1.561	-0.2743	DOWN	Rnf5
ENSMUSG00000015806	1.8392	-0.3511	DOWN	Qdpr
ENSMUSG00000016356	1.9564	-0.4908	DOWN	Col20a1
ENSMUSG00000016503	3.1532	-0.5115	DOWN	Gtf3a
ENSMUSG00000016559	1.3036	-0.2465	DOWN	H3f3b
ENSMUSG00000017307	1.8176	-0.2987	DOWN	Acot8
ENSMUSG00000017830	1.7803	-0.5354	DOWN	Dhx58
ENSMUSG00000018160	1.3082	-0.1802	DOWN	Med1
ENSMUSG00000018554	2.6583	-0.3736	DOWN	Ybx2
ENSMUSG00000018727	1.8293	-0.4815	DOWN	Cpsf4l

ENSMUSG00000018752	1.3385	-1.252	DOWN	Tnfsfm13
ENSMUSG00000019373	1.5882	-0.3905	DOWN	Cops3
ENSMUSG00000019464	1.6674	-0.5183	DOWN	Ptger1
ENSMUSG00000019836	5.2324	-2.2237	DOWN	Amd-ps4
ENSMUSG00000020018	246.2977	-4.5429	DOWN	Snrpf
ENSMUSG00000020086	5.2334	-0.299	DOWN	Macroh2a2
ENSMUSG00000020205	7.6682	-0.523	DOWN	Phlda1
ENSMUSG00000020224	4.5761	-0.5502	DOWN	Llph
ENSMUSG00000020377	4.2046	-0.9109	DOWN	Ltc4s
ENSMUSG00000020430	2.278	-0.3628	DOWN	Pes1
ENSMUSG00000020544	4.7128	-0.6169	DOWN	Cox11
ENSMUSG00000020705	2.0266	-0.1933	DOWN	Ddx42
ENSMUSG00000020857	2.4491	-1.2969	DOWN	Nme2
ENSMUSG00000020900	1.4443	-0.1502	DOWN	Myh10
ENSMUSG00000020992	3.211	-0.9373	DOWN	4930512B01Rik
ENSMUSG00000021040	65.2903	-5.4056	DOWN	Slirp
ENSMUSG00000021253	3.0617	-0.401	DOWN	Tgfb3
ENSMUSG00000021262	2.0402	-0.1699	DOWN	Evl
ENSMUSG00000021395	1.3663	-0.1434	DOWN	Spin1
ENSMUSG00000022048	6.5535	-0.5535	DOWN	Dpysl2
ENSMUSG00000022185	3.5504	-0.2272	DOWN	Acin1
ENSMUSG00000022194	13.8653	-0.6941	DOWN	Pabpn1
ENSMUSG00000022219	7.8295	-2.2137	DOWN	Cideb
ENSMUSG00000022223	2.5849	-0.3378	DOWN	Sdr39u1
ENSMUSG00000022362	1.3714	-1.3331	DOWN	Gm29394
ENSMUSG00000022415	1.3712	-0.1494	DOWN	Syngr1
ENSMUSG00000022519	1.668	-0.5209	DOWN	Srl
ENSMUSG00000022615	2.3107	-0.9328	DOWN	Tymp
ENSMUSG00000022617	2.6694	-0.8399	DOWN	Chkb
ENSMUSG00000022842	9.3966	-0.501	DOWN	Ece2
ENSMUSG00000022956	41.6603	-1.7339	DOWN	Atp5o
ENSMUSG00000022972	6.5242	-0.4684	DOWN	Cfap298
ENSMUSG00000023861	5.5806	-0.6441	DOWN	Mpc1
ENSMUSG00000024018	2.2769	-0.4426	DOWN	Ccdc167
ENSMUSG00000024019	5.0772	-0.461	DOWN	Cmtr1
ENSMUSG00000024063	2.396	-0.3453	DOWN	Lbh
ENSMUSG00000024121	1.6375	-0.4449	DOWN	Atp6v0c
ENSMUSG00000024160	4.3226	-0.5822	DOWN	Spsb3
ENSMUSG00000024309	1.5444	-0.2819	DOWN	Pfdn6
ENSMUSG00000024397	1.321	-0.414	DOWN	Aif1
ENSMUSG00000024429	1.5547	-0.2193	DOWN	Gnl1
ENSMUSG00000024491	1.6842	-0.2275	DOWN	Rbm27
ENSMUSG00000024570	3.3254	-0.405	DOWN	Rbfa
ENSMUSG00000024742	6.8659	-1.0245	DOWN	Fen1
ENSMUSG00000024854	1.5315	-0.7817	DOWN	Pold4

ENSMUSG00000024959	5.4552	-0.9976	DOWN	Bad
ENSMUSG00000025204	7.6571	-0.8574	DOWN	Ndufb8
ENSMUSG00000025352	1.5608	-0.4476	DOWN	Gdf11
ENSMUSG00000025408	2.3355	-0.3267	DOWN	Ddit3
ENSMUSG00000025505	3.9217	-0.2738	DOWN	Tmem80
ENSMUSG00000025551	2.6478	-0.2996	DOWN	Fgf14
ENSMUSG00000025578	2.1557	-0.2816	DOWN	Cbx8
ENSMUSG00000025739	7.2018	-1.462	DOWN	Gng13
ENSMUSG00000026421	1.5187	-0.206	DOWN	Csrp1
ENSMUSG00000026434	3.9827	-0.3397	DOWN	Nucks1
ENSMUSG00000026500	4.7699	-1.1886	DOWN	Cox20
ENSMUSG00000026956	1.3204	-0.2185	DOWN	Uap1l1
ENSMUSG00000027001	3.8292	-0.5332	DOWN	Dusp19
ENSMUSG00000027245	14.5488	-1.2603	DOWN	Hypk
ENSMUSG00000027246	8.1702	-1.5023	DOWN	Ell3
ENSMUSG00000027253	1.5811	-0.3574	DOWN	Lrp4
ENSMUSG00000027419	2.5579	-0.2014	DOWN	Pcsk2
ENSMUSG00000027434	1.8597	-0.4083	DOWN	Nkx2-2
ENSMUSG00000027573	1.9218	-0.1834	DOWN	Gid8
ENSMUSG00000027637	6.7018	-0.5654	DOWN	Rab5if
ENSMUSG00000027777	19.2568	-1.1016	DOWN	Schip1
ENSMUSG00000027848	1.535	-0.4327	DOWN	Olfml3
ENSMUSG00000027983	2.2868	-0.4767	DOWN	Cyp2u1
ENSMUSG00000028104	7.4185	-0.3949	DOWN	Polr3gl
ENSMUSG00000028243	1.8066	-0.1754	DOWN	Ubxn2b
ENSMUSG00000028466	4.6213	-0.3309	DOWN	Creb3
ENSMUSG00000028618	1.383	-0.3082	DOWN	Tmem59
ENSMUSG00000028701	2.5846	-0.2549	DOWN	Lurap1
ENSMUSG00000028790	1.4714	-0.176	DOWN	Khdrbs1
ENSMUSG00000028850	1.7526	-0.2878	DOWN	Gpatch3
ENSMUSG00000028975	2.2901	-0.7186	DOWN	Pex14
ENSMUSG00000029028	5.7541	-0.341	DOWN	Lrrc47
ENSMUSG00000029426	1.9873	-0.2895	DOWN	Scarb2
ENSMUSG00000029433	2.0047	-0.5103	DOWN	Diablo
ENSMUSG00000029499	2.0419	-0.4102	DOWN	Pxmp2
ENSMUSG00000029570	1.5902	-0.2958	DOWN	Lfng
ENSMUSG00000029713	1.9673	-0.2321	DOWN	Gnb2
ENSMUSG00000029720	1.3468	-0.9286	DOWN	Gm20605
ENSMUSG00000029823	1.5959	-0.2704	DOWN	Luc7l2
ENSMUSG00000029838	1.3055	-0.1552	DOWN	Ptn
ENSMUSG00000029868	4.1775	-0.5438	DOWN	Trpv6
ENSMUSG00000030134	3.6821	-0.1488	DOWN	Rasgef1a
ENSMUSG00000030330	1.9936	-0.646	DOWN	Ing4
ENSMUSG00000030649	3.5504	-0.4191	DOWN	Anapc15
ENSMUSG00000030703	1.4347	-0.4516	DOWN	Gdpd3

ENSMUSG00000030739	1.5471	-0.2123	DOWN	Myh14
ENSMUSG00000030870	4.8287	-0.2778	DOWN	Ubfd1
ENSMUSG00000030890	11.2288	-2.8675	DOWN	Ilk
ENSMUSG00000031144	3.8729	-0.2563	DOWN	Syp
ENSMUSG00000031245	3.1697	-0.4988	DOWN	Hmgn5
ENSMUSG00000031299	6.2898	-0.19	DOWN	Pdha1
ENSMUSG00000031691	1.5372	-0.1995	DOWN	Tnpo2
ENSMUSG00000031732	2.2926	-0.2164	DOWN	Phlpp2
ENSMUSG00000031775	6.1521	-0.638	DOWN	Pllp
ENSMUSG00000032024	1.7526	-0.2518	DOWN	Clmp
ENSMUSG00000032040	3.1532	-0.327	DOWN	Dcps
ENSMUSG00000032050	1.488	-0.1498	DOWN	Rdx
ENSMUSG00000032066	2.2196	-0.5861	DOWN	Bco2
ENSMUSG00000032085	2.1452	-1.5854	DOWN	Tagln
ENSMUSG00000032097	3.0899	-0.3913	DOWN	Ddx6
ENSMUSG00000032215	1.9633	-0.3615	DOWN	Rsl24d1
ENSMUSG00000032666	2.9082	-0.1968	DOWN	1700025G04Rik
ENSMUSG00000032867	3.0276	-0.323	DOWN	Fbxw8
ENSMUSG00000032952	5.0267	-0.779	DOWN	Ap4b1
ENSMUSG00000033029	1.5382	-0.5238	DOWN	1700088E04Rik
ENSMUSG00000033152	8.7253	-0.4866	DOWN	Podxl2
ENSMUSG00000033475	2.7859	-2.0385	DOWN	Tomm6
ENSMUSG00000033565	1.5418	-0.2522	DOWN	Rbfox2
ENSMUSG00000033751	10.9943	-1.0769	DOWN	Gadd45gip1
ENSMUSG00000034042	1.9246	-0.1725	DOWN	Gpbp1l1
ENSMUSG00000034101	3.2123	-0.3241	DOWN	Ctnnd1
ENSMUSG00000034120	1.5541	-0.2022	DOWN	Srsf2
ENSMUSG00000034382	4.7468	-1.2994	DOWN	AI661453
ENSMUSG00000034613	3.7097	-0.2974	DOWN	Ppm1h
ENSMUSG00000034681	1.9218	-0.1491	DOWN	Rnps1
ENSMUSG00000034853	1.4461	-0.2699	DOWN	Acot11
ENSMUSG00000035086	7.0623	-0.2874	DOWN	Becn1
ENSMUSG00000035228	3.3303	-0.3077	DOWN	Ccdc106
ENSMUSG00000035383	1.3182	-0.8415	DOWN	Pmch
ENSMUSG00000035960	16.2819	-0.8473	DOWN	Apex1
ENSMUSG00000036155	1.7099	-0.2089	DOWN	Mgat5
ENSMUSG00000036430	5.5297	-0.5181	DOWN	Tbcc
ENSMUSG00000036560	2.7818	-0.4355	DOWN	Lgi4
ENSMUSG00000036731	3.3702	-0.6251	DOWN	Cysrt1
ENSMUSG00000036918	1.4842	-0.2783	DOWN	Ttc7
ENSMUSG00000037405	1.5218	-0.7625	DOWN	Icam1
ENSMUSG00000037434	2.0026	-0.2522	DOWN	Slc30a1
ENSMUSG00000037905	3.8524	-0.2091	DOWN	Bri3bp
ENSMUSG00000038152	2.5739	-1.3098	DOWN	5033430I15Rik
ENSMUSG00000038349	1.7299	-0.1912	DOWN	Plcl1

ENSMUSG00000038593	2.204	-0.2359	DOWN	Tctn1
ENSMUSG00000038717	16.7581	-0.8534	DOWN	Atp5l
ENSMUSG00000038803	7.5533	-0.6023	DOWN	Ost4
ENSMUSG00000038880	2.0341	-0.381	DOWN	Mrps34
ENSMUSG00000038976	2.4862	-0.2446	DOWN	Ppp1r9b
ENSMUSG00000039108	1.7665	-0.277	DOWN	Lsm14b
ENSMUSG00000039148	1.9898	-0.2174	DOWN	Sart1
ENSMUSG00000039221	6.1768	-0.9891	DOWN	Rpl22l1
ENSMUSG00000039253	1.7803	-0.2089	DOWN	Fn3krp
ENSMUSG00000039382	2.7354	-0.3133	DOWN	Wdr45
ENSMUSG00000039452	3.7974	-0.3157	DOWN	Snx22
ENSMUSG00000039556	5.1515	-0.6931	DOWN	Ppp1r3f
ENSMUSG00000039754	1.3499	-0.3295	DOWN	Alkbh4
ENSMUSG00000039771	2.4417	-0.57	DOWN	Polr2j
ENSMUSG00000040424	4.9489	-0.2506	DOWN	Hipk4
ENSMUSG00000040740	2.07	-0.5216	DOWN	Slc25a34
ENSMUSG00000040838	1.9472	-2.6224	DOWN	Gm11639
ENSMUSG00000040842	5.9838	-0.4614	DOWN	Szrd1
ENSMUSG00000040904	3.6441	-1.8932	DOWN	Gm21988
ENSMUSG00000041263	2.8646	-0.2432	DOWN	Rusc1
ENSMUSG00000041954	2.0774	-0.4858	DOWN	Tnfrsf18
ENSMUSG00000042275	4.5222	-0.6907	DOWN	Pelo
ENSMUSG00000042390	1.9063	-0.1944	DOWN	Gatad2b
ENSMUSG00000042419	1.561	-0.3858	DOWN	Nfkbil1
ENSMUSG00000042532	3.3577	-0.2838	DOWN	Golga7b
ENSMUSG00000042751	3.3796	-0.1818	DOWN	Nmnat2
ENSMUSG00000042831	3.2976	-0.9288	DOWN	Alkbh6
ENSMUSG00000043168	1.371	-0.4386	DOWN	4930426D05Rik
ENSMUSG00000043687	2.6769	-1.0362	DOWN	1190005I06Rik
ENSMUSG00000043962	1.6805	-0.3799	DOWN	Thrap3
ENSMUSG00000044068	2.0112	-0.4256	DOWN	Zrsr1
ENSMUSG00000044147	2.8804	-0.2621	DOWN	Arf6
ENSMUSG00000044795	3.7576	-0.4169	DOWN	Cyb5d1
ENSMUSG00000045934	1.4347	-0.33	DOWN	Mtmr11
ENSMUSG00000046532	1.9189	-0.2526	DOWN	Ar
ENSMUSG00000047379	4.8907	-1.7911	DOWN	B4gat1
ENSMUSG00000047632	1.7168	-0.2459	DOWN	Fgfbp3
ENSMUSG00000047658	2.2173	-0.5828	DOWN	Gal3st3
ENSMUSG00000047822	1.5702	-1.206	DOWN	Angptl8
ENSMUSG00000047988	2.6142	-0.5066	DOWN	4933428G20Rik
ENSMUSG00000048022	2.4021	-0.2411	DOWN	Tmem229a
ENSMUSG00000048096	1.4144	-0.8151	DOWN	Lmod1
ENSMUSG00000048206	3.1035	-2.5188	DOWN	Dnajb8
ENSMUSG00000048481	1.3204	-0.3198	DOWN	Mypop
ENSMUSG00000048483	1.589	-0.2217	DOWN	Zdhhc22

ENSMUSG00000049090	2.3903	-0.1874	DOWN	Zadh2
ENSMUSG00000049422	2.4831	-1.1444	DOWN	Chchd10
ENSMUSG00000049511	1.668	-0.4284	DOWN	Htr1b
ENSMUSG00000050702	1.4559	-1.4689	DOWN	4930563M21Rik
ENSMUSG00000050708	2.797	-0.5237	DOWN	Ftl1
ENSMUSG00000050860	4.6917	-1.684	DOWN	Phospho1
ENSMUSG00000050891	1.8888	-0.3277	DOWN	Tatdn1
ENSMUSG00000050954	3.8524	-0.3933	DOWN	Zfp169
ENSMUSG00000051149	1.4619	-0.7517	DOWN	Adnp
ENSMUSG00000051323	1.4598	-0.2289	DOWN	Pcdh19
ENSMUSG00000051391	5.0636	-0.2077	DOWN	Ywhag
ENSMUSG00000051527	2.003	-0.1746	DOWN	Usp29
ENSMUSG00000051537	2.9211	-0.2406	DOWN	Gm5124
ENSMUSG00000051627	3.7197	-1.1531	DOWN	H1f4
ENSMUSG00000051650	13.6491	-1.2038	DOWN	B3gnt2
ENSMUSG00000052040	2.0047	-0.3378	DOWN	Klf13
ENSMUSG00000052310	2.9689	-0.2259	DOWN	Slc39a1
ENSMUSG00000053093	3.637	-0.6951	DOWN	Myh7
ENSMUSG00000053128	4.1887	-1.2139	DOWN	Rnf26
ENSMUSG00000053192	2.7991	-0.2114	DOWN	Mllt11
ENSMUSG00000053291	10.5675	-1.2161	DOWN	Rab4b
ENSMUSG00000053769	2.0137	-0.4331	DOWN	Lysmd1
ENSMUSG00000053835	2.0658	-0.5621	DOWN	H2-T24
ENSMUSG00000053877	1.5702	-0.5966	DOWN	Srcap
ENSMUSG00000054034	3.3872	-0.6274	DOWN	Tceal5
ENSMUSG00000054256	2.7397	-0.2396	DOWN	Msi1
ENSMUSG00000054934	17.457	-1.414	DOWN	Kcnmb4
ENSMUSG00000055302	4.6224	-0.3658	DOWN	Mrfap1
ENSMUSG00000056144	1.589	-0.4213	DOWN	Trim34a
ENSMUSG00000056486	1.5165	-0.158	DOWN	Chn1
ENSMUSG00000056501	2.0932	-0.7588	DOWN	Cebpb
ENSMUSG00000056508	3.0901	-0.4295	DOWN	1700001K19Rik
ENSMUSG00000057137	1.449	-0.6231	DOWN	Tmem140
ENSMUSG00000057605	2.1948	-1.0477	DOWN	Gm6807
ENSMUSG00000057788	2.0459	-0.2414	DOWN	Ddx49
ENSMUSG00000058281	2.277	-1.2948	DOWN	Gm10038
ENSMUSG00000058546	9.3112	-1.5455	DOWN	Rpl23a
ENSMUSG00000058600	1.6951	-0.6059	DOWN	Rpl30
ENSMUSG00000059040	1.4436	-0.6886	DOWN	Eno1b
ENSMUSG00000059278	6.4287	-0.5358	DOWN	Naa38
ENSMUSG00000060538	5.5367	-0.5089	DOWN	Tmem219
ENSMUSG00000061099	3.7197	-0.7288	DOWN	Gapdhs
ENSMUSG00000061118	4.0705	-0.4142	DOWN	Dnajc30
ENSMUSG00000061718	1.5436	-0.3643	DOWN	Ppp1r1b
ENSMUSG00000062081	2.5899	-1.1091	DOWN	Gm6055

ENSMUSG00000062270	1.9333	-0.3058	DOWN	Morf4l1
ENSMUSG00000062661	3.7235	-0.1684	DOWN	Ncs1
ENSMUSG00000063235	2.2453	-0.5186	DOWN	Ptpmt1
ENSMUSG00000063556	3.4101	-0.7513	DOWN	Gm10132
ENSMUSG00000063856	1.488	-0.4813	DOWN	Gpx1
ENSMUSG00000064247	1.488	-0.2252	DOWN	Plcxd1
ENSMUSG00000064356	3.8347	-3.1097	DOWN	mt-Atp8
ENSMUSG00000064585	5.0636	-1.0332	DOWN	Gm25129
ENSMUSG00000064871	2.5526	-1.2229	DOWN	Snord58b
ENSMUSG00000065431	1.6166	-1.157	DOWN	Mir186
ENSMUSG00000065470	2.012	-1.1494	DOWN	Mir149
ENSMUSG00000065485	4.4282	-2.1073	DOWN	Mir219a-2
ENSMUSG00000065521	3.3172	-3.4556	DOWN	Mir296
ENSMUSG00000065524	2.6097	-1.0381	DOWN	Mir135a-2
ENSMUSG00000065530	1.727	-1.5693	DOWN	Mir99a
ENSMUSG00000065637	1.9853	-1.0581	DOWN	Gm26397
ENSMUSG00000065676	1.5145	-1.0436	DOWN	Snord42b
ENSMUSG00000066026	1.3784	-0.4047	DOWN	Dhrs3
ENSMUSG00000067547	7.6861	-1.0949	DOWN	Gm7666
ENSMUSG00000067653	4.3844	-1.1918	DOWN	Ankrd23
ENSMUSG00000067713	4.2269	-0.4465	DOWN	Prkag1
ENSMUSG00000067771	3.6238	-1.7667	DOWN	Pwwp4a
ENSMUSG00000068099	3.1606	-0.3767	DOWN	Smim45
ENSMUSG00000068206	3.5576	-0.3188	DOWN	Pick1
ENSMUSG00000068823	2.9336	-0.2479	DOWN	Csde1
ENSMUSG00000069014	7.9343	-1.4031	DOWN	Gm5641
ENSMUSG00000070498	1.8191	-0.2057	DOWN	Tmem132b
ENSMUSG00000071014	2.1387	-0.4444	DOWN	Ndufb6
ENSMUSG00000071076	8.5551	-0.7174	DOWN	Jund
ENSMUSG00000071265	2.4565	-0.3954	DOWN	1700086L19Rik
ENSMUSG00000071653	1.9385	-1.5353	DOWN	1810009A15Rik
ENSMUSG00000071757	1.4842	-0.2804	DOWN	Zhx2
ENSMUSG00000072494	2.5395	-0.3029	DOWN	Ppp1r3e
ENSMUSG00000072772	6.7407	-0.6129	DOWN	Grcc10
ENSMUSG00000073096	3.0897	-0.3843	DOWN	Lrrc61
ENSMUSG00000073460	1.5394	-0.5045	DOWN	Pnldc1
ENSMUSG00000073639	4.2711	-0.2116	DOWN	Rab18
ENSMUSG00000073755	2.2933	-0.2394	DOWN	5730409E04Rik
ENSMUSG00000074129	3.2217	-0.3005	DOWN	Rpl13a
ENSMUSG00000074346	1.3825	-0.5496	DOWN	Kcnd3os
ENSMUSG00000074513	3.6559	-0.4385	DOWN	Arfip1
ENSMUSG00000074643	1.3832	-1.1665	DOWN	Cpne1
ENSMUSG00000074800	9.3791	-1.7255	DOWN	Gm4149
ENSMUSG00000074884	28.5308	-1.1624	DOWN	Serf2
ENSMUSG00000075585	1.7803	-0.2679	DOWN	6330403L08Rik

ENSMUSG00000076269	5.9834	-2.2012	DOWN	Mir374b
ENSMUSG00000077450	1.385	-0.2594	DOWN	Rab11b
ENSMUSG00000077711	1.9333	-1.3057	DOWN	AF357399
ENSMUSG00000078566	2.5088	-0.4489	DOWN	Bnip3
ENSMUSG00000078580	6.0638	-0.4399	DOWN	E430018J23Rik
ENSMUSG00000078630	1.8561	-1.312	DOWN	Tomt
ENSMUSG00000078656	1.726	-1.188	DOWN	Vps25
ENSMUSG00000079019	1.7168	-1.2226	DOWN	Insl3
ENSMUSG00000079426	2.9657	-0.3046	DOWN	Arpc4
ENSMUSG00000079484	5.5929	-0.6732	DOWN	Phyhd1
ENSMUSG00000079564	1.8234	-0.8525	DOWN	Gm11149
ENSMUSG00000080268	1.606	-1.0412	DOWN	Brms1
ENSMUSG00000080364	1.7322	-0.9774	DOWN	Gm25777
ENSMUSG00000080747	1.4702	-2.4745	DOWN	Gm14016
ENSMUSG00000081382	2.754	-1.26	DOWN	Rpl18-ps1
ENSMUSG00000081455	3.1838	-1.8927	DOWN	Hmgb1-ps3
ENSMUSG00000081603	1.5993	-0.5963	DOWN	Gm14681
ENSMUSG00000081738	2.38	-1.2566	DOWN	Hmgb1-ps2
ENSMUSG00000081752	5.7604	-0.5795	DOWN	Sms-ps
ENSMUSG00000082144	3.2313	-0.9378	DOWN	Gm12788
ENSMUSG00000082329	1.3147	-0.8513	DOWN	Gm14287
ENSMUSG00000082383	3.5889	-1.7155	DOWN	Gm9670
ENSMUSG00000082585	12.2187	-1.5793	DOWN	Gm15387
ENSMUSG00000083307	3.6394	-0.4305	DOWN	AA414768
ENSMUSG00000083512	1.9645	-1.1895	DOWN	Gm12749
ENSMUSG00000083744	1.3932	-0.9368	DOWN	Gm14824
ENSMUSG00000084106	1.3783	-0.3439	DOWN	Gm6136
ENSMUSG00000084407	2.7645	-0.8643	DOWN	Gm14018
ENSMUSG00000084808	1.7276	-0.5838	DOWN	9430091E24Rik
ENSMUSG00000084880	5.9459	-0.9181	DOWN	Tomm6os
ENSMUSG00000084885	2.463	-0.586	DOWN	3010001F23Rik
ENSMUSG00000085071	2.2301	-0.5041	DOWN	Gm14066
ENSMUSG00000085151	3.7309	-0.6892	DOWN	1110018N20Rik
ENSMUSG00000085181	1.6925	-0.5657	DOWN	Gm12709
ENSMUSG00000085227	1.908	-0.6955	DOWN	6330418K02Rik
ENSMUSG00000085403	1.611	-1.0253	DOWN	Gm13068
ENSMUSG00000085442	14.959	-2.1848	DOWN	Gm3362
ENSMUSG00000085767	2.5465	-0.4251	DOWN	Gm13563
ENSMUSG00000085828	2.6786	-0.4612	DOWN	Gm15612
ENSMUSG00000085830	2.505	-0.4526	DOWN	Grin1os
ENSMUSG00000085923	1.3298	-0.7265	DOWN	Gm12781
ENSMUSG00000086067	2.6835	-0.8072	DOWN	Gm16183
ENSMUSG00000086119	9.8298	-0.6147	DOWN	Gm2415
ENSMUSG00000086193	3.1532	-0.5949	DOWN	Gm11508
ENSMUSG00000086268	1.6703	-1.2776	DOWN	Gm11670

ENSMUSG00000086288	2.3297	-1.0998	DOWN	Gm15265
ENSMUSG00000086328	1.4717	-1.1887	DOWN	2700033N17Rik
ENSMUSG00000086361	1.3667	-0.6104	DOWN	Gm6569
ENSMUSG00000086716	1.3468	-1.3839	DOWN	Gm11629
ENSMUSG00000086741	1.9192	-0.7159	DOWN	Gm15816
ENSMUSG00000086754	1.3211	-1.3247	DOWN	Gm16098
ENSMUSG00000086769	1.408	-0.6288	DOWN	Gm15587
ENSMUSG00000086784	1.3111	-0.4219	DOWN	Isoc2a
ENSMUSG00000086844	1.5112	-0.4492	DOWN	B230206H07Rik
ENSMUSG00000086905	3.5169	-0.9411	DOWN	Gm13716
ENSMUSG00000086918	1.359	-0.6001	DOWN	4930429F24Rik
ENSMUSG00000086921	1.5643	-1.0045	DOWN	Gm13189
ENSMUSG00000086930	1.4912	-0.5902	DOWN	Frs3os
ENSMUSG00000087026	1.8066	-0.3718	DOWN	A230103J11Rik
ENSMUSG00000087165	2.3215	-0.4	DOWN	2010001A14Rik
ENSMUSG00000087178	3.1579	-0.4501	DOWN	A230056P14Rik
ENSMUSG00000087249	1.4874	-0.8961	DOWN	Gm16062
ENSMUSG00000087334	1.6798	-0.5641	DOWN	AW495222
ENSMUSG00000087381	1.8678	-0.6382	DOWN	Gm16008
ENSMUSG00000087610	1.5259	-0.4947	DOWN	Gm16253
ENSMUSG00000087658	3.0205	-1.3125	DOWN	Hotairm1
ENSMUSG00000087667	1.3227	-1.7233	DOWN	Gm13381
ENSMUSG00000087672	5.5696	-0.8746	DOWN	Gm15122
ENSMUSG00000088054	2.7435	-1.4304	DOWN	Mir1968
ENSMUSG00000088378	2.2416	-2.2996	DOWN	Gm25184
ENSMUSG00000088901	2.2197	-1.7935	DOWN	Mir1943
ENSMUSG00000088982	1.3684	-1.815	DOWN	Gm25600
ENSMUSG00000089026	1.4722	-1.5418	DOWN	Gm25014
ENSMUSG00000089929	2.4179	-2.0549	DOWN	Bcl2a1b
ENSMUSG00000090087	1.6944	-2.1853	DOWN	Gm15939
ENSMUSG00000090113	1.3938	-0.7044	DOWN	Nhlrc4
ENSMUSG00000090150	1.7133	-0.3013	DOWN	Acad11
ENSMUSG00000090327	3.909	-0.7574	DOWN	Gm17111
ENSMUSG00000090582	2.1177	-1.3286	DOWN	Gm17024
ENSMUSG00000090589	1.4661	-0.8601	DOWN	Gm17180
ENSMUSG00000090625	2.3876	-1.4086	DOWN	Gm20721
ENSMUSG00000090761	2.2238	-0.4602	DOWN	Gm17201
ENSMUSG00000091021	6.1046	-1.5655	DOWN	Gm17300
ENSMUSG00000091102	2.7818	-0.9244	DOWN	5830462I19Rik
ENSMUSG00000091223	4.1558	-0.8805	DOWN	Gm8775
ENSMUSG00000091227	1.3792	-0.885	DOWN	Gm3755
ENSMUSG00000091272	1.6271	-0.9937	DOWN	Gm17641
ENSMUSG00000091803	37.2766	-1.433	DOWN	Cox16
ENSMUSG00000091811	2.6065	-0.3356	DOWN	Inafm1
ENSMUSG00000091906	2.2261	-2.3518	DOWN	1700099I09Rik

ENSMUSG00000092086	4.2214	-1.1763	DOWN	Gm6793
ENSMUSG00000092216	3.3956	-0.914	DOWN	Gm19345
ENSMUSG00000092375	5.3983	-1.0116	DOWN	A730060N03Rik
ENSMUSG00000092470	2.2056	-0.9116	DOWN	Gm20518
ENSMUSG00000092515	1.729	-2.0192	DOWN	C87198
ENSMUSG00000092595	9.0809	-3.04	DOWN	Gm20427
ENSMUSG00000092659	6.0588	-4.0983	DOWN	Mir3100
ENSMUSG00000092827	3.9827	-1.2071	DOWN	Mir3091
ENSMUSG00000093351	5.0772	-1.3461	DOWN	Mir3072
ENSMUSG00000093629	1.7168	-0.6939	DOWN	Prox2os
ENSMUSG00000093686	16.1419	-2.9183	DOWN	Gm4705
ENSMUSG00000095334	3.6203	-1.4468	DOWN	Gm21984
ENSMUSG00000096145	2.6712	-0.6942	DOWN	Vkorc1
ENSMUSG00000096210	1.5547	-0.3633	DOWN	H1f0
ENSMUSG00000096962	2.012	-1.8228	DOWN	Gm26622
ENSMUSG00000097187	3.2217	-0.7427	DOWN	Gm19426
ENSMUSG00000097204	3.0722	-0.6128	DOWN	Gm17690
ENSMUSG00000097239	1.7299	-1.4707	DOWN	Gm27029
ENSMUSG00000097320	4.8438	-1.014	DOWN	Tmem147os
ENSMUSG00000097343	1.8058	-0.4817	DOWN	9030407P20Rik
ENSMUSG00000097431	2.4565	-0.2741	DOWN	Gm26782
ENSMUSG00000097433	1.827	-0.6524	DOWN	Gm26781
ENSMUSG00000097748	1.3023	-1.1299	DOWN	Gm26533
ENSMUSG00000097788	7.7326	-1.6312	DOWN	Gm16596
ENSMUSG00000097929	1.6013	-0.1799	DOWN	Tunar
ENSMUSG00000098004	1.8255	-1.5302	DOWN	Gm27027
ENSMUSG00000098196	2.9899	-1.3281	DOWN	Gm26964
ENSMUSG00000098259	5.6356	-1.114	DOWN	Gm27616
ENSMUSG00000098269	1.502	-1.0796	DOWN	Mir8094
ENSMUSG00000098327	1.4643	-1.2719	DOWN	Mir7087
ENSMUSG00000098332	2.3104	-0.4677	DOWN	Pigbos1
ENSMUSG00000098620	1.7433	-0.5179	DOWN	Gm27209
ENSMUSG00000098661	1.8438	-1.1986	DOWN	Mir7052
ENSMUSG00000098893	3.3254	-1.5435	DOWN	Gm17828
ENSMUSG00000099272	1.6805	-2.4397	DOWN	Mir7093
ENSMUSG00000099284	1.895	-1.0559	DOWN	Mir7026
ENSMUSG00000099384	2.0976	-0.4872	DOWN	1700110C19Rik
ENSMUSG00000100552	1.52	-1.2806	DOWN	Gm29019
ENSMUSG00000100954	1.5608	-0.4447	DOWN	Gm10138
ENSMUSG00000101133	2.3087	-1.7575	DOWN	Gm29050
ENSMUSG00000101320	3.1102	-1.2609	DOWN	Gm28529
ENSMUSG00000101693	2.0545	-0.6266	DOWN	Gm19461
ENSMUSG00000101792	1.7526	-1.138	DOWN	Gm28447
ENSMUSG00000102144	1.63	-1.5965	DOWN	4930429P21Rik
ENSMUSG00000102545	2.0458	-0.7525	DOWN	6430573P05Rik

ENSMUSG00000103039	1.9323	-0.8341	DOWN	Gm37123
ENSMUSG00000104293	2.2469	-1.3255	DOWN	Gm38043
ENSMUSG00000104369	4.3978	-1.9912	DOWN	Gm38082
ENSMUSG00000104516	1.6856	-0.7431	DOWN	Gm9884
ENSMUSG00000104877	1.4643	-2.3604	DOWN	Gm42651
ENSMUSG00000105881	1.8656	-0.5516	DOWN	4932422M17Rik
ENSMUSG00000105922	2.2172	-0.9885	DOWN	Gm42769
ENSMUSG00000105969	4.5481	-1.8879	DOWN	Gm42525
ENSMUSG00000106692	1.6434	-1.2078	DOWN	Gm36447
ENSMUSG00000106967	1.3832	-0.8591	DOWN	Gm42477
ENSMUSG00000107068	22.1416	-2.5725	DOWN	Gm42742
ENSMUSG00000107927	2.1121	-1.0103	DOWN	Gm44090
ENSMUSG00000107944	1.794	-0.816	DOWN	Gm44280
ENSMUSG00000108070	1.8073	-1.3599	DOWN	Gm43872
ENSMUSG00000108320	3.4012	-1.3925	DOWN	Gm44877
ENSMUSG00000108378	1.8385	-1.6566	DOWN	Gm44641
ENSMUSG00000108452	12.9324	-2.0269	DOWN	4930413G21Rik
ENSMUSG00000109080	1.8742	-1.4532	DOWN	Gm38944
ENSMUSG00000109147	2.1606	-0.5333	DOWN	4930431P19Rik
ENSMUSG00000109205	1.535	-0.9045	DOWN	Gm44954
ENSMUSG00000109274	4.3763	-0.7015	DOWN	Gm45133
ENSMUSG00000109362	1.4643	-0.3576	DOWN	Gm44562
ENSMUSG00000109460	1.5204	-1.1354	DOWN	Gm45591
ENSMUSG00000110230	1.9923	-2.9061	DOWN	Gm45846
ENSMUSG00000111094	1.3503	-1.4776	DOWN	Gm34425
ENSMUSG00000111143	2.0201	-1.8523	DOWN	Gm47134
ENSMUSG00000111291	1.3034	-0.391	DOWN	Gm48604
ENSMUSG00000111793	1.5048	-0.5464	DOWN	Gm47950
ENSMUSG00000112135	3.0402	-1.7246	DOWN	Gm9029
ENSMUSG00000112201	2.5349	-1.1948	DOWN	4930532I03Rik
ENSMUSG00000112393	1.9192	-1.3129	DOWN	Gm48655
ENSMUSG00000112593	1.4966	-1.6993	DOWN	Gm48882
ENSMUSG00000112891	1.5702	-1.4619	DOWN	Gm47391
ENSMUSG00000112941	11.3583	-1.2938	DOWN	Gm48623
ENSMUSG00000113052	1.5463	-0.6464	DOWN	Gm31513
ENSMUSG00000113617	1.4702	-1.9251	DOWN	Gm8655
ENSMUSG00000114014	5.0643	-0.8198	DOWN	Gm48350
ENSMUSG00000114246	2.8336	-0.6459	DOWN	Gm48603
ENSMUSG00000114540	1.4963	-0.7283	DOWN	Gm6421
ENSMUSG00000114835	2.4565	-0.9032	DOWN	Gm48194
ENSMUSG00000115003	1.7803	-1.058	DOWN	Gm48933
ENSMUSG00000115005	2.7961	-1.5524	DOWN	Gm49195
ENSMUSG00000115138	1.3241	-0.3341	DOWN	Gm36899
ENSMUSG00000115410	1.3483	-2.1461	DOWN	2810457G06Rik
ENSMUSG00000115958	2.3002	-1.7359	DOWN	Gm28040

ENSMUSG00000116038	4.8917	-1.3624	DOWN	Gm46563
ENSMUSG00000116860	1.3144	-1.4519	DOWN	Gm46593
ENSMUSG00000117028	1.7553	-1.8952	DOWN	Gm49874
ENSMUSG00000117232	1.6016	-1.7424	DOWN	1600002D24Rik
ENSMUSG00000117310	10.7448	-1.6609	DOWN	Ptp4a1
ENSMUSG00000117375	1.7512	-1.0082	DOWN	Gm33373
ENSMUSG00000117421	2.098	-1.4784	DOWN	Gm41639
ENSMUSG00000117710	4.8769	-0.8614	DOWN	Gm10817
ENSMUSG00000117789	1.3254	-1.8363	DOWN	Gm50388
ENSMUSG00000117819	7.0343	-1.9729	DOWN	Gm50253
ENSMUSG00000117942	3.0858	-1.1176	DOWN	Maskbp3
ENSMUSG00000117973	1.5835	-2.7632	DOWN	Gm50140
ENSMUSG00000118502	3.485	-0.5543	DOWN	Gm52964
ENSMUSG00000118504	5.0772	-0.8942	DOWN	Gm53012
ENSMUSG00000118526	2.1585	-1.3816	DOWN	Gm52960
ENSMUSG00000119104	2.7471	-1.8969	DOWN	Gm25632
ENSMUSG00000119223	1.481	-0.9603	DOWN	Mir3070b
ENSMUSG00000119442	2.8774	-1.242	DOWN	Gm23966
ENSMUSG00000119873	1.3565	-1.1127	DOWN	Gm24497
ENSMUSG00000119906	3.1442	-2.9677	DOWN	Gm22887
ENSMUSG00000119987	1.3027	-1.758	DOWN	ENSMUSG00000119987
ENSMUSG00000120261	1.5966	-1.7903	DOWN	ENSMUSG00000120261
ENSMUSG00000120483	1.413	-0.4765	DOWN	ENSMUSG00000120483
ENSMUSG00000120615	5.3048	-1.8697	DOWN	ENSMUSG00000120615
ENSMUSG00000120681	9.1836	-0.7664	DOWN	ENSMUSG00000120681
ENSMUSG00000120806	2.2084	-1.3986	DOWN	ENSMUSG00000120806
ENSMUSG00000120819	2.1666	-1.3186	DOWN	ENSMUSG00000120819
ENSMUSG00000120846	1.3254	-1.4437	DOWN	ENSMUSG00000120846
ENSMUSG00000120857	1.406	-1.4889	DOWN	ENSMUSG00000120857
ENSMUSG00000121052	4.5991	-2.6523	DOWN	ENSMUSG00000121052
ENSMUSG00000121054	1.5418	-0.6586	DOWN	ENSMUSG00000121054
ENSMUSG00000121061	3.2123	-1.7896	DOWN	ENSMUSG00000121061
ENSMUSG00000121080	1.7353	-0.753	DOWN	ENSMUSG00000121080
ENSMUSG00000121083	1.3177	-0.9663	DOWN	ENSMUSG00000121083
ENSMUSG00000121121	1.3476	-0.6175	DOWN	ENSMUSG00000121121
ENSMUSG00000121153	1.5414	-1.2913	DOWN	ENSMUSG00000121153
ENSMUSG00000121256	1.4794	-1.0822	DOWN	ENSMUSG00000121256
ENSMUSG00002074925	6.2789	-1.3652	DOWN	5S_rRNA
ENSMUSG00002074982	3.1606	-1.642	DOWN	U6
ENSMUSG00002075075	19.3841	-2.3127	DOWN	ENSMUSG00002075075
ENSMUSG00002075231	3.418	-1.7363	DOWN	ENSMUSG00002075231
ENSMUSG00002075232	1.6718	-1.5397	DOWN	ENSMUSG00002075232
ENSMUSG00002076304	6.0584	-1.4266	DOWN	7SK
ENSMUSG00002076443	1.4675	-1.9232	DOWN	ENSMUSG00002076443
ENSMUSG00002076614	8.0097	-1.9403	DOWN	ENSMUSG00002076614

ENSMUSG00000000131	1.7123	0.1684	UP	Xpo6
ENSMUSG00000000340	2.0932	0.2056	UP	Dbt
ENSMUSG00000001082	2.1751	0.2758	UP	Mfsd10
ENSMUSG00000001441	1.342	0.2207	UP	Npepps
ENSMUSG00000002010	1.4846	0.2458	UP	Idh3g
ENSMUSG00000002014	4.2649	0.3103	UP	Ssr4
ENSMUSG00000003070	2.291	0.4303	UP	Efna2
ENSMUSG00000003545	2.4082	0.7482	UP	Fosb
ENSMUSG00000003873	1.7134	0.3008	UP	Bax
ENSMUSG00000004637	2.2238	0.2641	UP	Wwox
ENSMUSG00000006342	3.288	0.2507	UP	Susd2
ENSMUSG00000006732	1.4782	0.2158	UP	Mettl1
ENSMUSG00000008348	3.1025	0.4091	UP	Ubc
ENSMUSG00000016552	2.2263	0.2083	UP	Foxred2
ENSMUSG00000019470	1.3527	0.3092	UP	Xab2
ENSMUSG00000019726	1.359	0.1924	UP	Lyst
ENSMUSG00000020087	1.4945	0.239	UP	Tysnd1
ENSMUSG00000020530	1.3927	0.1664	UP	Ggnbp2
ENSMUSG00000020561	1.3924	0.1995	UP	Polr1f
ENSMUSG00000020674	1.4598	0.2178	UP	Pxdn
ENSMUSG00000020677	1.5111	0.2225	UP	Ddx52
ENSMUSG00000020794	1.321	0.2549	UP	Ube2g1
ENSMUSG00000020892	1.9353	0.2664	UP	Aloxe3
ENSMUSG00000020936	1.5896	0.1623	UP	Nmt1
ENSMUSG00000021290	2.6364	0.3624	UP	Atp5mpl
ENSMUSG00000021453	2.0201	0.3561	UP	Gadd45g
ENSMUSG00000021594	2.2319	0.2995	UP	Srd5a1
ENSMUSG00000021908	1.9353	0.2841	UP	Ncoa4-ps
ENSMUSG00000022066	1.4599	0.7411	UP	Entpd4b
ENSMUSG00000022108	1.4431	0.1847	UP	Itm2b
ENSMUSG00000022112	2.0391	0.192	UP	Gpc5
ENSMUSG00000022370	1.4643	0.3185	UP	Mrpl13
ENSMUSG00000022684	4.3558	0.2509	UP	Bfar
ENSMUSG00000023932	1.3919	0.5276	UP	Cdc5l
ENSMUSG00000024271	1.4829	0.1586	UP	Elp2
ENSMUSG00000024454	1.3241	0.2408	UP	Hdac3
ENSMUSG00000024580	2.3543	0.2638	UP	Grpel2
ENSMUSG00000024845	1.4233	0.2299	UP	Tmem134
ENSMUSG00000025201	5.229	2.0851	UP	Bloc1s2-ps
ENSMUSG00000025423	1.3525	0.1842	UP	Pias2
ENSMUSG00000025479	1.3698	1.1659	UP	Cyp2e1
ENSMUSG00000026227	1.488	0.2068	UP	2810459M11Rik
ENSMUSG00000026281	2.4371	0.1933	UP	Dtymk
ENSMUSG00000026571	1.7358	0.2194	UP	Dcaf6
ENSMUSG00000026849	1.898	0.2067	UP	Tor1a

ENSMUSG00000026869	1.3938	0.1621	UP	Psmd5
ENSMUSG00000027160	3.1293	0.3029	UP	Ccdc34
ENSMUSG00000027403	1.3124	0.7838	UP	Tgm6
ENSMUSG00000027466	2.0248	0.1776	UP	Rbck1
ENSMUSG00000027498	2.2612	0.2166	UP	Cstf1
ENSMUSG00000027716	1.5151	0.3482	UP	Trpc3
ENSMUSG00000027966	1.699	0.1975	UP	Col11a1
ENSMUSG00000028138	1.3476	0.1796	UP	Adh5
ENSMUSG00000028218	1.3768	0.3	UP	Cibar1
ENSMUSG00000028307	2.6216	0.3163	UP	Aldob
ENSMUSG00000028556	2.5033	0.1611	UP	Dock7
ENSMUSG00000029068	4.4943	0.1952	UP	Ccnl2
ENSMUSG00000029428	1.6692	0.2284	UP	Stx2
ENSMUSG00000029633	2.6636	1.4204	UP	Gm5578
ENSMUSG00000029822	4.9598	0.3143	UP	Osbpl3
ENSMUSG00000030083	1.359	0.2998	UP	Abtb1
ENSMUSG00000030127	2.5842	0.1704	UP	Cops7a
ENSMUSG00000030421	1.5739	0.2108	UP	Uri1
ENSMUSG00000030616	1.4986	0.2799	UP	Sytl2
ENSMUSG00000030986	2.9632	0.1919	UP	Dhx32
ENSMUSG00000031004	1.3425	0.763	UP	Mki67
ENSMUSG00000031487	1.7623	0.2025	UP	Brf2
ENSMUSG00000031760	3.8138	0.5854	UP	Mt3
ENSMUSG00000031879	1.6325	0.3343	UP	Ciao2b
ENSMUSG00000032475	1.6551	0.3502	UP	Nck1
ENSMUSG00000032503	1.3684	0.2238	UP	Arpp21
ENSMUSG00000032532	2.8436	0.4838	UP	Cck
ENSMUSG00000032563	1.8692	0.1843	UP	Mrpl3
ENSMUSG00000033004	1.8066	0.2159	UP	Mycbp2
ENSMUSG00000033014	1.4034	0.1968	UP	Trim33
ENSMUSG00000033323	1.9063	0.2362	UP	Ctdp1
ENSMUSG00000033499	1.8234	0.3081	UP	Larp4b
ENSMUSG00000033767	2.2119	0.269	UP	Tmem131l
ENSMUSG00000034620	1.9783	0.2724	UP	Rxylt1
ENSMUSG00000034848	1.7297	0.2992	UP	Ttc21b
ENSMUSG00000035129	3.6791	1.0078	UP	Gm6781
ENSMUSG00000035329	1.3515	0.3564	UP	Fbxo33
ENSMUSG00000035337	1.7123	1.3941	UP	Uchl4
ENSMUSG00000036292	1.401	0.2691	UP	Gramd1c
ENSMUSG00000036305	4.2649	1.361	UP	Rpl39-ps
ENSMUSG00000036427	3.1697	0.1766	UP	Gpi1
ENSMUSG00000036815	1.5724	0.1579	UP	Dpp10
ENSMUSG00000037096	2.4799	0.6532	UP	Gm9762
ENSMUSG00000037795	1.6805	0.1953	UP	N4bp2
ENSMUSG00000037876	2.0379	0.155	UP	Jmjd1c

ENSMUSG00000038102	1.3752	0.1951	UP	Trappc11
ENSMUSG00000039278	1.7276	0.2931	UP	Pcsk1n
ENSMUSG00000039308	2.2763	0.2954	UP	Ndst2
ENSMUSG00000039671	1.668	0.1856	UP	Zmynd8
ENSMUSG00000040128	1.6373	0.1622	UP	Pnrc1
ENSMUSG00000040136	2.0361	0.2347	UP	Abcc8
ENSMUSG00000040323	2.0103	0.7679	UP	Gm15429
ENSMUSG00000040554	1.6332	2.1016	UP	Aipl1
ENSMUSG00000040824	2.6642	0.2955	UP	Snrpd2
ENSMUSG00000041112	3.1102	0.2009	UP	Elmo1
ENSMUSG00000041453	5.5434	1.3323	UP	Rpl21
ENSMUSG00000041697	2.277	0.3292	UP	Cox6a1
ENSMUSG00000042165	2.2238	0.7228	UP	Gm9774
ENSMUSG00000042229	1.5256	0.1642	UP	Rabif
ENSMUSG00000042396	1.9192	0.2048	UP	Rbm7
ENSMUSG00000043165	1.8561	0.3063	UP	Lor
ENSMUSG00000043192	19.9354	2.4435	UP	Gpi-ps
ENSMUSG00000043801	3.6041	0.9576	UP	Oaz1-ps
ENSMUSG00000044268	1.867	1.1528	UP	Gm4895
ENSMUSG00000044285	4.9301	0.6512	UP	Ubb-ps
ENSMUSG00000044330	2.3585	1.4671	UP	Gm9790
ENSMUSG00000044709	3.2822	0.3499	UP	Gemin7
ENSMUSG00000044751	2.1316	1.2375	UP	Atp5pb-ps
ENSMUSG00000045055	6.22	1.2598	UP	Rpsa-ps2
ENSMUSG00000045083	2.528	0.2293	UP	Lingo2
ENSMUSG00000045104	7.4007	2.0188	UP	Ldhb-ps
ENSMUSG00000045886	3.2843	1.3481	UP	Pam16l
ENSMUSG00000046341	6.6641	0.8698	UP	Gm11223
ENSMUSG00000046580	2.777	1.7229	UP	Gm7862
ENSMUSG00000046687	1.6674	0.3886	UP	Gm5424
ENSMUSG00000047721	2.0313	0.647	UP	Bola2
ENSMUSG00000047843	5.5747	0.5292	UP	Bri3
ENSMUSG00000048334	1.5603	2.1729	UP	Gm8258
ENSMUSG00000048709	1.524	1.4551	UP	Gm8666
ENSMUSG00000048758	1.5415	0.452	UP	Rpl29
ENSMUSG00000049235	5.5929	1.1835	UP	Gm7324
ENSMUSG00000049658	1.8686	0.1797	UP	Bdp1
ENSMUSG00000049832	2.2366	1.3961	UP	Rbx1-ps
ENSMUSG00000049891	5.9509	1.5712	UP	Gm7984
ENSMUSG00000050243	1.9178	2.5785	UP	Gm5446
ENSMUSG00000050299	31.5088	1.6331	UP	Gm9843
ENSMUSG00000050550	1.7058	2.785	UP	Gm11868
ENSMUSG00000050855	1.9898	0.205	UP	Zfp940
ENSMUSG00000051373	1.5108	0.1812	UP	Plpp7
ENSMUSG00000052544	1.9192	0.312	UP	St6galnac3

ENSMUSG00000052748	2.2172	0.3315	UP	Swt1
ENSMUSG00000052837	1.3733	0.3911	UP	Junb
ENSMUSG00000052906	4.0496	0.2605	UP	Ubxn8
ENSMUSG00000053038	1.4843	0.622	UP	Gm6180
ENSMUSG00000053173	1.7223	1.8085	UP	Rpl18-ps2
ENSMUSG00000055681	1.7908	0.1794	UP	Cope
ENSMUSG00000056366	1.9935	1.2055	UP	Fabp3-ps1
ENSMUSG00000056412	3.1066	1.2602	UP	Psenen-ps
ENSMUSG00000057322	2.1987	0.3612	UP	Rpl38
ENSMUSG00000057469	1.82	0.292	UP	E2f6
ENSMUSG00000057580	4.255	2.8808	UP	Cox7c-ps1
ENSMUSG00000057990	1.6291	1.9207	UP	Gm53055
ENSMUSG00000058050	1.5165	0.809	UP	Gm9234
ENSMUSG00000058064	2.1325	1.0638	UP	Gm10036
ENSMUSG00000058126	3.6796	0.5135	UP	Tpm3-rs7
ENSMUSG00000058443	2.6065	1.7678	UP	Rpl10-ps3
ENSMUSG00000058581	3.0365	1.697	UP	Gm5801
ENSMUSG00000058809	2.4903	0.9649	UP	Hspd1-ps3
ENSMUSG00000059031	1.3104	1.2663	UP	Olfr482
ENSMUSG00000059049	3.3841	0.4827	UP	Frem1
ENSMUSG00000059070	2.1452	0.4138	UP	Rpl18
ENSMUSG00000059159	16.3583	1.9342	UP	Gm8129
ENSMUSG00000059183	1.4559	0.2848	UP	Mtfmt
ENSMUSG00000059461	5.5929	1.3609	UP	Gm7331
ENSMUSG00000059751	5.7184	1.5633	UP	Rps3a3
ENSMUSG00000059974	13.0499	0.3603	UP	Ntm
ENSMUSG00000060019	11.0571	1.7916	UP	Gm10073
ENSMUSG00000060198	4.8976	1.4961	UP	Gm11353
ENSMUSG00000060288	1.6573	0.8694	UP	Ppih
ENSMUSG00000060377	5.7693	1.992	UP	Rpl36a-ps1
ENSMUSG00000060438	6.9529	0.9576	UP	Rps10-ps1
ENSMUSG00000060467	4.0991	1.5348	UP	Gm10080
ENSMUSG00000060647	5.5755	1.377	UP	Gm7099
ENSMUSG00000060680	2.5302	2.4227	UP	Gm8894
ENSMUSG00000061167	2.0714	0.6955	UP	Rpl15-ps3
ENSMUSG00000061833	7.2176	2.2043	UP	Gm6311
ENSMUSG00000061848	7.401	1.9944	UP	Gm5805
ENSMUSG00000061950	1.5993	0.3196	UP	Ppp4r1
ENSMUSG00000062582	2.6302	2.4515	UP	Rpl30-ps8
ENSMUSG00000062611	6.7074	1.5103	UP	Rps3a2
ENSMUSG00000062846	2.3941	0.9021	UP	Gm14176
ENSMUSG00000063296	1.6077	0.2555	UP	Tmem117
ENSMUSG00000063314	3.6015	1.8886	UP	Gm12657
ENSMUSG00000063543	1.4133	2.4116	UP	Gm5616
ENSMUSG00000063953	2.3872	1.0884	UP	Amd2

ENSMUSG00000064193	4.9232	1.103	UP	Gm4735
ENSMUSG00000064317	5.8014	0.632	UP	Gm10146
ENSMUSG00000066068	4.335	2.2451	UP	Gm13611
ENSMUSG00000066148	1.6822	0.2034	UP	Prpf4
ENSMUSG00000066180	2.9657	2.186	UP	Gm10155
ENSMUSG00000066270	5.6072	1.4588	UP	Gm10157
ENSMUSG00000066315	4.3978	1.2654	UP	Gm12918
ENSMUSG00000066487	3.7111	1.9504	UP	Gm5786
ENSMUSG00000066491	15.1168	4.1457	UP	Cox6c2
ENSMUSG00000066543	2.3818	1.5778	UP	Rpl17-ps9
ENSMUSG00000066632	5.6833	1.1054	UP	Pgk1-rs7
ENSMUSG00000066724	4.98	1.6979	UP	Gm10175
ENSMUSG00000066842	1.5554	0.3712	UP	Hmcn1
ENSMUSG00000067121	8.6495	2.9019	UP	Gm7027
ENSMUSG00000067147	1.8066	1.4167	UP	Rpl7a-ps11
ENSMUSG00000067161	5.4432	1.6375	UP	Gm5560
ENSMUSG00000067321	3.3476	1.8407	UP	Gm7931
ENSMUSG00000067344	2.7769	1.7098	UP	Rps25-ps1
ENSMUSG00000067575	7.5828	2.4559	UP	Rpl35a-ps3
ENSMUSG00000067608	1.485	1.238	UP	Pcna-ps2
ENSMUSG00000067719	1.9813	0.4601	UP	Gm10221
ENSMUSG00000067924	1.4986	0.3286	UP	Rtl8b
ENSMUSG00000068243	1.4559	2.1235	UP	Gm7079
ENSMUSG00000068706	3.4051	0.5264	UP	Gm10250
ENSMUSG00000069011	4.6213	0.7626	UP	Gm10254
ENSMUSG00000069083	5.3983	3.2824	UP	Gm10259
ENSMUSG00000069379	4.4252	1.2682	UP	Gm4950
ENSMUSG00000069939	5.3951	1.3374	UP	Gm12070
ENSMUSG00000069972	4.9876	1.2494	UP	Rps13-ps2
ENSMUSG00000070343	17.6908	1.7949	UP	Gm10288
ENSMUSG00000070443	4.1351	2.6402	UP	Gm10291
ENSMUSG00000070490	4.2137	1.8888	UP	Gm10293
ENSMUSG00000070610	1.794	0.8472	UP	Gm13127
ENSMUSG00000070729	1.4443	0.3301	UP	Gm12966
ENSMUSG00000070871	1.7585	0.25	UP	Ccnyl1
ENSMUSG00000071035	10.1929	1.75	UP	Gm5499
ENSMUSG00000071078	1.3822	0.544	UP	Nr2c2ap
ENSMUSG00000071141	7.358	2.0089	UP	Rpl36a-ps3
ENSMUSG00000071151	2.2759	2.4247	UP	Gm4799
ENSMUSG00000071303	3.279	2.9908	UP	Rps8-ps1
ENSMUSG00000071343	2.777	1.8258	UP	Gm10327
ENSMUSG00000071415	2.5771	0.3314	UP	Rpl23
ENSMUSG00000071722	2.9189	0.7807	UP	Spin4
ENSMUSG00000072789	4.9267	2.6873	UP	Gm10420
ENSMUSG00000072915	1.8234	0.2327	UP	Gm12258

ENSMUSG00000073640	1.6557	1.3832	UP	Rpl27-ps3
ENSMUSG00000073737	7.9127	2.7742	UP	Gm10566
ENSMUSG00000073775	2.625	0.3091	UP	Kti12
ENSMUSG00000074034	3.1695	3.3125	UP	Gm5921
ENSMUSG00000075053	3.9544	0.9549	UP	Vdac3-ps1
ENSMUSG00000075279	3.7468	1.2902	UP	Mrpl23-ps1
ENSMUSG00000075391	2.9244	1.2381	UP	Glo1-ps
ENSMUSG00000075581	1.8967	2.3743	UP	Gm16409
ENSMUSG00000078126	1.8566	1.0677	UP	Rpl23a-ps3
ENSMUSG00000078139	5.6072	1.2879	UP	AK157302
ENSMUSG00000078162	1.6207	1.5236	UP	Gm2574
ENSMUSG00000078193	2.3359	1.1163	UP	Gm2000
ENSMUSG00000078480	3.9709	0.9629	UP	Mrpl48-ps
ENSMUSG00000078592	1.4388	1.2682	UP	Gm4609
ENSMUSG00000078897	4.5429	2.678	UP	Gm4724
ENSMUSG00000078967	8.3755	2.2844	UP	Gapdh-ps16
ENSMUSG00000079311	1.6619	1.2542	UP	Gm3222
ENSMUSG00000079501	3.0348	1.7711	UP	Gm5138
ENSMUSG00000079942	4.9125	1.5623	UP	Rpl28-ps3
ENSMUSG00000080006	1.484	2.5001	UP	Rps19-ps7
ENSMUSG00000080242	2.4163	0.7873	UP	Atp6v0c-ps2
ENSMUSG00000080746	2.3339	1.3199	UP	Rpsa-ps12
ENSMUSG00000080848	4.3116	1.3277	UP	Gm9385
ENSMUSG00000080859	3.7199	1.4929	UP	Rpl10-ps1
ENSMUSG00000080877	1.9438	1.8683	UP	Rpl22-ps1
ENSMUSG00000080902	2.275	0.5473	UP	Ywhaq-ps3
ENSMUSG00000080904	8.1702	4.5398	UP	Gm11966
ENSMUSG00000080921	1.3144	0.5448	UP	Rpl38-ps2
ENSMUSG00000080994	3.7517	2.9164	UP	Gm13464
ENSMUSG00000081010	1.7164	2.6051	UP	Gm13880
ENSMUSG00000081021	1.3154	1.6082	UP	Gm11964
ENSMUSG00000081049	4.2479	1.7827	UP	Rps24-ps3
ENSMUSG00000081087	3.5088	1.6401	UP	Rps15a-ps7
ENSMUSG00000081111	1.9774	1.3541	UP	Gm5913
ENSMUSG00000081113	2.433	1.0619	UP	Gm7308
ENSMUSG00000081121	3.5288	3.3568	UP	Gm12791
ENSMUSG00000081128	1.9638	1.9085	UP	Gm13328
ENSMUSG00000081157	1.8054	0.7833	UP	Gm13552
ENSMUSG00000081185	4.3776	2.4076	UP	Gm4852
ENSMUSG00000081201	1.3684	1.9846	UP	Smt3h2-ps4
ENSMUSG00000081214	5.0636	1.7663	UP	Rpl35a-ps2
ENSMUSG00000081221	3.2488	1.0037	UP	Gm14760
ENSMUSG00000081232	1.8519	3.0071	UP	Gm14373
ENSMUSG00000081239	2.4302	1.7815	UP	Gm11836
ENSMUSG00000081262	2.3285	1.3166	UP	Gm12261

ENSMUSG00000081281	3.6778	1.1815	UP	Rpl7-ps9
ENSMUSG00000081344	2.4154	1.417	UP	Gm14303
ENSMUSG00000081406	1.5023	0.7851	UP	Rps6-ps4
ENSMUSG00000081453	1.584	2.3329	UP	Gm6767
ENSMUSG00000081544	2.3631	1.3811	UP	Gm13077
ENSMUSG00000081559	1.9507	0.5983	UP	Gm12411
ENSMUSG00000081600	8.2128	2.3429	UP	Gm12286
ENSMUSG00000081643	1.7299	1.2101	UP	Gm11605
ENSMUSG00000081673	1.8678	1.9063	UP	Gm14794
ENSMUSG00000081684	2.6979	1.0022	UP	Rps2-ps13
ENSMUSG00000081700	4.331	2.9365	UP	Atp5k-ps2
ENSMUSG00000081788	7.0716	1.578	UP	Gm5898
ENSMUSG00000081824	3.0113	1.3296	UP	Ndufs5-ps
ENSMUSG00000081855	3.5965	1.0674	UP	Rpl17-ps5
ENSMUSG00000081888	2.0454	2.7993	UP	Spcs2-ps
ENSMUSG00000081926	4.335	3.6477	UP	Gm15536
ENSMUSG00000081957	2.507	1.1266	UP	Ak3l2-ps
ENSMUSG00000081975	1.5409	2.4278	UP	Gm12482
ENSMUSG00000081992	15.5859	2.7897	UP	Gm13408
ENSMUSG00000081999	6.5324	1.5706	UP	Gm13461
ENSMUSG00000082016	4.1947	0.5789	UP	Pgam1-ps2
ENSMUSG00000082029	3.2242	1.5867	UP	H3f3c
ENSMUSG00000082035	4.8196	1.9442	UP	Rpl17-ps8
ENSMUSG00000082044	5.9952	1.273	UP	Snrpert
ENSMUSG00000082072	3.103	2.174	UP	Gm15785
ENSMUSG00000082160	1.406	1.4707	UP	Gm11578
ENSMUSG00000082192	1.9853	0.8285	UP	Gm14719
ENSMUSG00000082193	2.0781	1.5726	UP	Rpl5-ps1
ENSMUSG00000082264	1.3678	1.1631	UP	Gm12799
ENSMUSG00000082274	2.0208	0.8377	UP	Gm14026
ENSMUSG00000082284	1.7123	1.2328	UP	H3f3a-ps1
ENSMUSG00000082394	2.037	2.0851	UP	Gm4596
ENSMUSG00000082424	10.7847	2.0278	UP	Gm13292
ENSMUSG00000082454	3.0692	0.7252	UP	Gm12183
ENSMUSG00000082475	21.5705	2.3526	UP	Gm7206
ENSMUSG00000082491	3.0523	3.3066	UP	Gm5909
ENSMUSG00000082530	5.0636	3.4253	UP	Gm12168
ENSMUSG00000082536	42.9803	2.1122	UP	Gm13456
ENSMUSG00000082778	1.8066	1.7823	UP	Gm15191
ENSMUSG00000082809	2.2367	0.9478	UP	Gm14150
ENSMUSG00000082876	1.5425	1.8218	UP	Gm11889
ENSMUSG00000082895	3.6441	2.0395	UP	Rpsa-ps9
ENSMUSG00000082896	17.4473	2.1458	UP	Gm5844
ENSMUSG00000082953	1.3873	0.9385	UP	Gm13217
ENSMUSG00000083011	1.7068	1.9634	UP	Gm12816

ENSMUSG00000083044	3.275	2.6836	UP	Gm12416
ENSMUSG00000083287	2.0604	1.0179	UP	Idi1-ps1
ENSMUSG00000083325	3.432	0.8778	UP	Gm14121
ENSMUSG00000083327	2.2263	0.4262	UP	Vcp-rs
ENSMUSG00000083328	1.5541	2.1308	UP	Gm11826
ENSMUSG00000083424	3.4012	2.2484	UP	Rpl35a-ps4
ENSMUSG00000083481	3.0262	2.6225	UP	Rps8-ps2
ENSMUSG00000083496	3.9655	1.4212	UP	Gm11263
ENSMUSG00000083563	8.7315	2.9643	UP	Gm13340
ENSMUSG00000083619	3.5895	3.2057	UP	Gm14414
ENSMUSG00000083621	25.2345	1.9271	UP	Gm14586
ENSMUSG00000083679	5.5806	1.7929	UP	Gm12892
ENSMUSG00000083692	2.5197	0.7884	UP	Gm9575
ENSMUSG00000083716	23.9553	2.3753	UP	Gm13436
ENSMUSG00000083820	6.0717	1.8693	UP	Ndufs6b
ENSMUSG00000083854	6.5367	1.2793	UP	Dnajc19-ps
ENSMUSG00000083899	12.2151	0.8127	UP	Gm12346
ENSMUSG00000083992	10.3637	2.5048	UP	Gm11478
ENSMUSG00000084013	2.4258	1.0887	UP	Gm14270
ENSMUSG00000084111	3.2789	0.7515	UP	Gm15710
ENSMUSG00000084131	6.7409	1.8845	UP	Rpl3-ps2
ENSMUSG00000084145	9.3966	2.0338	UP	Gm12263
ENSMUSG00000084159	4.0939	1.225	UP	Gm12696
ENSMUSG00000084166	1.7448	1.7337	UP	Gm6451
ENSMUSG00000084168	3.1995	1.5851	UP	Atp5l2-ps
ENSMUSG00000084235	8.2885	1.9757	UP	Gm15421
ENSMUSG00000084304	2.4237	2.3463	UP	Gm6142
ENSMUSG00000084314	2.4095	2.5068	UP	Rps15a-ps3
ENSMUSG00000084323	1.9476	1.6457	UP	Gm14438
ENSMUSG00000084329	1.8692	1.5419	UP	Gm6733
ENSMUSG00000084349	5.6678	0.7667	UP	Rpl3-ps1
ENSMUSG00000084817	7.5684	2.0726	UP	Gm5526
ENSMUSG00000084830	12.6138	1.0787	UP	Gm14539
ENSMUSG00000085342	6.5535	1.7973	UP	Gm12254
ENSMUSG00000085711	3.0251	1.0211	UP	Gm15163
ENSMUSG00000086567	11.4089	2.6319	UP	Gm2830
ENSMUSG00000086691	2.6859	1.7141	UP	Gm15432
ENSMUSG00000086925	1.8223	2.1931	UP	Gm6286
ENSMUSG00000087153	2.5899	0.638	UP	Gm6483
ENSMUSG00000087635	2.277	0.6796	UP	Gm13414
ENSMUSG00000087701	16.8999	1.2648	UP	Gm13493
ENSMUSG00000089756	1.7168	2.5449	UP	Zfp966
ENSMUSG00000089838	2.6221	1.1574	UP	Gm2962
ENSMUSG00000089936	1.3134	1.3116	UP	Gm16199
ENSMUSG00000089988	2.0783	2.1379	UP	Gm16238

ENSMUSG00000090021	1.5112	0.8453	UP	Gm6493
ENSMUSG00000090381	2.0844	1.0885	UP	Gm6158
ENSMUSG00000090389	3.2789	1.0957	UP	Cdv3-ps
ENSMUSG00000090516	5.3983	1.8996	UP	Rps11-ps1
ENSMUSG00000090602	6.0952	1.5124	UP	Gm5611
ENSMUSG00000090610	2.4778	0.4429	UP	Gm3571
ENSMUSG00000090665	2.6283	2.594	UP	Gad1-ps
ENSMUSG00000090704	5.048	3.0311	UP	Trp53-ps
ENSMUSG00000091086	4.8438	0.8919	UP	Rpl6l
ENSMUSG00000091269	7.6828	1.5936	UP	Gm6682
ENSMUSG00000091905	1.5051	0.4105	UP	Dnajb6-ps
ENSMUSG00000091989	3.3838	0.9649	UP	Ndufab1-ps
ENSMUSG00000092074	1.8547	0.3106	UP	Dynlt1a
ENSMUSG00000092281	1.436	1.8146	UP	Hmgb1-ps7
ENSMUSG00000093651	3.4397	1.6056	UP	Gm5873
ENSMUSG00000093798	2.8758	0.975	UP	Gm8355
ENSMUSG00000093826	1.5732	1.9322	UP	Gm6900
ENSMUSG00000094320	2.1614	1.6041	UP	Chchd2-ps
ENSMUSG00000094344	2.9531	1.023	UP	Gm11942
ENSMUSG00000094388	2.0952	1.6616	UP	Gm8783
ENSMUSG00000094463	2.079	1.1734	UP	Gm8546
ENSMUSG00000094497	2.6373	2.0648	UP	Gm8210
ENSMUSG00000094568	3.3868	0.909	UP	Smarce1-ps1
ENSMUSG00000094974	2.2868	2.3649	UP	Rps19-ps2
ENSMUSG00000095042	1.6526	0.96	UP	Gm12537
ENSMUSG00000095403	1.5549	0.7289	UP	Gm21092
ENSMUSG00000095588	5.2123	1.8665	UP	Gm12350
ENSMUSG00000095742	13.1767	1.1807	UP	ENSMUSG00000095742
ENSMUSG00000095847	10.3637	1.563	UP	Gm5451
ENSMUSG00000096160	3.5504	2.1553	UP	Gm3436
ENSMUSG00000096361	8.7159	1.7862	UP	Gm5814
ENSMUSG00000096403	2.669	1.1602	UP	Rnps1-ps
ENSMUSG00000096438	1.9853	1.9556	UP	Gapdh-ps15
ENSMUSG00000096449	1.5541	1.8484	UP	Gm4076
ENSMUSG00000096474	1.5678	2.0364	UP	Gm5561
ENSMUSG00000096712	1.6271	1.6709	UP	Gm15454
ENSMUSG00000096810	12.1374	2.7748	UP	Gm10481
ENSMUSG00000096842	3.3895	2.0653	UP	Gm10736
ENSMUSG00000097148	2.3339	1.7364	UP	Gm3839
ENSMUSG00000097245	6.5147	1.7382	UP	Gm5421
ENSMUSG00000097388	5.4747	3.0779	UP	Gm3200
ENSMUSG00000097464	2.1227	1.5281	UP	Gm26736
ENSMUSG00000097979	3.0155	1.545	UP	Gm4691
ENSMUSG00000097989	3.9413	2.5927	UP	Gm4335
ENSMUSG00000098019	6.7018	3.9412	UP	Gm2546

ENSMUSG00000098041	1.3587	0.4711	UP	Gm26981
ENSMUSG00000098240	1.4685	2.216	UP	Gm4575
ENSMUSG00000098915	4.2649	3.284	UP	Rpl15-ps2
ENSMUSG00000098985	2.3461	1.2503	UP	Gm27219
ENSMUSG00000099342	2.6694	1.4809	UP	Gm18180
ENSMUSG00000099377	1.8176	1.3252	UP	Gm6159
ENSMUSG00000099471	3.3049	1.2557	UP	Gm8451
ENSMUSG00000099492	6.7018	2.7744	UP	Gm5525
ENSMUSG00000099662	1.3919	1.7254	UP	Ppp1r2-ps2
ENSMUSG00000099779	8.6803	2.7077	UP	Gm8228
ENSMUSG00000099902	2.8459	1.6693	UP	Gm12115
ENSMUSG00000100104	5.991	3.3035	UP	Gm5644
ENSMUSG00000100215	5.7927	2.1558	UP	Gm8292
ENSMUSG00000100261	1.5516	1.1561	UP	Gm6473
ENSMUSG00000100397	2.0952	3.2025	UP	Gm28071
ENSMUSG00000100794	2.6578	2.6299	UP	Gm29667
ENSMUSG00000100863	1.3892	0.9194	UP	Gm12669
ENSMUSG00000100891	2.0318	0.5164	UP	2810049E08Rik
ENSMUSG00000101316	3.7596	0.4888	UP	Gm12663
ENSMUSG00000101431	3.6296	1.3778	UP	Gm7901
ENSMUSG00000101589	8.8058	2.3894	UP	Rbm6-ps1
ENSMUSG00000101795	9.7464	3.4145	UP	Gm5835
ENSMUSG00000102038	3.2116	1.0217	UP	Gm12345
ENSMUSG00000102070	1.3196	1.1349	UP	Gm28661
ENSMUSG00000102117	2.0187	2.2217	UP	Rpsa-ps1
ENSMUSG00000102386	2.5789	0.8515	UP	2900022M07Rik
ENSMUSG00000102478	3.3275	2.0692	UP	BC085271
ENSMUSG00000102747	3.9527	1.7256	UP	Gm37602
ENSMUSG00000103309	2.5708	1.4633	UP	BC037039
ENSMUSG00000103735	2.3994	0.803	UP	Gm38317
ENSMUSG00000103922	3.8524	1.0732	UP	Gm6123
ENSMUSG00000104126	1.9246	1.6304	UP	Gm37486
ENSMUSG00000104222	3.0617	1.242	UP	Gm7292
ENSMUSG00000104496	1.9192	0.9149	UP	Gm5837
ENSMUSG00000104649	3.5052	2.3865	UP	Gm43712
ENSMUSG00000104699	1.3204	1.6023	UP	Rps4x-ps
ENSMUSG00000104802	14.2529	3.7045	UP	Gm5869
ENSMUSG00000104913	4.6926	0.6933	UP	Gm6560
ENSMUSG00000105081	2.2076	1.9804	UP	Gm43110
ENSMUSG00000105144	3.0897	2.9798	UP	Rpl21-ps11
ENSMUSG00000105359	1.9476	1.7298	UP	Rpl21-ps10
ENSMUSG00000105558	5.7927	3.6774	UP	Gm5855
ENSMUSG00000105687	6.9415	1.4923	UP	Gm6157
ENSMUSG00000105814	22.599	4.4766	UP	Mir703
ENSMUSG00000105879	2.073	0.9438	UP	Gm6204

ENSMUSG00000106037	5.0132	1.3867	UP	Gm4332
ENSMUSG00000106133	1.3783	0.5564	UP	Gm3724
ENSMUSG00000106390	3.2772	1.7835	UP	Gm5551
ENSMUSG00000106574	3.7293	2.092	UP	Gm2451
ENSMUSG00000106831	3.4876	1.0682	UP	Ube2n-ps1
ENSMUSG00000106926	6.8706	2.8604	UP	Rpl7-ps7
ENSMUSG00000106988	3.5592	1.8195	UP	Tsg101-ps
ENSMUSG00000107257	2.2367	2.7234	UP	Gm43028
ENSMUSG00000107261	2.3876	2.1856	UP	Trmt112-ps1
ENSMUSG00000107369	5.8875	1.0093	UP	Gstm2-ps1
ENSMUSG00000107383	6.9915	0.5937	UP	Gm4366
ENSMUSG00000107470	1.4782	0.4317	UP	Gm3375
ENSMUSG00000107747	6.1036	1.9168	UP	Gm5881
ENSMUSG00000108231	4.0036	2.0747	UP	Gm4045
ENSMUSG00000108264	2.0462	1.8194	UP	Gm20371
ENSMUSG00000108314	1.9438	0.4357	UP	Prkcz2
ENSMUSG00000108366	1.9831	2.5166	UP	Gm5586
ENSMUSG00000108381	1.5394	1.1556	UP	Gm9299
ENSMUSG00000108702	3.779	1.0585	UP	Gm9333
ENSMUSG00000108772	1.3792	1.8026	UP	Gm6063
ENSMUSG00000108799	4.9876	1.1946	UP	Glud-ps
ENSMUSG00000108804	2.078	1.1861	UP	Gm30437
ENSMUSG00000108823	3.944	2.7697	UP	Gm18959
ENSMUSG00000108852	1.8885	0.4852	UP	Gm44911
ENSMUSG00000109509	1.5145	1.2572	UP	Rps12-ps4
ENSMUSG00000109536	4.3792	0.4549	UP	9330162G02Rik
ENSMUSG00000109610	3.3476	2.9471	UP	Gm7432
ENSMUSG00000109894	2.0033	2.0547	UP	Gm5904
ENSMUSG00000110057	5.1575	1.7811	UP	Gm2225
ENSMUSG00000110126	2.9007	1.9427	UP	Gm9347
ENSMUSG00000110275	9.6821	1.7548	UP	Gm5905
ENSMUSG00000110469	1.7382	2.229	UP	Gm10358
ENSMUSG00000110529	1.6	0.5436	UP	Gm45694
ENSMUSG00000110545	1.4945	0.9929	UP	Gm7730
ENSMUSG00000110644	2.2422	0.9771	UP	Gm7390
ENSMUSG00000110679	5.2538	1.805	UP	Rpl10-ps5
ENSMUSG00000110726	1.371	1.5297	UP	Gm18101
ENSMUSG00000110744	2.3215	1.7974	UP	Gm5171
ENSMUSG00000111594	1.4538	0.862	UP	Gm3365
ENSMUSG00000111897	45.3246	2.4572	UP	Gm19810
ENSMUSG00000111964	4.1417	3.7384	UP	Gm8942
ENSMUSG00000112515	2.7397	2.576	UP	Gm4928
ENSMUSG00000112693	2.7765	1.5799	UP	Gm5512
ENSMUSG00000112908	5.8458	2.2275	UP	Gm7392
ENSMUSG00000112909	2.1614	2.0331	UP	Gm10120

ENSMUSG00000113061	5.5245	1.2614	UP	Rps18-ps5
ENSMUSG00000113113	1.6066	1.8406	UP	Gm2614
ENSMUSG00000113188	2.6258	1.3833	UP	Gm35638
ENSMUSG00000113255	2.5789	0.867	UP	Tes3-ps
ENSMUSG00000113275	4.5489	2.154	UP	Tubb2a-ps2
ENSMUSG00000113389	7.2449	3.4063	UP	Gm9512
ENSMUSG00000113606	5.8786	3.0936	UP	Gm47441
ENSMUSG00000113637	1.8293	2.2251	UP	Gm7049
ENSMUSG00000113690	2.4799	2.0149	UP	Gm36501
ENSMUSG00000113743	3.5302	1.7714	UP	Gm8712
ENSMUSG00000114003	1.3946	0.4323	UP	Gm9616
ENSMUSG00000114488	7.3787	2.0422	UP	Gm5802
ENSMUSG00000114551	2.0201	2.4751	UP	Gm6035
ENSMUSG00000114571	10.5502	5.0521	UP	Gm35595
ENSMUSG00000114579	7.3608	1.1074	UP	Gm4130
ENSMUSG00000114886	2.0103	2.6429	UP	Gm48432
ENSMUSG00000114970	5.5568	1.076	UP	Gm49069
ENSMUSG00000114993	1.6573	1.6318	UP	Gm6363
ENSMUSG00000115160	1.9261	2.2818	UP	Gm6532
ENSMUSG00000115194	3.5504	3.3193	UP	Gm48909
ENSMUSG00000115205	4.1957	2.6824	UP	Gm16374
ENSMUSG00000115280	4.3226	1.2759	UP	Gm7107
ENSMUSG00000115312	1.7296	2.3796	UP	Gm8518
ENSMUSG00000115448	4.1031	1.9219	UP	Gm21178
ENSMUSG00000115457	2.7085	2.4448	UP	Gm2387
ENSMUSG00000115497	2.8366	2.3574	UP	Gm49207
ENSMUSG00000115637	2.7327	1.4345	UP	Gm30970
ENSMUSG00000116174	1.4826	2.0005	UP	Gm10362
ENSMUSG00000116757	1.3196	1.9064	UP	Gm4786
ENSMUSG00000116875	1.5882	0.3883	UP	Morf4l1-ps1
ENSMUSG00000116958	1.8555	1.9098	UP	Gm6705
ENSMUSG00000117187	2.0156	1.4826	UP	Gm4708
ENSMUSG00000117278	4.3751	2.5803	UP	Gm36684
ENSMUSG00000117405	1.7737	2.6648	UP	Rpl19-ps7
ENSMUSG00000117428	4.0365	2.0427	UP	Gm4833
ENSMUSG00000117458	2.3872	1.5263	UP	Gm6552
ENSMUSG00000117484	2.0706	1.7659	UP	Gm5500
ENSMUSG00000117621	1.3748	0.6479	UP	Hspe1-rs1
ENSMUSG00000117730	1.4144	1.1619	UP	Gm5503
ENSMUSG00000117822	15.4963	2.8996	UP	Eef1a1-ps1
ENSMUSG00000117875	16.6658	3.0374	UP	Gm6789
ENSMUSG00000118140	1.357	0.9544	UP	Gm4949
ENSMUSG00000118252	5.6296	2.3012	UP	Gm5521
ENSMUSG00000118264	3.5975	0.9848	UP	Rps15-ps3
ENSMUSG00000120022	1.5188	1.0293	UP	ENSMUSG00000120022

ENSMUSG00000120425	1.3342	1.1125	UP	ENSMUSG00000120425
ENSMUSG00000120678	1.7076	1.2802	UP	ENSMUSG00000120678
ENSMUSG00000121177	2.1071	1.0304	UP	ENSMUSG00000121177
**SNI: TRF_M_VS_A**				
ENSMUSG00000000214	17.0833	-0.7044	DOWN	Th
ENSMUSG00000008348	4.4859	-0.2628	DOWN	Ubc
ENSMUSG00000018822	7.4428	-0.5266	DOWN	Sfrp5
ENSMUSG00000020182	1.7683	-0.2424	DOWN	Ddc
ENSMUSG00000020889	1.5377	-0.2751	DOWN	Nr1d1
ENSMUSG00000020893	1.6190	-0.2976	DOWN	Per1
ENSMUSG00000021609	4.6876	-1.3770	DOWN	Slc6a3
ENSMUSG00000021775	2.8735	-0.2388	DOWN	Nr1d2
ENSMUSG00000024747	3.6853	-1.6558	DOWN	Aldh1a7
ENSMUSG00000025370	1.3977	-0.3930	DOWN	Cdh9
ENSMUSG00000025586	1.3166	-0.1845	DOWN	Cpeb1
ENSMUSG00000026686	2.0858	-0.4873	DOWN	Lmx1a
ENSMUSG00000028957	1.4777	-0.2622	DOWN	Per3
ENSMUSG00000029219	8.1534	-0.9562	DOWN	Slc10a4
ENSMUSG00000030110	7.2675	-0.4358	DOWN	Ret
ENSMUSG00000030495	1.4218	-0.1886	DOWN	Slc7a10
ENSMUSG00000031491	13.5994	-1.0425	DOWN	Chrna6
ENSMUSG00000031492	6.9024	-1.1244	DOWN	Chrnb3
ENSMUSG00000032572	3.7865	-1.0882	DOWN	Col6a4
ENSMUSG00000034892	10.1246	-0.5869	DOWN	Rps29
ENSMUSG00000038146	2.3501	-0.2894	DOWN	Notch3
ENSMUSG00000038550	2.5746	-0.6104	DOWN	Ciart
ENSMUSG00000038630	1.6433	-0.4592	DOWN	Zkscan16
ENSMUSG00000045193	1.5769	-0.2784	DOWN	Cirbp
ENSMUSG00000050856	1.4021	-0.3689	DOWN	Atp5k
ENSMUSG00000053279	6.8541	-0.4946	DOWN	Aldh1a1
ENSMUSG00000055866	1.9284	-0.2826	DOWN	Per2
ENSMUSG00000058665	6.7158	-1.5085	DOWN	En1
ENSMUSG00000059159	7.0362	-1.0552	DOWN	Gm8129
ENSMUSG00000062488	2.2300	-0.6726	DOWN	Ifit3b
ENSMUSG00000066116	1.7372	-0.8083	DOWN	Gm10154
ENSMUSG00000069917	2.0447	-0.6719	DOWN	Hba-a2
ENSMUSG00000071714	2.0989	-0.4738	DOWN	Csf2rb2
ENSMUSG00000072940	5.9799	-0.5075	DOWN	Gm10443
ENSMUSG00000081281	1.3938	-0.9660	DOWN	Rpl7-ps9
ENSMUSG00000082454	2.9455	-0.4541	DOWN	Gm12183
ENSMUSG00000083327	5.6405	-0.4565	DOWN	Vcp-rs
ENSMUSG00000084093	4.5692	-0.8183	DOWN	Gm16418
ENSMUSG00000089782	3.3643	-0.6568	DOWN	Btf3-ps1
ENSMUSG00000091639	6.8742	-0.6338	DOWN	Gm3756
ENSMUSG00000097023	2.0851	-0.3699	DOWN	Mir9-3hg

ENSMUSG00000100215	2.1655	-1.3237	DOWN	Gm8292
ENSMUSG00000104960	2.7888	-0.4155	DOWN	Snhg8
ENSMUSG00000107176	1.5766	-0.3111	DOWN	Gm9794
ENSMUSG00000111897	2.3501	-0.4567	DOWN	Gm19810
ENSMUSG00000114253	1.5818	-0.9639	DOWN	Gm47798
ENSMUSG00000114999	1.4973	-1.2090	DOWN	Gm7962
ENSMUSG00000115868	3.0205	-0.5584	DOWN	Gm2999
ENSMUSG00000000560	1.9867	0.2383	UP	Gabra2
ENSMUSG00000000957	1.3938	0.3777	UP	Mmp14
ENSMUSG00000001700	1.5377	0.2902	UP	Gramd3
ENSMUSG00000006154	2.0757	0.5683	UP	Eps8l1
ENSMUSG00000021071	1.5243	0.2082	UP	Trim9
ENSMUSG00000021270	1.4254	0.1651	UP	Hsp90aa1
ENSMUSG00000027236	1.3938	0.2765	UP	Eif3j1
ENSMUSG00000029817	1.6073	0.2626	UP	Tra2a
ENSMUSG00000030002	1.7367	0.1845	UP	Dusp11
ENSMUSG00000035711	1.9867	0.5110	UP	Dok3
ENSMUSG00000041378	2.1616	0.3953	UP	Cldn5
ENSMUSG00000041930	2.2829	0.3591	UP	Fam222a
ENSMUSG00000043091	4.0087	0.4516	UP	Tuba1c
ENSMUSG00000043872	2.2815	0.4506	UP	Zmym1
ENSMUSG00000047635	1.6625	0.3743	UP	Mtrfr
ENSMUSG00000047676	1.7067	0.4022	UP	Rpsa-ps10
ENSMUSG00000055116	2.5412	0.3999	UP	Arntl
ENSMUSG00000058443	1.7683	0.7848	UP	Rpl10-ps3
ENSMUSG00000060373	1.3161	0.1678	UP	Hnrnpc
ENSMUSG00000062456	4.7034	0.4373	UP	Rpl9-ps6
ENSMUSG00000064358	2.9959	0.8386	UP	mt-Co3
ENSMUSG00000064360	3.2623	0.4253	UP	mt-Nd3
ENSMUSG00000067038	6.9158	0.4239	UP	Rps12-ps3
ENSMUSG00000067870	3.5433	0.4461	UP	Rpl31-ps8
ENSMUSG00000069014	5.5957	1.2585	UP	Gm5641
ENSMUSG00000069939	6.8686	1.1726	UP	Gm12070
ENSMUSG00000074884	2.0447	0.3384	UP	Serf2
ENSMUSG00000075232	1.7372	0.2019	UP	Amd1
ENSMUSG00000078636	7.8038	1.4678	UP	Gm7336
ENSMUSG00000078967	1.9266	1.4895	UP	Gapdh-ps16
ENSMUSG00000079224	3.6206	0.8012	UP	Gm6565
ENSMUSG00000080859	2.1381	1.7891	UP	Rpl10-ps1
ENSMUSG00000081344	1.8062	0.4257	UP	Gm14303
ENSMUSG00000081400	1.9482	0.7666	UP	Gm13680
ENSMUSG00000082286	1.6957	0.3597	UP	Pisd-ps1
ENSMUSG00000082585	2.2815	0.9083	UP	Gm15387
ENSMUSG00000083773	5.0399	0.9774	UP	Gm13394
ENSMUSG00000083889	1.3938	0.4748	UP	E530001F21Rik

ENSMUSG00000083899	2.1228	0.3912	UP	Gm12346
ENSMUSG00000085442	3.0252	0.9853	UP	Gm3362
ENSMUSG00000094066	3.2623	1.8158	UP	Fam205a2
ENSMUSG00000097496	1.3075	1.0495	UP	Gm26653
ENSMUSG00000100007	2.3501	1.1403	UP	Gm5527
ENSMUSG00000100801	2.4063	0.4542	UP	Gm15459
ENSMUSG00000106831	1.7372	0.8968	UP	Ube2n-ps1
ENSMUSG00000111971	3.0447	0.9289	UP	Gm48678
ENSMUSG00000120241	5.3794	0.5213	UP	ENSMUSG00000120241
**SNI: TRF_A_VS_ALF_A**				
ENSMUSG00000023034	1.3258	-0.3967	DOWN	Nr4a1
ENSMUSG00000027694	1.9912	-0.6950	DOWN	Gm8325
ENSMUSG00000050900	1.8314	-1.2425	DOWN	Gm7327
ENSMUSG00000056579	1.4078	-0.1682	DOWN	Tug1
ENSMUSG00000064193	1.4078	-0.7483	DOWN	Gm4735
ENSMUSG00000064358	1.4010	-0.7294	DOWN	mt-Co3
ENSMUSG00000069939	2.0821	-1.1103	DOWN	Gm12070
ENSMUSG00000071341	1.4191	-0.7866	DOWN	Egr4
ENSMUSG00000078636	1.4191	-0.9505	DOWN	Gm7336
ENSMUSG00000078899	1.4010	-4.9126	DOWN	Gm4631
ENSMUSG00000082286	1.3258	-0.3307	DOWN	Pisd-ps1
ENSMUSG00000082536	1.4191	-0.5792	DOWN	Gm13456
ENSMUSG00000083563	1.4078	-0.4896	DOWN	Gm13340
ENSMUSG00000087701	1.3258	-0.6649	DOWN	Gm13493
ENSMUSG00000094066	2.3753	-1.9883	DOWN	Fam205a2
ENSMUSG00000094989	1.3057	-0.4737	DOWN	Rpl9-ps4
ENSMUSG00000095041	1.4078	-0.5724	DOWN	ENSMUSG00000095041
ENSMUSG00000096768	1.4010	-2.1173	DOWN	Gm47283
ENSMUSG00000100801	1.4010	-0.3065	DOWN	Gm15459
ENSMUSG00000101249	16.6215	-0.4970	DOWN	Gm29216
ENSMUSG00000006412	2.0309	0.2217	UP	Pfdn2
ENSMUSG00000020085	1.4191	0.2971	UP	Aifm2
ENSMUSG00000020163	1.4191	0.2468	UP	Uqcr11
ENSMUSG00000021606	1.4010	0.2828	UP	Ndufs6
ENSMUSG00000022890	2.5910	0.1939	UP	Atp5j
ENSMUSG00000030681	1.4078	0.3584	UP	Mvp
ENSMUSG00000035674	1.7933	0.2488	UP	Ndufa3
ENSMUSG00000037166	1.6513	0.3254	UP	Ppp1r14a
ENSMUSG00000054793	2.5910	0.1636	UP	Cadm4
ENSMUSG00000057177	1.4010	0.1633	UP	Gsk3a
ENSMUSG00000059159	1.4706	0.6613	UP	Gm8129
ENSMUSG00000090553	1.4191	0.3335	UP	Snrpe
ENSMUSG00000115868	2.0309	0.4017	UP	Gm2999
**sham: TRF_M_VS_A**				
ENSMUSG00000000093	1.324	-0.3764	DOWN	Tbx2

ENSMUSG00000000148	1.8393	-0.2383	DOWN	Brat1
ENSMUSG00000000732	2.1093	-0.4495	DOWN	Icosl
ENSMUSG00000000738	1.4022	-0.1562	DOWN	Spg7
ENSMUSG00000000787	1.3503	-0.1496	DOWN	Ddx3x
ENSMUSG00000000811	5.6295	-0.4789	DOWN	Txnrd3
ENSMUSG00000000823	3.6135	-0.2842	DOWN	Zfp512b
ENSMUSG00000001227	1.3878	-0.1884	DOWN	Sema6b
ENSMUSG00000001555	1.7871	-0.3459	DOWN	Fkbp10
ENSMUSG00000002059	4.9998	-0.4075	DOWN	Rab34
ENSMUSG00000002831	2.6054	-0.6004	DOWN	Plin4
ENSMUSG00000002835	1.6309	-0.6256	DOWN	Chaf1a
ENSMUSG00000002910	1.9433	-0.3961	DOWN	Arrdc2
ENSMUSG00000002949	2.4891	-0.2284	DOWN	Timm44
ENSMUSG00000003541	1.3483	-0.3616	DOWN	Ier3
ENSMUSG00000004328	11.8645	-1.3037	DOWN	Hif3a
ENSMUSG00000004996	1.5191	-0.2495	DOWN	Mri1
ENSMUSG00000005262	1.3673	-0.177	DOWN	Ufd1
ENSMUSG00000005514	1.3479	-0.1847	DOWN	Por
ENSMUSG00000005774	3.1701	-0.3137	DOWN	Rfx5
ENSMUSG00000005951	1.444	-0.4254	DOWN	Shpk
ENSMUSG00000006307	2.8578	-0.3255	DOWN	Kmt2b
ENSMUSG00000006471	1.7831	-0.3274	DOWN	Ndor1
ENSMUSG00000006611	1.3383	-0.3935	DOWN	Hfe
ENSMUSG00000007570	1.8954	-0.2585	DOWN	Fance
ENSMUSG00000007836	4.9844	-0.2776	DOWN	Hnrnpa0
ENSMUSG00000008200	1.736	-0.1797	DOWN	Fnbp4
ENSMUSG00000008307	1.4783	-0.5583	DOWN	1700109H08Rik
ENSMUSG00000008384	1.6626	-0.6803	DOWN	Sertad1
ENSMUSG00000009378	1.3107	-0.7664	DOWN	Slc16a12
ENSMUSG00000009905	1.4875	-0.1749	DOWN	Kdsr
ENSMUSG00000010307	1.8657	-0.4117	DOWN	Tmem86a
ENSMUSG00000010660	2.4419	-0.3412	DOWN	Plcd1
ENSMUSG00000011306	2.427	-0.2269	DOWN	Sugp1
ENSMUSG00000012017	2.4419	-0.5169	DOWN	Scarf2
ENSMUSG00000012126	1.4705	-0.2344	DOWN	Ubxn11
ENSMUSG00000014361	2.2423	-0.3163	DOWN	Mertk
ENSMUSG00000014791	1.6231	-0.3171	DOWN	Elmo3
ENSMUSG00000015217	5.1877	-0.4001	DOWN	Hmgb3
ENSMUSG00000015377	1.8065	-0.178	DOWN	Dennd6b
ENSMUSG00000016024	1.518	-0.5305	DOWN	Lbp
ENSMUSG00000016552	2.5436	-0.2669	DOWN	Foxred2
ENSMUSG00000016624	1.6464	-0.2959	DOWN	Phf21b
ENSMUSG00000017009	2.7359	-0.2122	DOWN	Sdc4
ENSMUSG00000018566	2.7379	-0.7277	DOWN	Slc2a4
ENSMUSG00000018669	1.6083	-0.239	DOWN	Cdk5rap3

ENSMUSG00000018678	1.4386	-0.2441	DOWN	Sp2
ENSMUSG00000018822	22.3787	-0.7704	DOWN	Sfrp5
ENSMUSG00000018862	2.0874	-0.9767	DOWN	Otop3
ENSMUSG00000019232	7.5461	-0.2811	DOWN	Etnppl
ENSMUSG00000019338	3.8749	-0.281	DOWN	Zfp687
ENSMUSG00000019437	1.4231	-0.2068	DOWN	Tlcd1
ENSMUSG00000020038	1.6559	-0.2371	DOWN	Cry1
ENSMUSG00000020072	1.4763	-0.57	DOWN	Pbld2
ENSMUSG00000020092	2.1364	-0.2626	DOWN	Pald1
ENSMUSG00000020131	1.621	-0.3694	DOWN	Pcsk4
ENSMUSG00000020287	1.8332	-0.3143	DOWN	Mpg
ENSMUSG00000020289	1.3846	-0.1818	DOWN	Nprl3
ENSMUSG00000020472	1.7862	-0.3124	DOWN	Zkscan17
ENSMUSG00000020474	1.4471	-0.2934	DOWN	Polm
ENSMUSG00000020538	3.6904	-0.2757	DOWN	Srebf1
ENSMUSG00000020733	3.7803	-0.3112	DOWN	Slc9a3r1
ENSMUSG00000020868	2.8524	-0.2556	DOWN	Xylt2
ENSMUSG00000020889	5.9079	-0.4331	DOWN	Nr1d1
ENSMUSG00000020893	3.0917	-0.475	DOWN	Per1
ENSMUSG00000021098	1.4415	-0.2293	DOWN	4930447C04Rik
ENSMUSG00000021136	1.5227	-0.2579	DOWN	Smoc1
ENSMUSG00000021179	1.8233	-0.3036	DOWN	Nrde2
ENSMUSG00000021180	1.5237	-0.2454	DOWN	Rps6ka5
ENSMUSG00000021215	3.1065	-0.4812	DOWN	Net1
ENSMUSG00000021357	2.2764	-0.2242	DOWN	Exoc2
ENSMUSG00000021432	1.4582	-0.2486	DOWN	Slc35b3
ENSMUSG00000021670	1.3455	-0.1489	DOWN	Hmgcr
ENSMUSG00000021685	1.7947	-0.2295	DOWN	Otp
ENSMUSG00000021763	2.4831	-0.4074	DOWN	Cspg4b
ENSMUSG00000021775	9.0787	-0.3027	DOWN	Nr1d2
ENSMUSG00000021898	1.3846	-0.6595	DOWN	Asb14
ENSMUSG00000021903	1.3736	-0.3893	DOWN	Galnt15
ENSMUSG00000021952	1.5191	-0.2467	DOWN	Xpo4
ENSMUSG00000022096	3.8712	-0.4282	DOWN	Hr
ENSMUSG00000022297	1.5273	-0.3515	DOWN	Fzd6
ENSMUSG00000022338	1.9929	-0.1988	DOWN	Eny2
ENSMUSG00000022389	2.8838	-0.1771	DOWN	Tef
ENSMUSG00000022562	1.3392	-0.2225	DOWN	Oplah
ENSMUSG00000022622	2.7208	-0.6888	DOWN	Acr
ENSMUSG00000022768	2.0894	-0.5054	DOWN	Ccdc116
ENSMUSG00000022797	7.625	-0.4889	DOWN	Tfrc
ENSMUSG00000022843	2.7427	-0.2328	DOWN	Clcn2
ENSMUSG00000024066	7.0898	-1.0708	DOWN	Xdh
ENSMUSG00000024327	1.9572	-0.6574	DOWN	Slc39a7
ENSMUSG00000024330	2.1602	-0.3828	DOWN	Col11a2

ENSMUSG00000024335	2.7477	-0.2055	DOWN	Brd2
ENSMUSG00000024493	1.8954	-0.2291	DOWN	Lars
ENSMUSG00000024750	2.0643	-0.1788	DOWN	Zfand5
ENSMUSG00000024831	1.3465	-0.2817	DOWN	Ighmbp2
ENSMUSG00000024835	2.4419	-0.2131	DOWN	Coro1b
ENSMUSG00000025086	5.2739	-0.3687	DOWN	Trub1
ENSMUSG00000025140	1.8069	-0.6275	DOWN	Pycr1
ENSMUSG00000025142	3.4376	-0.2731	DOWN	Aspscr1
ENSMUSG00000025159	2.6638	-0.2153	DOWN	Mms19
ENSMUSG00000025188	1.302	-0.186	DOWN	Hps1
ENSMUSG00000025324	1.9506	-0.4217	DOWN	Atp10a
ENSMUSG00000025407	1.6845	-0.3244	DOWN	Gli1
ENSMUSG00000025474	2.5988	-0.264	DOWN	Tubgcp2
ENSMUSG00000025485	1.863	-0.1812	DOWN	Ric8a
ENSMUSG00000025491	1.6872	-1.1169	DOWN	Ifitm1
ENSMUSG00000025586	1.9522	-0.1895	DOWN	Cpeb1
ENSMUSG00000025591	1.3411	-0.2705	DOWN	Tma16
ENSMUSG00000025792	1.7645	-0.2426	DOWN	Slc25a10
ENSMUSG00000026153	1.7174	-0.2216	DOWN	Fam135a
ENSMUSG00000026283	1.9415	-0.2698	DOWN	Ing5
ENSMUSG00000026409	2.2478	-0.2405	DOWN	Pfkfb2
ENSMUSG00000026489	2.4599	-0.2691	DOWN	Coq8a
ENSMUSG00000026556	1.3216	-0.1962	DOWN	Vangl2
ENSMUSG00000026675	1.7623	-0.2571	DOWN	Hsd17b7
ENSMUSG00000026694	1.9522	-0.2568	DOWN	Eef1aknmt
ENSMUSG00000026791	3.6785	-0.3628	DOWN	Slc2a8
ENSMUSG00000026843	1.6812	-0.1956	DOWN	Fubp3
ENSMUSG00000026930	2.0264	-0.1744	DOWN	Gpsm1
ENSMUSG00000026955	1.402	-0.5319	DOWN	Sapcd2
ENSMUSG00000026956	1.6863	-0.2337	DOWN	Uap1l1
ENSMUSG00000026977	1.8753	-0.1824	DOWN	Marchf7
ENSMUSG00000027006	3.7327	-0.2126	DOWN	Dnajc10
ENSMUSG00000027204	2.4177	-0.413	DOWN	Fbn1
ENSMUSG00000027346	1.6797	-0.2091	DOWN	Gpcpd1
ENSMUSG00000027408	3.5578	-0.5436	DOWN	Cpxm1
ENSMUSG00000027524	2.2855	-0.6214	DOWN	Edn3
ENSMUSG00000027566	1.3465	-0.1754	DOWN	Psma7
ENSMUSG00000028195	1.5564	-0.7548	DOWN	Ccn1
ENSMUSG00000028212	1.4135	-0.2874	DOWN	Ccne2
ENSMUSG00000028307	2.232	-0.5228	DOWN	Aldob
ENSMUSG00000028468	3.3135	-0.2525	DOWN	Rgp1
ENSMUSG00000028476	4.6784	-0.433	DOWN	Reck
ENSMUSG00000028568	2.2915	-0.2324	DOWN	Btf3l4
ENSMUSG00000028645	1.9016	-0.1796	DOWN	Slc2a1
ENSMUSG00000028862	1.5952	-0.6718	DOWN	Map3k6

ENSMUSG00000028878	5.1806	-0.3131	DOWN	Fam76a
ENSMUSG00000028957	12.2871	-0.5305	DOWN	Per3
ENSMUSG00000029009	2.1175	-0.2547	DOWN	Mthfr
ENSMUSG00000029022	1.9204	-0.2757	DOWN	Miip
ENSMUSG00000029049	2.0817	-0.4989	DOWN	Morn1
ENSMUSG00000029328	4.654	-0.268	DOWN	Hnrnpdl
ENSMUSG00000029513	1.3083	-0.2426	DOWN	Prkab1
ENSMUSG00000029570	1.9415	-0.2318	DOWN	Lfng
ENSMUSG00000029580	1.779	-0.1737	DOWN	Actb
ENSMUSG00000029602	1.3479	-0.2546	DOWN	Rasal1
ENSMUSG00000030087	1.4726	-0.2699	DOWN	Klf15
ENSMUSG00000030094	1.4732	-0.2618	DOWN	Xpc
ENSMUSG00000030096	2.5264	-0.2836	DOWN	Slc6a6
ENSMUSG00000030237	7.6586	-0.4086	DOWN	Slco1a4
ENSMUSG00000030254	1.8601	-0.3679	DOWN	Rad18
ENSMUSG00000030421	2.2839	-0.2506	DOWN	Uri1
ENSMUSG00000030539	2.1294	-0.2387	DOWN	Sema4b
ENSMUSG00000030747	6.1877	-0.4037	DOWN	Dgat2
ENSMUSG00000030757	5.7277	-0.424	DOWN	Zkscan2
ENSMUSG00000030795	1.8236	-0.143	DOWN	Fus
ENSMUSG00000031167	6.8613	-0.4818	DOWN	Rbm3
ENSMUSG00000031285	1.4337	-0.1953	DOWN	Dcx
ENSMUSG00000031323	1.9691	-0.4085	DOWN	Dmrtc1a
ENSMUSG00000031431	4.025	-0.3143	DOWN	Tsc22d3
ENSMUSG00000031938	1.8236	-0.2627	DOWN	4931406C07Rik
ENSMUSG00000031979	1.4783	-0.2101	DOWN	Cog2
ENSMUSG00000031986	1.3483	-0.2372	DOWN	Sprtn
ENSMUSG00000032010	5.1472	-0.3785	DOWN	Usp2
ENSMUSG00000032397	1.5607	-0.4014	DOWN	Tipin
ENSMUSG00000032410	1.8364	-0.2388	DOWN	Xrn1
ENSMUSG00000032515	2.3877	-0.6511	DOWN	Csrnp1
ENSMUSG00000032558	1.6783	-0.2882	DOWN	Nphp3
ENSMUSG00000032744	6.5523	-0.4654	DOWN	Heyl
ENSMUSG00000032940	6.1795	-0.5115	DOWN	Rbm11
ENSMUSG00000033105	1.5043	-0.2019	DOWN	Lss
ENSMUSG00000033106	2.2581	-0.2443	DOWN	Slc7a6os
ENSMUSG00000033166	1.4231	-0.2891	DOWN	Dis3
ENSMUSG00000033436	2.0313	-0.1764	DOWN	Armcx2
ENSMUSG00000033731	1.4883	-0.9278	DOWN	3300002A11Rik
ENSMUSG00000033857	1.3251	-0.2528	DOWN	Engase
ENSMUSG00000033863	5.2511	-0.3163	DOWN	Klf9
ENSMUSG00000033909	1.568	-0.2323	DOWN	Usp36
ENSMUSG00000034173	2.238	-0.332	DOWN	Zbed5
ENSMUSG00000034220	2.0849	-0.2183	DOWN	Gpc1
ENSMUSG00000034282	2.0874	-0.281	DOWN	Evpl

ENSMUSG00000034807	4.8113	-0.2686	DOWN	Colgalt1
ENSMUSG00000034858	2.4875	-0.3049	DOWN	Fam214a
ENSMUSG00000034898	1.4651	-0.4129	DOWN	Filip1
ENSMUSG00000035064	1.3504	-0.1734	DOWN	Eef2k
ENSMUSG00000035104	1.9394	-0.4428	DOWN	Eva1a
ENSMUSG00000035125	3.8025	-0.5864	DOWN	Gcfc2
ENSMUSG00000035696	1.3673	-0.1806	DOWN	Rnf38
ENSMUSG00000035824	4.2771	-0.2989	DOWN	Tk2
ENSMUSG00000035828	1.9375	-0.3901	DOWN	Pim3
ENSMUSG00000036181	1.6971	-0.3468	DOWN	H1f2
ENSMUSG00000036817	1.8113	-0.1855	DOWN	Sun1
ENSMUSG00000036916	1.3678	-0.2535	DOWN	Zfp280c
ENSMUSG00000037003	1.4339	-0.3327	DOWN	Tns2
ENSMUSG00000037089	1.552	-0.2789	DOWN	Slc35b2
ENSMUSG00000037266	3.5785	-0.3656	DOWN	Rsrp1
ENSMUSG00000037313	1.6971	-0.361	DOWN	Tacc3
ENSMUSG00000037337	1.9222	-0.4383	DOWN	Map4k1
ENSMUSG00000037344	2.4159	-0.2287	DOWN	Slc12a9
ENSMUSG00000037355	4.7841	-0.4613	DOWN	Uvssa
ENSMUSG00000037376	1.3717	-0.1901	DOWN	Trmt6
ENSMUSG00000037434	3.0799	-0.3078	DOWN	Slc30a1
ENSMUSG00000037709	1.3972	-0.2521	DOWN	Fam13a
ENSMUSG00000037808	1.6206	-0.2753	DOWN	Fam76b
ENSMUSG00000037813	1.8999	-0.3406	DOWN	D630003M21Rik
ENSMUSG00000038007	1.8332	-0.3481	DOWN	Acer2
ENSMUSG00000038026	1.3673	-0.2157	DOWN	Kcnj9
ENSMUSG00000038181	7.2358	-0.3913	DOWN	Chpf2
ENSMUSG00000038354	2.5414	-0.261	DOWN	Ankrd35
ENSMUSG00000038393	3.9599	-0.4999	DOWN	Txnip
ENSMUSG00000038550	13.7225	-0.8444	DOWN	Ciart
ENSMUSG00000038593	4.025	-0.2856	DOWN	Tctn1
ENSMUSG00000038685	3.6373	-0.2804	DOWN	Rtel1
ENSMUSG00000038776	1.7374	-0.2504	DOWN	Ephx1
ENSMUSG00000038777	1.3284	-0.2075	DOWN	Sema6c
ENSMUSG00000039461	2.7681	-0.306	DOWN	Tcta
ENSMUSG00000039765	1.3595	-0.2073	DOWN	Cc2d2a
ENSMUSG00000039781	5.048	-0.2767	DOWN	Cep131
ENSMUSG00000039838	1.3942	-0.1652	DOWN	Slc45a1
ENSMUSG00000039849	1.4705	-0.1944	DOWN	Pcif1
ENSMUSG00000040010	5.1845	-0.3344	DOWN	Slc7a5
ENSMUSG00000040170	1.7882	-1.2215	DOWN	Fmo2
ENSMUSG00000040272	2.6773	-0.3673	DOWN	Accs
ENSMUSG00000040478	1.395	-0.4127	DOWN	Prdm13
ENSMUSG00000040557	1.3942	-0.3555	DOWN	Mettl27
ENSMUSG00000040586	1.6692	-0.2927	DOWN	Ofd1

ENSMUSG00000040675	2.566	-0.3048	DOWN	Mthfd1l
ENSMUSG00000040701	2.1805	-0.3362	DOWN	Ap1g2
ENSMUSG00000040811	3.4376	-0.1874	DOWN	Eml2
ENSMUSG00000040820	1.4062	-0.2215	DOWN	Hlcs
ENSMUSG00000040857	2.5288	-0.37	DOWN	Erf
ENSMUSG00000040943	2.1201	-0.234	DOWN	Tet2
ENSMUSG00000040964	3.8339	-0.3109	DOWN	Arhgef10l
ENSMUSG00000041488	1.6797	-0.1989	DOWN	Stx3
ENSMUSG00000041598	1.4706	-0.2216	DOWN	Cdc42ep4
ENSMUSG00000041617	1.7454	-0.263	DOWN	Ccdc74a
ENSMUSG00000041679	3.2356	-0.7757	DOWN	Lrrc29
ENSMUSG00000042213	1.3259	-0.4143	DOWN	Zfand4
ENSMUSG00000042308	2.66	-0.3258	DOWN	Setd1a
ENSMUSG00000042354	2.21	-0.239	DOWN	Gnl3
ENSMUSG00000042510	1.4887	-0.4782	DOWN	AA986860
ENSMUSG00000042567	1.7151	-0.3232	DOWN	Nek10
ENSMUSG00000042644	1.3411	-0.5832	DOWN	Itpr3
ENSMUSG00000042810	2.9751	-0.2555	DOWN	Krba1
ENSMUSG00000042851	3.4594	-0.3583	DOWN	Zc3h6
ENSMUSG00000042942	2.349	-0.3873	DOWN	Greb1l
ENSMUSG00000042997	2.4801	-0.3576	DOWN	Nhlrc3
ENSMUSG00000043833	1.3148	-0.4156	DOWN	2900005J15Rik
ENSMUSG00000044197	4.5368	-0.346	DOWN	Gpr146
ENSMUSG00000044229	1.7831	-0.2881	DOWN	Nxpe4
ENSMUSG00000044345	2.349	-0.3632	DOWN	Marveld1
ENSMUSG00000044576	1.528	-0.2604	DOWN	Garem2
ENSMUSG00000044636	1.3999	-0.2165	DOWN	Csrnp2
ENSMUSG00000044730	1.6107	-0.2227	DOWN	9930104L06Rik
ENSMUSG00000045193	10.1722	-0.5689	DOWN	Cirbp
ENSMUSG00000045294	2.0692	-0.2323	DOWN	Insig1
ENSMUSG00000045322	1.6886	-0.6763	DOWN	Tlr9
ENSMUSG00000045411	2.1422	-0.196	DOWN	2410002F23Rik
ENSMUSG00000045466	1.3107	-0.2357	DOWN	Zfp956
ENSMUSG00000045795	2.1144	-0.273	DOWN	Whamm
ENSMUSG00000045838	2.703	-0.3783	DOWN	Ccdc9b
ENSMUSG00000045954	1.4453	-0.2939	DOWN	Cavin2
ENSMUSG00000046546	1.3846	-0.2707	DOWN	Fam43a
ENSMUSG00000046605	1.9162	-0.4006	DOWN	B3gntl1
ENSMUSG00000046610	2.5942	-0.9134	DOWN	Oacyl
ENSMUSG00000046792	1.7012	-0.2506	DOWN	Zfp787
ENSMUSG00000047330	1.9384	-0.923	DOWN	Kcne4
ENSMUSG00000047617	1.4763	-0.2396	DOWN	Paxx
ENSMUSG00000047909	3.0154	-0.3553	DOWN	Ankrd16
ENSMUSG00000048330	1.5952	-0.176	DOWN	Ric3
ENSMUSG00000048371	1.8624	-0.2972	DOWN	Pdp2

ENSMUSG00000048445	2.0849	-0.3112	DOWN	Ccdc57
ENSMUSG00000048930	1.6458	-0.2452	DOWN	Tada3
ENSMUSG00000049562	1.4889	-0.5024	DOWN	Ap5b1
ENSMUSG00000049672	1.4287	-0.1838	DOWN	Zbtb14
ENSMUSG00000050211	16.8666	-0.8846	DOWN	Pla2g4e
ENSMUSG00000050312	3.7439	-0.4653	DOWN	Nsun3
ENSMUSG00000050373	1.3595	-0.17	DOWN	Snx21
ENSMUSG00000050721	2.2508	-0.3019	DOWN	Plekho2
ENSMUSG00000051098	4.9998	-0.2953	DOWN	Mblac2
ENSMUSG00000051113	3.3562	-0.4825	DOWN	Fam71e1
ENSMUSG00000051343	1.3386	-0.1851	DOWN	Rab11fip5
ENSMUSG00000051351	5.1582	-0.5216	DOWN	Zfp46
ENSMUSG00000051390	2.2782	-0.2856	DOWN	Zbtb22
ENSMUSG00000051504	1.7713	-0.3753	DOWN	Siglech
ENSMUSG00000052392	2.5436	-0.7773	DOWN	Acot4
ENSMUSG00000052566	1.3216	-0.1682	DOWN	Hook2
ENSMUSG00000052676	1.303	-0.1674	DOWN	Zmat1
ENSMUSG00000052751	1.3377	-0.1701	DOWN	Repin1
ENSMUSG00000053914	1.9922	-0.8343	DOWN	Kdm4d
ENSMUSG00000054474	1.5478	-0.3533	DOWN	Thnsl2
ENSMUSG00000054717	1.4156	-0.4276	DOWN	Hmgb2
ENSMUSG00000054871	2.3899	-0.4028	DOWN	Tmem158
ENSMUSG00000054901	8.2765	-0.9584	DOWN	Arhgef33
ENSMUSG00000055493	3.3135	-0.2501	DOWN	Epm2a
ENSMUSG00000055629	2.5444	-0.2269	DOWN	B4galnt4
ENSMUSG00000055866	2.4571	-0.3275	DOWN	Per2
ENSMUSG00000056952	1.3868	-0.2046	DOWN	Tatdn2
ENSMUSG00000057858	1.4407	-0.2485	DOWN	Fam204a
ENSMUSG00000059743	1.7384	-0.8162	DOWN	Fdps
ENSMUSG00000059824	46.8995	-1.156	DOWN	Dbp
ENSMUSG00000059866	2.8013	-0.2707	DOWN	Tnip2
ENSMUSG00000061455	1.4003	-0.2817	DOWN	Stx17
ENSMUSG00000062044	1.4047	-0.2475	DOWN	Lmtk3
ENSMUSG00000062488	2.2581	-0.5267	DOWN	Ifit3b
ENSMUSG00000062822	1.3849	-0.1912	DOWN	4833420G17Rik
ENSMUSG00000062825	1.3628	-0.1867	DOWN	Actg1
ENSMUSG00000062861	1.7862	-0.2722	DOWN	Zfp28
ENSMUSG00000063108	2.5523	-0.3023	DOWN	Zfp26
ENSMUSG00000063558	2.3768	-0.489	DOWN	Aox1
ENSMUSG00000063887	1.4581	-0.1686	DOWN	Nlgn1
ENSMUSG00000064145	1.7965	-0.1788	DOWN	Arih2
ENSMUSG00000066245	2.0976	-1.189	DOWN	Gm10156
ENSMUSG00000066554	2.87	-0.6993	DOWN	Gm10167
ENSMUSG00000066829	2.1939	-0.3225	DOWN	Zfp810
ENSMUSG00000067942	1.5607	-0.2284	DOWN	Zfp160

ENSMUSG00000068154	1.6107	-0.3376	DOWN	Insm1
ENSMUSG00000068742	2.9818	-0.2091	DOWN	Cry2
ENSMUSG00000069045	2.1111	-0.24	DOWN	Ddx3y
ENSMUSG00000069835	1.4182	-0.3171	DOWN	Sat2
ENSMUSG00000070420	2.0874	-0.3415	DOWN	Zscan25
ENSMUSG00000070604	2.0476	-0.4648	DOWN	Vsig10l
ENSMUSG00000070729	1.6863	-0.3808	DOWN	Gm12966
ENSMUSG00000070803	1.354	-0.4863	DOWN	Cited4
ENSMUSG00000071076	1.3411	-0.2051	DOWN	Jund
ENSMUSG00000071253	2.7143	-0.3081	DOWN	Slc25a16
ENSMUSG00000071477	1.4551	-0.1752	DOWN	Zfp777
ENSMUSG00000071656	1.4789	-0.5548	DOWN	Lrrn4cl
ENSMUSG00000071665	1.3465	-0.4848	DOWN	Foxr2
ENSMUSG00000071691	1.6739	-0.5079	DOWN	Gm960
ENSMUSG00000071847	4.1399	-0.3361	DOWN	Apcdd1
ENSMUSG00000072487	1.4783	-0.917	DOWN	Mroh5
ENSMUSG00000072653	3.3135	-0.2735	DOWN	Zfp783
ENSMUSG00000072969	5.5132	-0.4773	DOWN	Armcx5
ENSMUSG00000073406	1.6174	-0.5805	DOWN	H2-Bl
ENSMUSG00000073436	1.4264	-0.3348	DOWN	Eme2
ENSMUSG00000073600	1.5632	-0.3024	DOWN	Prob1
ENSMUSG00000073779	2.0476	-0.4818	DOWN	Lrp8os2
ENSMUSG00000073792	1.3606	-0.2464	DOWN	Alg6
ENSMUSG00000074165	1.4042	-0.1725	DOWN	Zfp788
ENSMUSG00000074252	2.2899	-0.455	DOWN	Gm10654
ENSMUSG00000074896	3.9733	-0.6055	DOWN	Ifit3
ENSMUSG00000075225	2.0254	-0.6821	DOWN	Ccdc162
ENSMUSG00000078202	4.5368	-0.6145	DOWN	Nrarp
ENSMUSG00000079109	2.086	-0.3165	DOWN	Pms2
ENSMUSG00000079610	1.3383	-0.2998	DOWN	Ankrd39
ENSMUSG00000079654	2.7681	-0.4895	DOWN	Prrt4
ENSMUSG00000080316	2.3768	-0.2783	DOWN	Spaca6
ENSMUSG00000085148	2.0704	-0.389	DOWN	Mir22hg
ENSMUSG00000085442	1.3218	-0.3462	DOWN	Gm3362
ENSMUSG00000085873	1.3157	-0.2644	DOWN	Ttc39aos1
ENSMUSG00000087523	1.4156	-0.3983	DOWN	Gm12319
ENSMUSG00000089736	1.7733	-0.3811	DOWN	Tgfbr3l
ENSMUSG00000090125	1.5142	-0.456	DOWN	Pou3f1
ENSMUSG00000090291	1.3411	-0.3956	DOWN	Lrrc10b
ENSMUSG00000091345	1.3628	-0.4812	DOWN	Col6a5
ENSMUSG00000091994	2.537	-0.4835	DOWN	E130317F20Rik
ENSMUSG00000096966	1.3377	-0.6283	DOWN	Gm18336
ENSMUSG00000097023	3.5883	-0.4111	DOWN	Mir9-3hg
ENSMUSG00000097133	1.6035	-1.0056	DOWN	Gm26628
ENSMUSG00000097413	2.4599	-0.9589	DOWN	A830052D11Rik

ENSMUSG00000097622	4.7841	-1.2191	DOWN	A330033J07Rik
ENSMUSG00000098912	1.3846	-0.1683	DOWN	1500004A13Rik
ENSMUSG00000101995	1.6749	-0.389	DOWN	Gm29480
ENSMUSG00000102729	1.7882	-0.2784	DOWN	Gm32444
ENSMUSG00000103622	1.653	-0.7956	DOWN	B430319G15Rik
ENSMUSG00000104362	1.4783	-0.2727	DOWN	Gm37928
ENSMUSG00000105881	2.1645	-0.5211	DOWN	4932422M17Rik
ENSMUSG00000106535	1.7871	-0.6813	DOWN	Gm43031
ENSMUSG00000106951	1.4174	-0.6887	DOWN	5930430L01Rik
ENSMUSG00000110016	1.3124	-0.4873	DOWN	Gm20751
ENSMUSG00000110638	1.4789	-0.8489	DOWN	Gm45887
ENSMUSG00000116138	1.7454	-0.2242	DOWN	C030006K11Rik
ENSMUSG00000116180	1.7067	-0.3192	DOWN	Gm49492
ENSMUSG00000116946	1.6812	-0.7856	DOWN	Gm41442
ENSMUSG00000117322	1.3519	-0.944	DOWN	6330415G19Rik
ENSMUSG00000117679	2.4947	-0.2588	DOWN	Apbb3
ENSMUSG00000118668	1.6246	-0.2419	DOWN	Rps6ka4
ENSMUSG00000120774	1.4006	-0.6108	DOWN	ENSMUSG00000120774
ENSMUSG00000121107	1.9179	-0.4661	DOWN	9330133O14Rik
ENSMUSG00000121137	2.349	-0.7088	DOWN	ENSMUSG00000121137
ENSMUSG00000000378	1.7947	0.2241	UP	Ccm2
ENSMUSG00000000957	9.8369	0.6377	UP	Mmp14
ENSMUSG00000001089	1.4115	0.342	UP	Luzp1
ENSMUSG00000001128	2.4768	0.3563	UP	Cfp
ENSMUSG00000001143	2.413	0.2922	UP	Lman2l
ENSMUSG00000001229	1.716	0.2026	UP	Dpp9
ENSMUSG00000001441	6.6413	0.2789	UP	Npepps
ENSMUSG00000001552	2.197	0.2611	UP	Jup
ENSMUSG00000001700	4.7176	0.4658	UP	Gramd3
ENSMUSG00000001774	9.2281	0.6218	UP	Chordc1
ENSMUSG00000001829	1.3962	0.1657	UP	Clpb
ENSMUSG00000002416	3.9879	0.312	UP	Ndufb2
ENSMUSG00000002428	1.9572	0.2727	UP	Hltf
ENSMUSG00000002845	2.4084	0.3221	UP	Tmem39a
ENSMUSG00000003184	4.7227	0.3678	UP	Irf3
ENSMUSG00000003273	2.1468	0.2052	UP	Car11
ENSMUSG00000003546	1.4705	0.172	UP	Klc4
ENSMUSG00000003814	6.5235	0.3367	UP	Calr
ENSMUSG00000004360	2.2682	0.1995	UP	9330159F19Rik
ENSMUSG00000004460	2.5339	0.3072	UP	Dnajb11
ENSMUSG00000004748	1.4789	0.2381	UP	Mtfp1
ENSMUSG00000004980	2.2776	0.175	UP	Hnrnpa2b1
ENSMUSG00000005483	3.1288	0.4035	UP	Dnajb1
ENSMUSG00000005899	1.8695	0.2195	UP	Smpd4
ENSMUSG00000005958	2.7387	0.3856	UP	Ephb3

ENSMUSG00000006205	9.2832	0.3821	UP	Htra1
ENSMUSG00000006456	3.1597	0.3605	UP	Rbm14
ENSMUSG00000007617	1.789	0.3048	UP	Homer1
ENSMUSG00000007646	2.2478	0.5071	UP	Rad51c
ENSMUSG00000007656	2.0194	0.1939	UP	Arpp19
ENSMUSG00000007721	1.4054	0.2582	UP	Ccdc124
ENSMUSG00000009013	3.1782	0.279	UP	Dynll1
ENSMUSG00000010529	3.0128	0.5456	UP	Gm266
ENSMUSG00000010554	10.1303	0.4419	UP	Mettl16
ENSMUSG00000011589	1.8695	0.2054	UP	Fsd1
ENSMUSG00000012296	2.566	0.3638	UP	Tjap1
ENSMUSG00000012609	1.8085	0.2178	UP	Ttll5
ENSMUSG00000013089	6.6138	0.3955	UP	Etv5
ENSMUSG00000013150	3.4965	0.3273	UP	Gfod2
ENSMUSG00000014039	1.3661	0.2858	UP	Prdm15
ENSMUSG00000014177	2.0791	0.2331	UP	Tvp23b
ENSMUSG00000014226	1.9638	0.2422	UP	Cacybp
ENSMUSG00000014551	1.4012	0.2377	UP	Mrps25
ENSMUSG00000015224	2.6401	0.2301	UP	Cyp2j9
ENSMUSG00000015305	1.3688	0.247	UP	Sash1
ENSMUSG00000015476	3.2522	0.2957	UP	Prrt1
ENSMUSG00000015484	1.3824	0.4553	UP	Fam163a
ENSMUSG00000015656	6.8613	0.2848	UP	Hspa8
ENSMUSG00000015659	1.3216	0.2645	UP	Serac1
ENSMUSG00000015748	2.4768	0.2923	UP	Prpf3
ENSMUSG00000015869	1.4582	0.174	UP	Prpsap1
ENSMUSG00000016018	4.65	0.2765	UP	Mtrex
ENSMUSG00000016481	1.4686	0.2548	UP	Cr1l
ENSMUSG00000016664	3.293	0.2576	UP	Pacsin2
ENSMUSG00000017421	2.0924	0.1927	UP	Zfp207
ENSMUSG00000017724	2.2891	0.4247	UP	Etv4
ENSMUSG00000018167	2.9932	0.2868	UP	Stard3
ENSMUSG00000018398	1.7947	0.1545	UP	Septin8
ENSMUSG00000018634	2.1432	0.3246	UP	Crhr1
ENSMUSG00000018727	3.9915	0.8246	UP	Cpsf4l
ENSMUSG00000018849	1.7871	0.2352	UP	Wwc1
ENSMUSG00000018906	1.8756	0.419	UP	P4ha2
ENSMUSG00000019039	1.5809	0.1955	UP	Dalrd3
ENSMUSG00000019055	1.8047	0.2862	UP	Plod1
ENSMUSG00000019359	1.3994	0.2343	UP	Gdpd2
ENSMUSG00000019659	2.2919	0.2893	UP	Ccdc12
ENSMUSG00000019734	2.464	0.3411	UP	Tmc4
ENSMUSG00000019763	3.1782	0.346	UP	Rmnd1
ENSMUSG00000019804	1.7659	0.2113	UP	Snx3
ENSMUSG00000019874	16.9475	0.9249	UP	Fabp7

ENSMUSG00000019916	9.2512	0.6527	UP	P4ha1
ENSMUSG00000019969	1.3899	0.1603	UP	Psen1
ENSMUSG00000020048	3.2522	0.2513	UP	Hsp90b1
ENSMUSG00000020069	1.899	0.1991	UP	Hnrnph3
ENSMUSG00000020257	1.6992	0.1651	UP	Wdr82
ENSMUSG00000020288	4.4376	0.5037	UP	Ahsa2
ENSMUSG00000020290	2.723	0.2285	UP	Xpo1
ENSMUSG00000020361	2.1492	0.1767	UP	Hspa4
ENSMUSG00000020484	3.4376	0.2985	UP	Xbp1
ENSMUSG00000020513	3.0864	0.6562	UP	Tubd1
ENSMUSG00000020571	5.0131	0.3556	UP	Pdia6
ENSMUSG00000020580	2.1	0.1952	UP	Rock2
ENSMUSG00000020610	1.7552	0.1974	UP	Amz2
ENSMUSG00000020674	1.5043	0.2729	UP	Pxdn
ENSMUSG00000020841	1.9638	0.211	UP	Cpd
ENSMUSG00000020895	2.0976	0.4851	UP	Tmem107
ENSMUSG00000020935	1.4415	0.1899	UP	Dcakd
ENSMUSG00000021037	3.5655	0.2832	UP	Ahsa1
ENSMUSG00000021071	18.5421	0.3818	UP	Trim9
ENSMUSG00000021111	2.0976	0.1766	UP	Papola
ENSMUSG00000021116	1.4763	0.1968	UP	Eif2s1
ENSMUSG00000021196	2.3376	0.1871	UP	Pfkp
ENSMUSG00000021270	2.5277	0.2093	UP	Hsp90aa1
ENSMUSG00000021327	1.3554	0.1987	UP	Zkscan3
ENSMUSG00000021413	1.3361	0.1602	UP	Prpf4b
ENSMUSG00000021431	1.3377	0.1905	UP	Snrnp48
ENSMUSG00000021555	1.6183	0.1704	UP	Naa35
ENSMUSG00000021686	1.3956	0.1856	UP	Ap3b1
ENSMUSG00000021692	1.4731	0.3004	UP	Dimt1
ENSMUSG00000021725	1.8528	0.2021	UP	Parp8
ENSMUSG00000021758	1.7882	0.6006	UP	Ddx4
ENSMUSG00000021772	1.6368	0.236	UP	Nkiras1
ENSMUSG00000022054	1.7733	0.2822	UP	Nefm
ENSMUSG00000022132	1.4256	0.2435	UP	Cldn10
ENSMUSG00000022136	2.501	0.2584	UP	Dnajc3
ENSMUSG00000022160	4.7841	0.4049	UP	Mettl3
ENSMUSG00000022185	1.3465	0.1919	UP	Acin1
ENSMUSG00000022191	1.8047	0.1453	UP	Drosha
ENSMUSG00000022200	1.7016	0.178	UP	Golph3
ENSMUSG00000022234	1.4705	0.1648	UP	Cct5
ENSMUSG00000022332	4.6368	0.4094	UP	Khdrbs3
ENSMUSG00000022360	1.3099	0.3902	UP	Atad2
ENSMUSG00000022376	2.0873	0.2246	UP	Adcy8
ENSMUSG00000022403	2.6685	0.2208	UP	St13
ENSMUSG00000022505	1.5676	0.2622	UP	Emp2

ENSMUSG00000022537	2.8446	0.3489	UP	Tmem44
ENSMUSG00000022556	1.3457	0.1687	UP	Hsf1
ENSMUSG00000022621	2.8008	0.2324	UP	Rabl2
ENSMUSG00000022639	1.9375	0.399	UP	Dubr
ENSMUSG00000022663	1.7366	0.2388	UP	Atg3
ENSMUSG00000022678	1.8874	0.2961	UP	Nde1
ENSMUSG00000022769	9.4902	0.8574	UP	Sdf2l1
ENSMUSG00000022957	1.4638	0.1923	UP	Itsn1
ENSMUSG00000023020	1.659	0.2807	UP	Cox14
ENSMUSG00000023067	1.3849	0.4159	UP	Cdkn1a
ENSMUSG00000023272	11.9466	0.6522	UP	Creld2
ENSMUSG00000023932	1.8971	0.2124	UP	Cdc5l
ENSMUSG00000023939	1.3824	0.2508	UP	Mrpl14
ENSMUSG00000023944	1.5022	0.1876	UP	Hsp90ab1
ENSMUSG00000023994	4.166	0.4665	UP	Nfya
ENSMUSG00000024135	1.4959	0.2784	UP	Srbd1
ENSMUSG00000024136	2.487	0.6108	UP	Dnase1l2
ENSMUSG00000024269	1.3273	0.1829	UP	Tpgs2
ENSMUSG00000024376	1.3091	0.2491	UP	Epb41l4a
ENSMUSG00000024384	1.9144	0.2021	UP	Iws1
ENSMUSG00000024426	2.5491	0.2354	UP	Atat1
ENSMUSG00000024480	1.7442	0.2235	UP	Ap3s1
ENSMUSG00000024524	1.6067	0.1666	UP	Gnal
ENSMUSG00000024781	1.3745	0.2106	UP	Lipa
ENSMUSG00000024793	1.402	0.718	UP	Tnfrsf25
ENSMUSG00000024817	5.6542	0.3568	UP	Uhrf2
ENSMUSG00000024843	1.3231	0.1613	UP	Chka
ENSMUSG00000024921	1.5183	0.1516	UP	Smarca2
ENSMUSG00000024966	9.8369	0.4895	UP	Stip1
ENSMUSG00000024998	1.3479	0.1984	UP	Plce1
ENSMUSG00000025066	1.5478	0.2151	UP	Sfr1
ENSMUSG00000025083	2.1837	0.2905	UP	Afap1l2
ENSMUSG00000025175	3.4248	0.3251	UP	Fn3k
ENSMUSG00000025224	1.8161	0.1974	UP	Gbf1
ENSMUSG00000025232	1.8331	0.2554	UP	Hexa
ENSMUSG00000025283	2.9258	0.2462	UP	Sat1
ENSMUSG00000025316	4.0577	0.5301	UP	Banp
ENSMUSG00000025413	2.7885	0.2342	UP	Ttc4
ENSMUSG00000025473	1.4982	0.5709	UP	Adam8
ENSMUSG00000025574	2.4753	0.6873	UP	Tk1
ENSMUSG00000025757	1.7886	0.2427	UP	Hspa4l
ENSMUSG00000025823	4.9649	0.3875	UP	Pdia4
ENSMUSG00000025939	1.7672	0.197	UP	Ube2w
ENSMUSG00000025958	3.5944	0.3586	UP	Creb1
ENSMUSG00000025978	1.3392	0.2024	UP	Rftn2

ENSMUSG00000025980	1.7268	0.1839	UP	Hspd1
ENSMUSG00000026005	1.355	0.2319	UP	Rpe
ENSMUSG00000026014	2.0254	0.2372	UP	Raph1
ENSMUSG00000026074	2.232	0.2277	UP	Map4k4
ENSMUSG00000026080	1.3376	0.152	UP	Chst10
ENSMUSG00000026172	2.4753	0.306	UP	Bcs1l
ENSMUSG00000026173	1.4188	0.2251	UP	Plcd4
ENSMUSG00000026203	2.5739	0.1886	UP	Dnajb2
ENSMUSG00000026239	2.9741	0.3538	UP	Pde6d
ENSMUSG00000026254	1.659	0.1816	UP	Eif4e2
ENSMUSG00000026439	2.3862	0.2096	UP	Rbbp5
ENSMUSG00000026566	1.4582	0.1936	UP	Mpzl1
ENSMUSG00000026578	1.5979	0.2366	UP	Ccdc181
ENSMUSG00000026617	1.621	0.1696	UP	Bpnt1
ENSMUSG00000026737	2.3958	0.2719	UP	Pip4k2a
ENSMUSG00000026826	3.1369	0.408	UP	Nr4a2
ENSMUSG00000026831	2.2581	0.5	UP	1700007K13Rik
ENSMUSG00000026864	4.9998	0.3309	UP	Hspa5
ENSMUSG00000026941	1.4783	0.4293	UP	Mamdc4
ENSMUSG00000026999	1.6801	0.3281	UP	Nup35
ENSMUSG00000027166	1.4155	0.3292	UP	Dnajc24
ENSMUSG00000027184	3.4144	0.2456	UP	Caprin1
ENSMUSG00000027230	1.3251	0.3326	UP	Creb3l1
ENSMUSG00000027238	2.6316	0.219	UP	Frmd5
ENSMUSG00000027259	1.7454	0.2645	UP	Adal
ENSMUSG00000027274	5.7443	0.6061	UP	Mkks
ENSMUSG00000027333	3.3547	0.2302	UP	Smox
ENSMUSG00000027351	3.6571	0.2526	UP	Spred1
ENSMUSG00000027424	4.8159	0.552	UP	Mgme1
ENSMUSG00000027435	1.9506	0.4122	UP	Cd93
ENSMUSG00000027534	2.422	0.305	UP	Snx16
ENSMUSG00000027677	1.8912	0.165	UP	Ttc14
ENSMUSG00000027680	3.165	0.2406	UP	Fxr1
ENSMUSG00000027804	1.9365	0.2345	UP	Ppid
ENSMUSG00000027854	1.3043	0.1623	UP	Sike1
ENSMUSG00000027881	1.5431	0.1833	UP	Prpf38b
ENSMUSG00000028030	1.3942	0.2165	UP	Tbck
ENSMUSG00000028035	4.1217	0.2751	UP	Dnajb4
ENSMUSG00000028057	1.9522	0.2302	UP	Rit1
ENSMUSG00000028096	1.4003	0.2347	UP	Gpr89
ENSMUSG00000028221	1.7862	0.1895	UP	Pip4p2
ENSMUSG00000028243	1.3927	0.1953	UP	Ubxn2b
ENSMUSG00000028261	1.6144	0.2031	UP	Ndufaf4
ENSMUSG00000028274	1.6626	0.2142	UP	Rngtt
ENSMUSG00000028343	1.4096	0.1705	UP	Erp44

ENSMUSG00000028383	1.7715	0.2149	UP	Hsdl2
ENSMUSG00000028402	4.6784	0.3116	UP	Mpdz
ENSMUSG00000028410	4.0977	0.2998	UP	Dnaja1
ENSMUSG00000028527	1.552	0.1914	UP	Ak4
ENSMUSG00000028608	1.518	0.1771	UP	Czib
ENSMUSG00000028613	1.4763	0.1948	UP	Lrp8
ENSMUSG00000028656	1.8367	0.1631	UP	Cap1
ENSMUSG00000028820	12.1364	0.5279	UP	Sfpq
ENSMUSG00000028851	2.87	0.2001	UP	Nudc
ENSMUSG00000028899	2.2541	0.3219	UP	Taf12
ENSMUSG00000029106	2.232	0.1564	UP	Add1
ENSMUSG00000029283	7.4637	0.4311	UP	Cdc7
ENSMUSG00000029410	3.6879	0.8705	UP	Ppef2
ENSMUSG00000029534	1.4231	0.291	UP	St7
ENSMUSG00000029608	1.3129	0.1763	UP	Rph3a
ENSMUSG00000029657	6.1795	0.5213	UP	Hsph1
ENSMUSG00000029701	9.1737	0.4899	UP	Rbm28
ENSMUSG00000029763	1.4582	0.1769	UP	Exoc4
ENSMUSG00000029776	1.3846	0.1655	UP	Hibadh
ENSMUSG00000029817	8.6862	0.3768	UP	Tra2a
ENSMUSG00000029916	1.3289	0.2293	UP	Agk
ENSMUSG00000030002	3.2439	0.244	UP	Dusp11
ENSMUSG00000030257	1.7977	0.2053	UP	Srgap3
ENSMUSG00000030279	2.0476	0.1714	UP	C2cd5
ENSMUSG00000030352	1.4253	0.2172	UP	Tspan9
ENSMUSG00000030357	4.0464	0.3707	UP	Fkbp4
ENSMUSG00000030549	1.9436	0.9003	UP	Rhcg
ENSMUSG00000030629	2.1492	0.2202	UP	Zfand6
ENSMUSG00000030655	1.5043	0.192	UP	Smg1
ENSMUSG00000030878	1.5578	0.2332	UP	Cdr2
ENSMUSG00000030898	1.4463	0.3252	UP	Cckbr
ENSMUSG00000031060	2.4081	0.2419	UP	Rbm10
ENSMUSG00000031068	1.4966	0.2167	UP	Glrx3
ENSMUSG00000031245	1.7454	0.3381	UP	Hmgn5
ENSMUSG00000031385	1.5443	0.3611	UP	Plxnb3
ENSMUSG00000031513	2.4419	0.1788	UP	Leprotl1
ENSMUSG00000031527	1.5578	0.3367	UP	Eri1
ENSMUSG00000031585	2.6205	0.3759	UP	Gtf2e2
ENSMUSG00000031661	1.3012	0.2768	UP	Nkd1
ENSMUSG00000031667	1.5016	0.1989	UP	Aktip
ENSMUSG00000031760	2.7246	0.2959	UP	Mt3
ENSMUSG00000031785	1.3595	0.1452	UP	Adgrg1
ENSMUSG00000031826	1.5742	0.1683	UP	Usp10
ENSMUSG00000031885	1.7279	0.2119	UP	Cbfb
ENSMUSG00000032026	2.7771	0.2574	UP	Rexo2

ENSMUSG00000032050	1.4463	0.1469	UP	Rdx
ENSMUSG00000032060	3.6912	0.3956	UP	Cryab
ENSMUSG00000032187	2.5436	0.1967	UP	Smarca4
ENSMUSG00000032249	2.0998	0.2233	UP	Anp32a
ENSMUSG00000032285	2.0239	0.3401	UP	Dnaja4
ENSMUSG00000032349	2.1787	0.212	UP	Elovl5
ENSMUSG00000032360	1.7862	0.3468	UP	Hcrtr2
ENSMUSG00000032475	1.3878	0.199	UP	Nck1
ENSMUSG00000032556	3.293	0.6615	UP	Bfsp2
ENSMUSG00000032570	1.8244	0.1717	UP	Atp2c1
ENSMUSG00000032575	4.1399	0.4334	UP	Manf
ENSMUSG00000032582	2.3819	0.2267	UP	Rbm6
ENSMUSG00000032621	1.5971	0.1943	UP	Srek1
ENSMUSG00000032702	1.7645	0.2698	UP	Kank1
ENSMUSG00000032705	4.049	0.3756	UP	Exd2
ENSMUSG00000032766	1.5676	0.339	UP	Gng11
ENSMUSG00000032905	1.4783	0.1676	UP	Atg12
ENSMUSG00000032908	1.4331	0.3552	UP	Sgpp2
ENSMUSG00000033282	1.3242	0.2613	UP	Rpgrip1l
ENSMUSG00000033316	2.1896	0.2335	UP	Galnt9
ENSMUSG00000033885	1.6971	0.1992	UP	Pxk
ENSMUSG00000035126	1.8695	0.3049	UP	Dnai4
ENSMUSG00000035284	1.4118	0.1957	UP	Vps13c
ENSMUSG00000035401	1.4783	0.2043	UP	Emsy
ENSMUSG00000035575	2.4599	0.218	UP	Utp6
ENSMUSG00000035649	3.9915	0.2359	UP	Zcchc7
ENSMUSG00000035711	3.4144	0.6313	UP	Dok3
ENSMUSG00000035722	1.6083	0.2835	UP	Abca7
ENSMUSG00000035759	1.3851	0.3486	UP	Bbs10
ENSMUSG00000035868	1.6863	0.2258	UP	Zfp983
ENSMUSG00000035877	1.4582	0.1761	UP	Zhx3
ENSMUSG00000035898	9.8369	0.5859	UP	Uba6
ENSMUSG00000036078	1.3129	0.196	UP	Sigmar1
ENSMUSG00000036398	1.6311	0.2503	UP	Ppp1r11
ENSMUSG00000036580	1.7977	0.2017	UP	Spg20
ENSMUSG00000036864	1.3609	0.4546	UP	Proser3
ENSMUSG00000036879	1.4256	0.1697	UP	Phkb
ENSMUSG00000036932	1.6183	0.263	UP	Aifm1
ENSMUSG00000037010	7.2902	0.4641	UP	Apln
ENSMUSG00000037016	2.0239	0.7502	UP	Frem2
ENSMUSG00000037022	1.6332	0.2284	UP	Mmaa
ENSMUSG00000037058	2.0874	0.1946	UP	Paip2
ENSMUSG00000037243	2.9286	0.2327	UP	Zfp692
ENSMUSG00000037348	1.8085	0.152	UP	Paqr7
ENSMUSG00000037363	1.4054	0.2165	UP	Letm2

ENSMUSG00000037364	1.4711	0.1663	UP	Srrt
ENSMUSG00000037432	2.5444	0.3733	UP	Fer1l5
ENSMUSG00000037455	1.3661	0.2476	UP	Slc18b1
ENSMUSG00000037669	1.8069	0.214	UP	Ldah
ENSMUSG00000037712	1.8332	0.1649	UP	Fermt2
ENSMUSG00000037720	1.844	0.1886	UP	Tmem33
ENSMUSG00000037747	1.7691	0.187	UP	Phyhipl
ENSMUSG00000037788	1.4582	0.1571	UP	Vopp1
ENSMUSG00000037935	2.3764	0.2097	UP	Smarce1
ENSMUSG00000037990	1.3717	0.3371	UP	Sh3rf3
ENSMUSG00000038028	1.3959	0.2695	UP	Tigar
ENSMUSG00000038366	1.764	0.1839	UP	Lasp1
ENSMUSG00000038497	1.8695	0.1821	UP	Tmco3
ENSMUSG00000039065	2.2767	0.4778	UP	Atpsckmt
ENSMUSG00000039089	1.8544	0.2244	UP	L3mbtl3
ENSMUSG00000039254	1.4003	0.2233	UP	Pomt1
ENSMUSG00000039599	1.617	0.2154	UP	Fam149b
ENSMUSG00000039680	1.6559	0.2796	UP	Mrps6
ENSMUSG00000039682	2.1109	0.2236	UP	Lap3
ENSMUSG00000039804	2.4571	0.2769	UP	Ncoa5
ENSMUSG00000039830	2.703	0.2638	UP	Olig2
ENSMUSG00000039943	1.3846	0.1381	UP	Plcb4
ENSMUSG00000039982	2.5092	0.2527	UP	Dtx4
ENSMUSG00000040029	1.5273	0.1703	UP	Ipo8
ENSMUSG00000040242	1.5183	0.1719	UP	Fgfr1op2
ENSMUSG00000040260	1.3251	0.231	UP	Daam2
ENSMUSG00000040724	1.7472	0.2094	UP	Kcna2
ENSMUSG00000040738	7.0718	0.486	UP	Ints8
ENSMUSG00000040774	2.1615	0.2121	UP	Cept1
ENSMUSG00000041119	1.8123	0.2303	UP	Pde9a
ENSMUSG00000041278	1.3377	0.19	UP	Ttc1
ENSMUSG00000041298	1.4047	0.1842	UP	Katnal1
ENSMUSG00000041360	1.3129	0.2265	UP	Pum3
ENSMUSG00000041378	12.1129	0.7205	UP	Cldn5
ENSMUSG00000041439	1.6183	0.1421	UP	Mfsd6
ENSMUSG00000041459	2.6248	0.1832	UP	Tardbp
ENSMUSG00000041688	1.3476	0.2288	UP	Amot
ENSMUSG00000041926	3.0376	0.2864	UP	Rnpep
ENSMUSG00000042066	2.3877	0.1951	UP	Tmcc2
ENSMUSG00000042215	1.6174	0.3364	UP	Bag2
ENSMUSG00000042331	2.4753	0.2864	UP	Specc1
ENSMUSG00000042369	1.34	0.2175	UP	Rbm45
ENSMUSG00000042426	2.0874	0.2554	UP	Dhx29
ENSMUSG00000042670	1.7404	0.3651	UP	Immp1l
ENSMUSG00000043065	2.2212	0.2958	UP	Spice1

ENSMUSG00000043241	2.9858	0.2756	UP	Upf2
ENSMUSG00000043284	1.7279	0.3109	UP	Tmem11
ENSMUSG00000043872	5.9037	0.5717	UP	Zmym1
ENSMUSG00000044224	1.5022	0.2417	UP	Dnajc21
ENSMUSG00000044715	2.012	0.2086	UP	Gskip
ENSMUSG00000044881	2.319	0.5823	UP	Coa4
ENSMUSG00000044952	4.9918	0.5117	UP	Kctd21
ENSMUSG00000045092	3.1251	0.2173	UP	S1pr1
ENSMUSG00000045282	2.0219	0.3074	UP	Tmem86b
ENSMUSG00000045671	2.8795	0.2472	UP	Spred2
ENSMUSG00000046230	2.1143	0.2438	UP	Vps13a
ENSMUSG00000046312	1.632	0.2509	UP	Myorg
ENSMUSG00000047635	1.5676	0.3696	UP	Mtrfr
ENSMUSG00000047963	1.9375	0.6847	UP	Stbd1
ENSMUSG00000048782	1.4264	0.4603	UP	Insc
ENSMUSG00000048878	2.3602	0.2172	UP	Hexim1
ENSMUSG00000048988	1.3289	0.2694	UP	Elfn1
ENSMUSG00000049327	2.4419	0.2531	UP	Kmt5a
ENSMUSG00000049421	1.4237	0.1597	UP	Zfp260
ENSMUSG00000049511	1.3846	0.2735	UP	Htr1b
ENSMUSG00000049521	2.493	0.3193	UP	Cdc42ep1
ENSMUSG00000050017	1.4676	0.1641	UP	Pitpnb
ENSMUSG00000050244	2.1645	0.2574	UP	Heatr1
ENSMUSG00000051255	2.7347	0.3912	UP	Gm6563
ENSMUSG00000051256	2.0056	0.2798	UP	Jagn1
ENSMUSG00000051341	1.7882	0.3977	UP	Zfp52
ENSMUSG00000051515	3.4144	0.4995	UP	Fam181b
ENSMUSG00000051590	1.3734	0.3307	UP	Map3k19
ENSMUSG00000051721	2.1933	0.3939	UP	Wdcp
ENSMUSG00000052214	2.5942	0.2395	UP	Opa3
ENSMUSG00000052229	2.2915	0.3008	UP	Gpr17
ENSMUSG00000052406	2.5487	0.2508	UP	Rexo4
ENSMUSG00000052430	2.4419	0.3189	UP	Bmpr1b
ENSMUSG00000052833	1.5787	0.1763	UP	Sae1
ENSMUSG00000053004	1.3642	0.2528	UP	Hrh1
ENSMUSG00000053545	1.7566	0.6506	UP	6430503K07Rik
ENSMUSG00000055069	1.5606	0.6863	UP	Rab39
ENSMUSG00000055116	4.65	0.4469	UP	Arntl
ENSMUSG00000055334	1.3251	0.2815	UP	Snupn
ENSMUSG00000056692	1.6462	0.1647	UP	Ilrun
ENSMUSG00000056749	3.5944	0.5252	UP	Nfil3
ENSMUSG00000057101	2.2915	0.2666	UP	Zfp180
ENSMUSG00000057130	1.5478	0.2791	UP	Txnl4a
ENSMUSG00000057265	1.4858	0.3832	UP	Bbof1
ENSMUSG00000057315	1.3531	0.2176	UP	Arhgap24

ENSMUSG00000057329	2.2891	0.2394	UP	Bcl2
ENSMUSG00000057335	2.5891	0.2323	UP	Cep170
ENSMUSG00000057342	1.4705	0.2459	UP	Sphk2
ENSMUSG00000057836	1.438	0.583	UP	Xlr3a
ENSMUSG00000058006	1.9222	0.1773	UP	Mdn1
ENSMUSG00000058230	1.395	0.1766	UP	Arhgap35
ENSMUSG00000058388	3.6971	0.2832	UP	Phtf1
ENSMUSG00000059208	4.0564	0.2688	UP	Hnrnpm
ENSMUSG00000059423	3.5944	0.4334	UP	Zfp933
ENSMUSG00000059436	1.7268	0.1804	UP	Max
ENSMUSG00000059495	1.5183	0.1573	UP	Arhgef12
ENSMUSG00000059851	1.3251	0.2472	UP	Kmt5c
ENSMUSG00000060216	1.4783	0.1662	UP	Arrb2
ENSMUSG00000060261	1.7862	0.1591	UP	Gtf2i
ENSMUSG00000060373	5.9079	0.2937	UP	Hnrnpc
ENSMUSG00000060771	2.93	0.429	UP	Tsga10
ENSMUSG00000060862	4.3521	0.5677	UP	Zbtb40
ENSMUSG00000061353	1.3924	0.2915	UP	Cxcl12
ENSMUSG00000061718	1.8528	0.2496	UP	Ppp1r1b
ENSMUSG00000062393	3.4298	0.2894	UP	Dgkk
ENSMUSG00000062563	1.6971	0.3399	UP	Cys1
ENSMUSG00000062960	3.3562	0.4583	UP	Kdr
ENSMUSG00000063087	1.3846	0.3088	UP	Gm10125
ENSMUSG00000063239	1.3411	0.2468	UP	Grm4
ENSMUSG00000063317	4.9122	0.3743	UP	Usp31
ENSMUSG00000063535	2.4067	0.5539	UP	Zfp773
ENSMUSG00000063889	1.7552	0.3438	UP	Crem
ENSMUSG00000064127	1.3251	0.1634	UP	Med14
ENSMUSG00000064337	4.977	0.387	UP	mt-Rnr1
ENSMUSG00000064339	6.3716	0.364	UP	mt-Rnr2
ENSMUSG00000064343	1.8695	1.0399	UP	mt-Tq
ENSMUSG00000064350	1.304	0.3387	UP	mt-Ty
ENSMUSG00000066026	4.9704	0.447	UP	Dhrs3
ENSMUSG00000068184	1.3595	0.3127	UP	Ndufaf2
ENSMUSG00000068566	1.9673	0.2003	UP	Myadm
ENSMUSG00000069763	2.0667	0.3549	UP	Tmem100
ENSMUSG00000069806	2.6071	0.2205	UP	Cacng7
ENSMUSG00000070366	1.4107	0.3126	UP	Plpp4
ENSMUSG00000070461	1.4887	0.3829	UP	9230112E08Rik
ENSMUSG00000070532	1.3608	0.3057	UP	Ccdc190
ENSMUSG00000070544	3.3562	0.3037	UP	Top1
ENSMUSG00000070570	1.4783	1.1927	UP	Slc17a7
ENSMUSG00000070730	1.4547	0.1888	UP	Rmdn3
ENSMUSG00000071072	3.6383	0.2873	UP	Ptges3
ENSMUSG00000071753	2.4617	0.2651	UP	Cdr1os

ENSMUSG00000072572	2.4419	0.415	UP	Slc39a2
ENSMUSG00000072770	2.649	0.4231	UP	Acrbp
ENSMUSG00000072919	2.4599	1.1747	UP	Noxred1
ENSMUSG00000073676	1.7151	0.2102	UP	Hspe1
ENSMUSG00000074457	1.3299	0.2246	UP	S100a16
ENSMUSG00000074733	2.0143	0.2032	UP	Zfp950
ENSMUSG00000075232	2.0254	0.1732	UP	Amd1
ENSMUSG00000078684	3.5578	0.4971	UP	5830417I10Rik
ENSMUSG00000078903	3.0917	1.9068	UP	Gm14391
ENSMUSG00000078941	2.5339	0.5469	UP	Ak6
ENSMUSG00000079104	1.4705	0.3263	UP	Prps1l3
ENSMUSG00000079442	1.8971	0.2896	UP	St6galnac4
ENSMUSG00000079470	2.6105	0.3727	UP	Utp14b
ENSMUSG00000083396	1.5121	0.3671	UP	Gm15542
ENSMUSG00000083889	3.1608	0.5552	UP	E530001F21Rik
ENSMUSG00000084904	2.427	0.3215	UP	Gm14827
ENSMUSG00000085028	1.3606	0.2559	UP	Slc2a4rg-ps
ENSMUSG00000085069	2.8645	0.45	UP	Prdm16os
ENSMUSG00000085272	2.2501	0.6173	UP	Sbk3
ENSMUSG00000087260	2.1315	0.25	UP	Lamtor5
ENSMUSG00000087368	1.8695	0.6263	UP	BC065397
ENSMUSG00000089774	4.3313	0.4384	UP	Slc5a3
ENSMUSG00000089782	1.5227	0.6832	UP	Btf3-ps1
ENSMUSG00000089832	1.4339	0.2375	UP	Shkbp1
ENSMUSG00000090210	2.389	0.7179	UP	Itga10
ENSMUSG00000090266	1.3129	0.3942	UP	Mettl23
ENSMUSG00000090659	1.4731	0.588	UP	Zfp493
ENSMUSG00000090935	5.7773	0.3838	UP	Synj2bp
ENSMUSG00000091474	1.9222	0.2794	UP	2610021A01Rik
ENSMUSG00000092035	2.5523	0.3476	UP	Peg10
ENSMUSG00000092558	3.4522	0.322	UP	Med20
ENSMUSG00000093351	1.3146	0.7236	UP	Mir3072
ENSMUSG00000094103	2.0522	3.6882	UP	Fam177a2
ENSMUSG00000094392	1.7871	0.7397	UP	Gm3788
ENSMUSG00000097042	1.7279	0.4833	UP	Gm17491
ENSMUSG00000097391	2.2794	0.2309	UP	Mirg
ENSMUSG00000097649	1.6691	0.4559	UP	Gm10561
ENSMUSG00000100801	1.9695	0.2613	UP	Gm15459
ENSMUSG00000101840	1.4715	1.0611	UP	Gm28294
ENSMUSG00000105265	1.522	0.3234	UP	Sox2ot
ENSMUSG00000109179	2.1787	0.4179	UP	Gm35339
ENSMUSG00000110218	1.3294	0.514	UP	Tincr
ENSMUSG00000113029	2.1143	0.4291	UP	Gm40578
ENSMUSG00000113186	2.7246	0.5484	UP	A330076C08Rik
ENSMUSG00000114995	2.5666	0.4112	UP	Gm49284

ENSMUSG00000115813	2.0683	0.7052	UP	Gm46447
ENSMUSG00000116995	1.9375	0.2712	UP	Gm21926
ENSMUSG00000117465	4.1575	1.0319	UP	Gm49980
ENSMUSG00000117621	1.4022	0.6769	UP	Hspe1-rs1
ENSMUSG00000118219	3.0376	0.5045	UP	Gm29695
ENSMUSG00000120194	6.7536	1.2465	UP	ENSMUSG00000120194
ENSMUSG00000120241	6.7235	0.5705	UP	ENSMUSG00000120241
ENSMUSG00000120401	1.3987	0.8495	UP	ENSMUSG00000120401
ENSMUSG00000120908	1.4732	0.5379	UP	ENSMUSG00000120908
**sham: TRF_A_VS_ALF_A**				
ENSMUSG00000018727	1.4229	-0.7109	DOWN	Cpsf4l
ENSMUSG00000028820	3.4649	-0.3612	DOWN	Sfpq
ENSMUSG00000117465	2.2148	-1.096	DOWN	Gm49980
ENSMUSG00000005705	2.2148	0.7201	UP	Agrp
ENSMUSG00000078887	3.2052	1.6157	UP	Gm6710
ENSMUSG00000095478	3.4649	1.1643	UP	Gm9824

**Supplementary Table 3: Differentially expressed genes related to circadian or mitochondrial function**					
TRF: SNI_VS_sham		ALF: SNI_VS_sham		sham: TRF_M_VS_TRF_A	
Circadian-related	Mitochondria-related	Circadian-related	Mitochondria-related	Circadian-related	Mitochondria-related
ndufa9	cox5a	ndufa9	cox5a	cry1	spg7
kmt2a	dbt	nr2f6	dbt	srebf1	ddx3x
nr2f6	ndufa9	hdac3	ndufa9	rock2	fkbp10
adora2a	atp5pb	mycbp2	bax	nr1d1	clpb
id4	top1mt	becn1	etfb	per1	ndufb2
dyrk1a	oxa1l	pmch	tomm40l	nr1d2	timm44
hdac3	dhrs1	thrap3	ubc	mettl3	ler3
kat5	ndufa11	jund	rnf5	creb1	mtfp1
fxr1	clpp		cox11	fxr1	dynll1
rorc	alkbh7		nme2	sfpq	plcd1
prkg2	atg4d		slirp	per3	mrps25
atg7	qtrt1		mrpl13	usp2	serac1
nlgn3	mrpl4		tymp	hcrtr2	rmnd1
mycbp2	slc25a1		mpc1	ciart	psen1
becn1	coq8b		grpel2	kcna2	mpg
atf5	bax		bad	tardbp	hspa4
fhx3	fam162a		ndufb8	per2	eif2s1
agrn	etfb		cyp2e1	nfil3	hsp90aa1
atf4	tomm40l		dtymk	dbp	ap3b1
thrap3	mrpl49		cox20	nlgn1	golph3
ahcyl	bcl2l1		rab5if	crem	atg3
nrip1	ubc		cyp2u1	cry2	tfrc
nlgn1	vdac3		cibar1	top1	cox14
jund	dynll1		diablo	jund	mrpl14

	mcu		pdha1		lipa
	sdhb		mt3		pycr1
	mrpl52		bco2		slc25a10
	ndufs2		cck		creb1
	slc25a11		mrpl3		hspd1
	rnf5		tomm6		bcs1l
	serac1		rbfox2		coq8a
	surf1		gadd45gip1		mgme1
	ndufa1		acot11		ndufaf4
	mtif3		becn1		ak4
	mrps18c		bri3bp		agk
	cox7c		mrps34		tspan9
	cyb5r3		slc25a34		mt3
	mrpl45		cox6a1		rexo2
	tmem160		chchd10		exd2
	calm3		ywhag		armcx2
	ubb		mllt11		vps13c
	mrpl24		dnajc30		tk2
	wasf1		ptpmt1		aifm1
	ndufs7		gpx1		letm2
	uqcr11		mt-atp8		tigar
	adora2a		csde1		atpsckmt
	mpg		ndufb6		mrps6
	acsl6		ak157302		mthfd1l
	canx		bnip3		immp1l
	pnpt1		bcl2a1b		tmem11
	mrps24		acad11		coa4
	cox11		cox16		vps13a
	adap2		dynlt1a		nsun3
	nme2		pigbos1		rab11fip5
	gstz1		hspe1-rs1		bcl2
	slirp				sphk2
	glrx5				fdps
	isca2				arrb2
	ccdc127				stx17
	pdhb				kdr
	vdac2				mt-rnr1
	mrpl57				mt-rnr2
	ndufb9				ndufaf2
	mrpl13				rmdn3
	mief1				slc25a16
	ndufa6				hspe1
	smdt1				synj2bp
	cyc1				hspe1-rs1
	yars2				

	mrpl39				
	ndufa5				
	mpc1				
	slc25a27				
	ndufv3				
	ndufv2				
	eci1				
	hagh				
	cox7a2l				
	mrps18b				
	grpel2				
	timm21				
	sdhaf2				
	mrpl21				
	mrpl11				
	bad				
	ndufb8				
	twnk				
	hsd17b10				
	atp23				
	cyp2e1				
	sirt3				
	iscu				
	mrpl30				
	mff				
	capn10				
	dtymk				
	cox20				
	miga2				
	pmpca				
	mapk8ip1				
	mrps5				
	ndufaf5				
	nfs1				
	rab5if				
	acad9				
	gfm1				
	etfdh				
	cyp2u1				
	ppa2				
	cibar1				
	stoml2				
	hint2				
	park7				
	slc25a33				

	grpel1				
	nipsnap2				
	triap1				
	acads				
	gstk1				
	atg7				
	mrpl51				
	mrps11				
	ccdc90b				
	tmem126b				
	ndufc2				
	coq7				
	ndufab1				
	ndufb11				
	pdha1				
	mrps31				
	slc25a4				
	got2				
	mt3				
	arl2bp				
	rexo2				
	bckdhb				
	nme6				
	mrpl3				
	pebp1				
	rbfox2				
	sfxxn5				
	gadd45gip1				
	mrps10				
	acot11				
	mrpl34				
	mrpl54				
	vat1				
	becn1				
	lars2				
	slc25a21				
	ndufa3				
	mrps2				
	mrpl41				
	mrpl23				
	ppm1k				
	bri3bp				
	mrm3				
	mrps34				
	foxred1				

	mrps6				
	bmf				
	slc25a28				
	pcca				
	cox6a1				
	ndufa7				
	atf4				
	adprs				
	immp1l				
	bola3				
	cox19				
	mrps12				
	lyrm4				
	nat8l				
	timm29				
	ggnbp1				
	chchd10				
	micos13				
	selenon				
	ywhag				
	mllt11				
	nrgn				
	slc25a40				
	mrps21				
	nat8f1				
	antkmt				
	bak1				
	bckdha				
	dnajc30				
	cox5b				
	cebpzos				
	mrpl42				
	ptpmt1				
	sfxn4				
	gpx1				
	uqcrh				
	ethe1				
	mt-rnr1				
	mt-nd1				
	mt-nd2				
	mt-co2				
	mt-atp8				
	mt-atp6				
	mt-co3				
	mt-nd4				

	mt-nd6				
	mt-nd4l				
	romo1				
	htra2				
	csde1				
	ndufaf3				
	ndufb6				
	mpst				
	ptrh2				
	hspe1				
	sdhaf1				
	smim26				
	gpx4				
	ak157302				
	bnip3				
	ndufaf8				
	apoo				
	bcl2l2				
	bcl2a1b				
	bloc1s1				
	cox16				
	pigbos1				
	pnp				
	tmem223				

**Supplementary Table 4: A list of differentially variable genes (DVG) in different comparisons**				
**Gene**	**LogVarRatio**	***p*-value**	**Adj *p*-value**	**Expression**
**TRF: SNI_VS_sham**				
Dgat2	-2.7204	0	0.0631	down
Nrxn3	-2.2309	1.00E-04	0.3066	Down
Slco1a4	-1.5983	1.00E-04	0.3066	Down
Slc2a8	-2.589	2.00E-04	0.3066	down
Ptcd3	-2.9401	2.00E-04	0.3066	down
Dhrs3	-2.1486	2.00E-04	0.3066	down
Mpdz	-1.9611	2.00E-04	0.3066	down
Prrt4	-2.0894	2.00E-04	0.3066	down
Mettl3	-2.1442	3.00E-04	0.3066	down
Nkx2-1	-2.6784	3.00E-04	0.3066	down
Heyl	-1.6679	3.00E-04	0.3066	down
Drd2	-1.9189	4.00E-04	0.3066	down
Gdi1	-2.0021	4.00E-04	0.3066	down
Apcdd1	-1.9391	5.00E-04	0.3066	down
Dnajc10	-1.7213	5.00E-04	0.3066	down
Rbm28	-1.4235	5.00E-04	0.3066	down
Col11a2	-1.7413	5.00E-04	0.3066	down
2410002F23Rik	-2.4214	5.00E-04	0.3066	down
Kpna4	-2.7821	5.00E-04	0.3066	down
Uchl1	-1.8992	6.00E-04	0.3066	Down

Pacsin2	-2.2719	6.00E-04	0.3066	Down
H2-Q2	-3.6146	6.00E-04	0.3066	Down
Gm9824	-2.8931	6.00E-04	0.3066	Down
Mblac2	-1.5267	6.00E-04	0.3066	Down
Asb4	-3.6388	7.00E-04	0.3066	Down
Trub1	-1.8199	7.00E-04	0.3066	Down
Btf3l4	-2.5876	7.00E-04	0.3066	Down
Rprm	-2.2865	7.00E-04	0.3066	Down
Hnrnpa0	-1.7252	7.00E-04	0.3066	Down
Rbm11	-1.3341	8.00E-04	0.3066	Down
Thbs4	-3.3067	8.00E-04	0.3066	Down
Ddc	-1.5852	8.00E-04	0.3066	Down
Uba6	-1.4724	9.00E-04	0.3241	Down
Calr	-1.5669	0.001	0.3253	Down
Rgs14	-2.478	0.0011	0.3253	Down
Epm2a	-1.8409	0.0011	0.3253	Down
Snrpe	-2.1967	0.0011	0.3253	Down
Mtrex	-1.7173	0.0012	0.3253	Down
Cplx1	-2.8249	0.0012	0.3253	Down
Xdh	-2.019	0.0012	0.3253	Down
Mzt2	-2.5492	0.0012	0.3253	Down
Ndnf	-1.9623	0.0012	0.3253	Down
Cpt1c	-2.3532	0.0012	0.3253	Down
Mthfd1l	-2.0491	0.0012	0.3253	Down
Rxrg	-1.9171	0.0013	0.334	Down
Rtn4rl1	-1.8699	0.0013	0.3364	Down
Tmcc1	-2.172	0.0013	0.3364	Down
Odad1	-2.5004	0.0013	0.3364	Down
Usp31	-1.5924	0.0015	0.3637	Down
Sbk3	-2.4635	0.0015	0.3649	Down
Plekha7	-1.9142	0.0016	0.3728	Down
Lncenc1	-2.354	0.0016	0.3728	Down
Cyp4f15	-1.8292	0.0017	0.3728	Down
Zfp777	-2.1404	0.0017	0.3728	Down
Ell2	-3.7012	0.0017	0.3728	Down
Rab34	-1.6272	0.0017	0.3728	Down
Gspt1	-1.6161	0.0017	0.3734	Down
Sfrp4	-2.9413	0.0019	0.3928	Down
Creld2	-1.3094	0.0019	0.3933	Down
Anxa6	-1.5904	0.0019	0.3941	Down
Mbtps2	-3.0845	0.002	0.3941	Down
Stip1	-1.4955	0.0021	0.3941	Down
Araf	-2.4793	0.0021	0.3941	Down
Arx	-1.8424	0.0021	0.3941	Down
Hmgb3	-1.4608	0.0021	0.3941	Down
Nrarp	-1.6459	0.0021	0.3941	Down
Srebf1	-1.7926	0.0021	0.3941	Down
Slc12a2	-2.6624	0.0022	0.3957	Down
Foxd2	-1.7014	0.0022	0.3957	Down
Pla2g4e	-1.4514	0.0022	0.3957	Down
Shtn1	-1.8771	0.0023	0.3957	Down
Gm43305	-2.6417	0.0023	0.396	Down
Septin8	-2.2217	0.0024	0.396	Down
Arhgef10l	-1.6874	0.0025	0.4052	Down
Braf	-2.5467	0.0025	0.4054	Down
Elovl7	-2.8837	0.0026	0.4085	Down

Gpr50	-2.2866	0.0027	0.4085	Down
Dact3	-2.1743	0.0027	0.4085	Down
Pls3	-2.4202	0.0028	0.4085	Down
Nrip3	-2.712	0.0028	0.4085	Down
Idh3g	-1.6952	0.0029	0.4207	Down
Sytl4	-1.9206	0.003	0.427	Down
Fuca1	-1.5604	0.0032	0.435	Down
Otud1	-1.8021	0.0032	0.435	Down
Synj2bp	-1.2993	0.0032	0.435	Down
Bag6	-1.4634	0.0033	0.435	Down
Cttnbp2nl	-2.2587	0.0034	0.435	Down
Ubash3b	-2.8851	0.0034	0.435	Down
Ywhae	-1.8061	0.0034	0.435	Down
Nfkbia	-1.6376	0.0035	0.435	Down
Cpd	-2.3726	0.0035	0.435	Down
Vwa5a	-1.8796	0.0036	0.435	Down
Trim9	-0.7888	0.0036	0.435	Down
Mettl16	-1.0155	0.0036	0.435	Down
Sfrp2	-3.1794	0.0037	0.435	Down
Stim1	-2.0051	0.0038	0.435	Down
Impact	-1.7791	0.0038	0.435	Down
Nrsn2	-1.3471	0.0039	0.435	Down
Ccdc50	-2.6237	0.004	0.435	Down
Rbm24	-1.8679	0.004	0.435	Down
Gstcd	-2.7026	0.004	0.435	Down
Atic	-2.5481	0.004	0.435	Down
Ifi27	-1.4144	0.004	0.435	Down
Cerk	-3.2571	0.004	0.435	Down
Wdr37	-2.1157	0.0041	0.435	Down
Ube2m	-1.7142	0.0041	0.435	Down
Slc2a9	-2.7644	0.0041	0.435	Down
Purb	-1.6843	0.0041	0.435	Down
Fam76a	-1.2557	0.0042	0.435	Down
Scn9a	-1.9725	0.0043	0.435	Down
Fabp7	-1.3675	0.0043	0.435	Down
Snrnp48	-2.3221	0.0043	0.435	Down
Amigo2	-2.1539	0.0044	0.435	Down
Pced1b	-2.1132	0.0044	0.435	Down
Clic6	-2.0186	0.0044	0.435	Down
Itgb8	-1.7873	0.0044	0.435	Down
Gpm6b	-2.5001	0.0045	0.435	Down
Foxred2	-1.7527	0.0045	0.435	Down
Doc2b	-1.9866	0.0046	0.435	Down
Dnajc27	-1.781	0.0046	0.435	Down
Mettl23	-1.6444	0.0046	0.435	Down
Aspscr1	-1.4385	0.0046	0.435	Down
Adamts20	-1.7698	0.0047	0.435	Down
Pou3f1	-1.8308	0.0047	0.435	Down
Foxb1	-1.7659	0.0048	0.435	Down
Pak6	-2.0224	0.0048	0.435	Down
Slc22a21	-2.5883	0.0048	0.435	Down
Stac3	-2.3594	0.0048	0.435	Down
Hif1a	-1.782	0.0048	0.435	Down
Gm2415	-2.5801	0.0048	0.435	Down
Zfp956	-2.1192	0.0048	0.435	Down
Tspan9	-2.0252	0.0049	0.435	Down

Ptprz1	-2.3842	0.0049	0.435	Down
Miip	-1.8145	0.0049	0.435	Down
Nln	-2.1881	0.0049	0.435	Down
Bzw1	-1.6309	0.0049	0.435	Down
Carhsp1	-2.1662	0.0049	0.435	Down
Dnaja1	-1.5903	0.0051	0.4476	Down
Senp6	-1.8692	0.0052	0.4517	Down
Npas4	-1.1942	0.0053	0.4522	Down
B4galnt4	-1.5375	0.0053	0.4522	Down
Sdhaf3	-1.8339	0.0054	0.4539	Down
Tmem30a	-2.0491	0.0054	0.4539	Down
Sox12	-1.5839	0.0055	0.4539	Down
Myh9	-1.4856	0.0055	0.4539	Down
Hhip	-2.2455	0.0056	0.4552	Down
Esrrg	-2.4108	0.0057	0.4552	Down
H1f10	-1.6513	0.0057	0.4552	Down
Dnajc3	-2.0148	0.0057	0.4552	Down
Gabre	-2.1431	0.0058	0.4552	Down
Xylt2	-1.443	0.0058	0.4552	Down
Hpcal4	-2.2049	0.0059	0.4552	Down
Nsun3	-1.565	0.0061	0.4552	Down
Pcdhb7	-1.8221	0.0061	0.4552	Down
Tshr	-2.1083	0.0061	0.4552	Down
Pias3	-1.5418	0.0061	0.4552	Down
Gm10275	-1.5846	0.0062	0.4552	Down
Per3	-1.0501	0.0062	0.4552	Down
Cntnap2	-1.1857	0.0062	0.4552	Down
Dnai4	-1.6918	0.0063	0.4552	Down
Lmna	-1.9386	0.0063	0.4552	Down
Efnb1	-1.735	0.0063	0.4552	Down
4933406B17Rik	-1.9496	0.0063	0.4552	Down
Prkar1a	-2.3773	0.0063	0.4552	Down
Ttc21a	-2.31	0.0064	0.4552	Down
Cbln1	-1.7732	0.0065	0.4552	Down
Cry1	-2.0909	0.0065	0.4552	Down
Kcna5	-1.6131	0.0066	0.4552	Down
Napb	-1.355	0.0066	0.4552	Down
Cep131	-1.1495	0.0066	0.4552	Down
Prpf4b	-1.5617	0.0067	0.4552	Down
Fxr1	-1.6	0.0067	0.4552	Down
Armcx2	-1.6641	0.0067	0.4552	Down
Map4	-2.6483	0.0067	0.4552	Down
Susd2	-2.5318	0.0067	0.4552	Down
Tmem158	-2.4167	0.0067	0.4552	Down
Brinp3	-3.2727	0.0067	0.4552	Down
Drosha	-1.5324	0.0067	0.4552	Down
Stk32a	-2.5143	0.0068	0.4552	Down
Ubl7	-1.9519	0.0068	0.4552	Down
Sp3os	-1.9211	0.0069	0.4552	Down
Tbc1d24	-2.0145	0.0069	0.4552	Down
Rdx	-1.8223	0.0069	0.4552	Down
Srrt	-1.3041	0.007	0.4552	Down
Clgn	-1.958	0.007	0.4552	Down
Hba-a1	-1.8122	0.007	0.4552	Down
Sfpq	-0.9519	0.007	0.4552	Down
Aplp2	-1.7842	0.007	0.4552	Down

Gm10767	-2.1364	0.0071	0.4611	Down
Acsl3	-1.8099	0.0071	0.4616	Down
Myh7b	-2.4371	0.0072	0.4654	Down
Snx21	-1.6582	0.0073	0.469	Down
Spcs2	-1.7502	0.0074	0.469	Down
Atp2c1	-1.921	0.0074	0.469	Down
Grik5	-2.3982	0.0075	0.4692	Down
Tacr3	-2.1206	0.0075	0.4692	Down
Klf15	-2.3889	0.0075	0.4692	Down
Dbp	-0.7576	0.0076	0.4692	Down
Tnni1	-1.8911	0.0076	0.4692	Down
Tle2	-2.1961	0.0077	0.4692	Down
Kcnj16	-1.5148	0.0077	0.4692	Down
Bckdk	-2.5546	0.0077	0.4692	Down
Ppp1r12c	-2.1099	0.0078	0.4692	Down
Ecel1	-2.829	0.0078	0.47	Down
Mospd3	-2.1118	0.0079	0.47	Down
BC065397	-2.0259	0.0079	0.47	Down
Plvap	-1.9572	0.008	0.47	Down
Traf7	-1.9835	0.008	0.47	Down
Emp2	-1.6517	0.0081	0.47	Down
Cpne9	-2.0396	0.0081	0.47	Down
Yap1	-1.8612	0.0081	0.47	Down
Cacng7	-1.648	0.0082	0.47	Down
Sms	-2.8442	0.0083	0.47	Down
Ndrg1	-1.4566	0.0083	0.47	Down
Mrpl55	-2.3655	0.0083	0.47	Down
Mid1	-2.2384	0.0084	0.47	Down
Cyth1	-2.2136	0.0084	0.47	Down
Lpgat1	-2.2125	0.0084	0.47	Down
Rps27rt	-1.5996	0.0084	0.47	Down
Sulf1	-2.4861	0.0085	0.47	Down
Ddr1	-2.2334	0.0085	0.47	Down
Spred1	-1.3522	0.0086	0.47	Down
Rbm45	-2.0097	0.0086	0.47	Down
Baiap3	-1.326	0.0087	0.4711	Down
Pou3f2	-2.6581	0.0088	0.4711	Down
Eps15	-2.5851	0.0088	0.4711	Down
Arih2	-1.6029	0.0088	0.4711	Down
Higd2a	-1.8955	0.0088	0.4711	Down
Garnl3	-1.5084	0.0089	0.4715	Down
Etnk2	-2.2463	0.0089	0.4715	Down
Ninl	-2.6181	0.0092	0.4823	Down
Irf3	-1.3417	0.0092	0.4847	Down
Fam20a	-1.8011	0.0093	0.4847	Down
Tle3	-1.3089	0.0093	0.4847	Down
Zic1	-1.4371	0.0094	0.4847	Down
Pkia	-1.7118	0.0094	0.4847	Down
Tspyl2	-2.3107	0.0094	0.4847	Down
Map7d1	-2.1749	0.0095	0.4874	Down
Rnf112	-1.9493	0.0096	0.4874	Down
Pdlim2	-2.5349	0.0096	0.4874	Down
Zfp109	-1.1145	0.0096	0.4874	Down
Fah	-2.1111	0.0096	0.4875	Down
Slc6a8	-1.4265	0.0098	0.4914	Down
Ddx3y	-1.81	0.0098	0.4915	Down

Mkln1	-1.9607	0.0099	0.4915	Down
Chchd3	-1.7855	0.0099	0.4915	Down
Rhot1	-1.636	0.01	0.4915	Down
Rps12-ps3	2.1779	0	0.0631	Up
Gm13394	3.2291	1.00E-04	0.3066	Up
Gm28439	2.7042	3.00E-04	0.3066	Up
Gm3756	2.1643	7.00E-04	0.3066	Up
Stx6	3.2837	8.00E-04	0.3066	Up
Rps29	2.2534	8.00E-04	0.3066	Up
Gm6565	2.0107	8.00E-04	0.3066	Up
Gm7336	2.6018	8.00E-04	0.3066	Up
Slc6a7	1.7484	0.0011	0.3253	Up
Gm48678	2.245	0.0012	0.3253	Up
Gm3362	1.7191	0.0012	0.3253	Up
Ubb-ps	2.4677	0.0014	0.3561	Up
Rpl30	1.5376	0.0019	0.3941	Up
Pisd-ps2	3.2103	0.0021	0.3941	Up
Dgcr2	2.5333	0.0023	0.3957	Up
Pin4	2.3453	0.0024	0.396	Up
Gm5577	2.8433	0.0025	0.4052	Up
Gabbr1	1.6803	0.0026	0.4085	Up
Gm12070	2.0607	0.0027	0.4085	Up
Gm10925	2.5847	0.0027	0.4085	Up
Gm15387	2.3192	0.0028	0.4085	Up
Gxylt1	2.866	0.003	0.4311	Up
Gm10222	1.4764	0.0033	0.435	Up
Lrsam1	2.1966	0.0034	0.435	Up
Gm5641	1.9554	0.0039	0.435	Up
Mef2a	1.9125	0.0039	0.435	Up
Gm14326	1.8565	0.0044	0.435	Up
Rpl7a-ps5	1.989	0.0044	0.435	Up
Rpl15-ps3	2.5799	0.0046	0.435	Up
Gm8325	1.8745	0.0047	0.435	Up
Tug1	1.8904	0.0048	0.435	Up
Gm15459	1.3007	0.0051	0.4476	Up
Itga5	2.7397	0.0054	0.4539	Up
Trim30a	2.2615	0.0054	0.4539	Up
Lhx1	2.629	0.0055	0.4539	Up
Rpl9-ps6	1.5749	0.006	0.4552	Up
Cox16	2.1574	0.0061	0.4552	Up
Oaz1-ps	1.6048	0.0061	0.4552	Up
Bax	1.0797	0.0064	0.4552	Up
Ints5	3.3623	0.0068	0.4552	Up
Gm15710	1.3547	0.0076	0.4692	Up
Med22	2.2225	0.0077	0.4692	Up
ENSMUSG00000121115	2.9291	0.0078	0.4692	Up
Herpud2	1.7583	0.0079	0.47	Up
Zan	2.697	0.0083	0.47	Up
Bub1b	2.004	0.0084	0.47	Up
Serf2	1.3088	0.0085	0.47	Up
Ing1	1.3739	0.0085	0.47	Up
Gm44677	1.6638	0.0088	0.4711	Up
Polr2k	1.4551	0.0088	0.4711	Up
Gsg1l	2.6865	0.0088	0.4711	Up
Gm42715	1.703	0.0095	0.4874	Up
Hjurp	1.3862	0.0098	0.4914	Up

ALF: SNI_VS_sham				
Mgat5	-2.1799	4.00E-04	0.4376	Down
Slc20a2	-3.1572	4.00E-04	0.4376	Down
Gab2	-2.5593	4.00E-04	0.4376	Down
Trp53i11	-3.0899	4.00E-04	0.4376	Down
Ado	-2.9875	7.00E-04	0.4376	Down
Sars	-3.1974	0.0014	0.4376	Down
Uqcrfs1	-2.8357	0.0017	0.4376	Down
Asb4	-3.3949	0.0019	0.4376	Down
Irs4	-2.4849	0.0023	0.4376	Down
ENSMUSG00000095041	-2.4272	0.0026	0.4376	Down
Tmeff2	-3.2683	0.0026	0.4376	Down
Cacna1c	-3.171	0.0027	0.4376	Down
Sncb	-2.3722	0.0028	0.4376	Down
Pdhb	-4.231	0.0031	0.4376	Down
Foxb1	-3.2236	0.0032	0.4376	Down
Atp6v1d	-3.6168	0.0033	0.4376	Down
Slc6a3	-2.0032	0.0036	0.4376	Down
Hcrt	-2.3257	0.0037	0.4376	Down
Cmas	-3.1969	0.0037	0.4376	Down
Tenm2	-3.8095	0.0038	0.4376	Down
Grhpr	-2.4803	0.0038	0.4376	Down
Rps6ka2	-2.8046	0.0039	0.4376	Down
Wdr35	-1.9455	0.0041	0.4376	Down
Edc4	-2.531	0.0046	0.4376	Down
Tom1l2	-1.5175	0.0053	0.4376	Down
Nid2	-3.0009	0.0056	0.4376	Down
Atp6v1c1	-3.3903	0.0056	0.4376	Down
Aebp1	-1.9403	0.0056	0.4376	Down
Fzd5	-3.6841	0.0057	0.4376	Down
Rwdd2a	-3.5315	0.0058	0.4376	Down
Fdft1	-1.297	0.0063	0.4376	Down
Mog	-2.0045	0.0063	0.4376	Down
Iqsec1	-3.0217	0.0063	0.4376	Down
Sdk1	-4.0099	0.0064	0.4376	Down
Bmp7	-2.5073	0.0068	0.4376	Down
Hspg2	-3.1635	0.0068	0.4376	Down
Sec31a	-2.9948	0.0069	0.4376	Down
Fstl4	-3.6022	0.0072	0.4376	Down
Cdh4	-2.4196	0.0077	0.4376	Down
Akap13	-2.956	0.0079	0.4376	Down
Sox11	-2.4994	0.008	0.4376	Down
Agap2	-2.8754	0.008	0.4376	Down
Zfp142	-3.0377	0.0082	0.4376	Down
Arpp21	-2.0919	0.0083	0.4376	Down
Supt6	-2.7128	0.0085	0.4376	Down
Nrp2	-2.3263	0.0087	0.4376	Down
Sar1b	-2.733	0.0088	0.4376	Down
Pcdhb7	-1.9753	0.0089	0.4376	Down
Rbm4b	-2.3913	0.009	0.4376	Down
Tceal9	-2.7419	0.0094	0.4376	Down
Ptgis	-2.4714	0.0098	0.4376	Down
Klf7	-2.1176	0.0099	0.4376	Down
Trib1	3.3017	0.0023	0.4376	Up
Tmx3	1.9323	0.004	0.4376	Up
Zfp617	2.49	0.0064	0.4376	Up

1700029I15Rik	2.9943	0.008	0.4376	Up
Ppp2r1b	2.8035	0.0085	0.4376	Up
Cbx8	1.9229	0.0091	0.4376	Up
**SNI: TRF_M_VS_A**				
Krcc1	-2.881	0.0003	0.3092	Down
Stx6	-1.9691	0.0003	0.3092	Down
Gm11847	-2.9686	0.0005	0.3122	Down
P2ry12	-8.0122	0.0006	0.3122	Down
Aars	-3.6132	0.0007	0.3302	Down
Dnah6	-3.0518	0.0012	0.3374	Down
Armcx6	-3.8679	0.0016	0.3904	Down
Bag6	-3.3884	0.0016	0.3904	Down
Vegfc	-2.9037	0.0016	0.3904	Down
Rxra	-3.1997	0.0018	0.3982	Down
Mns1	-2.658	0.0018	0.3982	Down
4921507P07Rik	-3.7793	0.0018	0.3982	Down
Agpat5	-3.1777	0.0019	0.3982	Down
Gm49284	-2.9025	0.0019	0.3982	Down
Ntrk3	-3.414	0.0021	0.4057	Down
Lrrc58	-3.0451	0.0021	0.406	Down
Gpr137c	-3.4635	0.0022	0.406	Down
Slc3a2	-5.5548	0.0023	0.406	Down
Nacad	-2.3719	0.0026	0.406	Down
Bst2	-3.316	0.0027	0.406	Down
Snhg14	-3.7104	0.0027	0.406	Down
Ess2	-6.5214	0.0027	0.406	Down
Ppp2r3c	-3.5931	0.0027	0.406	Down
Gm42047	-4.5045	0.0029	0.406	Down
Prmt6	-4.1257	0.0031	0.406	Down
Zfhx4	-3.3855	0.0032	0.406	Down
H1f0	-2.9792	0.0032	0.406	Down
Cenpl	-3.9804	0.0033	0.406	Down
Cers6	-1.8786	0.0033	0.406	Down
Ubqln1	-3.4603	0.0033	0.406	Down
Tmem18	-3.3025	0.0034	0.4062	Down
Catsperg2	-4.064	0.0036	0.4078	Down
Spsb3	-2.585	0.0038	0.4078	Down
Taf1b	-2.4404	0.0038	0.4078	Down
Aqp9	-2.9914	0.0039	0.4078	Down
Mipol1	-1.8766	0.0041	0.4078	Down
Sgsm3	-2.8879	0.0043	0.4078	Down
Gm14681	-2.2098	0.0045	0.4078	Down
Tiparp	-2.7981	0.0045	0.4078	Down
Rdx	-3.3435	0.0045	0.4078	Down
Pank2	-3.3164	0.0046	0.4078	Down
Slc15a2	-1.4581	0.0048	0.4078	Down
Zranb1	-3.434	0.0052	0.4078	Down
Rngtt	-3.8094	0.0052	0.4078	Down
Zfp788	-4.8961	0.0053	0.4078	Down
Btd	-4.6555	0.0055	0.4078	Down
Ano7	-2.4888	0.0058	0.4078	Down
Eif4e2	-3.0783	0.0058	0.4078	Down
Gbp3	-4.69	0.0058	0.4078	Down
Erlec1	-1.8168	0.0059	0.4078	Down
Acin1	-4.0531	0.0062	0.4078	Down
Sos1	-2.5879	0.0062	0.4078	Down

Slc25a17	-3.1232	0.0062	0.4078	Down
Ppcs	-4.1874	0.0063	0.4078	Down
Gpr153	-3.9166	0.0065	0.4078	Down
Bri3bp	-2.9954	0.0065	0.4078	Down
Elavl2	-3.0658	0.0065	0.4078	Down
Zkscan6	-2.2496	0.0068	0.4078	Down
Ralgapa1	-3.1436	0.0068	0.4078	Down
Pomt1	-5.0438	0.0068	0.4078	Down
Arhgef19	-1.8487	0.0072	0.4078	Down
Yif1a	-3.8809	0.0072	0.4078	Down
Zfp597	-3.9394	0.0072	0.4078	Down
Psmb9	-2.8701	0.0072	0.4078	Down
Fbxo34	-2.5037	0.0074	0.4078	Down
Rasl12	-1.7988	0.0074	0.4078	Down
Rnf213	-2.7842	0.0075	0.4078	Down
Srpr	-3.3408	0.0075	0.4078	Down
Pdzd7	-2.6432	0.0075	0.4078	Down
Mfsd1	-3.3951	0.0077	0.4078	Down
Pcyox1	-2.8081	0.0078	0.4078	Down
Ptpn5	-4.6946	0.0079	0.4078	Down
Srpk1	-3.9106	0.008	0.4078	Down
Slc2a13	-4.1244	0.0081	0.4078	Down
Rhbdf2	-2.7401	0.0082	0.4078	Down
Faf1	-2.7216	0.0083	0.4078	Down
Gm2423	-4.2323	0.0083	0.4078	Down
Pld4	-2.9487	0.0083	0.4078	Down
Sgcb	-3.1106	0.0083	0.4078	Down
C4a	-1.5694	0.0084	0.4078	Down
Gm11734	-4.3659	0.0085	0.4078	Down
Npepps	-2.3621	0.0086	0.4078	Down
Spdya	-4.054	0.0087	0.4078	Down
Mapk6	-2.9354	0.0087	0.4078	Down
Msh3	-3.2765	0.0087	0.4078	Down
Gm6563	-1.6547	0.0088	0.4078	Down
H2-K1	-3.6205	0.0088	0.4078	Down
Sh3bgrl	-4.2864	0.0089	0.4078	Down
Il10ra	-3.5402	0.009	0.4078	Down
Lrrcc1	-3.9669	0.0091	0.4078	Down
Zfp984	-3.1061	0.0095	0.4078	Down
Fam110b	-3.3962	0.0096	0.4078	Down
Tmem151b	-2.2402	0.0097	0.4078	Down
Gm35040	-1.877	0.0098	0.4078	Down
Lrwd1	-2.2532	0.0099	0.4078	Down
Rnf214	-2.8159	0.01	0.4078	Down
Ppp1r10	4.2516	0	0.0503	Up
Uqcr11	3.3121	0	0.0503	Up
Mt3	3.4151	0	0.0503	Up
Prkg1	5.2386	0.0001	0.2633	Up
Sem1	5.2267	0.0001	0.2633	Up
Fkbp2	3.2348	0.0001	0.2633	Up
Cst3	2.7714	0.0001	0.2633	Up
Hint1	3.4952	0.0001	0.2633	Up
Rpl34	2.5306	0.0002	0.3092	Up
Atp5c1	3.0595	0.0003	0.3092	Up
Ldlr	4.8397	0.0003	0.3092	Up
Mdk	2.1662	0.0003	0.3092	Up

Fam71e1	4.5984	0.0003	0.3092	Up
Micos13	2.947	0.0003	0.3092	Up
Myt1l	3.0296	0.0003	0.3092	Up
Celf1	3.1318	0.0004	0.3122	Up
Ckb	3.113	0.0005	0.3122	Up
Antkmt	5.4428	0.0005	0.3122	Up
Gm12751	4.9013	0.0005	0.3122	Up
Elob	4.0644	0.0005	0.3122	Up
Rps19	3.0166	0.0005	0.3122	Up
Pebp1	3.7337	0.0006	0.3122	Up
Cox6c	2.0635	0.0006	0.3122	Up
Rrad	2.5751	0.0006	0.3122	Up
Mrps11	3.2341	0.0006	0.3122	Up
Ppid	3.3557	0.0006	0.3131	Up
Rps21	2.4672	0.0008	0.3302	Up
C1qtnf12	3.3411	0.0008	0.3302	Up
Atxn1l	3.9118	0.0008	0.3302	Up
Rps27a-ps2	5.8722	0.0008	0.3302	Up
Usp35	4.189	0.0008	0.3302	Up
Polr2k	1.8263	0.0008	0.3302	Up
Tomm7	3.9729	0.0009	0.3302	Up
Mag	2.9686	0.0009	0.3302	Up
Tns1	4.3057	0.0009	0.3302	Up
Gm27032	3.076	0.0009	0.3302	Up
Sarnp	2.25	0.0009	0.3302	Up
Acot7	5.4396	0.001	0.3302	Up
Dock6	3.1215	0.001	0.3302	Up
Ndufa1	3.0681	0.001	0.3302	Up
Cox7b	2.4034	0.001	0.3302	Up
Rplp1	3.0091	0.001	0.3302	Up
Aff1	2.4528	0.0011	0.3302	Up
Zic1	2.3449	0.0011	0.3302	Up
Cox14	4.0429	0.0011	0.3374	Up
Ston2	2.1427	0.0012	0.3374	Up
Fam174a	3.2892	0.0012	0.3374	Up
Tmem9b	3.8563	0.0012	0.3374	Up
Gm8066	4.3845	0.0012	0.3374	Up
Rpl31	3.125	0.0013	0.3449	Up
Mcts2	5.0081	0.0013	0.3449	Up
Phyhipl	2.5043	0.0014	0.3758	Up
Cbln2	3.5839	0.0016	0.3904	Up
3830408C21Rik	4.2572	0.0016	0.3904	Up
Emd	3.916	0.0017	0.3955	Up
Meltf	6.4312	0.0017	0.3955	Up
Polr3h	4.0062	0.0018	0.3982	Up
Supt4a	2.1369	0.0019	0.3982	Up
Chchd1	2.3612	0.0019	0.3982	Up
Tcf3	4.1619	0.0019	0.3982	Up
Slc22a17	4.5006	0.002	0.4051	Up
Tanc1	3.1457	0.0021	0.4057	Up
Col6a3	2.8331	0.0021	0.406	Up
Exoc2	3.9774	0.0022	0.406	Up
AW047730	4.0824	0.0023	0.406	Up
Commd6	3.8092	0.0023	0.406	Up
Dtx3l	2.5921	0.0023	0.406	Up
Focad	5.2248	0.0024	0.406	Up

Pcdhga7	2.8184	0.0024	0.406	Up
Zbtb7c	2.0234	0.0025	0.406	Up
Gapdh	4.2086	0.0025	0.406	Up
Kif6	2.8326	0.0026	0.406	Up
Gm9821	3.3626	0.0027	0.406	Up
Snrpd3	3.1783	0.0027	0.406	Up
Fa2h	2.8835	0.0027	0.406	Up
Uvrag	4.3867	0.0028	0.406	Up
Ltbp4	5.018	0.0028	0.406	Up
Isca1	2.8666	0.0028	0.406	Up
Psmb3	3.4314	0.0029	0.406	Up
D430041D05Rik	3.0664	0.0029	0.406	Up
Zbtb37	3.5721	0.0029	0.406	Up
Higd1a	5.0228	0.0029	0.406	Up
mt-Nd2	1.9905	0.003	0.406	Up
Eno2	2.2366	0.003	0.406	Up
Rev1	4.0885	0.003	0.406	Up
Rps8	2.0907	0.003	0.406	Up
Ube2b	2.7166	0.0031	0.406	Up
Snap25	5.7388	0.0031	0.406	Up
Atp10a	3.5713	0.0031	0.406	Up
Hspbp1	6.5859	0.0032	0.406	Up
Tbc1d9	3.2931	0.0033	0.406	Up
Cadm4	3.204	0.0033	0.406	Up
Reps2	3.2156	0.0033	0.406	Up
Cd53	2.1443	0.0033	0.406	Up
Hmcn2	3.8791	0.0034	0.4078	Up
Lims2	2.3457	0.0034	0.4078	Up
Sdk1	3.5014	0.0035	0.4078	Up
Gm1673	3.4275	0.0036	0.4078	Up
Tomm22	2.5173	0.0036	0.4078	Up
Gm20515	3.5968	0.0036	0.4078	Up
Adam10	2.7972	0.0036	0.4078	Up
C130021I20Rik	2.5268	0.0037	0.4078	Up
Atg5	2.8681	0.0037	0.4078	Up
Camk2n2	2.7252	0.0039	0.4078	Up
Alg1	5.4696	0.0039	0.4078	Up
Pfn1	2.5132	0.0039	0.4078	Up
Cnp	2.9218	0.004	0.4078	Up
Gm10036	3.1038	0.0041	0.4078	Up
Xpo5	3.8079	0.0043	0.4078	Up
ENSMUSG00000120970	2.2958	0.0043	0.4078	Up
Lama5	2.8256	0.0044	0.4078	Up
Rsph1	4.2529	0.0044	0.4078	Up
Pfdn4	2.6343	0.0045	0.4078	Up
Ube2d3	2.8948	0.0046	0.4078	Up
Pdpr	2.748	0.0047	0.4078	Up
Ppp1r11	5.8206	0.0047	0.4078	Up
Fuom	2.7948	0.0047	0.4078	Up
2300009A05Rik	3.0025	0.0048	0.4078	Up
Nrip2	4.5013	0.005	0.4078	Up
Zfp827	4.4232	0.005	0.4078	Up
Rps20	1.8981	0.005	0.4078	Up
Gm6206	1.8485	0.005	0.4078	Up
Flrt1	1.9115	0.0051	0.4078	Up
Cdyl2	3.2633	0.0051	0.4078	Up

Vcp-rs	2.2264	0.0051	0.4078	Up
Acsl1	5.2967	0.0052	0.4078	Up
Nav2	4.0104	0.0052	0.4078	Up
Gm15427	3.2302	0.0052	0.4078	Up
Chpf2	4.1529	0.0052	0.4078	Up
Cul4a	2.212	0.0052	0.4078	Up
Hes5	2.4652	0.0052	0.4078	Up
Gosr2	2.4167	0.0053	0.4078	Up
Snap47	2.3684	0.0053	0.4078	Up
Pcnt	4.1048	0.0053	0.4078	Up
Uchl3	3.6496	0.0054	0.4078	Up
Sh3pxd2a	3.1655	0.0056	0.4078	Up
Timm10	4.1804	0.0056	0.4078	Up
Uqcrc2	3.6259	0.0056	0.4078	Up
Slu7	2.909	0.0057	0.4078	Up
Apln	1.9504	0.0057	0.4078	Up
Mobp	5.0213	0.0058	0.4078	Up
Megf6	2.1474	0.0058	0.4078	Up
Bmpr1a	3.0743	0.0058	0.4078	Up
Med12	3.3241	0.0059	0.4078	Up
Sdr39u1	3.5552	0.006	0.4078	Up
Ndufa4	2.5011	0.0061	0.4078	Up
Sqle	3.4258	0.0061	0.4078	Up
Fundc1	4.2816	0.0061	0.4078	Up
Tmbim1	2.7134	0.0062	0.4078	Up
Chchd6	2.7117	0.0062	0.4078	Up
Pptc7	2.6435	0.0062	0.4078	Up
Atp6v0b	3.9749	0.0062	0.4078	Up
Mbd6	3.2418	0.0062	0.4078	Up
Snu13	2.7096	0.0063	0.4078	Up
Grk3	2.569	0.0063	0.4078	Up
Zswim7	3.1058	0.0063	0.4078	Up
Cspg4b	2.9097	0.0063	0.4078	Up
Psmb5	2.5772	0.0064	0.4078	Up
Gm26703	3.3797	0.0064	0.4078	Up
Fbxl15	2.8088	0.0064	0.4078	Up
Mrps17	4.6413	0.0065	0.4078	Up
Tm6sf1	4.5934	0.0065	0.4078	Up
Gpalpp1	2.9486	0.0065	0.4078	Up
Adamtsl1	4.6797	0.0066	0.4078	Up
Zfp974	3.4019	0.0067	0.4078	Up
Pfas	3.9186	0.0067	0.4078	Up
Retreg2	3.942	0.0067	0.4078	Up
Itpkb	2.9695	0.0067	0.4078	Up
Akap1	3.3851	0.0068	0.4078	Up
Ran	2.184	0.0068	0.4078	Up
Ndufa5	2.1033	0.0068	0.4078	Up
Tpcn1	2.8639	0.0069	0.4078	Up
Trpc4	3.3622	0.0069	0.4078	Up
Atp5e	1.6602	0.0069	0.4078	Up
Sspn	2.6149	0.0069	0.4078	Up
Sirt7	2.734	0.007	0.4078	Up
Kat6b	3.6814	0.007	0.4078	Up
Gm14295	2.5267	0.007	0.4078	Up
Rnf169	2.9549	0.0071	0.4078	Up
Mif	1.945	0.0071	0.4078	Up

Rbm5	2.9622	0.0071	0.4078	Up
Lpcat2	3.1877	0.0071	0.4078	Up
Gm14117	1.9346	0.0072	0.4078	Up
S1pr5	3.3215	0.0072	0.4078	Up
Ntn1	2.957	0.0072	0.4078	Up
Dapk3	2.4536	0.0073	0.4078	Up
C1qtnf4	3.5523	0.0074	0.4078	Up
Ramp1	2.5258	0.0075	0.4078	Up
Pkn1	3.4512	0.0075	0.4078	Up
AI467606	2.8788	0.0075	0.4078	Up
Emc9	2.1808	0.0076	0.4078	Up
Mtln	2.8711	0.0076	0.4078	Up
Septin4	2.4929	0.0076	0.4078	Up
Nr2c2	3.023	0.0076	0.4078	Up
Hagh	3.2601	0.0077	0.4078	Up
Ar	3.3691	0.0077	0.4078	Up
Invs	4.198	0.0077	0.4078	Up
Bcap31	2.9629	0.0078	0.4078	Up
Sin3a	3.0069	0.0079	0.4078	Up
Gamt	2.9735	0.0079	0.4078	Up
Calb2	3.8079	0.0079	0.4078	Up
Prelid1	2.7994	0.0079	0.4078	Up
Ctdsp2	3.746	0.008	0.4078	Up
Psmc4	2.954	0.008	0.4078	Up
Rps29	1.6416	0.0081	0.4078	Up
Phactr3	3.1657	0.0082	0.4078	Up
Ftl1	1.895	0.0082	0.4078	Up
Kl	1.6336	0.0083	0.4078	Up
Slc4a2	2.5905	0.0083	0.4078	Up
Car2	3.8076	0.0083	0.4078	Up
Rdm1	3.0427	0.0084	0.4078	Up
Mlh3	3.8287	0.0084	0.4078	Up
Pdgfa	3.7624	0.0084	0.4078	Up
Pcdhgb5	3.1203	0.0084	0.4078	Up
Foxk1	3.5914	0.0085	0.4078	Up
Tnrc6c	3.5344	0.0086	0.4078	Up
BC031181	3.943	0.0086	0.4078	Up
Csde1	2.3368	0.0086	0.4078	Up
Ercc3	3.0577	0.0087	0.4078	Up
Nsd2	3.9683	0.0087	0.4078	Up
Elp1	3.2032	0.0087	0.4078	Up
Gemin7	2.1351	0.0088	0.4078	Up
Ssbp4	2.741	0.0088	0.4078	Up
Rpl22l1	1.2939	0.0088	0.4078	Up
Scn1b	5.7536	0.0088	0.4078	Up
Map3k7	3.7246	0.0089	0.4078	Up
Mlx	1.6652	0.0089	0.4078	Up
Sidt2	2.0533	0.009	0.4078	Up
Gm15612	3.8993	0.009	0.4078	Up
Cplx1	1.5465	0.009	0.4078	Up
Gm14164	3.1241	0.0091	0.4078	Up
Ap2m1-ps	2.0934	0.0091	0.4078	Up
Trmt112	2.2786	0.0091	0.4078	Up
Map3k9	2.211	0.0091	0.4078	Up
Gm43481	2.371	0.0092	0.4078	Up
Dhx35	3.5092	0.0092	0.4078	Up

Pcdhga8	2.7545	0.0092	0.4078	Up
Atp5g1	2.0708	0.0092	0.4078	Up
Ndufs2	2.5681	0.0092	0.4078	Up
Tomm5	3.4543	0.0092	0.4078	Up
mt-Rnr2	3.7893	0.0093	0.4078	Up
Cmpk2	2.3347	0.0093	0.4078	Up
Epc1	3.0153	0.0094	0.4078	Up
Trappc13	3.694	0.0094	0.4078	Up
Bod1l	2.5835	0.0094	0.4078	Up
Per3	1.9554	0.0095	0.4078	Up
Ccdc137	2.4799	0.0096	0.4078	Up
Cyp51	2.1383	0.0096	0.4078	Up
Ccdc3	1.575	0.0097	0.4078	Up
Zfp369	2.8795	0.0097	0.4078	Up
Cda	2.6475	0.0098	0.4078	Up
Cd200	2.5876	0.0098	0.4078	Up
Rreb1	2.4872	0.0098	0.4078	Up
Dynll1	3.0853	0.0099	0.4078	Up
Proca1	2.1565	0.0099	0.4078	Up
Cybc1	4.119	0.0099	0.4078	Up
Penk	2.1199	0.01	0.4078	Up
Dpcd	2.695	0.01	0.4078	Up
**SNI: TRF_A_VS_ALF_A**				
Gapdh	-4.0099	0	0.0029	Down
Gm15427	-3.873	0.0001	0.2204	Down
Pdcd6	-2.6399	0.0001	0.3362	Down
Psmd5	-5.4673	0.0003	0.3525	Down
Gm43305	-3.7951	0.0004	0.3525	Down
Ugt8a	-2.3153	0.0005	0.3525	Down
Gfm1	-4.0515	0.0005	0.3525	Down
Hook1	-3.4992	0.0006	0.3525	Down
Palmd	-3.1168	0.0006	0.3525	Down
Rps12-ps3	-2.8398	0.0007	0.3672	Down
Ppp1r11	-5.5459	0.0007	0.3672	Down
Gatad2b	-4.1161	0.0008	0.3764	Down
Rab1b	-3.9631	0.0009	0.4115	Down
Hspbp1	-7.0759	0.0009	0.4189	Down
Csf3r	-3.1908	0.0011	0.4209	Down
Pcdhb14	-2.0256	0.0012	0.4509	Down
Akr7a5	-1.9763	0.0013	0.4578	Down
Rpl37	-2.7976	0.0013	0.4578	Down
Icam5	-3.9904	0.0015	0.4597	Down
Tmem88b	-4.0795	0.0016	0.4597	Down
Rsph1	-3.8192	0.0017	0.4597	Down
Rnf168	-3.851	0.0017	0.4597	Down
Myh7	-2.5401	0.0017	0.4597	Down
Setdb1	-3.395	0.0018	0.4597	Down
1810059H22Rik	-4.3783	0.0019	0.4597	Down
Rab5a	-2.806	0.002	0.4609	Down
Ubl7	-3.3624	0.0021	0.4609	Down
Kcnk12	-2.5652	0.0022	0.4671	Down
Hsf4	-3.1686	0.0023	0.4671	Down
Babam1	-2.1579	0.0023	0.4671	Down
Arl6ip6	-2.2657	0.0024	0.4671	Down
Vcp-rs	-1.9781	0.0025	0.4671	Down
Mkln1os	-2.5835	0.0027	0.4671	Down

Xkrx	-5.8637	0.0027	0.4671	Down
Fntb	-2.103	0.0027	0.4671	Down
Mib1	-2.9837	0.0028	0.4671	Down
Kras	-3.4856	0.0028	0.4671	Down
5430405H02Rik	-2.5269	0.0028	0.4671	Down
Retreg2	-4.0926	0.0032	0.4671	Down
4930503L19Rik	-2.2883	0.0032	0.4671	Down
Vit	-3.0019	0.0033	0.4671	Down
Rplp2	-2.1061	0.0033	0.4671	Down
Rcbtb1	-3.7088	0.0033	0.4671	Down
Tspan7	-3.2297	0.0034	0.4671	Down
Zfp263	-4.4196	0.0035	0.4671	Down
Nus1	-3.5714	0.0035	0.4671	Down
Eps15	-4.9737	0.0036	0.4671	Down
Eps8l1	-3.7283	0.0038	0.4671	Down
Ssbp4	-4.1828	0.0038	0.4671	Down
Tmtc3	-3.2766	0.0038	0.4671	Down
Ndufb7	-2.5066	0.0038	0.4671	Down
Cul2	-6.1634	0.0039	0.4671	Down
Zfp768	-3.3639	0.0041	0.4671	Down
Ctps2	-3.7435	0.0042	0.4671	Down
Emc9	-2.7509	0.0045	0.4671	Down
Rogdi	-2.037	0.0045	0.4671	Down
Cobll1	-2.9237	0.0045	0.4671	Down
Mrpl55	-4.0086	0.0046	0.4671	Down
Mcm7	-3.3783	0.0047	0.4671	Down
Tmem30a	-3.888	0.0047	0.4671	Down
Emc1	-3.4842	0.0048	0.4671	Down
Gm36937	-3.6307	0.0048	0.4671	Down
Csrnp3	-3.9361	0.0049	0.4671	Down
Wdr83os	-2.4927	0.0049	0.4671	Down
Stk32a	-4.1568	0.0049	0.4671	down
Ltbp4	-3.9409	0.005	0.4671	down
Gm19938	-2.7547	0.0052	0.4671	down
Aldoa	-2.726	0.0052	0.4671	down
Dnm1l	-2.8468	0.0052	0.4671	down
Adamts1	-3.5275	0.0052	0.4671	down
Rtn4	-4.7179	0.0053	0.4671	down
Gm12346	-2.385	0.0053	0.4671	down
Mcts2	-5.5708	0.0053	0.4671	down
Casd1	-4.8651	0.0055	0.4671	down
St3gal3	-3.3937	0.0055	0.4671	down
Rbms3	-3.4536	0.0057	0.4671	down
Mbnl2	-4.2473	0.0057	0.4671	down
Peli3	-3.1118	0.0057	0.4671	down
Gm13835	-4.3517	0.0058	0.4671	down
Fuca1	-4.7049	0.0058	0.4671	down
Ube2d3	-4.3714	0.0058	0.4671	down
Slc17a6	-3.2199	0.0059	0.4671	down
Gtf3a	-4.1053	0.0059	0.4671	down
Kif5b	-3.2047	0.006	0.4671	down
Rab3a	-4.5195	0.0061	0.4671	down
L3mbtl2	-2.9442	0.0061	0.4671	down
Zfp282	-2.4219	0.0062	0.4671	down
Tsnaxip1	-3.4916	0.0062	0.4671	down
Sgce	-2.8446	0.0062	0.4671	down

Erbb4	-3.7306	0.0063	0.4671	down
Naaa	-3.769	0.0064	0.4671	down
Nvl	-3.6425	0.0065	0.4671	down
Hagh	-3.6843	0.0065	0.4671	down
Thumpd1	-2.3425	0.0066	0.4671	down
3000002C10Rik	-2.6559	0.0066	0.4671	down
Ptov1	-3.9444	0.0067	0.4671	down
Ppp3cb	-2.6655	0.0067	0.4671	down
Gm49204	-3.7943	0.007	0.4671	down
Limd2	-5.1498	0.0072	0.4671	down
Polr3b	-4.5892	0.0072	0.4671	down
Nrsn2	-3.5281	0.0073	0.4671	down
Szrd1	-2.869	0.0074	0.4671	down
Zfp711	-4.9315	0.0074	0.4671	down
Atg4c	-2.8881	0.0074	0.4671	down
Fam234a	-2.6324	0.0074	0.4671	down
Nt5c2	-2.4344	0.0075	0.4671	down
AW146154	-3.0676	0.0075	0.4671	down
Tmem68	-2.6918	0.0075	0.4671	down
Mkln1	-5.1154	0.0075	0.4671	down
0610012G03Rik	-3.0264	0.0075	0.4671	down
Acd	-3.3994	0.0075	0.4671	down
Gdap1l1	-2.9438	0.0076	0.4671	down
Ints6	-2.7144	0.0077	0.4671	down
Cdipt	-3.6224	0.0077	0.4671	down
Dpy19l4	-5.1553	0.0079	0.4671	down
Sms	-2.2757	0.0079	0.4671	down
Coq2	-3.9129	0.0079	0.4671	down
Pld3	-3.664	0.0079	0.4671	down
Cdk9	-4.6418	0.0082	0.4685	down
Gadd45g	-2.2507	0.0082	0.4685	down
Gm14410	-1.824	0.0082	0.4685	down
Psip1	-3.7566	0.0083	0.4698	down
Eml2	-4.2714	0.0083	0.4698	down
Ckb	-3.9574	0.0084	0.4711	down
Cpeb4	-2.8426	0.0084	0.4711	down
Cpsf6	-3.53	0.0087	0.4763	down
Capns1	-3.7832	0.0087	0.4763	down
mt-Rnr2	-2.0518	0.0089	0.4772	down
Slc22a17	-4.3836	0.009	0.4783	down
Dhdds	-1.9853	0.0091	0.4783	down
Copz1	-5.0378	0.0091	0.4783	down
Gm14117	-2.3186	0.0091	0.4783	down
Rtl8c	-3.5036	0.0094	0.4843	down
Rplp1	-3.0521	0.0095	0.4843	down
Gm9844	-2.9087	0.0095	0.4843	down
Nae1	-3.2401	0.0095	0.4843	down
C130021I20Rik	-3.0856	0.0096	0.4843	down
Nsmf	-3.3443	0.0097	0.4843	down
Necab2	-2.5682	0.0098	0.4875	down
Psmb3	-3.1508	0.0098	0.4875	down
Dkc1	-1.8513	0.0099	0.4875	down
Pigb	-2.5148	0.0099	0.4875	down
Sptan1	6.0092	0	0.2204	up
Egr1	3.1105	0	0.2204	up
Stx6	2.2547	0.0001	0.2807	up

Tro	3.5907	0.0002	0.3362	up
Brca2	3.0258	0.0002	0.3525	up
Cers6	3.3859	0.0002	0.3525	up
Kif21b	2.8309	0.0003	0.3525	up
Glul	2.7451	0.0004	0.3525	up
Itpr1	4.214	0.0005	0.3525	up
Btg2	2.0731	0.0005	0.3525	up
Gm5424	1.7844	0.0005	0.3525	up
Fam107a	1.8459	0.0005	0.3525	up
Vwf	2.1769	0.0005	0.3525	up
Ddo	4.128	0.0005	0.3525	up
Tmcc2	4.9435	0.0006	0.3525	up
Gm13803	5.5419	0.0006	0.3525	up
Jph4	3.2274	0.0006	0.3525	up
Immp1l	3.4407	0.0007	0.3672	up
Srsf5	2.6666	0.0007	0.3672	up
Arhgef19	2.3588	0.001	0.4209	up
Hnrnpa1	2.8421	0.001	0.4209	up
Ap4s1	3.7788	0.001	0.4209	up
Cdk5r2	2.287	0.001	0.4209	up
Plxna1	2.9987	0.0011	0.4209	up
4921507P07Rik	4.528	0.0011	0.4209	up
Mipol1	2.968	0.0012	0.4351	up
Hjurp	2.9354	0.0014	0.4597	up
Sec11c	3.5668	0.0015	0.4597	up
Ilrun	2.8391	0.0015	0.4597	up
Mical2	2.898	0.0015	0.4597	up
Gad1	2.59	0.0016	0.4597	up
Rxra	3.308	0.0017	0.4597	up
Nxpe4	2.3124	0.0018	0.4597	up
Tob1	2.1965	0.0019	0.4597	up
Syn2	3.8466	0.0019	0.4597	up
Trh	4.4514	0.0019	0.4597	up
Swap70	3.1205	0.0019	0.4597	up
Abca2	2.8487	0.0019	0.4597	up
Rsph10b	2.8711	0.002	0.4597	up
Gm9843	3.0654	0.002	0.4597	up
Ydjc	2.9722	0.0021	0.4609	up
Ralgapa1	4.2607	0.0021	0.4609	up
Nfib	5.4454	0.0023	0.4671	up
P4ha2	3.6403	0.0023	0.4671	up
Exoc4	3.8896	0.0024	0.4671	up
Gtf2h5	3.6053	0.0025	0.4671	up
Zfp810	3.4065	0.0025	0.4671	up
Tob2	1.8457	0.0025	0.4671	up
Pnma8b	3.1094	0.0026	0.4671	up
Alkbh5	1.9141	0.0026	0.4671	up
Klhl17	4.1063	0.0028	0.4671	up
Timp2	3.4441	0.0028	0.4671	up
Fastkd2	2.2815	0.0028	0.4671	up
Atg16l2	3.1686	0.0028	0.4671	up
Rab11fip3	2.9722	0.0029	0.4671	up
Tceal9	3.8313	0.0029	0.4671	up
Mtss2	2.6615	0.0031	0.4671	up
Aldob	3.0038	0.0032	0.4671	up
Samd9l	4.1147	0.0032	0.4671	up

Syngap1	1.9837	0.0032	0.4671	up
Zfp110	3.3359	0.0034	0.4671	up
Ksr1	3.3757	0.0035	0.4671	up
Gnao1	4.1464	0.0039	0.4671	up
9030025P20Rik	2.5773	0.0039	0.4671	up
Ptprs	4.6539	0.0039	0.4671	up
Sfxn5	2.9379	0.0041	0.4671	up
Zkscan6	2.0079	0.0041	0.4671	up
Kbtbd11	3.1631	0.0041	0.4671	up
Phrf1	2.473	0.0042	0.4671	up
Gad2	3.4165	0.0043	0.4671	up
Bmp7	2.9869	0.0043	0.4671	up
Dync1h1	2.2009	0.0044	0.4671	up
Bub1b	2.5649	0.0045	0.4671	up
Slc6a13	3.4467	0.0045	0.4671	up
Acot11	2.6289	0.0045	0.4671	up
Map2k6	3.875	0.0045	0.4671	up
Ppp1r9b	3.9362	0.0046	0.4671	up
Zmym3	4.7534	0.0047	0.4671	up
Adcy6	3.2627	0.0047	0.4671	up
Hmgcs1	3.2226	0.0047	0.4671	up
Slc15a2	1.4394	0.0048	0.4671	up
Paqr8	1.7519	0.0049	0.4671	up
Tac1	2.0512	0.0049	0.4671	up
Srrm2	3.8683	0.0051	0.4671	up
Lamc3	2.5114	0.0052	0.4671	up
Lrwd1	3.2298	0.0053	0.4671	up
Sowaha	3.7954	0.0053	0.4671	up
Apba2	2.8485	0.0054	0.4671	up
Gga2	1.8221	0.0055	0.4671	up
Rab3gap1	2.701	0.0056	0.4671	up
Acadl	3.0729	0.0058	0.4671	up
Aldh1a1	3.3117	0.0058	0.4671	up
Fcgrt	2.6999	0.0058	0.4671	up
Zfp882	2.0836	0.0059	0.4671	up
Mrpl9	3.8235	0.0059	0.4671	up
Rfk	3.7796	0.0059	0.4671	up
Nbeal2	5.3642	0.006	0.4671	up
Slc23a2	3.2704	0.0061	0.4671	up
Patl1	2.6971	0.0061	0.4671	up
Irs2	4.7	0.0061	0.4671	up
Mpg	2.374	0.0062	0.4671	up
Sf3a1	1.9301	0.0062	0.4671	up
Rad18	2.5142	0.0062	0.4671	up
Fbxo34	2.3397	0.0063	0.4671	up
Cdk16	2.7135	0.0063	0.4671	up
Noct	2.5001	0.0064	0.4671	up
Spsb3	2.5403	0.0065	0.4671	up
Midn	2.6459	0.0065	0.4671	up
Pabpc1	3.7559	0.0065	0.4671	up
Arhgap44	4.3364	0.0065	0.4671	up
Cops4	3.4463	0.0066	0.4671	up
Adarb2	4.3247	0.0067	0.4671	up
Acta2	1.812	0.0067	0.4671	up
Bax	1.5808	0.0068	0.4671	up
Ganab	2.3433	0.0068	0.4671	up

Timp3	2.4569	0.0068	0.4671	up
H1f0	2.4892	0.0068	0.4671	up
Tmem18	2.8217	0.0069	0.4671	up
Hif3a	1.6384	0.0069	0.4671	up
Tmem151b	2.2661	0.0069	0.4671	up
Trf	2.3741	0.007	0.4671	up
Atp5mpl	2.4122	0.0071	0.4671	up
Mroh1	2.6405	0.0071	0.4671	up
Thsd4	3.8038	0.0072	0.4671	up
Mmp17	3.3346	0.0072	0.4671	up
Lrrc47	2.1306	0.0072	0.4671	up
Nfil3	3.4754	0.0073	0.4671	up
Lrrn1	2.5039	0.0073	0.4671	up
Bicc1	2.0702	0.0074	0.4671	up
Irf9	2.6738	0.0075	0.4671	up
Klhl3	3.2694	0.0075	0.4671	up
Pold3	2.9822	0.0075	0.4671	up
Ccpg1os	2.8215	0.0076	0.4671	up
Tiparp	2.0846	0.0076	0.4671	up
Scara3	3.1383	0.0077	0.4671	up
Rph3a	3.9442	0.0077	0.4671	up
Zfp14	1.9352	0.0078	0.4671	up
5031439G07Rik	2.2269	0.0078	0.4671	up
Grm2	2.7768	0.0079	0.4671	up
Basp1	3.778	0.008	0.4675	up
Slc6a20a	2.9856	0.0081	0.4675	up
Cacng2	5.8964	0.0081	0.4675	up
Pon2	2.7724	0.0081	0.4675	up
P3h3	3.2677	0.0081	0.4675	up
Polr2k	3.8512	0.0083	0.4698	up
Dnal4	3.3842	0.0084	0.4711	up
Cerox1	2.3538	0.0086	0.4763	up
Gm38020	4.1636	0.0086	0.4763	up
Ankrd13d	2.1402	0.0087	0.4763	up
Vps29	2.0844	0.0087	0.4772	up
Gbp7	3.6081	0.0088	0.4772	up
Vps33b	5.2991	0.0088	0.4772	up
Grid1	3.9931	0.0089	0.4772	up
Bmp6	3.1965	0.0089	0.4772	up
Xpo6	2.1177	0.0089	0.4776	up
Aamdc	3.0852	0.0089	0.4776	up
Fam76a	4.9126	0.0091	0.4783	up
Mical3	2.0485	0.0092	0.4791	up
Gtf3c1	4.6776	0.0093	0.4818	up
Carmil3	4.2126	0.0093	0.4818	up
Cxadr	2.8561	0.0095	0.4843	up
Shkbp1	1.8942	0.0095	0.4843	up
Tmem62	4.498	0.0096	0.4843	up
Nf1	2.7388	0.0096	0.4843	up
Mrps15	4.5833	0.0099	0.4875	up
Fbxo17	2.5377	0.01	0.4909	up
**sham: TRF_M_VS_A**				
Sgk1	-3.772	0	0.0977	down
Tbcb	-3.0125	0	0.0977	down
Nr2f6	-4.3697	0	0.0977	down
Trim8	-3.7425	1.00E-04	0.0994	down

Mfap1b	-2.0913	1.00E-04	0.0994	down
Irf2bpl	-2.6924	1.00E-04	0.0994	down
Ndufa12	-2.0603	2.00E-04	0.0994	down
Tlnrd1	-3.0347	2.00E-04	0.0994	down
Zfp219	-3.4227	2.00E-04	0.1075	down
Cacng8	-2.8043	2.00E-04	0.1075	down
Phf23	-4.314	4.00E-04	0.1268	down
Aoc2	-4.9714	4.00E-04	0.1268	down
Mex3d	-4.0088	5.00E-04	0.1458	down
Dld	-4.852	7.00E-04	0.1667	down
Map7d1	-2.0906	0.0011	0.1911	down
Tmem267	-4.0733	0.0011	0.1911	down
Armc1	-3.9633	0.0011	0.1911	down
Nt5c2	-3.1172	0.0012	0.1937	down
Mzt1	-4.5215	0.0014	0.1989	down
Nacad	-2.6724	0.0015	0.202	down
Fbxo22	-3.3761	0.0015	0.202	down
Ccdc97	-3.5285	0.0015	0.202	down
Zfp280d	-2.7821	0.0016	0.202	down
2900052L18Rik	-4.3704	0.0017	0.2054	down
Gpm6a	-4.7883	0.0018	0.2062	down
Marchf7	-3.5244	0.0022	0.2278	down
Midn	-2.8141	0.0023	0.2278	down
Dapk3	-3.7665	0.0024	0.2278	down
Nfkbia	-3.9229	0.0025	0.2278	down
Tnfrsf25	-4.2345	0.0025	0.2278	down
Pxdc1	-3.2611	0.0027	0.2356	down
Neurl1a	-3.321	0.0029	0.2373	down
Fam171a2	-1.6758	0.0029	0.2373	down
Nr4a3	-2.5357	0.0031	0.2447	down
Pals2	-3.142	0.0032	0.2471	down
Loxl1	-2.5638	0.0034	0.2512	down
Mcl1	-3.4058	0.0034	0.2512	down
Srrm3	-1.9461	0.0034	0.2512	down
Guf1	-2.6978	0.0035	0.2512	down
Kcnk3	-2.3347	0.0036	0.2519	down
Cep70	-3.6224	0.0037	0.2519	down
Nol11	-2.8084	0.0039	0.2519	down
Alg9	-2.7862	0.0039	0.2519	down
Mid1	-4.559	0.0039	0.2519	down
Rpl7a	-1.4742	0.004	0.2519	down
Cnot2	-2.5776	0.0041	0.2519	down
Marcks	-3.6285	0.0041	0.2519	down
Dtx2	-3.6897	0.0042	0.2519	down
Otud1	-1.941	0.0043	0.2519	down
Invs	-4.9496	0.0045	0.256	down
Prpf40b	-2.6383	0.0046	0.2576	down
Agbl4	-3.6861	0.0047	0.2586	down
Hspb6	-3.7896	0.0049	0.2608	down
Slc38a6	-4.392	0.0052	0.2683	down
Ltbr	-2.3704	0.0052	0.2683	down
Cacna2d3	-2.6686	0.0054	0.2707	down
Samd14	-3.3813	0.0055	0.273	down
Slc25a28	-2.8149	0.0056	0.273	down
Slc38a10	-2.9656	0.0057	0.2742	down
Cdkn2aipnl	-2.6095	0.0057	0.2748	down

Marchf9	-2.9536	0.0057	0.2748	down
Pura	-2.8311	0.0058	0.2748	down
Jun	-1.7538	0.0058	0.2748	down
Irf2bp2	-2.574	0.006	0.2821	down
Prdm5	-3.5325	0.0062	0.2836	down
Gm15663	-2.3821	0.0062	0.2836	down
Cpsf7	-4.0214	0.0063	0.2836	down
Cldn5	-4.4418	0.0063	0.2836	down
Csf2rb2	-2.611	0.0064	0.2836	down
Hccs	-3.1326	0.0064	0.2836	down
Fam3c	-3.4435	0.0065	0.2847	down
Skil	-2.2637	0.0065	0.2847	down
Gm43305	-2.7942	0.0066	0.2847	down
Zfp553	-2.7616	0.0068	0.2847	down
Pes1	-3.2852	0.0072	0.2852	down
Rcan1	-2.7253	0.0072	0.2852	down
Zfp523	-3.4777	0.0073	0.2852	down
Zpr1	-2.5491	0.0074	0.2852	down
Stt3b	-2.9928	0.0074	0.2852	down
AI593442	-2.5006	0.0075	0.2852	down
Jph4	-1.8905	0.0077	0.2882	down
Maz	-2.114	0.0077	0.2889	down
Frat2	-2.9531	0.0078	0.2907	down
Basp1	-2.342	0.0079	0.2914	down
Slc16a9	-4.6197	0.0082	0.2944	down
Dusp6	-1.6848	0.0086	0.2967	down
Jmjd6	-1.6416	0.0089	0.2998	down
Crip3	-2.7319	0.0089	0.2998	down
Dsg2	-3.21	0.009	0.2998	down
Pak1	-2.8437	0.009	0.2998	down
Inafm2	-5.0542	0.0092	0.3	down
Ctps2	-2.0358	0.0094	0.3018	down
Zbtb12	-1.9536	0.0094	0.3018	down
Oxct1	-3.0384	0.0095	0.3024	down
Suds3	-3.805	0.0098	0.3056	down
Gm10443	-1.7965	0.0098	0.3056	down
Klhl5	-2.8882	0.0099	0.3064	down
Rpl12	4.6306	0	0.0977	up
Flrt1	3.5892	0	0.0977	up
Aldh1a1	2.3893	0	0.0994	up
Cep250	3.0405	1.00E-04	0.0994	up
Sptbn2	2.9125	1.00E-04	0.0994	up
Castor2	4.0908	1.00E-04	0.0994	up
Eef1a1	4.3894	1.00E-04	0.0994	up
Adarb1	4.963	1.00E-04	0.0994	up
Adcy9	2.5923	1.00E-04	0.0994	up
Nudt9	5.13	1.00E-04	0.0994	up
Chd5	2.3371	1.00E-04	0.0994	up
Gpatch2l	6.1997	1.00E-04	0.0994	up
Elfn2	2.4621	1.00E-04	0.0994	up
Micall1	2.7847	1.00E-04	0.0994	up
Arrb1	3.5129	2.00E-04	0.0994	up
Fam120c	2.5385	2.00E-04	0.0994	up
Snx27	3.9786	2.00E-04	0.0994	up
Atxn7	3.6623	2.00E-04	0.0994	up
Rpl26	2.8587	2.00E-04	0.0994	up

Coro1c	5.7541	2.00E-04	0.0994	up
Brsk2	4.1803	2.00E-04	0.0994	up
Sacm1l	4.1619	2.00E-04	0.1001	up
Tpgs1	3.4861	2.00E-04	0.1075	up
Commd9	3.253	2.00E-04	0.1075	up
Rps2	3.8741	3.00E-04	0.1083	up
Rpl10	3.0158	3.00E-04	0.1083	up
Igf1r	4.6255	3.00E-04	0.1083	up
Garre1	2.4376	3.00E-04	0.1231	up
Tbc1d30	3.6223	3.00E-04	0.1237	up
Eci1	3.3892	3.00E-04	0.1237	up
Tecpr2	2.1655	3.00E-04	0.1237	up
Rps5	2.9878	4.00E-04	0.1268	up
Tro	3.6639	4.00E-04	0.1273	up
Slc16a1	2.5116	4.00E-04	0.128	up
Fabp3	3.603	4.00E-04	0.1417	up
Hivep1	4.2335	5.00E-04	0.1417	up
Grip2	4.016	5.00E-04	0.1417	up
Mybl1	5.4093	5.00E-04	0.1458	up
Ramp1	4.9053	5.00E-04	0.1458	up
Usp47	3.8456	5.00E-04	0.1458	up
Lmtk2	3.0711	5.00E-04	0.1458	up
Ppm1f	3.173	6.00E-04	0.1529	up
Rpl23a	2.7184	6.00E-04	0.1532	up
Myo18a	2.3843	6.00E-04	0.1579	up
Rbfox2	3.785	6.00E-04	0.1579	up
Ankrd52	1.7911	6.00E-04	0.1622	up
Map1a	1.9784	7.00E-04	0.1641	up
Bmp2k	4.6328	7.00E-04	0.1641	up
Herc2	3.203	7.00E-04	0.1667	up
Rpl27a	2.8918	7.00E-04	0.1684	up
Pcp4	4.6887	7.00E-04	0.1684	up
Rps10-ps2	3.2858	7.00E-04	0.1703	up
Ttc39c	4.9914	8.00E-04	0.1821	up
Add2	2.9064	8.00E-04	0.1821	up
Rpl35a	1.7834	8.00E-04	0.1821	up
Map3k9	3.5464	8.00E-04	0.1825	up
Cit	2.5535	9.00E-04	0.1843	up
Tbc1d14	3.4504	9.00E-04	0.1911	up
Srebf2	3.3233	9.00E-04	0.1911	up
Sparc	3.155	0.001	0.1911	up
Pdia4	4.7146	0.001	0.1911	up
Dpp8	2.9688	0.001	0.1911	up
Clstn2	4.7513	0.001	0.1911	up
Ubr2	2.368	0.0011	0.1911	up
Kcnq2	2.3885	0.0011	0.1911	up
Atp2a2	3.9054	0.0011	0.1911	up
Fam13b	1.8152	0.0011	0.1911	up
Itsn1	2.9205	0.0011	0.1911	up
Dpf1	3.8389	0.0011	0.1911	up
Atg101	4.734	0.0011	0.1911	up
Wdr35	2.9965	0.0011	0.1911	up
Zc3h13	2.9861	0.0012	0.1911	up
Rps21	2.1034	0.0012	0.1911	up
Atp2b3	3.1505	0.0012	0.1911	up
Asxl1	5.0981	0.0012	0.1911	up

Robo1	5.2402	0.0012	0.1911	up
Rgs9	3.1334	0.0012	0.194	up
Pfdn6	2.4357	0.0013	0.1989	up
Tubgcp6	1.856	0.0013	0.1989	up
Camsap2	2.667	0.0013	0.1989	up
Gstp1	2.1592	0.0013	0.1989	up
Gm49654	3.129	0.0013	0.1989	up
Spred3	3.2139	0.0013	0.1989	up
Mvb12b	3.3058	0.0014	0.1989	up
Eif3i	2.9682	0.0014	0.1989	up
Ypel2	3.2326	0.0014	0.1989	up
Ncoa2	3.3254	0.0014	0.1989	up
Rps18	2.695	0.0014	0.1989	up
Rps14	2.2739	0.0014	0.1989	up
Srrm1	3.2332	0.0014	0.1989	up
Rffl	2.3109	0.0015	0.202	up
Rps4x	2.1059	0.0015	0.202	up
Taf2	3.7017	0.0015	0.202	up
Rps3	2.2092	0.0016	0.202	up
Eef1b2	2.8404	0.0016	0.202	up
Hacd1	4.4035	0.0016	0.202	up
Crebbp	3.4907	0.0016	0.202	up
Focad	3.1393	0.0016	0.202	up
Rps20	2.8286	0.0016	0.202	up
B4galnt4	3.0143	0.0017	0.2043	up
Mpped2	5.7083	0.0017	0.2043	up
Rplp0	2.7895	0.0017	0.2054	up
Prex1	1.8563	0.0017	0.2054	up
Ppip5k1	3.3217	0.0018	0.2054	up
Rpl31-ps8	3.6998	0.0018	0.2054	up
Rpl9	2.4466	0.0018	0.2056	up
Srcin1	1.7983	0.0018	0.2056	up
Psmd6	2.9871	0.0018	0.206	up
Sc5d	3.2027	0.0018	0.206	up
Kpna6	2.8964	0.0018	0.2061	up
Map4k4	2.4708	0.0019	0.2131	up
Slc9a5	3.2224	0.002	0.2175	up
Anks1	2.129	0.002	0.2175	up
Gm10275	1.5259	0.002	0.2197	up
Tmppe	2.8287	0.002	0.2208	up
Jkamp	5.419	0.0021	0.2275	up
Nipal3	2.7245	0.0022	0.2278	up
Pik3c2a	3.3898	0.0022	0.2278	up
Kif13a	3.9134	0.0022	0.2278	up
Gm6136	2.471	0.0022	0.2278	up
Tomm7	2.1583	0.0023	0.2278	up
Crebl2	2.7932	0.0023	0.2278	up
Tppp3	1.9509	0.0023	0.2278	up
Ppp1ca	2.2113	0.0023	0.2278	up
Pcm1	2.6705	0.0023	0.2278	up
Vwa8	3.3694	0.0023	0.2278	up
Stard10	2.4818	0.0023	0.2278	up
Marchf6	2.5922	0.0023	0.2278	up
Rpl13	2.106	0.0024	0.2278	up
Lcmt1	2.8632	0.0024	0.2278	up
Gfpt1	2.5783	0.0024	0.2278	up

Faxc	2.0629	0.0025	0.2278	up
Rpl37	2.6789	0.0025	0.2278	up
Bsn	1.8584	0.0025	0.2278	up
Slc25a18	2.1772	0.0025	0.2278	up
Vps72	3.3309	0.0025	0.2278	up
Cnst	3.0879	0.0025	0.2294	up
Nuak1	3.0379	0.0026	0.2321	up
Timp4	4.7384	0.0026	0.2342	up
Sypl	4.2652	0.0027	0.2356	up
Dscaml1	2.0254	0.0027	0.2356	up
Mtmr4	2.7437	0.0027	0.2356	up
Pacsin1	2.9021	0.0027	0.2356	up
Bmf	3.9937	0.0027	0.2356	up
Hectd4	2.0337	0.0028	0.2356	up
Tjp1	2.2675	0.0028	0.2356	up
Cacna1i	3.039	0.0028	0.2364	up
Clptm1l	3.334	0.0028	0.2364	up
2610507B11Rik	2.7274	0.0029	0.2364	up
Arfgef2	3.0127	0.0029	0.2364	up
Lpin2	4.0179	0.0029	0.2364	up
Stk32a	2.2328	0.0029	0.2364	up
Sipa1l2	2.3928	0.0029	0.2364	up
Urod	2.0216	0.0029	0.2364	up
Lancl3	4.0586	0.003	0.2405	up
Zmat3	3.2671	0.003	0.2405	up
Hepacam	4.0868	0.003	0.2411	up
Clic6	1.8977	0.0031	0.2411	up
Larp1	2.6145	0.0031	0.2426	up
Cmbl	4.1677	0.0032	0.2482	up
Slit2	2.4271	0.0032	0.2484	up
Tmem223	1.8816	0.0032	0.2488	up
Cntn4	3.2692	0.0033	0.2489	up
Ndst1	3.3597	0.0033	0.2494	up
Cntnap1	2.6483	0.0033	0.2512	up
Rae1	3.5168	0.0034	0.2512	up
Arih1	2.4374	0.0034	0.2512	up
Rims3	1.8449	0.0034	0.2512	up
Arhgdia	3.2015	0.0034	0.2512	up
Rbm28	3.2909	0.0035	0.2512	up
Xbp1	2.3353	0.0035	0.2519	up
Ankrd26	1.5545	0.0035	0.2519	up
Snx29	2.0598	0.0035	0.2519	up
Wdr3	4.3254	0.0036	0.2519	up
Zbtb4	2.4327	0.0036	0.2519	up
Nacc2	3.2932	0.0036	0.2519	up
Flot1	2.4333	0.0037	0.2519	up
Fry	2.8182	0.0037	0.2519	up
Padi2	3.1061	0.0037	0.2519	up
Prrc2b	1.4356	0.0037	0.2519	up
Larp1b	4.1445	0.0038	0.2519	up
Grcc10	2.9474	0.0038	0.2519	up
Chaserr	3.1713	0.0038	0.2519	up
Myrf	3.9211	0.0038	0.2519	up
Nit1	5.1965	0.0038	0.2519	up
Cst3	1.8945	0.0038	0.2519	up
Tnk2	2.1119	0.0038	0.2519	up

Amot	3.9746	0.0039	0.2519	up
Akr1a1	1.8539	0.0039	0.2519	up
Kcnc1	2.5573	0.0039	0.2519	up
Atrnl1	3.5298	0.004	0.2519	up
Arf3	3.5659	0.004	0.2519	up
Tmcc3	2.6087	0.004	0.2519	up
Nktr	2.2552	0.0041	0.2519	up
Zfp664	2.7153	0.0041	0.2519	up
Exosc2	3.5652	0.0041	0.2519	up
Fam13c	2.9845	0.0041	0.2519	up
Arl5a	2.7748	0.0041	0.2519	up
Rps3a1	2.6288	0.0042	0.2519	up
Rnh1	5.2826	0.0042	0.2519	up
Apol8	3.4465	0.0042	0.2519	up
Map4k2	3.5827	0.0042	0.2519	up
Ptpru	2.4017	0.0042	0.2519	up
Aip	2.3683	0.0042	0.2519	up
Rgs2	2.8082	0.0043	0.2519	up
2010204K13Rik	2.5372	0.0043	0.2519	up
Plekhh1	3.5822	0.0043	0.2532	up
Fam162a	3.1074	0.0043	0.2532	up
Commd8	5.8505	0.0043	0.2538	up
Plch2	2.3879	0.0044	0.2552	up
Hspa5	2.1359	0.0045	0.256	up
Hsp90ab1	3.1328	0.0045	0.256	up
Tmem167b	2.9698	0.0045	0.256	up
Vmac	3.0599	0.0045	0.256	up
Smg7	2.8973	0.0045	0.256	up
Zfyve1	6.0191	0.0045	0.256	up
Dnmt3a	2.1817	0.0045	0.256	up
Nsd1	3.3063	0.0046	0.2576	up
Cacna1g	2.2279	0.0046	0.2576	up
Cntn2	4.9962	0.0046	0.2576	up
Bicd2	1.967	0.0047	0.2586	up
Timm50	2.8402	0.0047	0.2597	up
Tspan14	3.3529	0.0048	0.2597	up
Fibp	3.3273	0.0048	0.2597	up
Dstyk	2.2321	0.0048	0.2597	up
Rnps1	2.9651	0.0048	0.2598	up
Dnajc16	2.789	0.0048	0.2598	up
Sstr5	2.4349	0.0048	0.2598	up
Dad1	2.4931	0.0048	0.2598	up
Ap1s2	2.8315	0.0049	0.2601	up
AY036118	5.1006	0.0049	0.2608	up
Pdxk	1.5071	0.0049	0.2608	up
Ndufa6	2.8937	0.005	0.2642	up
Rn18s-rs5	4.7351	0.005	0.2649	up
Phc3	3.1699	0.0051	0.2656	up
Ptprj	2.3176	0.0051	0.2663	up
Tmem63a	3.1555	0.0051	0.2663	up
Rps6	1.582	0.0052	0.2683	up
Rpl14-ps1	1.8091	0.0052	0.2683	up
Noc2l	2.4319	0.0053	0.2699	up
Celf1	2.8442	0.0053	0.2699	up
Chst8	3.3196	0.0053	0.2699	up
Sdhaf4	1.9762	0.0053	0.2704	up

Otud7b	3.7031	0.0054	0.2713	up
Pum3	4.4902	0.0054	0.2713	up
Rack1	1.9002	0.0055	0.273	up
Itga2b	3.0416	0.0055	0.273	up
Arpc2	2.2814	0.0056	0.273	up
Rab11fip5	2.808	0.0056	0.273	up
Efr3b	2.6427	0.0056	0.273	up
Cracd	2.0226	0.0056	0.273	up
Ubn1	1.4698	0.0056	0.2731	up
Syt5	4.5978	0.0056	0.2735	up
Dlc1	2.6446	0.0058	0.2748	up
Gm5124	3.5499	0.0058	0.2772	up
Npas3	2.4723	0.006	0.2821	up
Zfp688	3.0646	0.006	0.2821	up
Gpam	4.0531	0.006	0.2821	up
Nsmce1	2.421	0.006	0.2821	up
Arhgef25	2.8914	0.0061	0.2836	up
Hapln4	2.1066	0.0061	0.2836	up
Cluh	2.1582	0.0062	0.2836	up
Cd9	2.4999	0.0063	0.2836	up
Rab3gap2	3.0621	0.0063	0.2836	up
Tmed3	2.8088	0.0063	0.2836	up
Tm2d2	2.0415	0.0063	0.2836	up
Clmn	3.8366	0.0064	0.2836	up
Zfp2	5.1472	0.0064	0.2836	up
Tsr3	2.1192	0.0064	0.2836	up
Pcdh17	2.8208	0.0064	0.2836	up
Kcnc3	1.2849	0.0064	0.2836	up
Tmem115	4.3004	0.0064	0.2836	up
Rpl18	1.7687	0.0065	0.2847	up
Peli3	2.7463	0.0066	0.2847	up
Ndufb11	2.2103	0.0066	0.2847	up
Nr2c2	2.2339	0.0066	0.2847	up
Rprd1a	2.4107	0.0066	0.2847	up
Rnf167	2.1088	0.0066	0.2847	up
Copz1	3.3026	0.0067	0.2847	up
Serpinb1a	2.5998	0.0067	0.2847	up
Igsf21	3.0317	0.0068	0.2847	up
Cnot10	3.3333	0.0068	0.2847	up
Myh10	4.0595	0.0068	0.2847	up
Hcn2	3.8174	0.0068	0.2847	up
Trak2	5.446	0.0068	0.2847	up
Nxph4	1.9014	0.0068	0.2847	up
Dnttip1	2.6395	0.0068	0.2847	up
Zkscan16	1.957	0.0068	0.2847	up
Baz1b	2.6508	0.0069	0.2847	up
Crabp1	2.8537	0.0069	0.2852	up
Dynlt1b	2.7598	0.0069	0.2852	up
Mfsd2b	3.9364	0.007	0.2852	up
Cptp	2.3498	0.007	0.2852	up
Slit3	1.8042	0.007	0.2852	up
Cd63	1.9843	0.007	0.2852	up
Traf3	2.0478	0.0071	0.2852	up
Gpr88	2.011	0.0071	0.2852	up
Armcx4	2.0527	0.0071	0.2852	up
Gclm	3.1479	0.0072	0.2852	up

Btrc	2.8428	0.0072	0.2852	up
Nt5dc3	3.4016	0.0072	0.2852	up
Aatf	2.914	0.0072	0.2852	up
Luzp1	2.1983	0.0072	0.2852	up
Sf3b2	3.2157	0.0072	0.2852	up
Rab1b	3.3009	0.0072	0.2852	up
Sspo	3.4987	0.0072	0.2852	up
Klhl18	2.3113	0.0073	0.2852	up
Krtcap2	2.0234	0.0073	0.2852	up
Wapl	2.5659	0.0073	0.2852	up
Tril	2.2738	0.0074	0.2852	up
Dnajc13	3.2023	0.0074	0.2852	up
Pptc7	2.895	0.0074	0.2852	up
Rab11fip4	1.8894	0.0075	0.2852	up
Slmap	2.3247	0.0075	0.2852	up
Csgalnact2	3.9001	0.0075	0.2852	up
Tmem222	3.3754	0.0075	0.2852	up
Rab15	1.7854	0.0076	0.2882	up
Enpp4	2.4619	0.0077	0.2882	up
Ptpn11	1.8731	0.0077	0.2882	up
Nipsnap1	1.5664	0.0077	0.2882	up
Srp14	3.0039	0.0077	0.2882	up
Pacs2	4.0117	0.0077	0.2889	up
Rcor1	2.4897	0.0078	0.2908	up
Garem1	1.5811	0.0078	0.2908	up
Resp18	1.6888	0.0079	0.2924	up
Tex2	3.0598	0.008	0.2938	up
Grin2d	1.911	0.008	0.2938	up
Glg1	2.3304	0.008	0.2941	up
Ngb	3.6717	0.0081	0.2942	up
Pde4a	2.5089	0.0081	0.2944	up
Rictor	3.464	0.0081	0.2944	up
Nrgn	3.1469	0.0081	0.2944	up
Slc44a1	3.7921	0.0082	0.2944	up
Jade2	3.456	0.0082	0.295	up
1700123O20Rik	5.5614	0.0083	0.295	up
Ppm1e	3.6073	0.0083	0.295	up
Atm	2.2367	0.0083	0.295	up
Snrpa	2.3893	0.0083	0.295	up
Nelfb	3.1201	0.0083	0.295	up
St3gal1	2.6806	0.0083	0.295	up
Epb41l1	3.132	0.0083	0.295	up
Rplp2	1.837	0.0084	0.295	up
Il33	2.0459	0.0084	0.295	up
Rab8b	4.2351	0.0084	0.295	up
Caskin1	1.5179	0.0084	0.2952	up
Crim1	3.7301	0.0085	0.2952	up
Nipsnap2	3.8926	0.0085	0.2952	up
Rps19	1.3418	0.0085	0.2965	up
Tac1	4.2732	0.0085	0.2967	up
Auts2	2.0714	0.0086	0.2967	up
4930579G18Rik	2.5514	0.0087	0.2998	up
Fbxo2	2.2577	0.0088	0.2998	up
Ppm1h	3.2804	0.0088	0.2998	up
Tubgcp5	2.7101	0.0088	0.2998	up
Tmem205	2.4451	0.0088	0.2998	up

Cpsf4	2.7091	0.0088	0.2998	up
Gigyf2	2.8793	0.0089	0.2998	up
Plxna1	1.4821	0.0089	0.2998	up
Rbpj	2.6435	0.0089	0.2998	up
Rpl7	1.8495	0.0089	0.2998	up
Guk1	1.9681	0.0089	0.2998	up
Irx2	4.1929	0.009	0.2998	up
Smpd2	3.5665	0.009	0.2998	up
Rbbp7	3.5848	0.009	0.2998	up
Yipf1	3.6724	0.0091	0.2999	up
Mmd	2.4455	0.0091	0.2999	up
Fam214a	2.8175	0.0091	0.2999	up
Zfp410	2.8551	0.0091	0.2999	up
Psmd8	2.4896	0.0092	0.2999	up
Sbf1	1.7146	0.0092	0.2999	up
Kif13b	3.0176	0.0092	0.2999	up
Pex14	2.9793	0.0092	0.2999	up
Tdrp	2.6778	0.0092	0.3003	up
Net1	3.7904	0.0093	0.3015	up
Lrch3	2.0901	0.0093	0.3018	up
Pgam5	3.282	0.0094	0.3018	up
Erbb3	3.3766	0.0094	0.3018	up
Ankrd11	2.4303	0.0095	0.3024	up
Adcyap1r1	2.0246	0.0095	0.3024	up
Sik2	1.6246	0.0095	0.3031	up
Scaf11	3.8188	0.0096	0.305	up
Gm42853	2.3329	0.0096	0.305	up
Nol4	2.7907	0.0097	0.3053	up
Mea1	1.885	0.0097	0.3056	up
Unc13a	1.89	0.0097	0.3056	up
Mapk8ip3	1.603	0.0098	0.3056	up
Wdr18	3.4707	0.0098	0.3056	up
Nmur2	2.583	0.0098	0.3056	up
Cox19	3.2932	0.0098	0.3056	up
Rpl37a	1.3222	0.0099	0.3064	up
Celsr2	1.3652	0.0099	0.3066	up
**sham: TRF_A_VS_ALF_A**				
Mdga1	-5.1863	0	0.0879	down
Chgb	-4.0746	0	0.0879	down
Crat	-4.0436	0	0.1525	down
Fam210b	-3.3645	0	0.1525	down
Rn18s-rs5	-3.0522	1.00E-04	0.1525	down
Tmem11	-3.5791	1.00E-04	0.1525	down
Tceal9	-2.187	1.00E-04	0.1525	down
Vwf	-2.8283	1.00E-04	0.1525	down
Cby1	-3.9216	1.00E-04	0.1525	down
Clstn2	-5.7017	1.00E-04	0.1525	down
Dbp	-2.3985	1.00E-04	0.1525	down
Ube2v2	-2.789	1.00E-04	0.1525	down
Clcn2	-2.9747	2.00E-04	0.1525	down
Adamtsl4	-2.9847	2.00E-04	0.1525	down
Myrf	-3.1639	2.00E-04	0.1525	down
Zfpm1	-4.4573	2.00E-04	0.1525	down
Heyl	-3.2728	2.00E-04	0.1525	down
Slc2a8	-4.2242	2.00E-04	0.1748	down
Ntn1	-2.496	2.00E-04	0.1748	down

BC005624	-2.7699	2.00E-04	0.1748	down
Fxr1	-5.8442	2.00E-04	0.1748	down
Zfp36l2	-3.2142	3.00E-04	0.1822	down
Epb41l1	-3.2742	3.00E-04	0.1942	down
Ptrh2	-3.5016	3.00E-04	0.1942	down
Elfn1	-3.208	4.00E-04	0.1942	down
Dgkz	-8.2472	4.00E-04	0.1942	down
Bex4	-3.6791	4.00E-04	0.1942	down
Phldb1	-2.6264	4.00E-04	0.1942	down
Pum3	-4.521	4.00E-04	0.1942	down
Pfdn6	-2.9787	4.00E-04	0.1942	down
Ntng2	-4.7201	4.00E-04	0.1942	down
Scrt1	-2.2136	5.00E-04	0.1968	down
Grip2	-3.6657	5.00E-04	0.1968	down
Ppig	-3.0728	5.00E-04	0.1968	down
Map4k2	-4.3006	5.00E-04	0.1968	down
Dnaja1	-2.961	5.00E-04	0.1968	down
Col11a2	-2.2899	5.00E-04	0.1968	down
Gm2999	-2.1868	5.00E-04	0.1968	down
Rab11fip5	-3.0592	6.00E-04	0.1968	down
Cntnap1	-2.6408	6.00E-04	0.1968	down
Dnajc7	-2.9677	6.00E-04	0.1968	down
Arhgef10	-2.5363	6.00E-04	0.1968	down
Nudc	-2.9029	6.00E-04	0.1968	down
Prmt5	-4.633	6.00E-04	0.1968	down
Srp54c	-3.3438	6.00E-04	0.1968	down
Erbb3	-2.7232	7.00E-04	0.1968	down
Eif3f	-3.5129	7.00E-04	0.1968	down
Zfp385a	-4.514	7.00E-04	0.1968	down
Insm1	-2.7807	7.00E-04	0.1968	down
Safb	-3.4082	7.00E-04	0.1968	down
Ift57	-4.442	7.00E-04	0.1968	down
Larp7	-3.0961	7.00E-04	0.1973	down
Plcb4	-3.3394	7.00E-04	0.1973	down
Ppm1e	-3.237	8.00E-04	0.1999	down
Ace	-2.3825	8.00E-04	0.1999	down
Mylk	-1.8745	8.00E-04	0.1999	down
Inf2	-2.0369	8.00E-04	0.1999	down
Pcp4	-5.1857	8.00E-04	0.2011	down
Cluh	-3.0209	8.00E-04	0.2011	down
Nucb2	-2.4527	9.00E-04	0.2011	down
Fhl2	-3.1424	9.00E-04	0.2011	down
Spcs2	-2.2355	9.00E-04	0.2011	down
Igf1r	-4.3886	9.00E-04	0.2011	down
Mrpl24	-3.7122	9.00E-04	0.2011	down
Pdxk	-2.0459	9.00E-04	0.2011	down
Vtn	-1.3681	9.00E-04	0.2011	down
Ppm1g	-3.3685	9.00E-04	0.2011	down
Cnot10	-4.9278	0.001	0.2018	down
Rbbp7	-3.1479	0.001	0.2018	down
Hivep1	-4.0434	0.001	0.2018	down
Dkc1	-2.9423	0.001	0.2018	down
Git1	-4.1028	0.001	0.2084	down
Pdgfb	-2.5401	0.001	0.2084	down
Hs3st3a1	-4.2548	0.0011	0.2112	down
Fam102a	-2.9589	0.0011	0.2112	down

Snrnp27	-2.4518	0.0011	0.2112	down
Ddrgk1	-2.7835	0.0011	0.2152	down
Zrsr1	-2.7198	0.0012	0.2155	down
Hcn2	-4.3042	0.0012	0.2155	down
C1ql1	-1.9488	0.0012	0.2155	down
Eif3a	-6.2674	0.0012	0.2155	down
Grin2d	-2.1758	0.0012	0.2155	down
Fam53b	-2.0134	0.0012	0.2155	down
Ube2e1	-2.882	0.0013	0.2155	down
Creb3l1	-3.0192	0.0013	0.2155	down
Mvb12b	-3.2434	0.0013	0.2155	down
Tmem35a	-3.401	0.0013	0.2155	down
Dnajc8	-1.8822	0.0013	0.2155	down
Rgs2	-2.8175	0.0013	0.2171	down
Arhgap23	-1.7656	0.0014	0.2201	down
Akr1a1	-2.3576	0.0014	0.2236	down
Pacsin1	-3.3656	0.0015	0.2254	down
Castor2	-3.4356	0.0015	0.2254	down
ENSMUSG00000121141	-2.5781	0.0015	0.2254	down
Gabrb3	-2.8596	0.0015	0.2254	down
Tbc1d16	-2.0139	0.0016	0.2333	down
Tmem132e	-2.1561	0.0016	0.2378	down
Arhgdia	-3.0627	0.0017	0.2475	down
BC031181	-2.338	0.0017	0.2475	down
Nudt21	-3.8579	0.0018	0.2475	down
Ncbp3	-4.0183	0.0018	0.2475	down
Fnbp1	-2.9415	0.0018	0.2475	down
Tent2	-3.697	0.0018	0.2475	down
Snhg18	-3.6938	0.0019	0.2475	down
Lamtor2	-3.1333	0.0019	0.2475	down
Xrcc3	-2.4407	0.0019	0.2475	down
Suv39h1	-2.1995	0.0019	0.2482	down
Psme2b	-2.3618	0.0019	0.2482	down
Mcfd2	-2.9123	0.0019	0.2484	down
Yme1l1	-3.9718	0.002	0.2529	down
Timp4	-4.8958	0.002	0.2529	down
Selenoo	-2.1259	0.0021	0.2529	down
Itgb4	-1.874	0.0021	0.2529	down
Hapln4	-3.2559	0.0021	0.2529	down
Col18a1	-2.0351	0.0021	0.2529	down
Atp6v1g1	-3.1194	0.0021	0.2529	down
Ckap4	-4.3397	0.0021	0.2529	down
Gnl3	-2.3665	0.0021	0.2529	down
Strn4	-2.7244	0.0021	0.2533	down
Cfap418	-3.1684	0.0022	0.2553	down
Ap4s1	-2.7393	0.0022	0.2553	down
Pdia6	-2.1388	0.0022	0.2553	down
Mccc2	-5.2617	0.0023	0.2566	down
Zcchc7	-4.4185	0.0023	0.2566	down
Hnrnpm	-2.4279	0.0023	0.2566	down
Gadd45gip1	-2.8499	0.0024	0.2566	down
Wdr5	-3.4174	0.0024	0.2566	down
Eif1	-3.7921	0.0024	0.2566	down
Ypel2	-2.9728	0.0024	0.2566	down
Dok7	-3.0261	0.0024	0.2566	down
Aldh1a1	-1.6224	0.0024	0.2566	down

Srp54a	-5.3051	0.0024	0.2566	down
Herc3	-2.6995	0.0024	0.2566	down
Cav2	-2.6204	0.0025	0.2574	down
Rab33b	-4.2164	0.0025	0.2574	down
Mfap1a	-5.0297	0.0026	0.2653	down
Lpar6	-5.1047	0.0026	0.2653	down
Uba3	-1.8144	0.0026	0.2653	down
Prpf18	-2.6235	0.0027	0.2653	down
Slc12a5	-2.064	0.0027	0.2653	down
Rsl24d1	-3.6923	0.0027	0.2661	down
Gprasp1	-2.3934	0.0027	0.2672	down
Abcf3	-2.4284	0.0028	0.2672	down
Golga4	-3.7588	0.0028	0.2672	down
Gm4204	-1.5138	0.0028	0.2673	down
Picalm	-2.9169	0.0028	0.2673	down
Tsnax	-2.0149	0.0029	0.2673	down
Rps14	-2.1241	0.0029	0.2673	down
Kif13a	-3.2988	0.0029	0.2673	down
Gxylt1	-4.0565	0.0029	0.2673	down
Fbln5	-2.1439	0.003	0.2673	down
Atad2b	-3.1503	0.003	0.2673	down
Iqsec1	-2.6416	0.003	0.2673	down
Cnot7	-2.7718	0.003	0.2673	down
Ptcd2	-2.3639	0.003	0.2673	down
Vps4b	-2.7352	0.003	0.2673	down
Keap1	-2.2614	0.003	0.2673	down
Arpp19	-3.9223	0.0031	0.2673	down
Itm2a	-2.0867	0.0031	0.2673	down
Dach2	-4.0873	0.0031	0.2673	down
Sparc	-3.8991	0.0032	0.2733	down
B3galt6	-3.259	0.0032	0.275	down
Gdi1	-1.7525	0.0032	0.275	down
Aatk	-2.2513	0.0033	0.2824	down
Tceal3	-2.1661	0.0034	0.2824	down
Syt5	-3.5164	0.0034	0.2826	down
Pold3	-2.4739	0.0034	0.2847	down
Ei24	-3.7007	0.0035	0.2849	down
Gda	-2.0318	0.0035	0.2849	down
Gm10925	-4.2124	0.0035	0.2849	down
Rusc1	-2.5803	0.0035	0.2849	down
Plekhm2	-3.2919	0.0035	0.2849	down
Acta2	-1.7434	0.0035	0.2849	down
Sft2d1	-6.1484	0.0036	0.2849	down
Stac2	-2.0682	0.0036	0.2897	down
Fbxo44	-3.5976	0.0037	0.2943	down
Larp1b	-4.2947	0.0038	0.2943	down
Srrt	-2.8934	0.0038	0.2943	down
Smc1a	-3.4944	0.0039	0.2943	down
1110038B12Rik	-2.7138	0.0039	0.2943	down
Nectin1	-3.4576	0.0039	0.2943	down
Polb	-4.5367	0.0039	0.2943	down
Max	-2.1582	0.0039	0.2943	down
Cntn4	-3.6266	0.0039	0.2943	down
Eed	-3.335	0.0039	0.2943	down
Man1c1	-1.8676	0.0039	0.2943	down
Polr2g	-1.5147	0.004	0.2943	down

Smoc1	-1.3008	0.004	0.2943	down
Dync2i1	-2.5838	0.004	0.2943	down
Slc1a2	-2.5859	0.004	0.2948	down
Bex3	-1.6368	0.0041	0.2975	down
Tigd5	-3.3359	0.0041	0.2975	down
3110082I17Rik	-2.4791	0.0041	0.2975	down
Cmbl	-3.9814	0.0042	0.2975	down
Gprasp2	-3.0935	0.0043	0.2975	down
Prkdc	-3.6937	0.0043	0.2975	down
Rasd2	-2.4957	0.0044	0.2975	down
Hacd2	-3.0978	0.0044	0.2975	down
Atp13a2	-2.5547	0.0044	0.2975	down
Dph6	-6.2976	0.0044	0.2975	down
Dync1i2	-2.5479	0.0044	0.2975	down
Abt1	-2.2641	0.0045	0.2975	down
AI597479	-2.6137	0.0045	0.2975	down
Myl9	-1.4953	0.0045	0.2975	down
Syt12	-4.1687	0.0045	0.2975	down
Zcrb1	-1.6843	0.0045	0.2975	down
Ttc39c	-5.2133	0.0046	0.2975	down
Spred2	-2.943	0.0046	0.2975	down
Slc15a2	-2.9974	0.0046	0.2975	down
Twf1	-3.3999	0.0046	0.2975	down
Fgd1	-2.3909	0.0046	0.2975	down
Pja1	-2.1549	0.0046	0.2975	down
Prkce	-3.1038	0.0046	0.2975	down
Mgrn1	-2.9818	0.0046	0.2975	down
Pacs2	-2.8969	0.0047	0.2975	down
Tma7	-1.7843	0.0047	0.2975	down
C4b	-2.0091	0.0047	0.2975	down
Mat2a	-2.215	0.0047	0.2975	down
Lin7c	-3.0712	0.0047	0.2975	down
Rnf123	-5.2958	0.0048	0.2975	down
Sppl3	-2.5387	0.0048	0.2975	down
Igsf21	-3.8642	0.0048	0.2975	down
Ddx27	-2.5119	0.0049	0.2975	down
Arfgap2	-2.5628	0.0049	0.2975	down
Cntn2	-3.7626	0.0049	0.2975	down
Alkbh6	-2.3257	0.0049	0.2975	down
Gm13716	-3.1844	0.0049	0.2975	down
Syn1	-3.1967	0.0049	0.2975	down
Zfp777	-3.8194	0.0049	0.2975	down
Morf4l2	-3.5025	0.005	0.2975	down
Psmd8	-2.7899	0.005	0.2975	down
Gm5417	-4.3103	0.005	0.2975	down
Gpatch11	-3.3892	0.005	0.2975	down
Jup	-2.1293	0.0051	0.2975	down
Nsfl1c	-1.5475	0.0051	0.2975	down
Myh11	-1.6088	0.0051	0.2975	down
Ppp2r5b	-4.5618	0.0051	0.2987	down
Hnrnph1	-3.4354	0.0051	0.2995	down
Luc7l3	-2.0743	0.0052	0.2995	down
Sgce	-2.1963	0.0052	0.2995	down
Nkain1	-3.4472	0.0053	0.2995	down
Itgb8	-4.7196	0.0053	0.2995	down
Selenot	-2.7893	0.0053	0.2995	down

Lpin2	-4.3183	0.0053	0.2995	down
Hcn1	-2.3448	0.0053	0.2995	down
Slc6a9	-5.1163	0.0053	0.2995	down
Adra1b	-3.0451	0.0054	0.2995	down
Ccdc27	-3.7686	0.0054	0.2995	down
Paf1	-1.809	0.0054	0.2995	down
Plce1	-3.4953	0.0054	0.2995	down
C2cd5	-2.7219	0.0055	0.2995	down
Rgs7bp	-3.3752	0.0055	0.3	down
Chml	-4.3268	0.0055	0.3007	down
Alas1	-3.986	0.0055	0.3007	down
Klf15	-1.6953	0.0056	0.3007	down
Anapc13	-1.9257	0.0057	0.3051	down
Ppa2	-2.1686	0.0057	0.3055	down
Trappc12	-2.7574	0.0057	0.3055	down
Rhou	-3.3617	0.0057	0.3055	down
Srsf3	-3.8829	0.0058	0.3055	down
Frg1	-2.3323	0.0058	0.3055	down
Rgs20	-2.8666	0.0058	0.3055	down
Nt5dc3	-4.6987	0.0058	0.3055	down
Gpr37l1	-4.9326	0.0058	0.3056	down
Gm39244	-4.0862	0.0059	0.306	down
Pef1	-2.4904	0.0059	0.306	down
Prex1	-2.3636	0.0059	0.3064	down
Ipo7	-2.5907	0.0059	0.3064	down
Safb2	-2.1718	0.006	0.3064	down
Nudt11	-2.6954	0.006	0.3064	down
Tmem87b	-2.2798	0.006	0.3069	down
Btrc	-2.7437	0.006	0.3069	down
Bmp2k	-3.9679	0.0061	0.3099	down
Commd8	-6.5626	0.0061	0.3103	down
Sox17	-2.2045	0.0062	0.3108	down
Psmd11	-3.3673	0.0064	0.3179	down
Pak3	-3.3437	0.0064	0.3194	down
Sap30l	-1.9692	0.0064	0.3194	down
Carmil3	-3.8197	0.0064	0.3194	down
Lamc3	-2.0273	0.0066	0.3209	down
Ngb	-2.81	0.0066	0.3209	down
Cartpt	-2.7382	0.0066	0.3209	down
Jph3	-2.6256	0.0066	0.3209	down
Bcas3	-2.9254	0.0066	0.3209	down
Baz2b	-2.7407	0.0066	0.3209	down
Gabra5	-2.64	0.0067	0.3213	down
Lin37	-4.5749	0.0068	0.3262	down
Copb2	-2.3403	0.0068	0.3262	down
Cltb	-2.2629	0.0069	0.3271	down
Pnpt1	-2.5208	0.007	0.3271	down
Nell2	-4.8075	0.007	0.3271	down
Usp33	-3.5741	0.007	0.3271	down
Nsmf	-2.9526	0.007	0.3271	down
Kdsr	-2.5583	0.007	0.3271	down
Coro1c	-6.8056	0.0071	0.3286	down
Smdt1	-2.2877	0.0071	0.3287	down
Lrrn3	-2.0704	0.0071	0.3288	down
Gabrq	-3.2826	0.0072	0.3288	down
Use1	-2.6589	0.0072	0.3288	down

Prpf3	-2.485	0.0072	0.3288	down
Tmem9	-1.9332	0.0072	0.3288	down
Rai1	-1.6847	0.0072	0.3288	down
Kcnt1	-2.0356	0.0072	0.3288	down
Fbxl3	-3.017	0.0073	0.3303	down
Eipr1	-3.2791	0.0074	0.3319	down
Anapc11	-1.5268	0.0074	0.3319	down
Btbd2	-3.0362	0.0075	0.3319	down
Cwf19l1	-2.9156	0.0075	0.3319	down
mt-Co2	-1.6491	0.0075	0.3319	down
Ppp2r2c	-2.2954	0.0075	0.3319	down
Sema6c	-2.4234	0.0075	0.3319	down
Nbr1	-2.4331	0.0075	0.3319	down
Ap3m1	-2.5465	0.0075	0.3327	down
Gpsm1	-2.3268	0.0077	0.3349	down
Als2	-4.488	0.0077	0.3349	down
Abcb1a	-1.9094	0.0078	0.3349	down
Tshz2	-2.9312	0.0078	0.3349	down
Slc22a23	-2.0303	0.0079	0.3349	down
Taf1	-2.3351	0.0079	0.3349	down
Frmd8	-2.5073	0.0079	0.3349	down
Ubxn11	-2.9481	0.0079	0.3349	down
Gtf2h1	-2.0612	0.008	0.3349	down
Cetn2	-2.4531	0.008	0.3349	down
Sac3d1	-2.253	0.0081	0.3349	down
Polr2j	-2.2445	0.0081	0.3349	down
Fam183b	-2.9708	0.0081	0.3349	down
Wdr18	-4.6183	0.0081	0.3349	down
Nxpe4	-2.9965	0.0081	0.3349	down
Slc32a1	-1.1029	0.0081	0.3349	down
Car14	-1.8822	0.0081	0.3349	down
Hnrnph2	-2.6646	0.0081	0.3349	down
Papss2	-5.2878	0.0081	0.3349	down
Hepacam	-4.1648	0.0082	0.3349	down
Pcnx3	-1.8405	0.0082	0.3355	down
Brsk2	-5.0428	0.0082	0.3355	down
St3gal1	-4.0709	0.0082	0.3355	down
Jkamp	-5.2177	0.0083	0.3355	down
Tmem115	-3.1454	0.0083	0.3368	down
Drap1	-1.8928	0.0084	0.3368	down
Mgat3	-2.1857	0.0084	0.3371	down
Wapl	-2.9985	0.0085	0.3389	down
Capn2	-5.8508	0.0085	0.3389	down
Rgma	-3.7216	0.0085	0.3389	down
Plcd3	-2.5183	0.0086	0.3389	down
Nedd4l	-1.763	0.0086	0.3389	down
Cep170b	-1.9697	0.0086	0.3389	down
Mapre2	-3.6663	0.0086	0.3389	down
Sox1	-2.8261	0.0087	0.3401	down
Ddx5	-3.1484	0.0087	0.3401	down
Agk	-1.8594	0.0087	0.3401	down
Dusp8	-1.7634	0.0088	0.3411	down
Cacna1g	-1.8119	0.0088	0.3415	down
Sec31a	-4.057	0.0088	0.3415	down
Sdk1	-2.5232	0.0088	0.3417	down
Thoc3	-4.1798	0.0089	0.3417	down

Fam149b	-2.8291	0.0089	0.3418	down
Chchd2	-2.7696	0.0089	0.342	down
Asxl1	-4.9039	0.009	0.3444	down
Rhbdf1	-2.1133	0.0091	0.3482	down
Pdia3	-1.9371	0.0091	0.3482	down
Kpna3	-2.325	0.0092	0.3482	down
Hip1r	-1.8698	0.0093	0.3482	down
Sc5d	-3.2172	0.0093	0.3482	down
Neurl1a	-2.1235	0.0094	0.3482	down
Arl5a	-3.5343	0.0094	0.3482	down
Limk1	-2.8698	0.0094	0.3482	down
Eng	-1.6534	0.0094	0.3482	down
Sypl	-4.2966	0.0094	0.3482	down
Erp44	-2.3633	0.0094	0.3482	down
Tbk1	-2.7424	0.0095	0.3488	down
Rtl8c	-2.1578	0.0095	0.3502	down
Sphkap	-2.9981	0.0095	0.3502	down
Lmf2	-3.6489	0.0096	0.3502	down
Pnn	-2.0621	0.0097	0.3502	down
Pisd-ps2	-4.2605	0.0097	0.3502	down
Tmem200c	-1.4797	0.0097	0.3502	down
Abca2	-2.499	0.0097	0.3502	down
Atg4b	-3.0156	0.0098	0.3502	down
Vps52	-2.009	0.0098	0.3502	down
Prdx4	-2.2523	0.0098	0.3502	down
Zfp512	-2.1696	0.0098	0.3502	down
Fsd1l	-2.8354	0.0098	0.3502	down
Hnrnpu	-2.6216	0.0099	0.3502	down
Ccdc85c	-1.2725	0.0099	0.3502	down
Sox10	-1.6932	0.0099	0.3502	down
Pltp	-1.7092	0.0099	0.3502	down
Mob3b	-3.753	0.0099	0.3502	down
Dscaml1	-2.062	0.01	0.3502	down
Phc3	-3.8057	0.01	0.3504	down
Nr4a3	3.99	3.00E-04	0.1942	up
Ddc	2.8618	6.00E-04	0.1968	up
Mfap1b	1.7121	8.00E-04	0.1999	up
Fosl2	3.4547	0.0011	0.2112	up
Stk38	3.5363	0.0013	0.2155	up
Mcl1	4.4817	0.0016	0.2378	up
Gm12191	2.6313	0.0018	0.2475	up
Tmc7	3.6394	0.0018	0.2475	up
Mfsd5	3.3908	0.0021	0.2529	up
Jun	2.3264	0.0022	0.2553	up
Rnf41	3.2512	0.0023	0.2566	up
Junb	4.2854	0.0025	0.2574	up
Vrk1	3.7808	0.0026	0.2653	up
1700048O20Rik	3.5091	0.0029	0.2673	up
Jmjd6	2.5356	0.003	0.2673	up
Cdc42se1	3.363	0.0043	0.2975	up
Dusp6	2.3199	0.0044	0.2975	up
Prdm16	2.5201	0.0044	0.2975	up
Rgs5	1.9835	0.0046	0.2975	up
Plac9	1.718	0.0047	0.2975	up
Pwwp3a	3.9533	0.0047	0.2975	up
Ddx18	3.9968	0.005	0.2975	up

Alg9	2.7989	0.0052	0.2995	up
Wdr44	3.0252	0.0053	0.2995	up
Adgrl4	4.3536	0.0055	0.2995	up
Tnfrsf25	4.0223	0.0059	0.306	up
Garnl3	3.2953	0.0062	0.3125	up
Trim56	2.6904	0.0066	0.3209	up
Dedd	2.4734	0.0066	0.3209	up
Ciart	3.6078	0.0069	0.3271	up
Tes	2.7765	0.007	0.3271	up
4931406C07Rik	4.517	0.0076	0.3343	up
Trib1	3.2169	0.0077	0.3349	up
Chchd6	2.4303	0.0079	0.3349	up
Slc35d1	2.7282	0.0079	0.3349	up
Kri1	3.3013	0.0081	0.3349	up
Rasgrp3	4.2595	0.0083	0.3368	up
Gm11611	4.0814	0.0086	0.3389	up
Fzd1	1.5127	0.0092	0.3482	up
Otof	2.4713	0.0092	0.3482	up
Ing3	2.8529	0.0094	0.3482	up
Dmap1	3.6253	0.0097	0.3502	up
Mafg	4.8247	0.0099	0.3502	up

Supplementary Table 1: Total number of differentially expressed genes (DEGs) in different comparisons.
TRF = time-restricted feeding, ALF = ad libitum feeding, SNI = spared nerve injury, VS = versus, M = morning, A = afternoon, UP = upregulated, DOWN = downregulated.

Supplementary table 2: A list of DEGs in different comparisons.
TRF = time-restricted feeding, ALF = ad libitum feeding, SNI = spared nerve injury, VS = versus, M = morning, A = afternoon, UP = upregulated, DOWN = downregulated.

Supplementary Table 3: Differentially expressed genes related to circadian or mitochondrial function.

Supplementary Table 4: A list of differentially variable genes (DVGs) in different comparison.
LogVarRatio = ratio of the log change in group variance, TRF = time-restricted feeding, ALF = ad libitum feeding, SNI = spared nerve injury, VS = versus, M = morning, A = afternoon, up = increased variability, down = decreased variability.
